# Disrupting Kaposi’s Sarcoma-Associated Herpesvirus (KSHV) Latent Replication with a Small Molecule Inhibitor

**DOI:** 10.1021/acs.jmedchem.3c00990

**Published:** 2023-07-28

**Authors:** Aylin Berwanger, Saskia C. Stein, Andreas M. Kany, Melissa Gartner, Brigitta Loretz, Claus-Michael Lehr, Anna K. H. Hirsch, Thomas F. Schulz, Martin Empting

**Affiliations:** † Helmholtz-Institute for Pharmaceutical Research Saarland (HIPS), Campus E8.1, 66123 Saarbrücken, Germany; ‡ Department of Pharmacy, Saarland University, Campus E8.1, 66123 Saarbrücken, Germany; § German Centre for Infection Research (DZIF), Partner Site Hannover-Braunschweig, 66123 Saarbrücken, Germany; ∥ Institute of Virology, Hannover Medical School, Carl-Neuberg-Str. 1, 30625 Hannover, Germany; ⊥ Cluster of Excellence RESIST (EXC 2155), Hannover Medical School, Carl-Neuberg-Str. 1, 30625 Hannover, Germany

## Abstract

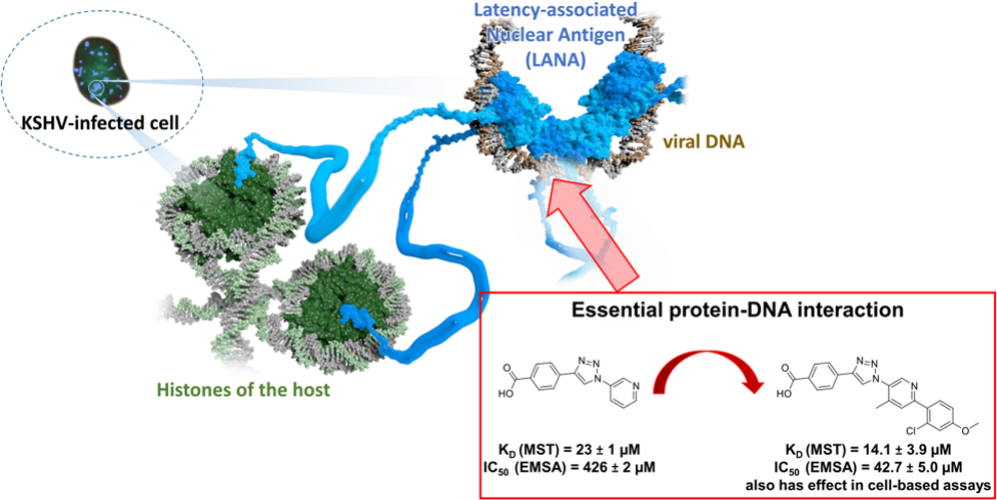

Kaposi’s sarcoma-associated herpesvirus (KSHV) can establish latent lifelong infections in infected individuals. During viral latency, the latency-associated nuclear antigen (LANA) mediates the replication of the latent viral genome in dividing cells and tethers them to mitotic chromosomes, thus ensuring their partitioning into daughter cells during mitosis. This study aims to inhibit Kaposi’s sarcoma-associated herpesvirus (KSHV) latent replication by targeting the LANA–DNA interaction using small molecular entities. Drawing from first-generation inhibitors and using growth vectors identified through STD-NMR, we expanded these compounds using Suzuki–Miyaura cross-coupling. This led to a deeper understanding of SAR achieved by microscale thermophoresis (MST) measurements and cell-free tests via electrophoretic mobility shift assays (EMSA). Our most potent compounds successfully inhibit LANA-mediated replication in cell-based assays and demonstrate favorable *in vitro* ADMET-profiles, including suitable metabolic stability, Caco-2 permeability, and cytotoxicity. These compounds could serve as qualified leads for the future refinement of small molecule inhibitors of KSHV latent replication.

## 1 Introduction

The majority of the human population is infected with at least one herpesvirus during their life span.^1,2^ Once infected, these pathogens will persist within the host in lifelong latent infections with potential acute lytic episodes.^3^ The family of herpesviruses can be divided into three different subfamilies: α-herpesviruses, β-herpesviruses, and γ-herpesviruses.^2,3^ Nine different herpesviruses have been discovered so far: herpes simplex virus (HHV-1 and HHV-2), varicella zoster virus (HHV-3), Epstein–Barr virus (HHV-4), cytomegalovirus (HHV-5), human herpesvirus 6 (HHV-6A and HHV-6B), human herpesvirus 7 (HHV-7), and human herpesvirus 8 (HHV-8), also known as Kaposi’s sarcoma-associated herpesvirus (KSHV).^2^ KSHV is classified as a rhadinovirus (γ_2_-herpesvirus) in the γ-herpesvirus subfamily.^2^ KSHV is the etiological agent of Kaposi’s sarcoma (KS) and of primary effusion lymphoma (PEL), as well as of many cases of the plasma cell variant of multicentric Castleman’s disease (MCD).^4−9^ The virus infects B-lymphocytes, epithelial cells, endothelial cells, dendritic cells, monocytes, and fibroblasts.^1,10,11^ It is found in all four epidemiological forms of KS: classic KS, endemic KS, iatrogenic KS, and epidemic KS/AIDS-KS.^12^ Classic KS affects older HIV-negative men, more frequently in case of a Jewish, Mediterranean, Eastern European, or Middle East background and typically affects the lower extremities.^10,12−14^ Endemic KS is a clinically more aggressive variant that occurs in HIV-negative people from East and Central sub-Saharan Africa.^12,13^ Iatrogenic KS affects immunosuppressed people, e.g., after an organ transplantation.^10,12,13^ AIDS-KS occurs in AIDS patients and still is one of the most frequent malignancies in this group of patients. Mucous membranes, lymph nodes, stomach, gut, lungs, and liver are often affected.^12,13,15^ After initial infection, KSHV establishes a latent infection in the host organism. In this phase, only a fraction of viral genes are expressed. In contrast, during the productive (“lytic”) phase of viral replication, many viral genes are expressed, and new infectious virus particles are produced. The switch from the latent into the lytic phase occurs spontaneously through various extracellular or intracellular triggers.^2,4,5,7,8,10,13^

Among the relatively few viral genes expressed in all latently infected cells are open reading frames (ORFs) 71, 72, 73, and a set of viral microRNAs.^16,17^ Depending on the cell type, additional viral genes, such as, e.g., vIRF3, may also be expressed during latency. ORF73 encodes for the latency-associated nuclear antigen (LANA).^8,18,19^ This protein is required for replication, persistence, and gene transcription of the viral KSHV genome as well as a stable segregation of the viral episome to the daughter cells during mitosis.^5,8,17,20^ LANA is found in all tumor cells infected with KSHV (KS, PEL, and MCD).^17,21^ In addition to its essential role during latent viral persistence, it has also been reported to act as an oncoprotein and to contribute to KSHV pathogenesis.^17,19,20,22^ The *C*-terminal end of LANA contains a DNA-binding domain (DBD), which binds to three different LANA-binding sites (LBS) found in each of the multiple terminal repeat subunits (TRs) flanking the long unique region (LUR) of the viral genome; these TRs serve as latent origins of replication.^19,20^ The *N-*terminal end of LANA contains a chromatin-binding domain (CBD), which mediates the tethering of LANA-occupied viral episomes to histones H2A and H2B on mitotic chromosomes.^5,19−21^

Hence, LANA is considered a promising drug target that offers the potential for LANA–DNA interaction inhibitors to impact the latent persistence of KSHV.

**Figure 1:**
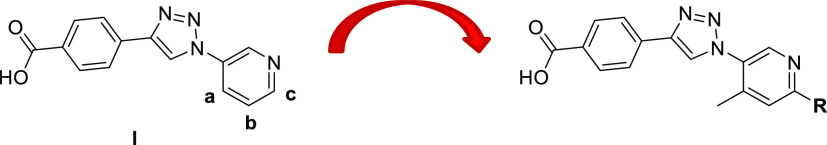
Compound growth of the previously identified Inhibitor **I**.

The work presented here is based on the previously reported hit scaffold Inhibitor **I** (see Figure 1).^23^ We have already made efforts to grow this inhibitor in three different directions. For the current series of compounds, we made use of Suzuki–Miyaura cross-couplings to enable facile SAR exploration. Compound affinities were determined via microscale thermophoresis (MST), while functional disruption of the LANA–DNA interaction was evaluated by an electrophoretic mobility shift assay (EMSA). Furthermore, we used a panel of *in vitro* assays to profile ADMET properties, including kinetic solubility, lipophilicity, metabolic stability, permeability (Caco-2), and cytotoxicity. Finally, we tested our best compounds for their ability to inhibit LANA-mediated replication of the viral latent replication origin in transfected cells to provide first evidence for their *in cellulo* activity.

## 2 Design Concept

The design of the new LANA inhibitors is based on previously identified growth vectors, which were deduced from STD-NMR spectra combined with docking studies.^23−25^

Additionally, the activity of Inhibitor **I** was improved by attaching a methyl group in *ortho-*position **a**, which hints at a steric *ortho-*effect.^25^ Now, further enlargement of Inhibitor **I** was achieved by applying Suzuki–Miyaura couplings in direction **c** (Figure 1).

## 3 Results and Discussion

### 3.1 Chemistry

**Scheme 1:**
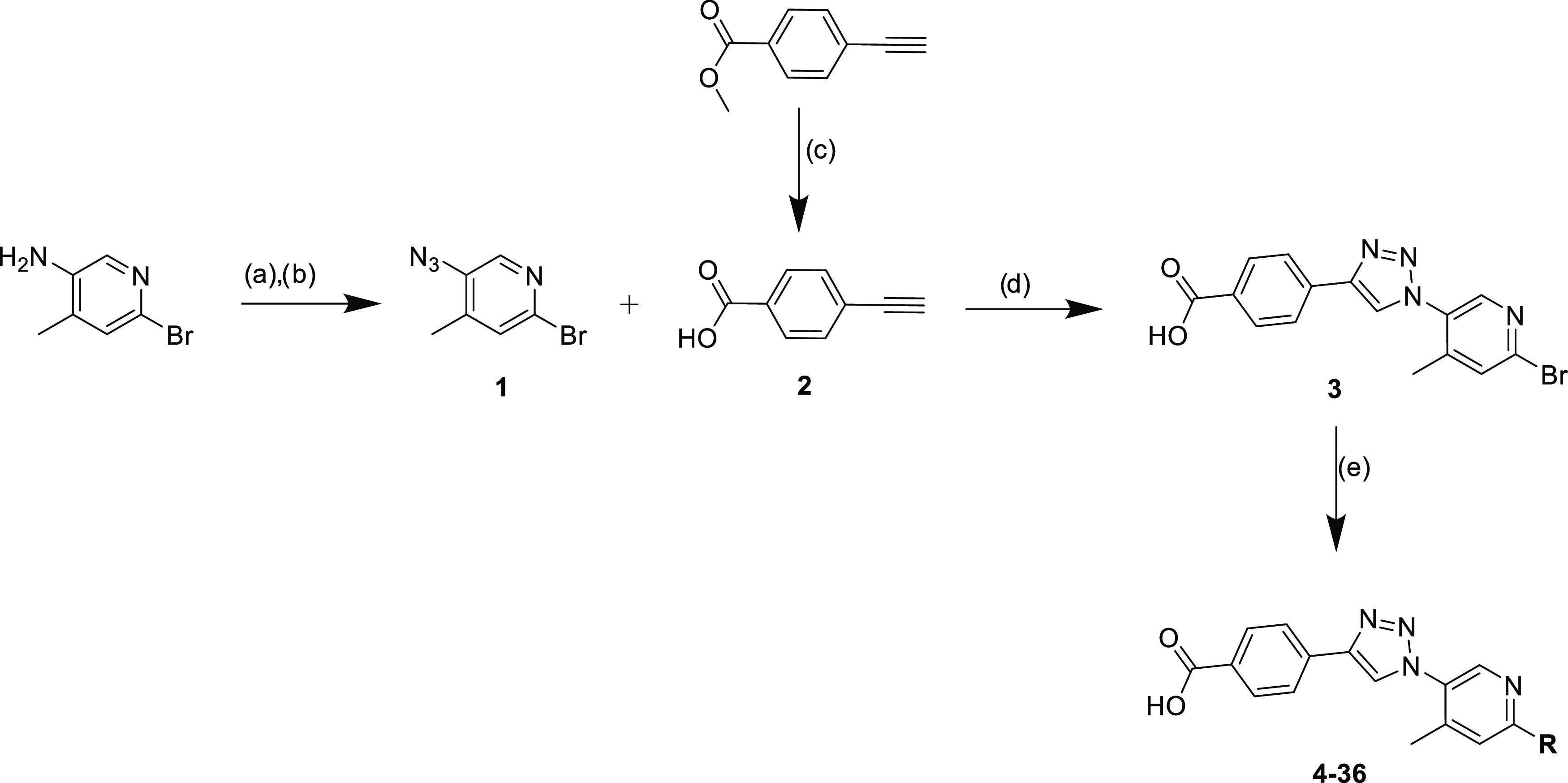
Synthesis of New Small Molecules via Click Chemistry and Suzuki–Miyaura Coupling **Reagents and conditions:** (a) 1.7 eq. NaNO_2_, 6M HCl, EtOAc, H_2_O, 0 °C, 30 min; (b) 1.7 eq. NaN_3_, rt, 1h; (c) 1 M NaOH, THF/MeOH 1:1, rt, overnight; (d) 2.0 eq. DIPEA, 0.5 eq. Na-ascorbate, 0.5 eq. CuSO_4_·5H_2_O, MeOH/H_2_O 1:1, argon, rt, overnight; (e) boronic acid/pinacol ester, 3.0 eq. Na_2_CO_3_, 0,1 eq. Pd(PPh_3_)_4_, H_2_O/1,4-dioxane 1:1, argon, 80 °C, overnight.

The synthesis of the compounds was carried out, as shown in Scheme 1. 5-Azido-2-bromo-4-methylpyridine **1** was synthesized by using a standard method for azidation with 5-Amino-2-bromo-4-methylpyridine, NaNO_2_, 6M HCl, and NaN_3_ in EtOAc.^25^ As the next step, a copper-catalyzed alkyne-azide cycloaddition (CuAAC) was carried out to obtain triazole **3** using azido-2-bromo-4-methylpyridine **1** and 4-ethynylbenzoic acid **2**, which was previously hydrolyzed by using methyl 4-ethynylbenzoate with 1 M NaOH in THF/MeOH.^25,26^ For derivatization in the final step, a Suzuki–Miyaura coupling was applied by using different boronic acids and boronic acid pinacol esters as well as Na_2_CO_3_ and tetrakis(triphenylphosphine)palladium(0) in water/1,4-dioxane.^25^ This resulted in 33 new small molecule inhibitors (**4–36**), which are listed in Table 1.

**Table 1:**
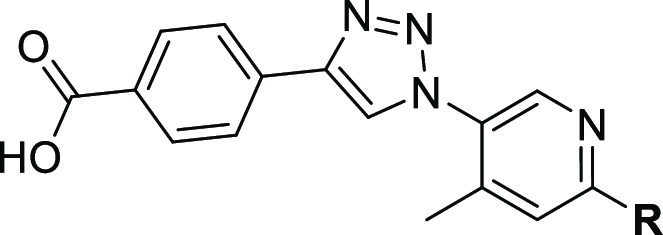

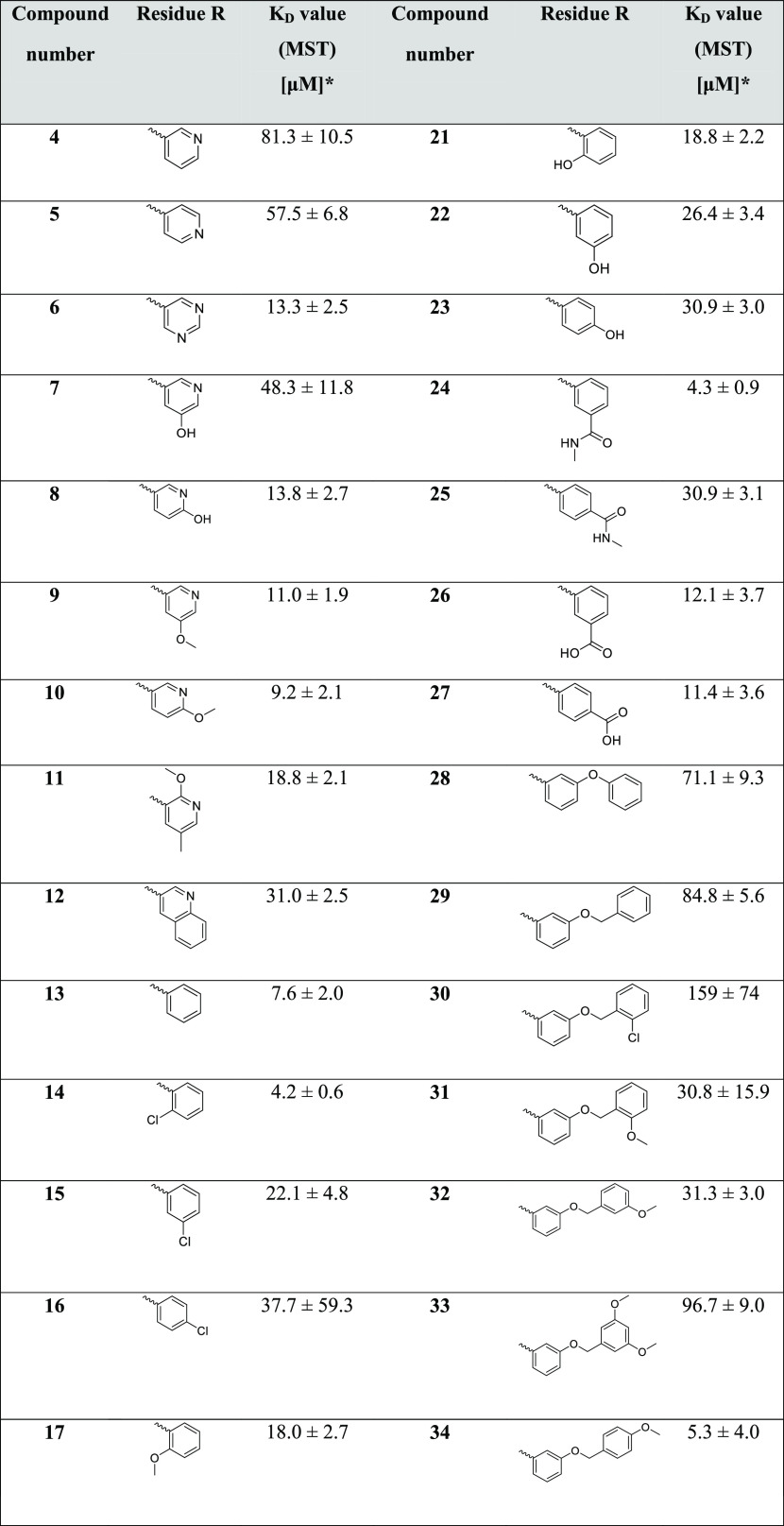

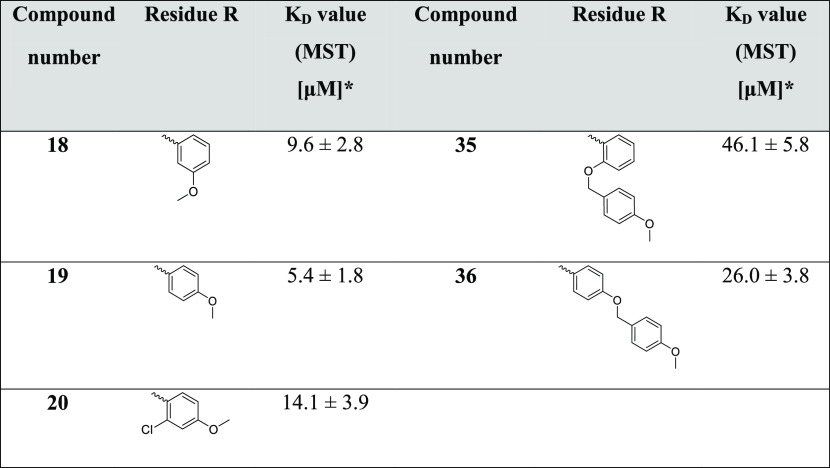
Synthesized Compounds via Suzuki–Miyaura Coupling and Their *K*_D_ Values Determined via MST^a^

a values are means of at least three replicates.

### 3.2 Biological Evaluation and SAR Studies

The new potential LANA–DNA inhibitors were tested for their binding affinity to LANA in a microscale thermophoresis (MST) assay as well as for their inhibition using an electrophoretic mobility shift assay (EMSA) at 250 μM. In these assays, an oligomerization-deficient mutant of a LANA *C*-terminal protein fragment (aa 1008–1146) and an oligonucleotide representing LANA-binding site (LBS) 1 were used, which are convenient to use and handle for the described biophysical methods, while maintaining the essential DNA-binding interface.^25^

All final compounds and their *K*_D_ values are listed in Table 1. Compounds with improved or similar binding affinity compared to hit **I** (*K*_D_ 23 μM^23^) were further selected for EMSA experiments as a second filter to screen LANA-binding compounds for their ability to disrupt the LANA–DNA interaction (Table 2). For further investigations, we selected all of the compounds that inhibited the LANA–DNA interaction in the EMSA assay by more than 50% at a concentration of 250 μM.

**Table 2:**
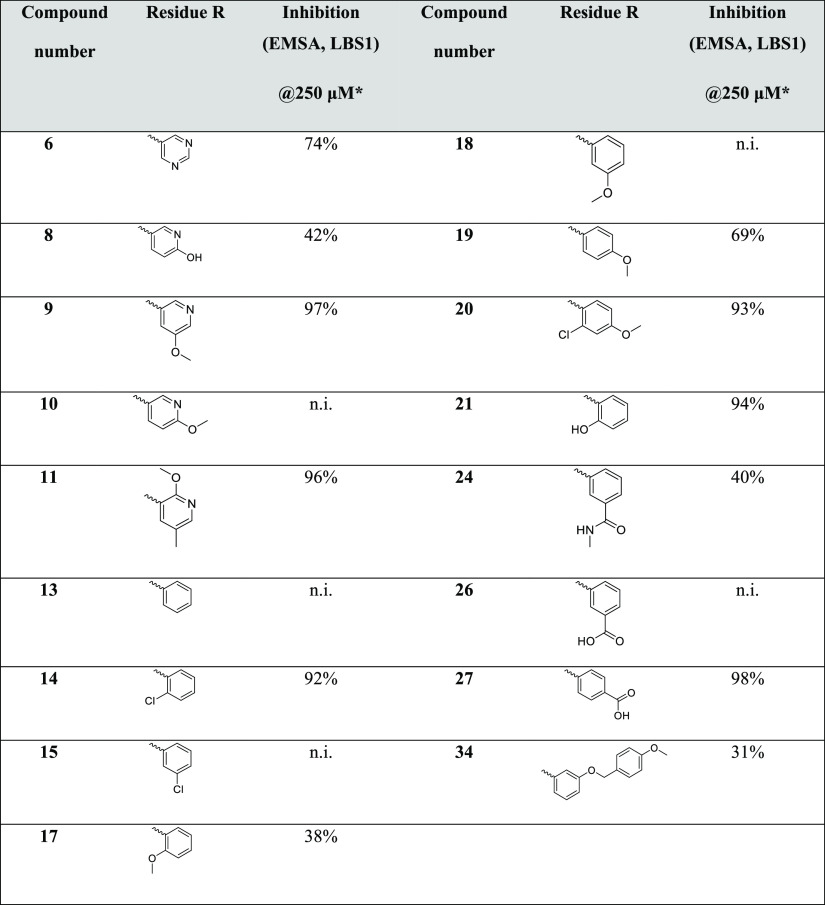
Evaluation of the Best LANA Binders in EMSA Experiments Using a Double-Stranded Oligonucleotide Representing LBS1 and a LANA Fragment (aa 1008–1146) Containing Mutations that Affect the Multimerization of the LANA DBD^a^,^b^

a Compounds were used at a concentration of 250 μM (n.i. = no inhibition for inhibition <10%).

b values are means of at least three replicates.

**Figure 2:**
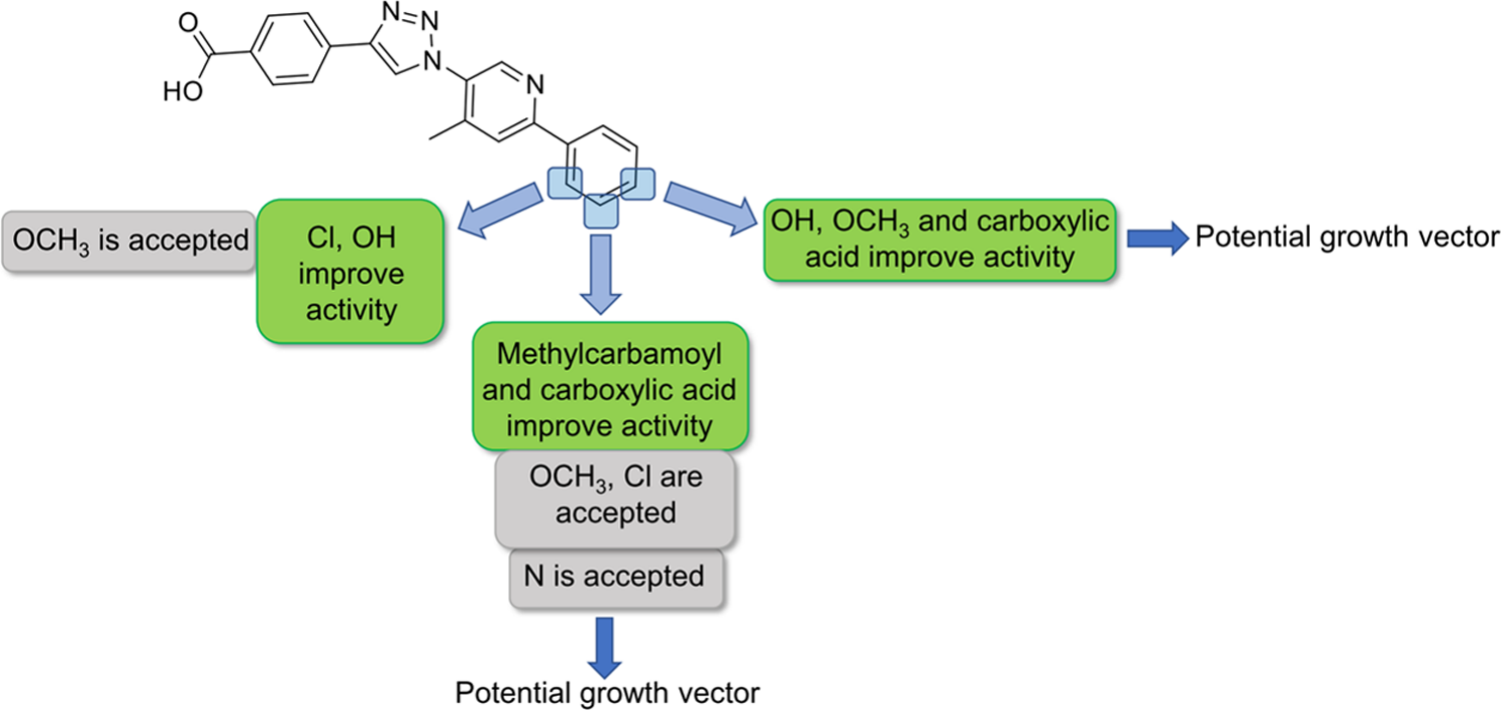
Summary of the SAR exploration.

For these SAR studies, we enlarged our previously found inhibitor in the eastern part of the molecule as we observed potential growth vectors in this direction.^23^ By attaching different phenyl and pyridine rings, we noticed that nitrogen in *meta-*position is accepted but not necessary for a good binding affinity and inhibition (see Figure 2).

In the *ortho-*position, methoxy groups retain activity, and chlorine as well as hydroxyl groups, will improve the activity. In the *meta-*position, we determined methylcarbamoyl and carboxylic acid groups as beneficial. Chlorine and methoxy groups do not impair activity. Hydroxyl, methoxy, and carboxylic acid groups will increase the activity in the *para-*position. All in all, we detected the *meta-* and *para-*positions as potential growth vectors. The substituents in the *ortho*-position might, again, induce a steric *ortho*-effect.

### 3.3 *In Vitro* ADMET Properties

The next step was an *in vitro* ADMET profiling of the best seven compounds so far: **9**, **11**, **14**, **19**, **20**, **21**, and **27**. The compounds were tested for their kinetic solubility (1% DMSO in PBS, pH 7.4), lipophilicity (chromatographic Log *D*_7.4_ value), cellular permeability (using Caco-2 cells), and metabolic stability (using mouse and human liver S9 fractions) (see Table 3).

**Table 3:**
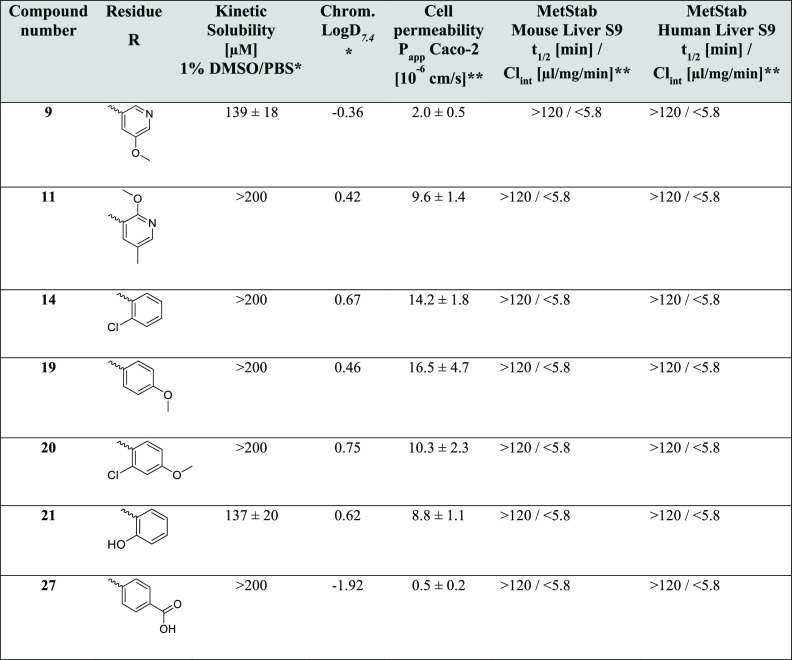
*In Vitro* ADMET Data for the Best Inhibitors^a^,^b^

a values are means of at least two replicates.

b values are means of at least three replicates.

All of the selected compounds showed a decent kinetic solubility in PBS buffer containing 1% DMSO of >100 μM. As expected, and in line with the excellent solubilities, the chromatographically determined Log *D*_7.4_ values characterizing these compounds as rather hydrophilic (Log *D*_7.4_ < 1), hinting at further opportunities for medicinal chemistry optimization exploring lipophilic interactions in the future. In the presence of mouse and human liver S9 fractions, the compounds were metabolically stable with *t*_1/2_ > 120 min. This finding, together with the high polarity of the compound class, hints at the dominance of renal elimination *in vivo* rather than hepatic metabolism. Importantly, Caco-2 permeability was high (Papp > 8 × 10^–6^ cm/s) for five of the seven compounds. The two compounds displaying significantly lower P_app_ were characterized by negative Log *D*_7.4_, making this a potential compound design tool to favor cell permeability. Therefore, these five compounds (**11**, **14**, **19**, **20**, and **21**) were selected for efficacy testing in a cell-based replication assay.

### 3.4 Cell-Based Assays

**Figure 3:**
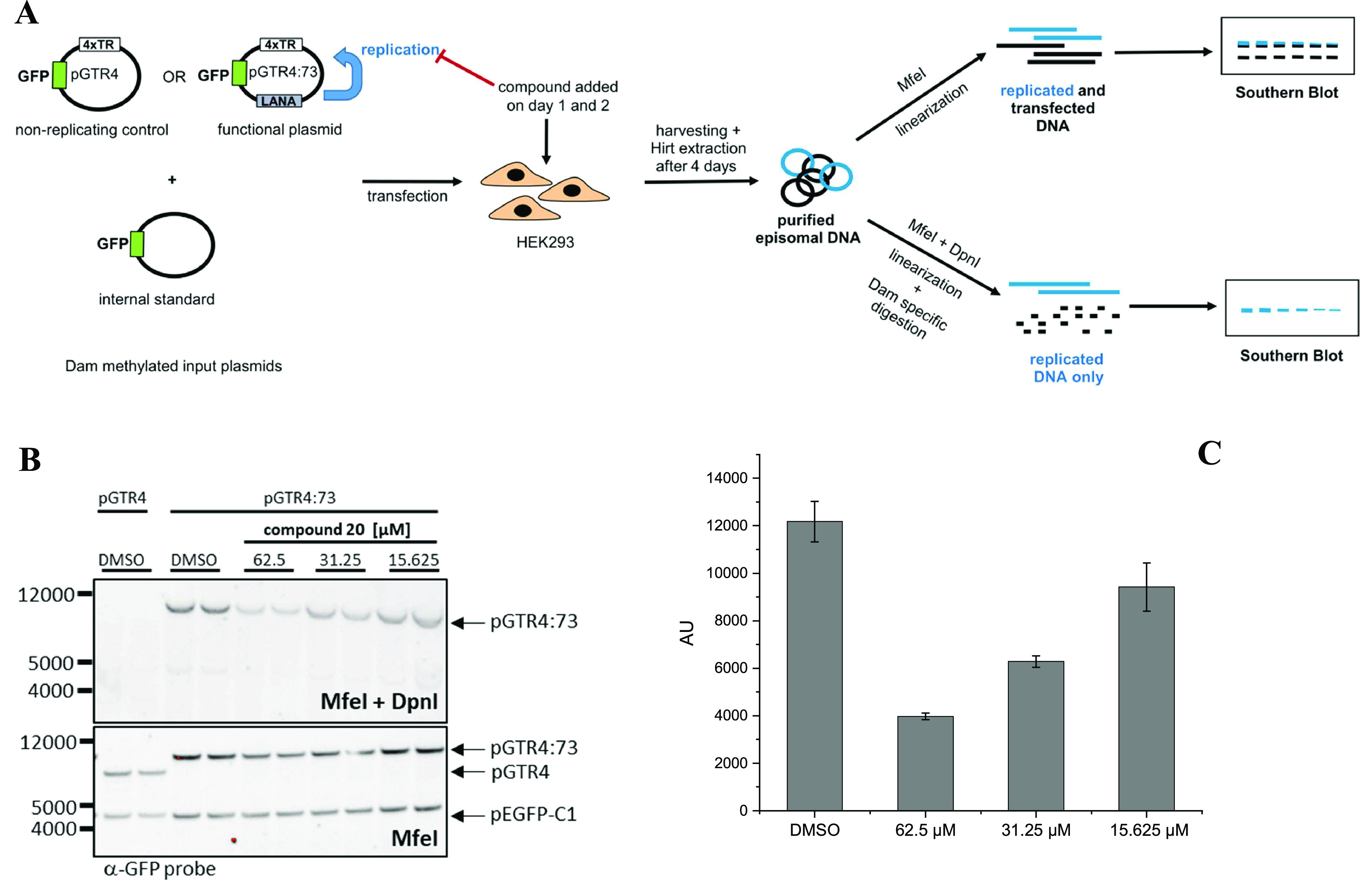
(A) Illustration of the replication assay. (B) Southern blot of extrachromosomal DNA extracted from transfected HEK293 cells and digested with *Mfe*I and *Dpn*I (this shows only the replicated pGTR4:73 plasmid, as it is resistant to *Dpn*I) and *Mfe*I (this shows both input and replicated plasmid) after three days of treatment with either DMSO or different concentrations of compound **20**. pGTR4:73 contains four terminal repeats (TR) and a LANA expression cassette. pGTR4 lacks the LANA expression cassette (nonreplicating control). pEGFP-1 serves as a nonreplicating transfection control. Replication assay shows a dose-dependent reduction in LANA-dependent replication of TR-containing plasmid in HEK293 cells treated with compound **20** (*Mfe*I + *Dpn*I). (C) Bar graph of the intensity of the pGTR4:73 band after digestion with *Mfe*I and *Dpn*I on the Southern blot shown in panel **B** at different concentrations for compound **20**. Values are means of two replicates.

After *in vitro* activity and ADMET profiling, we selected compounds for testing in an elaborated cell-based replication assay (Figure 3A–C).

For the replication assay (Figure 3A), plasmids containing four copies of the terminal repeat of the viral genome and a LANA expression cassette are transfected into HEK293 cells. The control represents a plasmid that does not contain a LANA expression cassette. The cells that can express LANA will now amplify the plasmids with the help of LANA and maintain them over several days of cell growth. Where LANA is not expressed, only the transfected plasmids remain and are slowly thinned out by cell growth. After transfection, cells are treated with the compounds or DMSO and allowed to grow for three days. Then, the plasmid DNA is isolated from the cell. The DNA is digested in two separate batches. First, to linearize them (*Mfe*I) and second, with an enzyme (*Dpn*I) that only digests DNA that has a specific methylation pattern derived from the bacteria, in which these plasmids had been amplified. The transfected DNA therefore has this pattern, while the DNA replicated in human cells does not have this modification, so it is not digested by *Dpn*I. One batch contains both enzymes, and only the replicated linearized DNA remains there. The second batch (control) contains only the linearizing enzyme. This shows us the input. The DNA is separated on a gel by electrophoresis and transferred to a membrane for immobilization (Southern blot). The DNA can then be visualized on this membrane using an enzyme-labeled probe, since the enzyme can generate chemiluminescence with a specific substrate. This assay is designed to specifically probe the LANA-mediated replication of plasmid DNA indicating on-target cellular activity. We performed the assay in a concentration range between 15.625 and 62.5 μM. For compound **20**, we observed the strongest inhibitory effect (Figure 3B,C). The observed disruption of LANA-mediated replication was concentration-dependent, resulting in a 67% inhibition at 62.5 μM. For this compound, we estimated an IC_50_ value of 33.2 ± 3.6 μM in the Southern blot.

## 4 Conclusions

In this study, we synthesized new LANA–DNA interaction inhibitors by applying Suzuki–Miyaura couplings. 33 different derivatives based on inhibitor **I** were obtained. The evaluation of affinity and *in cellulo* activity was done using an MST assay and EMSA experiments, respectively. A panel of *in vitro* ADMET studies was conducted with the seven most promising compounds showing overall suitable profiles with low lipophilicity combined with high solubility, permeability, and metabolic stability. The SAR exploration led to more potent inhibitors with *K*_D_ values in the low micromolar range and a slightly improved inhibition in EMSA experiments compared to the initial starting point. Importantly, we were able to show for the first time an on-target action in a cell-based replication assay for compound **20**. This effect hints at the potential to interfere with the latent life cycle of KSHV by small molecular entities and holds promise to eventually provide a true therapeutic approach by enabling not only treatment of the lytic stages but also potential removal of the virus from latently infected host cells, thereby potentially curbing viral persistence. This result represents an important guidepost toward the overarching goal of pursuing KSHV LANA as a druggable target. Due to the adequate *in vitro* ADMET properties and demonstrated cellular effect, the reported structures will serve as new starting points for the next optimization cycle, now focusing on optimizing the cellular effect.

## 5 Experimental Section

### 5.1 Chemistry

The chemicals were purchased from commercial suppliers. Preparative high-performance liquid chromatography (HPLC, UltiMate 3000 UHPLC+, Thermo Scientific) was used for the purification of the final compounds. Therefore, a reversed-phase column (VP 250/16 Nucleodur C18 Gravity, 5 μm, Macherey-Nagel, Germany) and water (containing 0.05% FA) and Acetonitril (containing 0.05% FA) as solvents were used. Reaction control was carried out by using TLC (TLC Silica Gel 60 F_254_ plates, Merck, Darmstadt, Germany) or by reversed-phase liquid chromatography–mass spectrometry (LC-MS, Thermo Scientific DIONEX UltiMate 3000). The purity of the final compounds was determined by LC-MS (Thermo Scientific DIONEX UltiMate 3000, wavelength: 254 nm) and was >95% for all compounds. The ^1^H- and ^13^C-NMR spectra were recorded on a Bruker UltraShield 500 Plus nuclear magnetic resonance spectrometer at 499.90 and 125.70 MHz, respectively. The spectra were evaluated with the software ACD/Spectrus Processor 2019.2.1. The signals were calibrated to the signal of the solvent DMSO-*d*_6_. The chemical shifts (δ) were given in parts per million (ppm), and the coupling constants (*J*) were given in hertz (Hz). Multiplicities are designated as: s: singlet, br.s.: broad singlet, d: doublet, dd: doublet of a doublet, t: triplet, m: multiplet. The spectra can be found in the Supporting Information. High-resolution masses (HRMS) were determined by LC-MS/MS using a Thermo Scientific Q Exactive Focus (Germany) with a DIONEX UltiMate 3000 UHPLC + focused.

#### 5.1.1 General Procedure 1 (GP1): Suzuki–Miyaura Coupling for Compounds **4–36**

1.0 eq. of the halogenated educt was solved in a mixture of 1,4-dioxane and water (1:1). This solution was flushed with argon. Then, 3.0 eq. Na_2_CO_3_, 2.0 eq. of the boronic acid or boronic acid pinacol ester, and 0.1 eq. Pd(PPh_3_)_4_ were added. The mixture was stirred at 80 °C overnight. After full conversion (LC-MS control), the reaction mixture was acidified with 1 M HCl (pH ∼ 1), and the precipitated product was isolated *via* vacuum filtration. The products were washed with water and dried under vacuum. The products were purified using preparative HPLC with solvents water (containing 0.05% FA) and MeCN (containing 0.05% FA) (gradient elution, MeCN:H_2_O 1:9 - > 9:1).

#### 5.1.2 Synthesis and Characterization of 5-Azido-2-bromo-4-methylpyridine (**1**)^25^

To a solution of the amine (100 mg; 0.53 mmol; 1.0 eq.) in EtOAc, H_2_O, and 6M HCl, 1.7 eq. of NaNO_2_ (62 mg) was added slowly at 0 °C. The reaction was stirred for 30 min at 0 °C. Then, sodium azide (59 mg; 1.7 eq.) was added at 0 °C, and the reaction was warmed up to room temperature. After full conversion (TLC control), the mixture was basified with sat. NaHCO_3_ to pH ∼ 10. The mixture was extracted three times with EtOAc, and the combined organic layers were dried over Na_2_SO_4_. The crude products were obtained after evaporation of the solvent (107 mg; 0.50 mmol; 94%).

TLC (PE (40–60 °C)/EtOAc 7:3): *R*_f_ = 0.58

LC-MS (ESI): [M + H]^+^ calcd: 212.98, found: 212.97

#### 5.1.3 Synthesis and Characterization of 4-Ethynylbenzoic Acid (**2**)^26^

1000 mg (6.2 mmol) of methyl 4-ethynylbenzoate was dissolved in 15 ml of THF/MeOH (1:1). Then, 7 ml of 1 M aq. NaOH was added slowly. The reaction mixture was stirred overnight at room temperature. The solvent was removed under reduced pressure. The residue was acidified with 1 M HCl to pH ∼ 1. The precipitated compound was filtered under reduced pressure and dried under vacuum to give 4-Ethylnylbenzoic acid (903 mg, 6.18 mmol, 99%). The crude product was used in the next step without further purification.

LC-MS (ESI): [M + H]^+^ calcd: 147.04, found: 147.06

^1^H-NMR (500 MHz, DMSO-*d*_6_, δ in ppm): 7.93 (m, 2H); 7.59 (m, 2H); 4.44 (s, 1H).

^13^C-NMR (126 MHz, DMSO-*d*_6_, δ in ppm): 166.8; 132.0; 130.9; 129.6; 126.1; 83.7; 82.9.

#### 5.1.4 Synthesis and characterization of 4-(1-(6-Bromo-4-methylpyridin-3-yl)-1H-1,2,3-triazol-4-yl)benzoic Acid (**3**)^25^

1.2 eq. (102 mg; 0.48 mmol) of the azide 1 was dissolved in a 1:1 mixture of H_2_O/MeOH under argon. 2.0 eq. (136 μL; 0.80 mmol) of DIPEA, 0.5 eq. (50 mg; 0.20 mmol) of CuSO_4_·5H_2_O, 0.5 eq. (40 mg; 0.20 mmol) of sodium ascorbate, and 1.0 eq. (58 mg; 0.40 mmol) of the alkine 2 were added. The reaction mixture was stirred at room temperature overnight. After full conversion (LC-MS control), 1 M HCl was added (pH ∼ 1), and the product was precipitated. To obtain the crude product (133 mg; 0.37 mmol; 93%), the solids were collected over vacuum filtration and dried under vacuum. The products were purified using preparative HPLC with solvents water (containing 0.05% FA) and MeCN (containing 0.05% FA) (gradient elution, MeCN:H_2_O 1:9 - > 9:1).

HRMS (ESI): [M + H]^+^ calcd: 359.01381, found: 359.01232

^1^H-NMR (500 MHz, DMSO-*d*_6_, δ in ppm): 9.18 (s, 1H); 8.60 (s, 1H); 8.07 (m, 4H); 7.94 (s, 1H); 2.31 (s, 3H).

^13^C-NMR (126 MHz, DMSO-*d*_6_, δ in ppm): 147.0; 146.7; 146.5; 142.5; 134.4; 133.7; 130.5; 124.9; 17.5.

Further experimental details, as well as biological assay description and data, are provided in the Supporting Information.

## Supporting Information Available

Further experimental details, supplementary data, including, e.g., NMR and MS spectra, MST traces, etc., (PDF)
List of molecular formula strings and the associated biochemical and biological data (CSV)

## Abbreviations Used

CBD: chromatin-binding domain
CuAAC: copper-catalyzed alkyne-azide-cycloaddition
DBD: DNA-binding domain
DIPEA: *N,N*-Diisopropylethylamine
EMSA: electrophoretic mobility shift assay
EtOAc: ethyl acetate
HHV: human herpesvirus
*K*_D_: dissociation constant
KS: Kaposi’s sarcoma
KSHV: Kaposi’s sarcoma-associated herpesvirus
LANA: latency-associated nuclear antigen
LBS: LANA-binding site
Log *D*: distribution coefficient
LUR: long unique region
MCD: multicentric Castleman’s disease
MeCN: Acetonitril
MeOH: methanol
MST: microscale thermophoresis
ORF: open reading frame
P_app_: apparent permeability coefficient
PE: petrol ether
PEL: primary effusion lymphoma
STD: saturation transfer difference
TR: terminal repeat
