# Supplementary material for: Disrupting Kaposi’s Sarcoma-Associated Herpesvirus (KSHV) Latent Replication with a Small Molecule Inhibitor
Source: J Med Chem. 2023 Jul 28;66(15):10782–90. doi: 10.1021/acs.jmedchem.3c00990 (PMC10424179; doi:10.1021/acs.jmedchem.3c00990)
Supplement: Supplementary file 2 — jm3c00990_si_002.pdf [file jm3c00990_si_002.pdf]

Supporting Information

Disrupting Kaposi's sarcoma-associated herpesvirus (KSHV) latent replication with a small molecule inhibitor

Aylin Berwanger[1,2,3], Saskia C. Stein[3,4,5], Andreas M. Kany[1], Melissa Gartner[2], Brigitta Loretz[1], Claus-Michael Lehr[1,2], Anna K. H. Hirsch[1,2,5], Thomas F. Schulz*[,3,4,5], Martin Empting*[,1,2,3,5]

[1]Helmholtz-Institute for Pharmaceutical Research Saarland (HIPS), Campus E8.1, 66123 Saarbrücken, Germany.

[2]Department of Pharmacy, Saarland University, Campus E8.1, 66123 Saarbrücken, Germany.

[3]German Centre for Infection Research (DZIF), Partner Site Hannover-Braunschweig, 66123 Saarbrücken, Germany.

[4]Institute of Virology, Hannover Medical School, Carl-Neuberg-Strasse 1, 30625 Hannover, Germany.

[5]Cluster of Excellence RESIST (EXC 2155), Hannover Medical School, Carl-Neuberg-Str. 1, 30625 Hannover, Germany.

Corresponding Authors:

Martin.Empting@helmholtz-hips.de; Schulz.Thomas@mh-hannover.de

Table of Contents

1. Materials and Methods S2
   1.1 Chemistry S2
   1.2 Protein Expression S14
   1.3 Microscale Thermophoresis (MST) Assay S15
   1.4 Electrophoretic Mobility Shift Assay (EMSA) S20
   1.5 Kinetic Solubility S22
   1.6 Chromatographic LogD S22

1.7 Metabolic Stability in Liver S9 Fractions S22
1.8 Cytotoxicity S23
1.9 Cell permeability S24
1.10 Replication assay S25
2. ^{1}H and ^{13}C Spectra S27
3. High Resolution Mass Spectra S59
4. HPLC Traces S74
5. References S77

1. Materials and Methods

1.1 Chemistry

Synthesis and characterization of 4-(1-(4-methyl-[2,3'-bipyridin]-5-yl)-1H-1,2,3-triazol-4-yl)benzoic acid (4)

The triazole was synthesized according to **GP1** using 70 mg (0.19 mmol) of the triazole **3**, 60 mg (0.57 mmol) of Na_2CO_3, 47 mg (0.38 mmol) of Pyridin-3-yl-boronic acid and 0.1 eq. of $Pd(PPh_3)_4$. 67 mg (0.19 mmol; 99%) of the crude product were obtained.

HRMS (ESI): $[M+H]^+$ calculated: 358.12985 found: 358.12815

^{1}H-NMR (500 MHz, DMSO-*d6*, *δ* in ppm): 9.24 (s, 1H); 8.86 (s, 1H); 8.54 (d, *J* = 8.1 Hz, 1H); 8.31 (s, 1H); 8.09 (m, 5H); 7.60 (m, 2H); 2.42 (s, 3H).

^{13}C-NMR (126 MHz, DMSO-*d6*, *δ* in ppm): 167.2; 155.0; 146.2; 146.1; 143.8; 134.4; 132.9; 130.3; 125.6; 124.5; 123.3; 17.6.

Synthesis and characterization of 4-(1-(4-methyl-[2,4'-bipyridin]-5-yl)-1H-1,2,3-triazol-4-yl)benzoic acid (5)

The triazole was synthesized according to **GP1** using 70 mg (0.19 mmol) of the triazole **3**, 60 mg (0.57 mmol) of Na_2CO_3, 78 mg (0.38 mmol) of 4-Pyridineboronic acid pinacol ester and 0.1 eq. of $Pd(PPh_3)_4$. 67 mg (0.19 mmol; 99%) of the crude product were obtained.

HRMS (ESI): $[M+H]^+$ calculated: 358.12985 found: 358.12809

^{1}H-NMR (500 MHz, DMSO-*d6*, *δ* in ppm): 9.24 (s, 1H); 8.90 (s, 1H); 8.77 (m, 2H); 8.38 (s, 1H); 8.16 (m, 2H); 8.06 (m, 4H); 2.45 (s, 3H).

^{13}C-NMR (126 MHz, DMSO-*d6*, *δ* in ppm): 154.2; 150.5; 146.1; 144.4; 143.7; 133.5; 130.1; 125.2; 124.2; 123.7; 121.0; 17.5.

Synthesis and characterization of 4-(1-(4-methyl-6-(pyrimidin-5-yl)pyridin-3-yl)-1H-1,2,3-triazol-4-yl)benzoic acid (6)

The triazole was synthesized according to **GP1** using 70 mg (0.19 mmol) of the triazole **3**, 60 mg (0.57 mmol) of Na_2CO_3, 47 mg (0.38 mmol) of Pyrimidin-5-yl-boronic acid and 0.1 eq. of $Pd(PPh_3)_4$. 68 mg (0.19 mmol; 99%) of the crude product were obtained.

HRMS (ESI): $[M+H]^+$ calculated: 359.12510 found: 359.12354

^{1}H-NMR (500 MHz, DMSO-*d6*, *δ* in ppm): 9.54 (s, 2H); 9.33 (s, 1H); 9.22 (s, 1H); 8.92 (s, 1H); 8.41 (s, 1H); 8.04 (m, 4H); 2.44 (s, 3H).

^{13}C-NMR (126 MHz, DMSO-*d6*, *δ* in ppm): 166.9; 158.7; 155.0; 152.0; 146.1; 145.9; 143.6; 133.9; 133.0; 130.7; 130.0; 125.2; 124.2; 123.4; 17.4.

Synthesis and characterization of 4-(1-(5'-hydroxy-4-methyl-[2,3'-bipyridin]-5-yl)-1H-1,2,3-triazol-4-yl)benzoic acid (7)

The triazole was synthesized according to **GP1** using 120 mg (0.33 mmol) of the triazole **3**, 105 mg (0.99 mmol) of Na_2CO_3, 92 mg (0.66 mmol) of (5-Hydroxypyridin-3-yl)boronic acid and 0.1 eq. of $Pd(PPh_3)_4$. 109 mg (0.29 mmol; 88%) of the crude product were obtained.

HRMS (ESI): $[M+H]^+$ calculated: 374.12477 found: 374.12444

^{1}H-NMR (500 MHz, DMSO-*d6*, *δ* in ppm): 9.22 (s, 1H); 8.84 (m, 2H); 8.25 (d, 2H); 8.07 (m, 4H); 7.91 (s, 1H); 2.40 (s, 3H).

^{13}C-NMR (126 MHz, DMSO-*d6*, *δ* in ppm): 154.3; 154.1; 145.7; 143.1; 138.7; 138.5; 133.5; 132.3; 129.8; 124.9; 123.9; 122.7; 119.6; 17.1.

Synthesis and characterization of 4-(1-(6'-hydroxy-4-methyl-[2,3'-bipyridin]-5-yl)-1H-1,2,3-triazol-4-yl)benzoic acid (8)

The triazole was synthesized according to **GP1** using 70 mg (0.19 mmol) of the triazole **3**, 60 mg (0.57 mmol) of Na_2CO_3, 84 mg (0.38 mmol) of 6-Hydroxypyridine-3-boronic acid pinacol ester and 0.1 eq. of $Pd(PPh_3)_4$. 70 mg (0.19 mmol; 99%) of the crude product were obtained.

HRMS (ESI): $[M+H]^+$ calculated: 374.12477 found: 374.12321

^{1}H-NMR (500 MHz, DMSO-*d6*, *δ* in ppm): 9.18 (s, 1H); 8.69 (s, 1H); 8.28 (m, 2H); 8.07 (m, 5H); 6.50 (d, *J* = 10.4 Hz, 1H); 2.34 (s, 3H).

^{13}C-NMR (126 MHz, DMSO-*d6*, *δ* in ppm): 167.1; 163.2; 146.4; 146.1; 137.2; 134.3; 130.3; 125.5; 124.5; 123.7; 17.6.

Synthesis and characterization of 4-(1-(5'-methoxy-4-methyl-[2,3'-bipyridin]-5-yl)-1H-1,2,3-triazol-4-yl)benzoic acid (9)

The triazole was synthesized according to **GP1** using 70 mg (0.19 mmol) of the triazole **3**, 60 mg (0.57 mmol) of Na_2CO_3, 89 mg (0.38 mmol) of 3-Methoxypyridine-5-boronic acid pinacol ester and 0.1 eq. of $Pd(PPh_3)_4$. 73 mg (0.19 mmol; 99%) of the crude product were obtained.

HRMS (ESI): $[M+H]^+$ calculated: 388.14042 found: 388.13857

^{1}H-NMR (500 MHz, DMSO-*d6*, *δ* in ppm): 9.28 (s, 1H); 8.90 (s, 1H); 8.45 (m, 2H); 8.08 (m, 5H); 4.03 (s, 3H); 2.44 (s, 3H).

^{13}C-NMR (126 MHz, DMSO-*d6*, *δ* in ppm): 167.5; 155.8; 154.4; 146.1; 146.0; 143.5; 140.2; 138.7; 133.8; 133.5; 132.8; 130.1; 125.2; 124.2; 123.4; 117.9; 55.7; 17.5.

Synthesis and characterization of 4-(1-(6'-methoxy-4-methyl-[2,3'-bipyridin]-5-yl)-1H-1,2,3-triazol-4-yl)benzoic acid (10)

The triazole was synthesized according to **GP1** using 120 mg (0.33 mmol) of the triazole **3**, 105 mg (0.99 mmol) of Na_2CO_3, 101 mg (0.66 mmol) of (6-Methoxypyridin-3-yl)boronic acid and 0.1 eq. of $Pd(PPh_3)_4$. 73 mg (0.19 mmol; 58%) of the crude product were obtained.

HRMS (ESI): $[M+H]^+$ calculated: 388.14042 found: 388.13930

^{1}H-NMR (500 MHz, DMSO-*d6*, *δ* in ppm): 9.19 (s, 1H); 8.98 (s, 1H); 8.78 (s, 1H); 8.45 (m, 1H); 8.19 (s, 1H); 8.06 (s, 4H); 6.99 (m, 1H), 3.94 (s, 3H); 2.38 (s, 3H).

^{13}C-NMR (126 MHz, DMSO-*d6*, *δ* in ppm): 164.9; 155.2; 146.5; 146.4; 146.3; 143.8; 138.1; 134.3; 132.6; 132.0; 130.6; 129.3; 127.4; 125.7; 124.8; 122.4; 111.2; 54.0; 18.9.

Synthesis and characterization of 4-(1-(2'-methoxy-4,5'-dimethyl-[2,3'-bipyridin]-5-yl)-1H-1,2,3-triazol-4-yl)benzoic acid (11)

The triazole was synthesized according to **GP1** using 70 mg (0.19 mmol) of the triazole **3**, 60 mg (0.57 mmol) of Na_2CO_3, 63 mg (0.38 mmol) of (2-Methoxy-5-methylpyridin-3-yl)boronic acid and 0.1 eq. of $Pd(PPh_3)_4$. 76 mg (0.19 mmol; 99%) of the crude product were obtained.

HRMS (ESI): $[M+H]^+$ calculated: 402.15607 found: 402.15422

^{1}H-NMR (500 MHz, DMSO-*d6*, *δ* in ppm): 9.23 (s, 1H); 8.82 (s, 1H); 8.10 (m, 7H); 3.98 (s, 3H); 2.38 (s, 3H); 2.32 (s, 3H).

^{13}C-NMR (126 MHz, DMSO-*d6*, *δ* in ppm): 167.0; 158.9; 154.3; 147.2; 145.9; 145.7; 142.4; 140.3; 134.3; 132.0; 130.3; 130.2; 126.3; 125.4; 124.4; 120.5; 53.5; 17.5; 16.9.

Synthesis and characterization of 4-(1-(4-methyl-6-(quinolin-3-yl)pyridin-3-yl)-1H-1,2,3-triazol-4-yl)benzoic acid (12)

The triazole was synthesized according to **GP1** using 70 mg (0.19 mmol) of the triazole **3**, 60 mg (0.57 mmol) of Na_2CO_3, 66 mg (0.38 mmol) of Quinolin-3-yl-boronic acid and 0.1 eq. of $Pd(PPh_3)_4$. 77 mg (0.19 mmol; 99%) of the crude product were obtained.

HRMS (ESI): $[M+H]^+$ calculated: 408.14550 found: 408.14371

^{1}H-NMR (500 MHz, DMSO-*d6*, *δ* in ppm): 9.31 (s, 1H); 8.96 (s, 1H); 8.75 (m, 1H); 8.28 (t, *J* = 7.7 Hz, 2H); 8.10 (m, 4H); 8.03 (s, 1H); 7.87 (m, 1H); 7.79 (m, 1H); 7.62 (m, 2H); 2.45 (s, 3H).

^{13}C-NMR (126 MHz, DMSO-*d6*, *δ* in ppm): 167.2; 154.8; 149.2; 148.0; 146.3; 143.8; 134.3; 132.2; 131.7; 131.6; 130.3; 129.0; 128.9; 127.5; 125.5; 17.7.

Synthesis and characterization of 4-(1-(4-methyl-6-(quinolin-3-yl)pyridin-3-yl)-1H-1,2,3-triazol-4-yl)benzoic acid (13)

The triazole was synthesized as described previously. [1]

Synthesis and characterization of 4-(1-(6-(2-chlorophenyl)-4-methylpyridin-3-yl)-1H-1,2,3-triazol-4-yl)benzoic acid (14)

The triazole was synthesized according to **GP1** using 150 mg (0.42 mmol) of the triazole **3**, 134 mg (1.26 mmol) of Na_2CO_3, 131 mg (0.84 mmol) of (2-Chlorophenyl)boronic acid and 0.1 eq. of $Pd(PPh_3)_4$. 164 mg (0.42 mmol; 99%) of the crude product were obtained.

HRMS (ESI): $[M+H]^+$ calculated: 391.09563 found: 391.09451

^{1}H-NMR (500 MHz, DMSO-*d6*, *δ* in ppm): 9.26 (s, 1H); 8.85 (s, 1H): 8.08 (s, 4H); 7.87 (s, 1H); 7.65 (m, 2H); 7.52 (m, 2H); 2.40 (s, 3H).

^{13}C-NMR (126 MHz, DMSO-*d6*, *δ* in ppm): 167.3; 157.2; 146.1; 145.7; 142.7; 138.0; 133.9; 132.4; 131.7; 131.2; 130.6; 130.1; 130.0; 127.5; 126.9; 125.3; 124.4; 17.3.

Synthesis and characterization of 4-(1-(6-(3-chlorophenyl)-4-methylpyridin-3-yl)-1H-1,2,3-triazol-4-yl)benzoic acid (15)

The triazole was synthesized according to **GP1** using 300 mg (0.84 mmol) of the triazole **3**, 267 mg (2.52 mmol) of Na_2CO_3, 263 mg (1.68 mmol) of (3-Chlorophenyl)boronic acid and 0.1 eq. of $Pd(PPh_3)_4$. 268 mg (0.69 mmol; 82%) of the crude product were obtained.

HRMS (ESI): $[M+H]^+$ calculated: 391.09563 found: 391.09441

^{1}H-NMR (500 MHz, DMSO-*d6*, *δ* in ppm): 9.22 (s, 1H); 8.82 (s, 1H); 8.28 (s, 1H); 8.23 (s, 1H); 8.15 (m, 1H); 8.07 (s, 4H); 7.58 (m, 2H); 2.41 (s, 3H).

^{13}C-NMR (126 MHz, DMSO-*d6*, *δ* in ppm): 166.9; 154.8; 145.7; 145.5; 143.2; 139.2; 133.6; 132.4; 130.5; 129.8; 129.2; 126.2; 125.2; 125.0; 124.0; 122.7; 17.1.

Synthesis and characterization of 4-(1-(6-(4-chlorophenyl)-4-methylpyridin-3-yl)-1H-1,2,3-triazol-4-yl)benzoic acid (16)

The triazole was synthesized as described previously. [1]

Synthesis and characterization of 4-(1-(6-(2-methoxyphenyl)-4-methylpyridin-3-yl)-1H-1,2,3-triazol-4-yl)benzoic acid (17)

The triazole was synthesized according to **GP1** using 150 mg (0.42 mmol) of the triazole **3**, 134 mg (1.26 mmol) of Na_2CO_3, 128 mg (0.84 mmol) of (2-Methoxyphenyl)boronic acid and 0.1 eq. of $Pd(PPh_3)_4$. 103 mg (0.27 mmol; 65%) of the crude product were obtained.

HRMS (ESI): $[M+H]^+$ calculated: 387.14517 found: 387.14396

^{1}H-NMR (500 MHz, DMSO-*d6*, δ in ppm): 9.18 (s, 1H); 8.78 (s, 1H); 8.04 (s, 4H); 7.98 (s, 1H); 7.76 (m, 1H); 7.45 (m, 1H); 7.20 (m, 1H); 7.10 (m, 1H); 3.88 (s, 3H); 2.35 (s, 3H).

^{13}C-NMR (126 MHz, DMSO-*d6*, δ in ppm): 168.0; 157.2; 156.6; 146.4; 145.9; 142.3; 133.8; 132.1; 131.1; 130.4; 127.6; 127.2; 125.5; 124.6; 121.1; 112.4; 56.1; 17.8.

Synthesis and characterization of 4-(1-(6-(3-methoxyphenyl)-4-methylpyridin-3-yl)-1H-1,2,3-triazol-4-yl)benzoic acid (18)

The triazole was synthesized according to **GP1** using 150 mg (0.42 mmol) of the triazole **3**, 134 mg (1.26 mmol) of Na_2CO_3, 128 mg (0.84 mmol) of (3-Methoxyphenyl)boronic acid and 0.1 eq. of $Pd(PPh_3)_4$. 162 mg (0.42 mmol; 99%) of the crude product were obtained.

HRMS (ESI): $[M+H]^+$ calculated: 387.14517 found: 387.14396

^{1}H-NMR (500 MHz, DMSO-*d6*, δ in ppm): 9.19 (s, 1H); 8.80 (s, 1H); 8.21 (s, 1H); 8.06 (s, 4H); 7.76 (m, 1H); 7.73 (m, 1H); 7.46 (m, 1H); 7.08 (m, 1H); 3.86 (s, 3H); 2.39 (s, 3H).

^{13}C-NMR (126 MHz, DMSO-*d6*, δ in ppm): 167.4; 159.8; 156.6; 146.1; 145.7; 143.3; 139.0; 133.7; 132.4; 130.1; 130.0; 125.2; 124.3; 122.7; 119.2; 115.6; 112.0; 55.3; 17.4.

Synthesis and characterization of 4-(1-(6-(4-methoxyphenyl)-4-methylpyridin-3-yl)-1H-1,2,3-triazol-4-yl)benzoic acid (19)

The triazole was synthesized according to **GP1** using 150 mg (0.42 mmol) of the triazole **3**, 134 mg (1.26 mmol) of Na_2CO_3, 128 mg (0.84 mmol) of (4-Methoxyphenyl)boronic acid and 0.1 eq. of $Pd(PPh_3)_4$, 145 mg (0.37 mmol; 91%) of the crude product were obtained.

HRMS (ESI): $[M+H]^+$ calculated: 387.14517 found: 387.14392

^{1}H-NMR (500 MHz, DMSO-*d6*, δ in ppm): 9.16 (s, 1H); 8.74 (s, 1H); 8.16 (m, 2H); 8.12 (s, 1H); 8.04 (s, 4H); 7.10 (m, 2 H); 3.85 (s, 3H); 2.37 (s, 3H).

^{13}**C-NMR** (126 MHz, DMSO-*d6*, *δ* in ppm): 160.9; 156.8; 146.3; 145.8; 143.3; 133.5; 131.8; 130.2; 130.1; 128.5; 125.3; 124.3; 121.7; 114.5; 55.5; 17.6.

Synthesis and characterization of 4-(1-(6-(2-chloro-4-methoxyphenyl)-4-methylpyridin-3-yl)-1H-1,2,3-triazol-4-yl)benzoic acid (20)

The triazole was synthesized according to **GP1** using 169 mg (0.47 mmol) of the triazole **3**, 149 mg (1.41 mmol) of Na_2CO_3, 175 mg (0.94 mmol) of (2-Chloro-4-methoxyphenyl)boronic acid and 0.1 eq. of $Pd(PPh_3)_4$, 197 mg (0.47 mmol; 99%) of the crude product were obtained.

HRMS (ESI): $[M+H]^+$ calculated: 421.10619 found: 421.10554

^{1}**H-NMR** (500 MHz, DMSO-*d6*, *δ* in ppm): 9.25 (s, 1H); 8.81 (s, 1H); 8.08 (s, 4H); 7.83 (s, 1H); 7.61 (m, 1H); 7.20 (s, 1H); 7.09 (m, 1 H); 3.86 (s, 3H); 2.37 (s, 3H).

^{13}**C-NMR** (126 MHz, DMSO-*d6*, *δ* in ppm): 167.0; 160.3; 156.9; 145.9; 145.6; 142.5; 134.3; 132.6; 132.0; 130.3; 130.2; 130.1; 126.7; 125.3; 124.5; 115.1; 113.7; 55.8; 17.3.

Synthesis and characterization of 4-(1-(6-(2-hydroxyphenyl)-4-methylpyridin-3-yl)-1H-1,2,3-triazol-4-yl)benzoic acid (21)

The triazole was synthesized as described previously. [1]

Synthesis and characterization of 4-(1-(6-(3-hydroxyphenyl)-4-methylpyridin-3-yl)-1H-1,2,3-triazol-4-yl)benzoic acid (22)

The triazole was synthesized according to **GP1** using 120 mg (0.33 mmol) of the triazole **3**, 105 mg (0.99 mmol) of Na_2CO_3, 91 mg (0.66 mmol) of (3-Hydroxyphenyl)boronic acid and 0.1 eq. of $Pd(PPh_3)_4$. 120 mg (0.32 mmol; 98%) of the crude product were obtained.

HRMS (ESI): $[M+H]^+$ calculated: 373.12952 found: 373.12886

^{1}**H-NMR** (500 MHz, DMSO-*d6*, *δ* in ppm): 9.19 (s, 1H); 8.78 (s, 1H); 8.10 (s, 1H); 8.05 (s, 4H); 7.61 (m, 2H); 7.33 (m, 2H); 6.91 (m, 1H); 2.38 (s, 3H).

^{13}**C-NMR** (126 MHz, DMSO-*d6*, *δ* in ppm): 167.5; 157.9; 156.9; 146.1; 145.7; 143.2; 138.9; 133.4; 132.2; 130.0; 129.9; 125.1; 124.1; 122.5; 117.6; 116.8; 113.7; 17.4.

Synthesis and characterization of 4-(1-(6-(4-hydroxyphenyl)-4-methylpyridin-3-yl)-1H-1,2,3-triazol-4-yl)benzoic acid (23)

The triazole was synthesized according to **GP1** using 120 mg (0.33 mmol) of the triazole **3**, 105 mg (0.99 mmol) of Na_2CO_3, 91 mg (0.66 mmol) of (4-Hydroxyphenyl)boronic acid and 0.1 eq. of $Pd(PPh_3)_4$. 122 mg (0.33 mmol; 99%) of the crude product were obtained.

HRMS (ESI): $[M+H]^+$ calculated: 373.12952 found: 373.12891

^{1}H-NMR (500 MHz, DMSO-*d6*, *δ* in ppm): 9.19 (s, 1H); 8.70 (s, 1H); 8.08 (s, 4H); 8.04 (m, 3H); 6.91 (m, 2H); 2.35 (s, 3H).

^{13}C-NMR (126 MHz, DMSO-*d6*, *δ* in ppm): 167.1; 159.2; 157.1; 145.9; 145.5; 143.0; 134.3; 131.3; 130.1; 128.4; 125.3; 124.4; 121.1; 115.7; 17.4.

Synthesis and characterization of 4-(1-(4-methyl-6-(3-(methylcarbamoyl)phenyl)pyridin-3-yl)-1H-1,2,3-triazol-4-yl)benzoic acid (24)

The triazole was synthesized according to **GP1** using 70 mg (0.19 mmol) of the triazole **3**, 60 mg (0.57 mmol) of Na_2CO_3, 68 mg (0.38 mmol) of (3-(Methylcarbamoyl)phenyl)boronic acid and 0.1 eq. of $Pd(PPh_3)_4$. 78 mg (0.19 mmol; 99%) of the crude product were obtained.

HRMS (ESI): $[M+H]^+$ calculated: 414.15607 found: 414.15512

^{1}H-NMR (500 MHz, DMSO-*d6*, *δ* in ppm): 9.20 (s, 1H); 8.80 (s, 1H); 8.59 (m, 2H); 8.28 (m, 1H); 8.21 (s, 1H); 8.05 (m, 4H); 7.92 (m, 1H); 7.60 (t, *J* = 7.8 Hz, 1H); 2.81 (d, *J* = 4.4 Hz, 3H); 2.38 (s, 3H).

^{13}C-NMR (126 MHz, DMSO-*d6*, *δ* in ppm): 167.1; 166.4; 156.3; 146.0; 145.8; 143.4; 137.6; 135.3; 134.2; 132.5; 131.5; 130.7; 130.1; 129.4; 129.0; 128.8; 128.3; 125.6; 125.3; 124.4; 122.8; 26.3; 17.5.

Synthesis and characterization of 4-(1-(4-methyl-6-(4-(methylcarbamoyl)phenyl)pyridin-3-yl)-1H-1,2,3-triazol-4-yl)benzoic acid (25)

The triazole was synthesized according to **GP1** using 70 mg (0.19 mmol) of the triazole **3**, 60 mg (0.57 mmol) of Na_2CO_3, 68 mg (0.38 mmol) of (4-(Methylcarbamoyl)phenyl)boronic acid and 0.1 eq. of $Pd(PPh_3)_4$. 78 mg (0.19 mmol; 99%) of the crude product were obtained.

HRMS (ESI): $[M+H]^+$ calculated: 414.15607 found: 414.15433

^{1}H-NMR (500 MHz, DMSO-*d6*, *δ* in ppm): 9.25 (s, 1H); 8.85 (d, *J* = 10.5 Hz, 1H); 8.35 (m, 1H); 8.27 (d, *J* = 8.7 Hz, 1H); 8.09 (m, 5H); 8.04 (m, 1H); 7.88 (m, 1H); 2.83 (m, 3H); 2.42 (s, 3H).

^{13}C-NMR (126 MHz, DMSO-*d6*, *δ* in ppm): 167.3; 166.6; 156.2; 146.3; 146.2; 144.0; 140.0; 134.6; 130.5; 128.0; 127.5; 127.1; 125.7; 124.7; 123.7; 123.4; 26.6; 17.7.

Synthesis and characterization of 3-(5-(4-(4-Carboxyphenyl)-1H-1,2,3-triazol-1-yl)-4-methylpyridin-2-yl)benzoic acid (26)

The triazole was synthesized according to **GP1** using 120 mg (0.33 mmol) of the triazole **3**, 105 mg (0.99 mmol) of Na_2CO_3, 110 mg (0.66 mmol) of 3-Boronobenzoic acid and 0.1 eq. of $Pd(PPh_3)_4$. 124 mg (0.31 mmol; 99%) of the crude product were obtained.

HRMS (ESI): $[M+H]^+$ calculated: 401.12443 found: 401.12262

^{1}H-NMR (500 MHz, DMSO-*d6*, *δ* in ppm): 9.23 (s, 1H); 8.84 (s, 1H); 8.76 (s, 1H); 8.42 (br.d., *J* = 7.93 Hz, 1H); 8.27 (s, 1H); 8.09 (m, 5H); 7.68 (t, *J* = 7.71 Hz, 1H); 2.42 (s, 3H).

^{13}C-NMR (126 MHz, DMSO-*d6*, *δ* in ppm): 167.1; 167.0; 163.1; 155.7; 145.8; 143.5; 141.9; 137.7; 134.1; 131.0; 130.0; 127.5; 125.2; 124.3; 123.7; 17.3.

Synthesis and characterization of 4-(5-(4-(4-Carboxyphenyl)-1H-1,2,3-triazol-1-yl)-4-methylpyridin-2-yl)benzoic acid (27)

The triazole was synthesized according to **GP1** using 120 mg (0.33 mmol) of the triazole **3**, 105 mg (0.99 mmol) of Na_2CO_3, 110 mg (0.66 mmol) of 4-Boronobenzoic acid and 0.1 eq. of $Pd(PPh_3)_4$. 124 mg (0.31 mmol; 99%) of the crude product were obtained.

HRMS (ESI): $[M+H]^+$ calculated: 401.12443 found: 401.12372

^{1}H-NMR (500 MHz, DMSO-*d6*, *δ* in ppm): 9.24 (s, 1H); 8.85 (s, 1H); 8.29 (m, 3H); 8.06 (m, 6H); 2.42 (s, 3H).

^{13}C-NMR (126 MHz, DMSO-*d6*, *δ* in ppm): 167.0; 155.7; 146.5; 146.2; 146.0; 143.5; 142.0; 134.0; 133.2; 132.7; 130.0; 127.0; 125.4; 124.4; 123.3; 17.0.

Synthesis and characterization of 4-(1-(4-methyl-6-(3-phenoxyphenyl)pyridin-3-yl)-1H-1,2,3-triazol-4-yl)benzoic acid (28)

The triazole was synthesized according to **GP1** using 70 mg (0.19 mmol) of the triazole **3**, 60 mg (0.57 mmol) of Na_2CO_3, 81 mg (0.38 mmol) of (3-Phenoxyphenyl)boronic acid and 0.1 eq. of $Pd(PPh_3)_4$. 69 mg (0.15 mmol; 81%) of the crude product were obtained.

HRMS (ESI): $[M+H]^+$ calculated: 449.16082 found: 449.15882

^{1}H-NMR (500 MHz, DMSO-*d6*, *δ* in ppm): 9.21 (s, 1H); 8.78 (s, 1H); 8.22 (s, 1H); 8.08 (s, 4H); 7.96 (d, *J* = 8.1 Hz, 1H); 7.84 (t, *J* = 2.0 Hz, 1H); 7.57 (t, *J* = 8.1 Hz, 1H); 7.44 (m, 2H); 7.16 (m, 2H); 7.11 (m, 2H); 2.39 (s, 3H).

^{13}C-NMR (126 MHz, DMSO-*d6*, *δ* in ppm): 167.5; 157.9; 157.0; 146.4; 146.2; 144.0; 140.0; 134.7; 132.9; 131.2; 130.7; 130.6; 125.9; 124.9; 124.3; 119.3; 117.2; 17.9.

Synthesis and characterization of 4-(1-(6-(3-(benzyloxy)phenyl)-4-methylpyridin-3-yl)-1H-1,2,3-triazol-4-yl)benzoic acid (29)

The triazole was synthesized according to **GP1** using 70 mg (0.19 mmol) of the triazole **3**, 60 mg (0.57 mmol) of Na_2CO_3, 87 mg (0.38 mmol) of (3-(Benzyloxy)phenyl)boronic acid and 0.1 eq. of $Pd(PPh_3)_4$. 49 mg (0.11 mmol; 56%) of the crude product were obtained.

HRMS (ESI): $[M+H]^+$ calculated: 463.17647 found: 463.17451

^{1}H-NMR (500 MHz, DMSO-*d6*, *δ* in ppm): 9.22 (s, 1H); 8.80 (s, 1H); 8.22 (s, 1H); 8.08 (s, 4H); 7.83 (m, 1H); 7.78 (d, *J* = 7.8 Hz, 1H); 7.45 (m, 5H); 7.36 (d, *J* = 7.3 Hz ,1H); 7.16 (dd, *J* = 8.2, 2.1 Hz, 1H); 5.22 (s, 2H); 2.39 (s, 3H).

^{13}C-NMR (126 MHz, DMSO-*d6*, *δ* in ppm): 167.3; 159.2; 156.8; 146.2; 146.0; 143.6; 139.2; 137.3; 132.6; 130.4; 128.8; 128.2; 128.0; 125.7; 124.7; 123.0; 119.8; 116.6; 113.4; 69.7; 17.7.

Synthesis and characterization of 4-(1-(6-(3-((2-chlorobenzyl)oxy)phenyl)-4-methylpyridin-3-yl)-1H-1,2,3-triazol-4-yl) benzoic acid (30)

The triazole was synthesized according to **GP1** using 70 mg (0.19 mmol) of the triazole **3**, 60 mg (0.57 mmol) of Na_2CO_3, 100 mg (0.38 mmol) of (3-((2-Chlorobenzyl)oxy)phenyl)boronic acid and 0.1 eq. of $Pd(PPh_3)_4$. 34 mg (0.07 mmol; 36%) of the crude product were obtained.

HRMS (ESI): $[M+H]^+$ calculated: 497.13749 found: 497.13545

^{1}H-NMR (500 MHz, DMSO-*d6*, *δ* in ppm): 9.24 (s, 1H); 8.81 (s, 1H); 8.24 (s, 1H); 8.09 (m, 4H); 7.86 (m, 1H); 7.82 (m, 1H); 7.68 (m, 1H); 7.55 (m, 1H); 7.50 (t, *J* = 7.9 Hz,1H); 7.43 (m, 2H); 7.19 (dd, *J* = 8.2, 2.1 Hz, 1H); 5.29 (s, 2H); 2.41 (s, 3H).

^{13}C-NMR (126 MHz, DMSO-*d6*, *δ* in ppm): 167.2; 158.9; 156.6; 146.1; 145.8; 139.2; 134.4; 132.9; 130.5; 130.4; 130.3; 130.2; 129.6; 127.6; 125.5; 124.5; 122.9; 119.9; 116.3; 113.2; 67.2; 17.5.

Synthesis and characterization of 4-(1-(6-(3-((2-methoxybenzyl)oxy)phenyl)-4-methylpyridin-3-yl)-1H-1,2,3-triazol-4-yl) benzoic acid (31)

The triazole was synthesized according to **GP1** using 70 mg (0.19 mmol) of the triazole **3**, 60 mg (0.57 mmol) of Na_2CO_3, 98 mg (0.38 mmol) of (3-((2-Methoxybenzyl)oxy)phenyl)boronic acid and 0.1 eq. of $Pd(PPh_3)_4$. 58 mg (0.12 mmol; 63%) of the crude product were obtained.

HRMS (ESI): $[M+H]^+$ calculated: 493.18703 found: 493.18522

^{1}H-NMR (500 MHz, DMSO-*d6*, *δ* in ppm): 9.22 (s, 1H); 8.80 (s, 1H); 8.22 (s, 1H); 8.08 (m, 4H); 7.82 (m, 1H); 7.77 (m, 1H); 7.46 (m, 2H); 7.35 (m, 1H); 7.14 (m, 1H); 7.07 (d, *J* = 8.1 Hz, 1H); 6.99 (m, 1H); 5.17 (s, 2H); 3.85 (s, 3H); 2.39 (s, 3H).

^{13}C-NMR (126 MHz, DMSO-*d6*, *δ* in ppm): 167.2; 159.2; 157.1; 156.7; 146.1; 145.8; 143.5; 134.4; 132.5; 130.3; 129.7; 129.4; 125.5; 124.7; 124.5; 120.5; 119.5; 112.9; 111.1; 64.8; 55.6; 17.5.

Synthesis and characterization of 4-(1-(6-(3-((3-methoxybenzyl)oxy)phenyl)-4-methylpyridin-3-yl)-1H-1,2,3-triazol-4-yl) benzoic acid (32)

The triazole was synthesized according to **GP1** using 70 mg (0.19 mmol) of the triazole **3**, 60 mg (0.57 mmol) of Na_2CO_3, 98 mg (0.38 mmol) of (3-((3-Methoxybenzyl)oxy)phenyl)boronic acid and 0.1 eq. of $Pd(PPh_3)_4$. 32 mg (0.06 mmol; 34%) of the crude product were obtained.

HRMS (ESI): $[M+H]^+$ calculated: 493.18703 found: 493.18504

^{1}H-NMR (500 MHz, DMSO-*d6*, *δ* in ppm): 9.14 (s, 1H); 8.79 (s, 1H); 8.21 (s, 1H); 7.99 (m, 4H); 7.83 (m, 1H); 7.77 (m, 1H); 7.46 (t, *J* = 7.9 Hz, 1H); 7.32 (m, 1H); 7.15 (dd, *J* = 8.2, 2.1 Hz, 1H); 7.08 (m, 2H); 6.91 (m, 1H); 5.20 (s, 2H); 3.77 (s, 3H); 2.39 (s, 3H).

^{13}C-NMR (126 MHz, DMSO-*d6*, *δ* in ppm): 159.0; 158.5; 156.1; 146.2; 145.3; 143.0; 138.6; 138.3; 131.6; 129.8; 129.3; 124.4; 123.4; 122.4; 119.4; 119.1; 113.0; 112.8; 112.7; 68.9; 54.7; 17.1.

Synthesis and characterization of 4-(1-(6-(3-((3,5-dimethoxybenzyl)oxy)phenyl)-4-methylpyridin-3-yl)-1H-1,2,3-triazol-4-yl) benzoic acid (33)

The triazole was synthesized according to **GP1** using 70 mg (0.19 mmol) of the triazole **3**, 60 mg (0.57 mmol) of Na_2CO_3, 109 mg (0.38 mmol) of (3-((3,5-Dimethoxybenzyl)oxy)phenyl)boronic acid and 0.1 eq. of $Pd(PPh_3)_4$. 51 mg (0.10 mmol; 53%) of the crude product were obtained.

HRMS (ESI): $[M+H]^+$ calculated: 523.19760 found: 523.19575

^{1}H-NMR (500 MHz, DMSO-*d6*, *δ* in ppm): 9.20 (s, 1H); 8.79 (s, 1H); 8.21 (s, 1H); 8.06 (s, 4H); 7.82 (m, 1H); 7.77 (d, *J* = 7.8 Hz, 1H); 7.46 (t, *J* = 7.9 Hz, 1H); 7.14 (dd, *J* = 8.2, 2.0 Hz, 1H); 6.66 (d, *J* = 2.3 Hz, 2H); 6.45 (t, *J* = 2.3 Hz, 1H); 5.15 (s, 2H); 3.75 (s, 6H); 2.39 (s, 3H).

^{13}C-NMR (126 MHz, DMSO-*d6*, *δ* in ppm): 167.4; 160.8; 159.0; 156.8; 146.2; 145.9; 143.6; 139.6; 139.1; 132.6; 130.3; 125.5; 124.5; 123.0; 119.7; 116.5; 113.3; 105.7; 99.6; 69.4; 55.4; 17.6.

Synthesis and characterization of 4-(1-(6-(3-((4-methoxybenzyl)oxy)phenyl)-4-methylpyridin-3-yl)-1H-1,2,3-triazol-4-yl) benzoic acid (34)

The triazole was synthesized according to **GP1** using 70 mg (0.19 mmol) of the triazole **3**, 60 mg (0.57 mmol) of Na_2CO_3, 98 mg (0.38 mmol) of (3-((4-Methoxybenzyl)oxy)phenyl)boronic acid and 0.1 eq. of $Pd(PPh_3)_4$. 68 mg (0.14 mmol; 73%) of the crude product were obtained.

HRMS (ESI): $[M+H]^+$ calculated: 493.18703 found: 493.18514

^{1}H-NMR (500 MHz, DMSO-*d6*, *δ* in ppm): 9.22 (s, 1H); 8.81 (s, 1H); 8.23 (s, 1H); 8.08 (s, 4H); 7.82 (m, 1H); 7.77 (m, 1H); 7.45 (m, 3H); 7.15 (dd, *J* = 8.2, 2.2 Hz, 1H); 6.97 (d, *J* = 8.5 Hz, 2H); 5.14 (s, 2H); 3.77 (s, 3H); 2.40 (s, 3H).

^{13}C-NMR (126 MHz, DMSO-*d6*, *δ* in ppm): 167.3; 159.2; 159.1; 156.8; 146.1; 145.8; 143.5; 139.0; 132.5; 130.3; 129.7; 129.0; 125.5; 124.5; 122.9; 114.0; 113.2; 69.3; 55.3; 17.5.

Synthesis and characterization of 4-(1-(6-(2-((4-methoxybenzyl)oxy)phenyl)-4-methylpyridin-3-yl)-1H-1,2,3-triazol-4-yl)benzoic acid (35)

The triazole was synthesized according to **GP1** using 120 mg (0.33 mmol) of the triazole **3**, 105 mg (0.99 mmol) of Na_2CO_3, 170 mg (0.66 mmol) of (2-((4-Methoxybenzyl)oxy)phenyl)boronic acid and 0.1 eq. of $Pd(PPh_3)_4$. 160 mg (0.32 mmol; 98%) of the crude product were obtained.

HRMS (ESI): $[M+H]^+$ calculated: 493.18703 found: 493.18691

^{1}H-NMR (500 MHz, DMSO-*d6*, *δ* in ppm): 9.25 (s, 1H); 8.84 (s, 1H); 8.44 (s, 1H); 8.09 (m, 4H); 7.22 (m, 2H); 7.12 (m, 1H); 6.94 (m, 1H); 6.81 (m, 4H); 3.92 (s, 2H); 3.71 (m, 3H); 2.44 (s, 3H).

^{13}C-NMR (126 MHz, DMSO-*d6*, *δ* in ppm): 167.0; 157.8; 157.5; 157.4; 157.2; 152.3; 146.0; 145.0; 142.6; 132.7; 131.6; 130.5; 130.2; 129.7; 129.6; 128.1; 125.6; 124.5; 122.3; 119.6; 118.7; 117.6; 113.7; 113.6; 55.0; 34.6; 34.3; 17.8.

Synthesis and characterization of 4-(1-(6-(4-((4-methoxybenzyl)oxy)phenyl)-4-methylpyridin-3-yl)-1H-1,2,3-triazol-4-yl)benzoic acid (36)

The triazole was synthesized according to **GP1** using 300 mg (0.84 mmol) of the triazole **3**, 267 mg (2.52 mmol) of Na_2CO_3, 434 mg (1.68 mmol) of (4-((4-Methoxybenzyl)oxy)phenyl)boronic acid and 0.1 eq. of $Pd(PPh_3)_4$. 413 mg (0.84 mmol; 99%) of the crude product were obtained.

HRMS (ESI): $[M+H]^+$ calculated: 493.18703 found: 493.18695

^{1}H-NMR (500 MHz, DMSO-*d6*, *δ* in ppm): 9.18 (s, 1H); 8.74 (s, 1H); 8.13 (m, 2H); 8.06 (s, 4H); 7.42 (m, 2H); 7.16 (m, 2H); 6.97 (m, 3H); 5.12 (s, 2H); 3.77 (s, 3H); 2.37 (s, 3H).

^{13}C-NMR (126 MHz, DMSO-*d6*, *δ* in ppm): 167.5; 160.0; 159.2; 156.8; 146.2; 145.8; 143.3; 133.9; 131.8; 130.2; 129.8; 128.9; 128.5; 127.4; 127.1; 125.4; 124.4; 121.7; 115.8; 115.3; 114.9; 114.0; 55.3; 30.9; 17.6.

1.2 Protein Expression

The protein expression and purification of the His-tagged oligomerization-deficient mutant of the KSHV LANA *C*-terminal DNA binding domain (DBD; aa1008-1146) was carried out according to a previous publication. [2]

1.3 Microscale Thermophoresis (MST) Assay

Microscale Thermophoresis Assay was done as mentioned in a previous publication. [2]

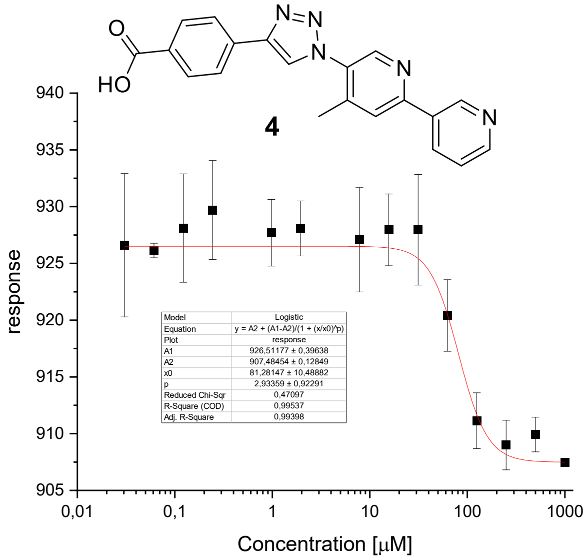

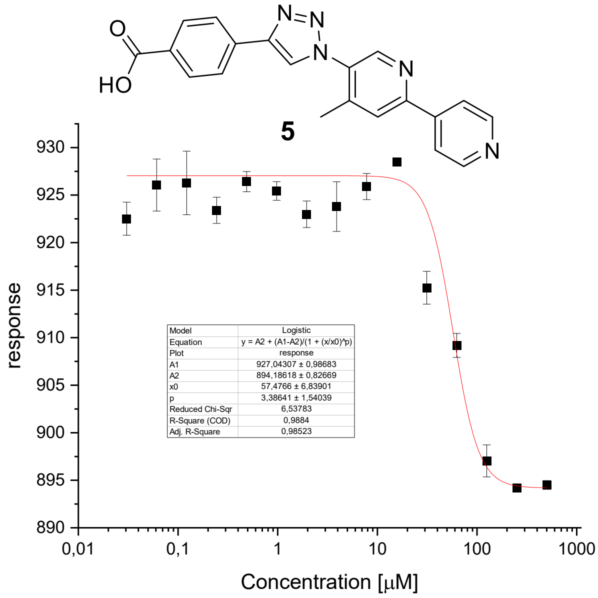

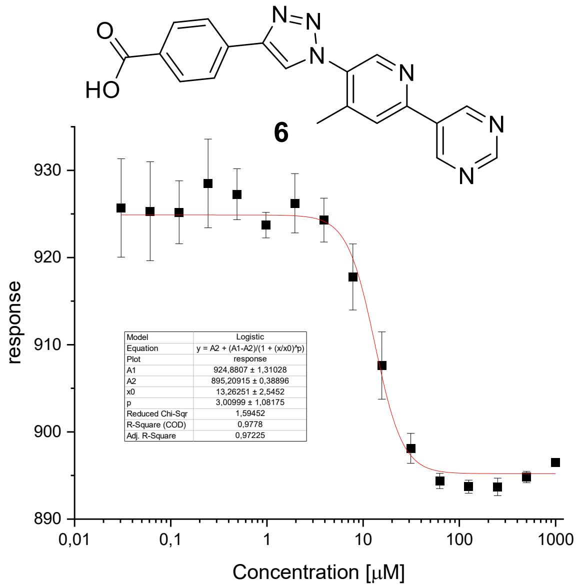

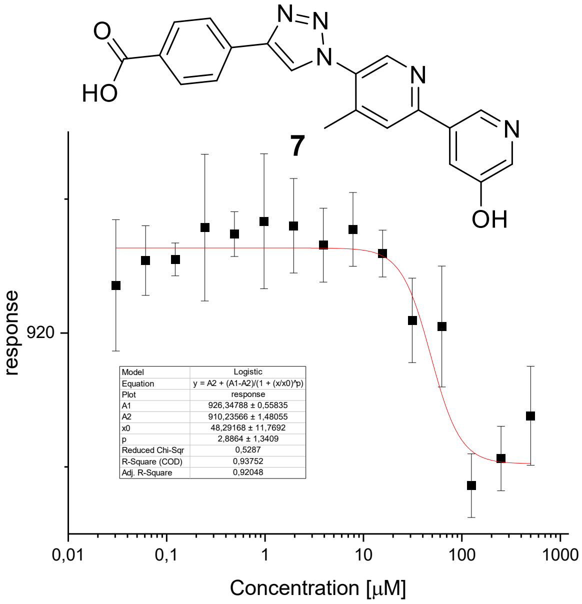

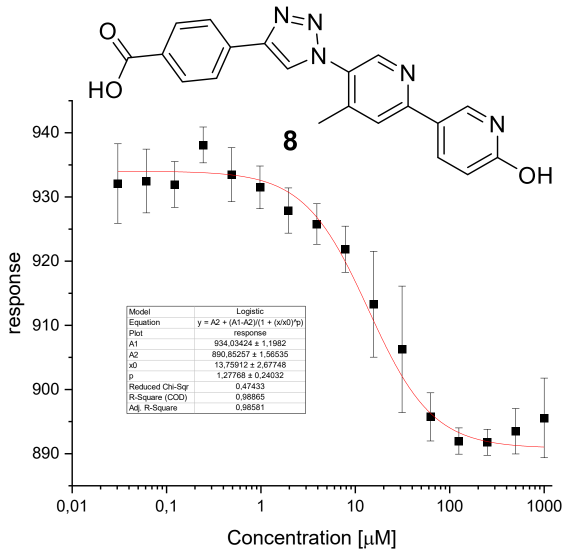

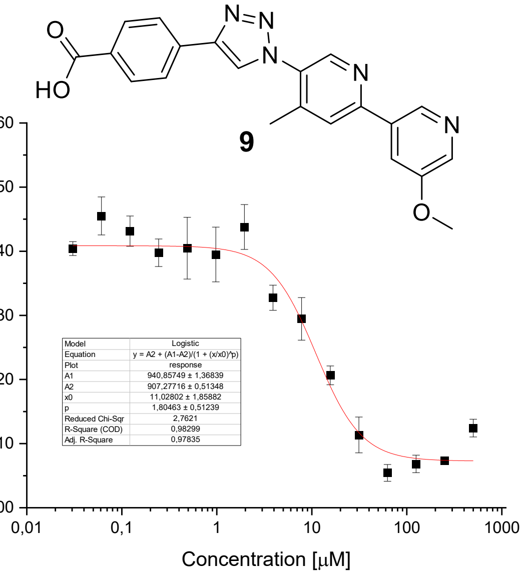

response

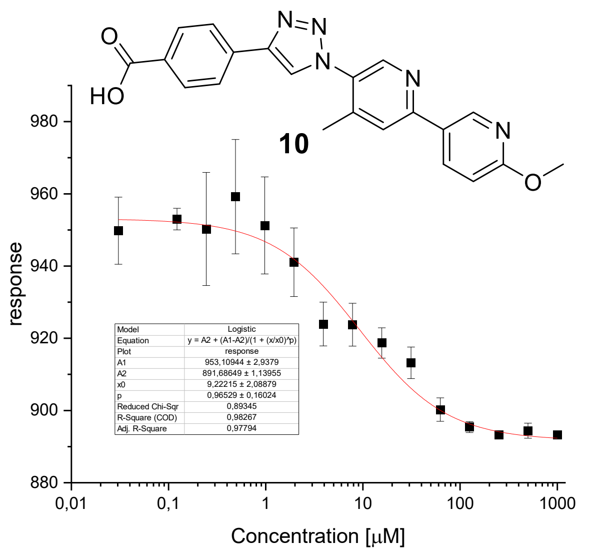
10
response
980
960
940
920
900
880
0,01
0,1
1
10
100
1000
Concentration [μM]
Model | Logistic
Equation | y = A2 + (A1-A2)/(1 + (x/x0)^p)
Plot | response
A1 | 953,10944 ± 2,9379
A2 | 891,68649 ± 1,13955
x0 | 9,22215 ± 2,08879
p | 0,96529 ± 0,16024
Reduced Chi-Sqr | 0,89345
R-Square (COD) | 0,98267
Adj. R-Square | 0,97794

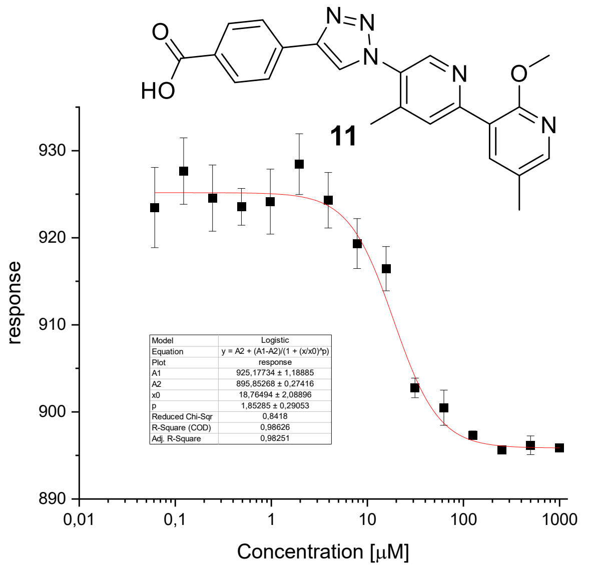
11
response
930
920
910
900
890
0,01
0,1
1
10
100
1000
Concentration [μM]
Model | Logistic
Equation | y = A2 + (A1-A2)/(1 + (x/x0)^p)
Plot | response
A1 | 925,17734 ± 1,18885
A2 | 895,85268 ± 0,27416
x0 | 18,76494 ± 2,08896
p | 1,85285 ± 0,29053
Reduced Chi-Sqr | 0,8418
R-Square (COD) | 0,98626
Adj. R-Square | 0,98251

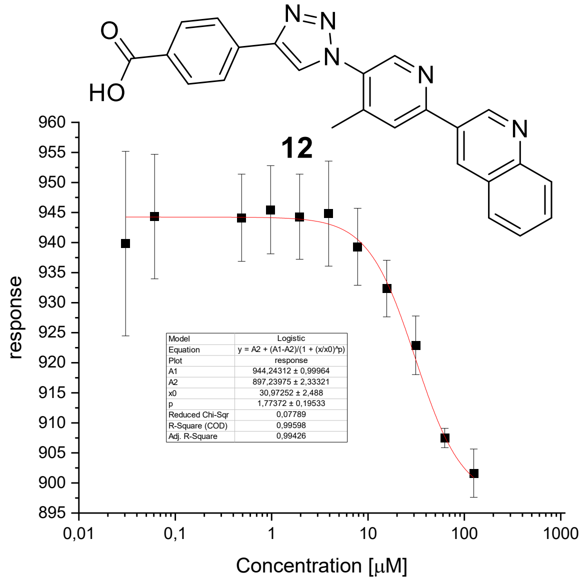
12
response
960
955
950
945
940
935
930
925
920
915
910
905
900
895
0,01
0,1
1
10
100
1000
Concentration [μM]
Model | Logistic
Equation | y = A2 + (A1-A2)/(1 + (x/x0)^p)
Plot | response
A1 | 944,24312 ± 0,99964
A2 | 897,23975 ± 2,33321
x0 | 30,97252 ± 2,488
p | 1,77372 ± 0,19533
Reduced Chi-Sqr | 0,07789
R-Square (COD) | 0,99598
Adj. R-Square | 0,99426

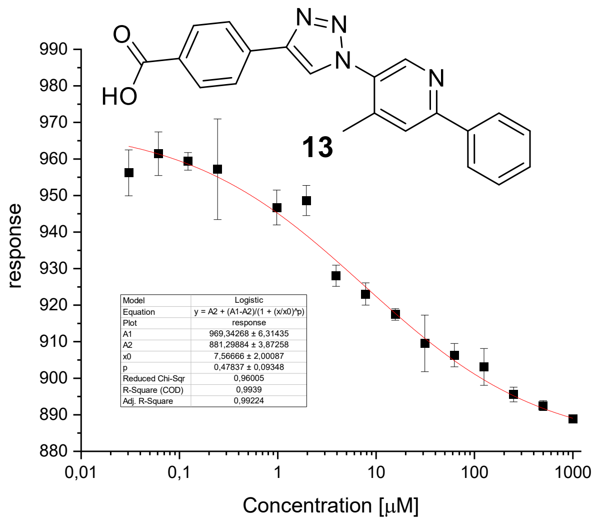
13
response
990
980
970
960
950
940
930
920
910
900
890
880
0,01
0,1
1
10
100
1000
Concentration [μM]
Model | Logistic
Equation | y = A2 + (A1-A2)/(1 + (x/x0)^p)
Plot | response
A1 | 969,34268 ± 6,31435
A2 | 881,29884 ± 3,87258
x0 | 7,56666 ± 2,00087
p | 0,47837 ± 0,09348
Reduced Chi-Sqr | 0,96005
R-Square (COD) | 0,9939
Adj. R-Square | 0,99224

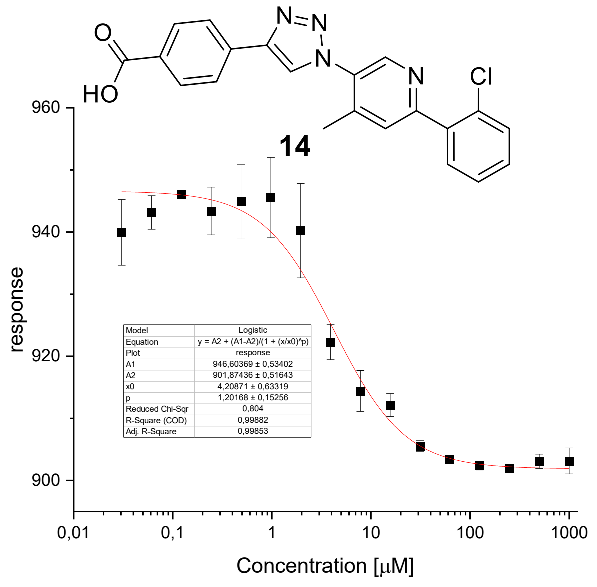
14
response
960
940
920
900
0,01
0,1
1
10
100
1000
Concentration [μM]
Model | Logistic
Equation | y = A2 + (A1-A2)/(1 + (x/x0)^p)
Plot | response
A1 | 946,60369 ± 0,53402
A2 | 901,87436 ± 0,51643
x0 | 4,20871 ± 0,63319
p | 1,20168 ± 0,15256
Reduced Chi-Sqr | 0,804
R-Square (COD) | 0,99882
Adj. R-Square | 0,99853

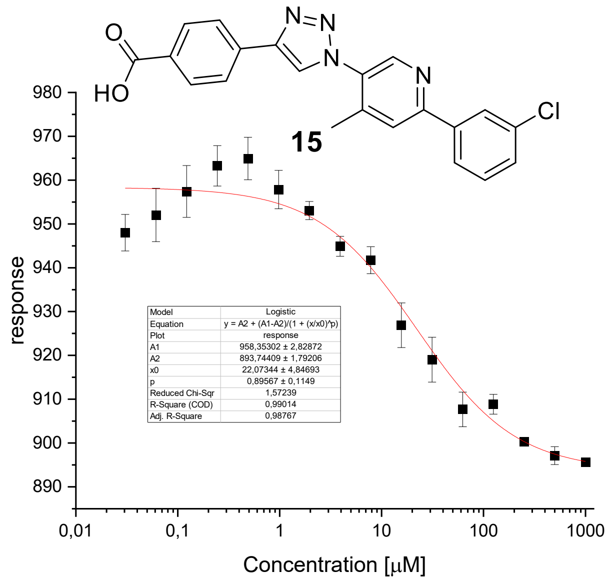
15
response
980
970
960
950
940
930
920
910
900
890
0,01
0,1
1
10
100
1000
Concentration [μM]
Model | Logistic
Equation | y = A2 + (A1-A2)/(1 + (x/x0)^p)
Plot | response
A1 | 958,35302 ± 2,82872
A2 | 893,74409 ± 1,79206
x0 | 22,07344 ± 4,84693
p | 0,89567 ± 0,1149
Reduced Chi-Sqr | 1,57239
R-Square (COD) | 0,99014
Adj. R-Square | 0,98767

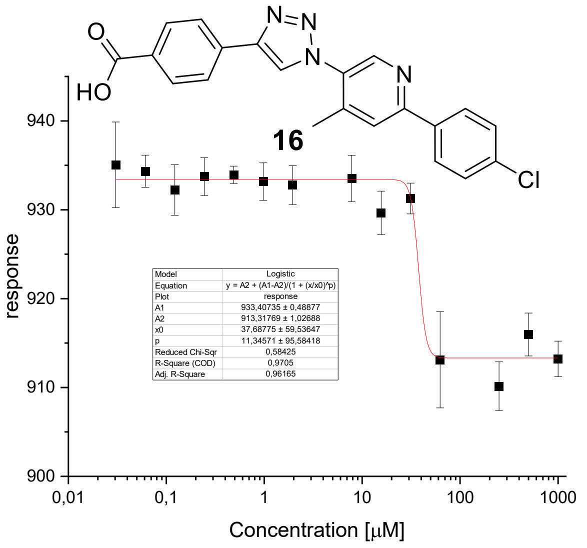
O
HO
N
N=N
N
16
Cl
940
930
920
910
900
response
0,01
0,1
1
10
100
1000
Concentration [µM]
Model | Logistic
Equation | y = A2 + (A1-A2)/(1 + (x/x0)^p)
Plot | response
A1 | 933,40735 ± 0,48877
A2 | 913,31769 ± 1,02688
x0 | 37,68775 ± 59,53647
p | 11,34571 ± 95,58418
Reduced Chi-Sqr | 0,58425
R-Square (COD) | 0,9705
Adj. R-Square | 0,96165

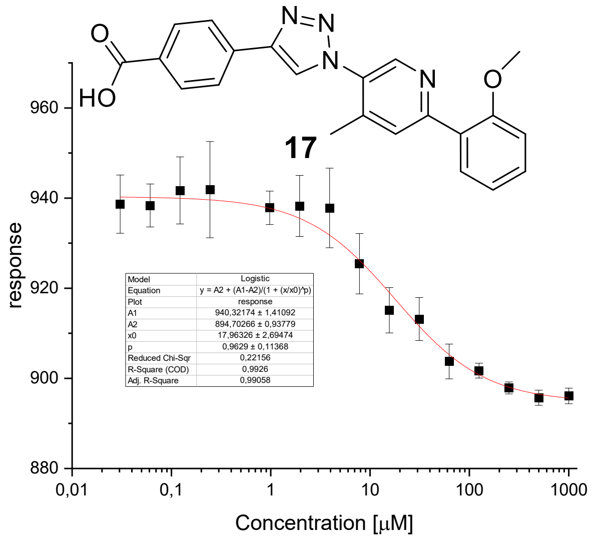
O
HO
N
N=N
N
O
17
960
940
920
900
880
response
0,01
0,1
1
10
100
1000
Concentration [µM]
Model | Logistic
Equation | y = A2 + (A1-A2)/(1 + (x/x0)^p)
Plot | response
A1 | 940,32174 ± 1,41092
A2 | 894,70266 ± 0,93779
x0 | 17,96326 ± 2,69474
p | 0,9629 ± 0,11368
Reduced Chi-Sqr | 0,22156
R-Square (COD) | 0,9926
Adj. R-Square | 0,99058

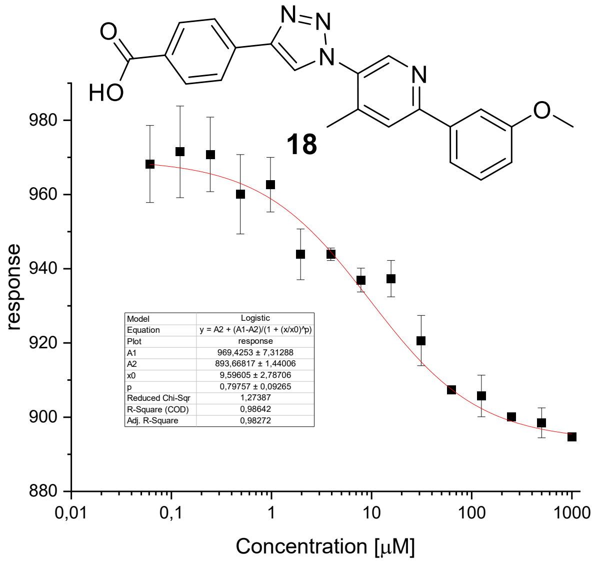
O
HO
N
N=N
N
O
18
980
960
940
920
900
880
response
0,01
0,1
1
10
100
1000
Concentration [µM]
Model | Logistic
Equation | y = A2 + (A1-A2)/(1 + (x/x0)^p)
Plot | response
A1 | 969,4253 ± 7,31288
A2 | 893,66817 ± 1,44006
x0 | 9,59605 ± 2,78706
p | 0,79757 ± 0,09265
Reduced Chi-Sqr | 1,27387
R-Square (COD) | 0,98642
Adj. R-Square | 0,98272

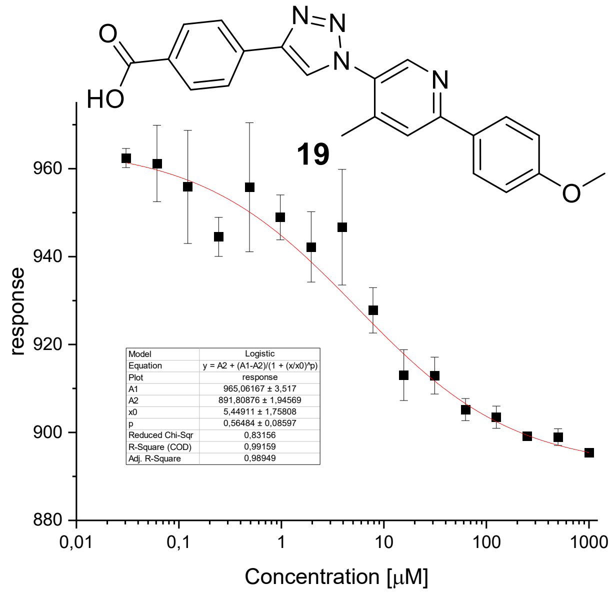
O
HO
N
N=N
N
19
O
960
940
920
900
880
response
0,01
0,1
1
10
100
1000
Concentration [µM]
Model | Logistic
Equation | y = A2 + (A1-A2)/(1 + (x/x0)^p)
Plot | response
A1 | 965,06167 ± 3,517
A2 | 891,80876 ± 1,94569
x0 | 5,44911 ± 1,75808
p | 0,56484 ± 0,08597
Reduced Chi-Sqr | 0,83156
R-Square (COD) | 0,99159
Adj. R-Square | 0,98949

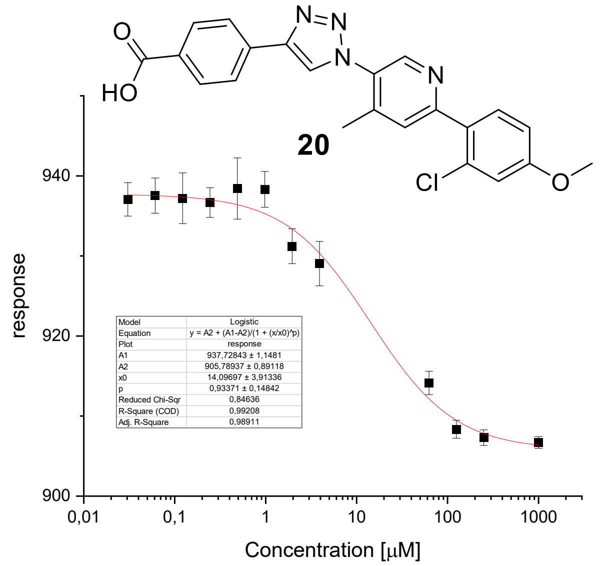
O
HO
N
N=N
N
20
Cl
O
940
920
900
response
0,01
0,1
1
10
100
1000
Concentration [µM]
Model | Logistic
Equation | y = A2 + (A1-A2)/(1 + (x/x0)^p)
Plot | response
A1 | 937,72843 ± 1,1481
A2 | 905,78937 ± 0,89118
x0 | 14,09697 ± 3,91336
p | 0,93371 ± 0,14842
Reduced Chi-Sqr | 0,84636
R-Square (COD) | 0,99208
Adj. R-Square | 0,98911

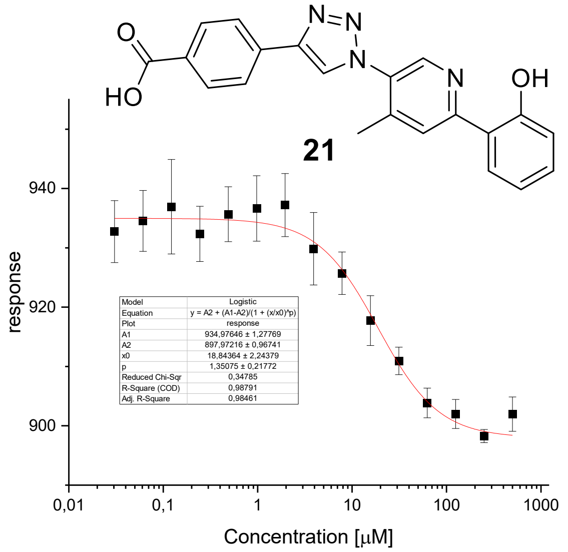
O
HO
N
N=N
N
OH
21
940
920
900
response
0,01
0,1
1
10
100
1000
Concentration [µM]
Model | Logistic
Equation | y = A2 + (A1-A2)/(1 + (x/x0)^p)
Plot | response
A1 | 934,97646 ± 1,27769
A2 | 897,97216 ± 0,96741
x0 | 18,84364 ± 2,24379
p | 1,35075 ± 0,21772
Reduced Chi-Sqr | 0,34785
R-Square (COD) | 0,98791
Adj. R-Square | 0,98461

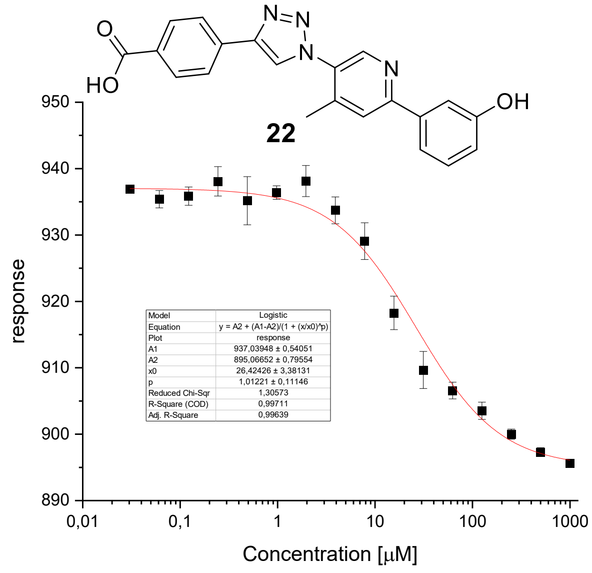
22
response
Concentration [μM]
Model Logistic
Equation y = A2 + (A1-A2)/(1 + (x/x0)^p)
Plot response
A1 937,03948 ± 0,54051
A2 895,06652 ± 0,79554
x0 26,42426 ± 3,38131
p 1,01221 ± 0,11146
Reduced Chi-Sqr 1,30573
R-Square (COD) 0,99711
Adj. R-Square 0,99639

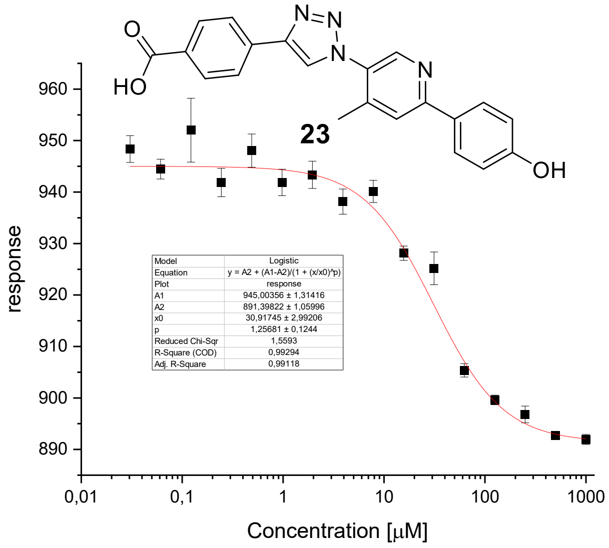
23
response
Concentration [μM]
Model Logistic
Equation y = A2 + (A1-A2)/(1 + (x/x0)^p)
Plot response
A1 945,00356 ± 1,31416
A2 891,39822 ± 1,05996
x0 30,91745 ± 2,99206
p 1,25681 ± 0,1244
Reduced Chi-Sqr 1,5593
R-Square (COD) 0,99294
Adj. R-Square 0,99118

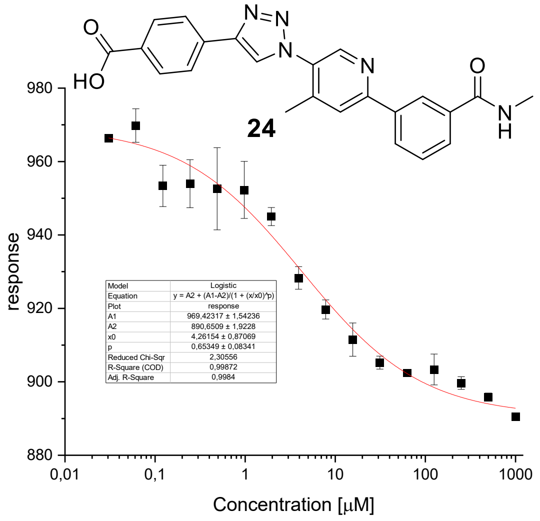
24
response
Concentration [μM]
Model Logistic
Equation y = A2 + (A1-A2)/(1 + (x/x0)^p)
Plot response
A1 969,42317 ± 1,54236
A2 890,6509 ± 1,9228
x0 4,26154 ± 0,87069
p 0,65349 ± 0,08341
Reduced Chi-Sqr 2,30556
R-Square (COD) 0,99872
Adj. R-Square 0,9984

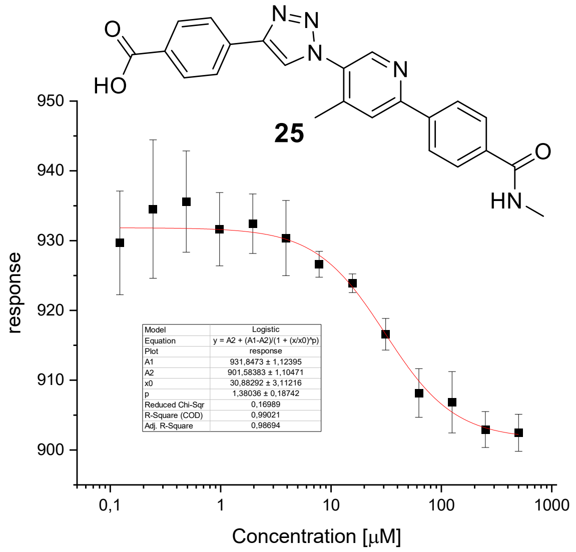
25
response
Concentration [μM]
Model Logistic
Equation y = A2 + (A1-A2)/(1 + (x/x0)^p)
Plot response
A1 931,8473 ± 1,12395
A2 901,58383 ± 1,10471
x0 30,88292 ± 3,11216
p 1,38036 ± 0,18742
Reduced Chi-Sqr 0,16989
R-Square (COD) 0,99021
Adj. R-Square 0,98694

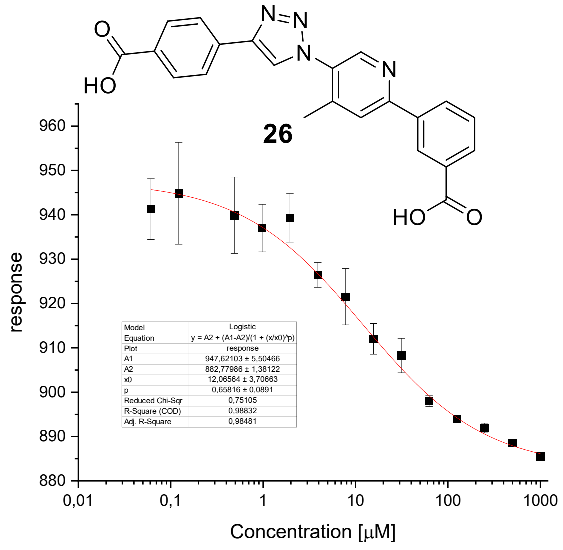
26
response
Concentration [μM]
Model Logistic
Equation y = A2 + (A1-A2)/(1 + (x/x0)^p)
Plot response
A1 947,62103 ± 5,50466
A2 882,77986 ± 1,38122
x0 12,06564 ± 3,70663
p 0,65816 ± 0,0891
Reduced Chi-Sqr 0,75105
R-Square (COD) 0,98832
Adj. R-Square 0,98481

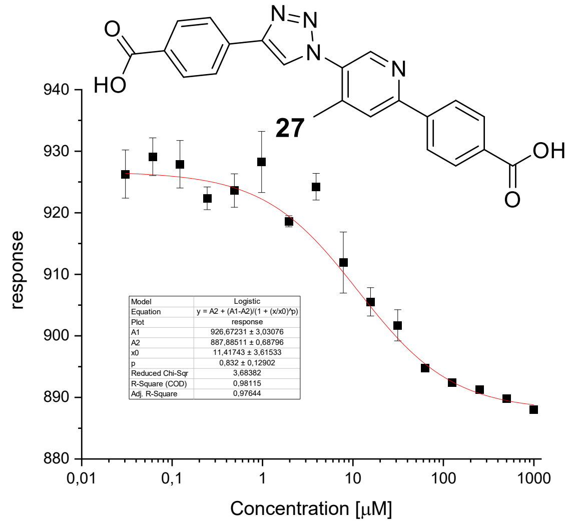
27
response
Concentration [μM]
Model Logistic
Equation y = A2 + (A1-A2)/(1 + (x/x0)^p)
Plot response
A1 926,67231 ± 3,03076
A2 887,88511 ± 0,68796
x0 11,41743 ± 3,61533
p 0,832 ± 0,12902
Reduced Chi-Sqr 3,68382
R-Square (COD) 0,98115
Adj. R-Square 0,97644

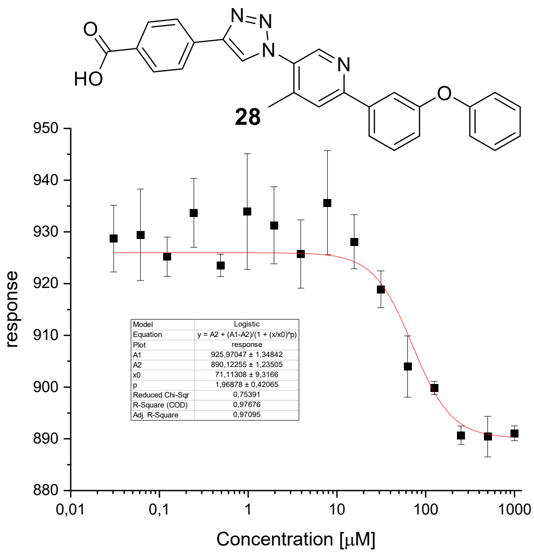
O
HO
N=N
N
N
28
950
940
930
920
910
900
890
880
response
0,01
0,1
1
10
100
1000
Concentration [µM]
Model | Logistic
Equation | y = A2 + (A1-A2)/(1 + (x/x0)^p)
Plot | response
A1 | 925,97047 ± 1,34842
A2 | 890,12255 ± 1,23505
x0 | 71,11308 ± 9,3166
p | 1,96878 ± 0,42065
Reduced Chi-Sqr | 0,75391
R-Square (COD) | 0,97676
Adj. R-Square | 0,97095

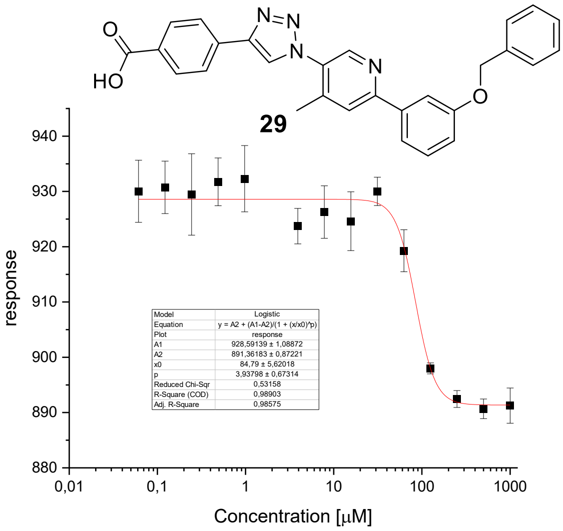
O
HO
N=N
N
N
O
29
940
930
920
910
900
890
880
response
0,01
0,1
1
10
100
1000
Concentration [µM]
Model | Logistic
Equation | y = A2 + (A1-A2)/(1 + (x/x0)^p)
Plot | response
A1 | 928,59139 ± 1,08872
A2 | 891,36183 ± 0,87221
x0 | 84,79 ± 5,62018
p | 3,93798 ± 0,67314
Reduced Chi-Sqr | 0,53158
R-Square (COD) | 0,98903
Adj. R-Square | 0,98575

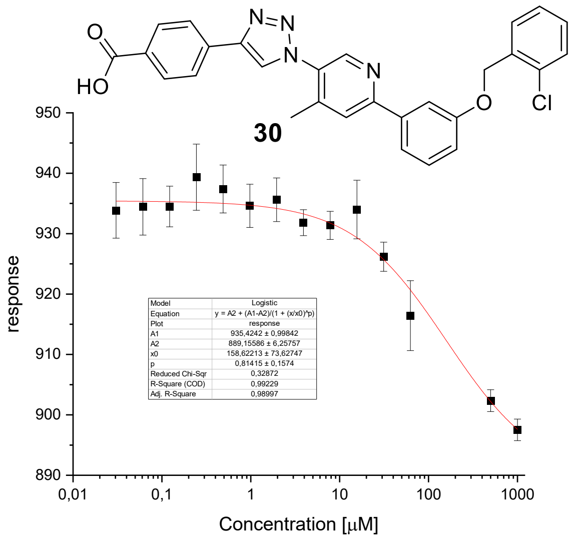
O
HO
N=N
N
N
O
Cl
30
950
940
930
920
910
900
890
response
0,01
0,1
1
10
100
1000
Concentration [µM]
Model | Logistic
Equation | y = A2 + (A1-A2)/(1 + (x/x0)^p)
Plot | response
A1 | 935,4242 ± 0,99842
A2 | 889,15586 ± 6,25757
x0 | 158,62213 ± 73,62747
p | 0,81415 ± 0,1574
Reduced Chi-Sqr | 0,32872
R-Square (COD) | 0,99229
Adj. R-Square | 0,98997

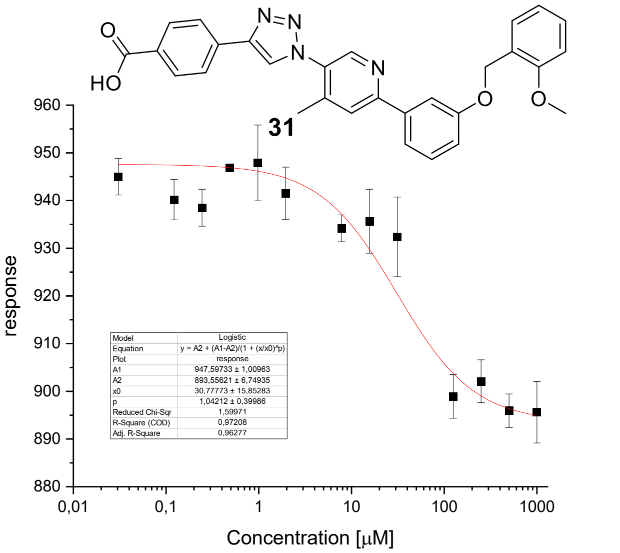
O
HO
N=N
N
N
O
O
31
960
950
940
930
920
910
900
890
880
response
0,01
0,1
1
10
100
1000
Concentration [µM]
Model | Logistic
Equation | y = A2 + (A1-A2)/(1 + (x/x0)^p)
Plot | response
A1 | 947,59733 ± 1,00963
A2 | 893,55621 ± 6,74935
x0 | 30,77773 ± 15,85283
p | 1,04212 ± 0,39986
Reduced Chi-Sqr | 1,59971
R-Square (COD) | 0,97208
Adj. R-Square | 0,96277

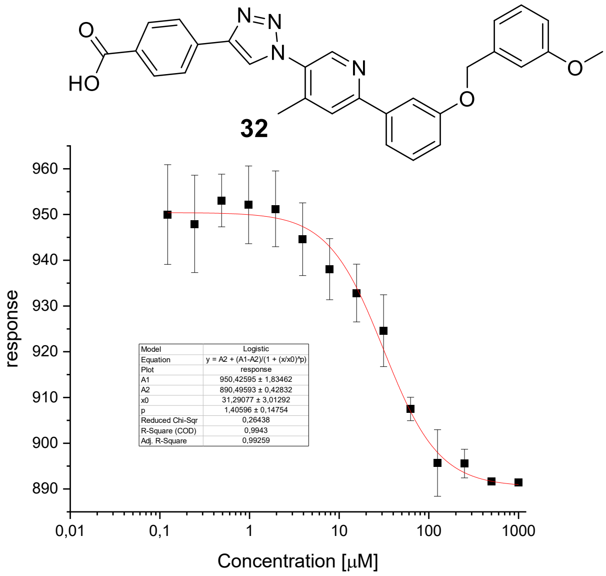
O
HO
N=N
N
N
O
O
32
960
950
940
930
920
910
900
890
response
0,01
0,1
1
10
100
1000
Concentration [µM]
Model | Logistic
Equation | y = A2 + (A1-A2)/(1 + (x/x0)^p)
Plot | response
A1 | 950,42595 ± 1,83462
A2 | 890,49593 ± 0,42832
x0 | 31,29077 ± 3,01292
p | 1,40596 ± 0,14754
Reduced Chi-Sqr | 0,26438
R-Square (COD) | 0,9943
Adj. R-Square | 0,99259

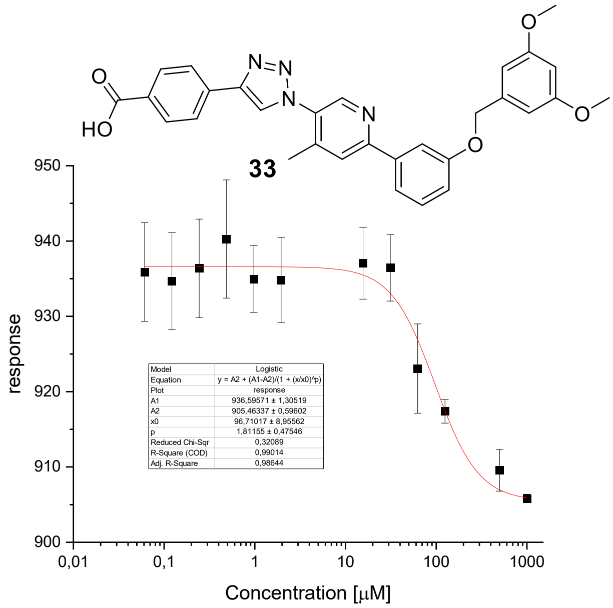
O
O
HO
N=N
N
N
O
O
33
950
940
930
920
910
900
response
0,01
0,1
1
10
100
1000
Concentration [µM]
Model | Logistic
Equation | y = A2 + (A1-A2)/(1 + (x/x0)^p)
Plot | response
A1 | 936,59571 ± 1,30519
A2 | 905,46337 ± 0,59602
x0 | 96,71017 ± 8,95562
p | 1,81155 ± 0,47546
Reduced Chi-Sqr | 0,32089
R-Square (COD) | 0,99014
Adj. R-Square | 0,98644

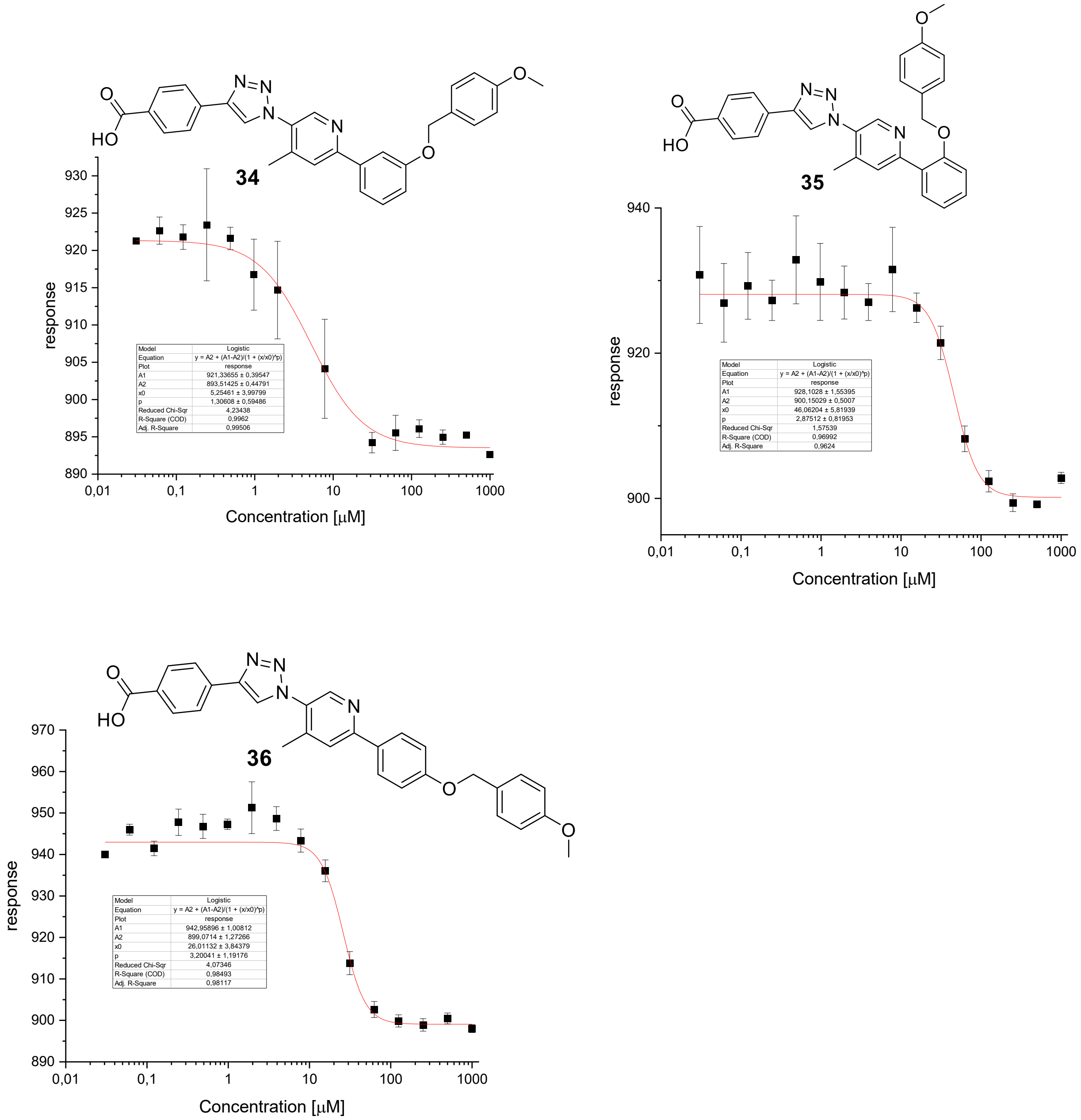

Fig S1: Binding affinity curves *via* MST for all compounds. Values are means of at least three replicates.

1.4 Electrophoretic Mobility Shift Assay (EMSA)

The EMSA was carried out with slight modifications as described previously [2] based on the assay in Hellert et al. (2013). [3] His-tagged oligomerization-deficient LANA DBD (aa1008-1146) mutant protein or LANA DBD (aa934-1162) wild type protein (200 nM final) was incubated with a 5´-Dy682-labeled double stranded DNA probe (IBA Lifesciences) (20 nM final) for 30 minutes at RT in the dark. The reaction buffer consisted of 30 mM Tris HCl pH 7.5, 50 mM KCl, 10 mM MgCl2, 1 mM DTT, 1 mM EDTA, 10% glycerol, 0.25% Tween20, 0.5 mg/mL BSA and 0.05 mg/mL poly(dI-dC). After the initial incubation period 14 µL of the reaction was added to one microliter of DMSO or the compounds (at a concentration to reach the final desired concentration in the final volume of 15 µL) and incubated for another 30 minutes at RT in the

dark. 10 µL of the reaction was run on a pre-run native 5% acrylamide gel for 45 minutes at 100V with tris-borate-EDTA buffer. Images of the gels were acquired and analysed with the Odyssey (Licor) using the Image Studio software.

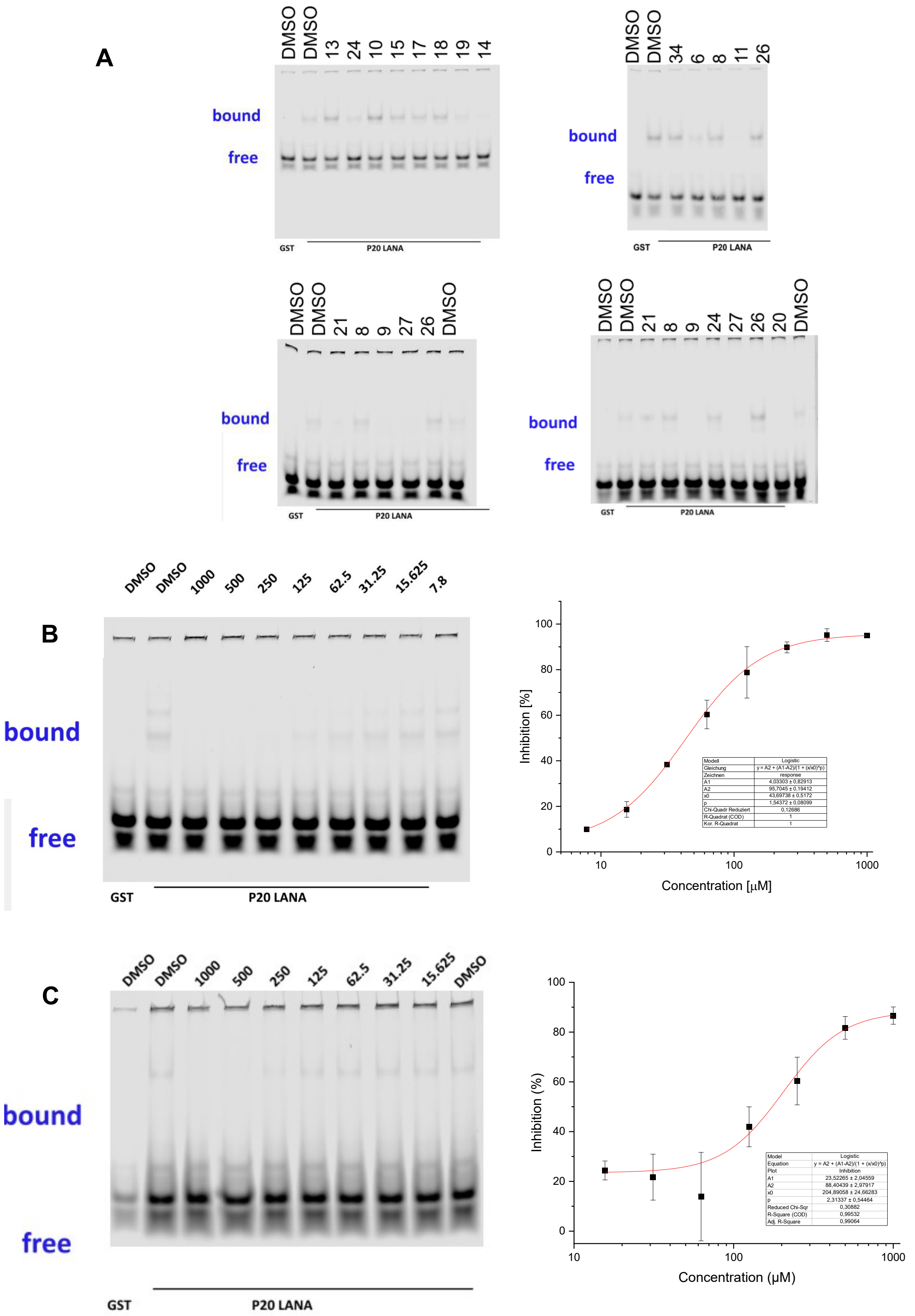

Fig S2: Gels of EMSA experiments at 250 µM for all compounds (**A**) and dose-dependent curves for compound **20** using LANA DBD mutant (**B**) and LANA wildtype (**C**) as well as LBS1. Values are means of three replicates.

1.5 Kinetic Solubility

The desired compounds were sequentially diluted in DMSO in a 96-well plate. 1.5 µL of each well were transferred into another 96-well plate and mixed with 148.5 µL of PBS. Plates were shaken for 5 min at 600 rpm at room temperature (r.t.), and the absorbance at 620 nm was measured. Absorbance values were normalized by blank subtraction and plotted using GraphPad Prism 8.4.2 (GraphPad Software, San Diego, CA, USA). Solubility (S) was determined based on the First X value of AUC function using a threshold of 0.005. Values are means of two replicates.

1.6 Chromatographic LogD

$LogD_{7.4}$ was analyzed using an HPLC−MS based method. The retention time of 12 compounds with known $LogD_{7.4}$ was determined and plotted toward their $LogD_{7.4}$. Linear regression was used to determine the $LogD_{7.4}$ of unknown compounds. Analysis was performed using a Dionex Ultimate 3000 HPLC system coupled to a TSQ Quantum Access MAX (Thermo Fisher, Dreieich, Germany) with the following conditions: EC150/2 NUCLEODUR C18 Pyramid column, 5 µM (Macherey Nagel, Düren, Germany); eluent A: 50 mM NH4OAc pH 7.4, eluent B: acetonitrile, and flow: 0.6 mL/min. The gradient was set to 0−100% B from 0 to 2.5 min, 100% B from 2.5 to 3.0 min, 100−0% B from 3.0 to 3.2 min, and 0% B from 3.2−5.0. Values are means of two replicates.

1.7 Metabolic Stability in Liver S9 Fractions

For the evaluation of combined phase I and phase II metabolic stability, the compound (1 µM) was incubated with 1 mg/mL pooled mouse liver S9 fraction (Xenotech, Kansas City, USA) or human liver S9 fraction (Corning, USA), 2 mM NADPH, 1 mM UDPGA, 10 mM $MgCl_2$, 5 mM GSH and 0.1 mM PAPS at 37 °C for 240 min. The metabolic stability of testosterone, verapamil and ketoconazole were determined in parallel to confirm the enzymatic activity of mouse S9 fractions, for human S9 testosterone, diclofenac and propranolol were used. The incubation was stopped after defined time points by precipitation of aliquots of S9 enzymes with 2 volumes of cold acetonitrile containing internal standard (150 nM diphenhydramine). Samples were stored on ice until the end of the incubation and precipitated protein was removed by centrifugation (15 min, 4 °C, 4,000 g). Concentration of the remaining test compound at the different time points was analyzed by HPLC-MS/MS (TSQ Quantum Access MAX, Thermo Fisher, Dreieich, Germany) and used to determine half-life ($t_{1/2}$). Values are means of three replicates.

1.8 Cytotoxicity

Caco-2: Cytotoxicity of the compounds was assessed in Caco2 (HTB-37) cells, passages 8 to 22 using a MTT assay. 20,000 cells per well were seeded in 96 well plates and allowed to grow for 3 days, in DMEM medium 10% FCS and 1% NEAA. Cells were exposed to the compounds in Hanks Buffered Salt Solution (HBSS) in the concentration range of 3.125 – 200µM. Control samples were untreated cells as live control (100% viability) and 1% Trition-X100 as dead control (0% viability). After 4 h incubation time the samples were removed, cells were washed with HBSS and medium containing 10% (v/v) 3-(4,5-dimethylthiazol-2-yl)-2,5-diphenyltetrazolium bromide (MTT reagent) added. After a further 4 h incubation time the medium was removed and cells were lysed with DMSO. Absorbance at 550nm was read with a plate reader. Cell viability was calculated as relative percentage compared to untreated cells.

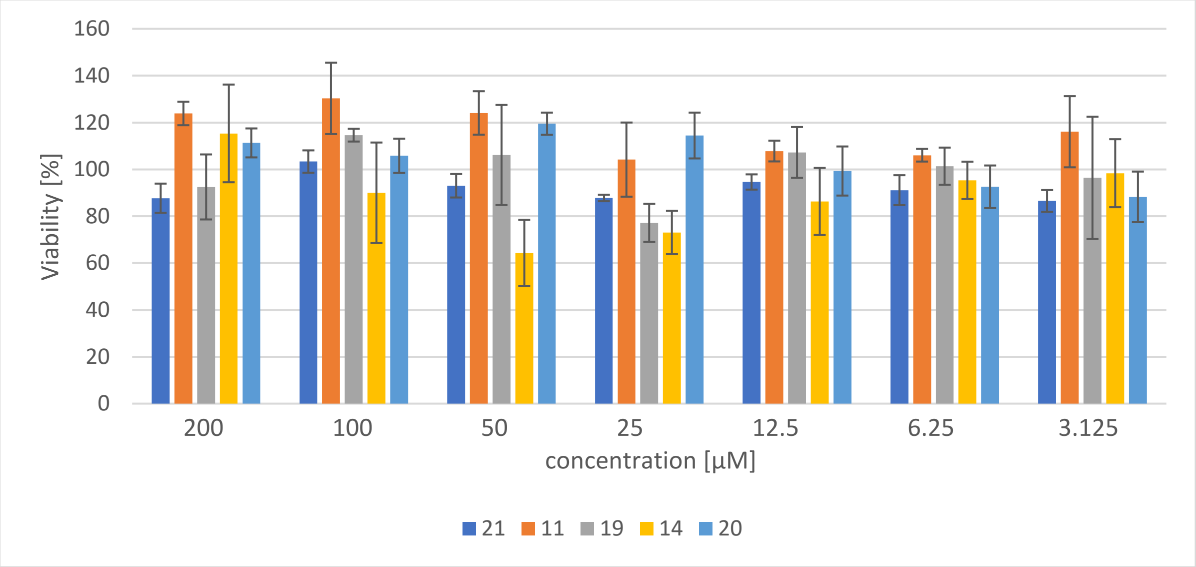

Fig S3: MTT assay for compounds **11**, **14**, **19**, **20** and **21** using Caco-2 cells. Values are means of three replicates.

HEK293: 3 x 10^4 HEK293 cells were seeded per well in 100 µL DMEM 10% FCS in 96-well plates with the indicated dilutions of compound or DMSO and incubated in an humidified incubator at 37°C, 5% CO_2. After 48 hours 50 µL of media supplemented with 333 µg/mL thiazolyl blue was added per well and the plates were incubated for an additional two hours in an humidified incubator at 37°C, 5% CO_2, to allow the cells to metabolize the dye. To end the incubation the supernatant was removed and the formazan solubilized in 50 µL acidified isopropanol (40 mM HCl) on a shaker at room temperature, covered with foil for 30 minutes. The absorbance was read with a Cytation3 plate reader at 595/630 nm. The reference wavelength (OD630) was substracted from the OD595 values and the resulting data plotted with Excel (**Fig. S4**).

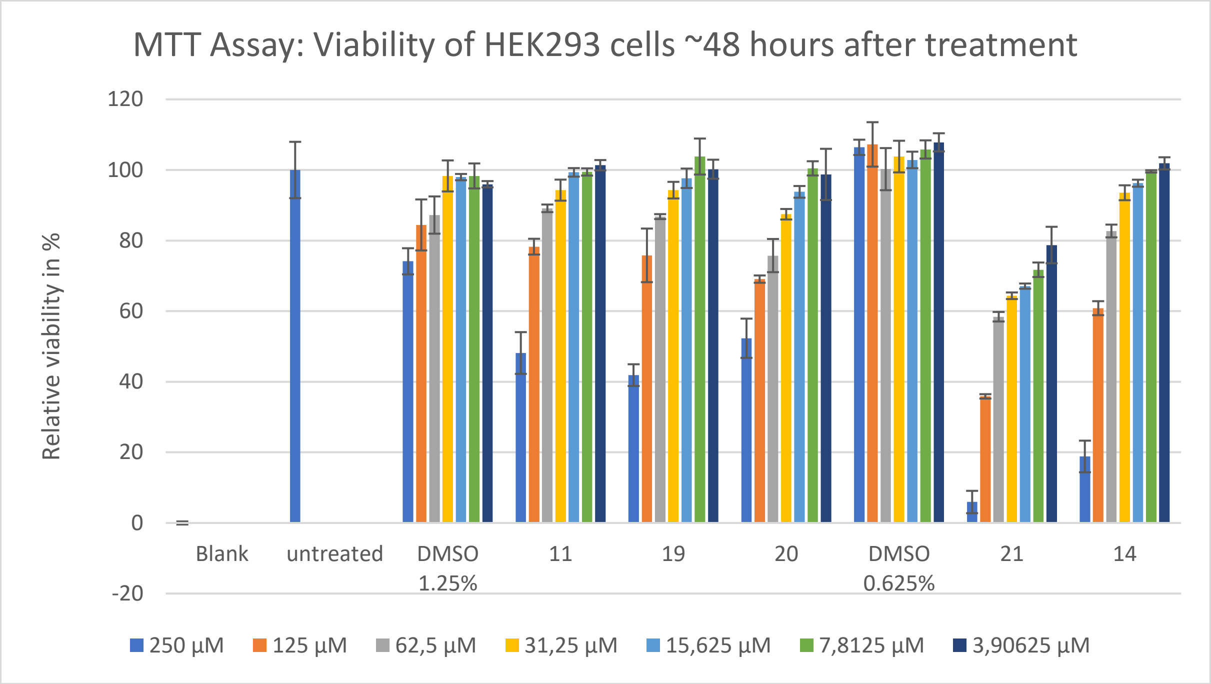

Fig S4: MTT assay for compounds **11**, **14**, **19**, **20** and **21** using HEK293 cells. Values are means of three replicates.

1.9 Cell permeability

Permeability of the compounds was assessed using Caco2 monolayers grown on Transwell® (3460 Costar, Corning). In brief, cells were seeded 60.000 cells/well in 12-well Transwell plates in DMEM medium supplemented with 10% FCS and 1% NEAA. Cells were grown for 7 to 9 days with medium replacement every other day until they reached a tight monolayer. Tightness was defined as transepithelial resistance (TEER) values >300 Ω/cm² (measured using an EVOM Volt-Ohmmeter, World Precision Instruments). At the day of permeability assay, the compound stock solutions in DMSO were diluted to 10µM in Krebs-Ringer-buffer (KRB, pH 7.4). Cells treated with KRB only served as control. Monolayer was washed two times with prewarmed KRB and incubated with 500 µl sample on the apical compartment. Samples from the basolateral side were drawn at t = 0, 15, 30, 60, 120, 180 and 300 min and replaced with fresh buffer. The plates were incubated for 5 h in a cell culture incubator with lateral shaking (200 rpm). TEER values were measured again at the end of the permeability test. Compound concentration was determined with HPLC-ESI-MS/MS in SRM mode and calculated for the cumulative transport. Values are means of three replicates.

1.10 Replication assay

This assay is based on protocols described previously and was modified as described in the following.[4] 8 x 10^5 HEK 293 cells were plated per well in a 6-well plate. On the next day, the cells were transfected with plasmids pGTR4 or pGTR4:73 that contain four KSHV terminal repeat (TR) sequences (0.75 µg/well). pGTR4:73 in addition to the TR sequences also contains an expression cassette for the KSHV ORF73 encoding LANA, allowing replication of the TR-containing plasmid. As a non-replicating internal control, pEGFP-C1 (Clontech) was cotransfected (0.25 µg/well). Six hours after transfection the medium was replaced with medium containing either DMSO (solvent control) or the indicated compounds. On the next day the cells were split into two well of a 6-well plated into medium containing either DMSO or the same compounds as before. The cells were harvested 72 hours after transfection in lysis buffer (10 mM Tris, 10 mM EDTA, 0.6% SDS) and the chromosomal DNA precipitated with 0.85 M NaCl rolling overnight at 4°C and pelleted by centrifugation. The episomal DNA was purified by phenol-chloroform using Gel lock columns (5PRIME), precipitated with 0.3 M Na-Acetate pH 5.2 and ethanol and the pellet was dissolved in 20 µL of water. 90% of the DNA was digested for 72 hours with MfeI-HF (NEB) to linearize the plasmids and additionally with DpnI (NEB) to digest the input plasmid. 10% of the DNA was just linearized by MfeI-HF (NEB) as a control for the total DNA isolated. The digested DNA was separated on a 8% agarose gel in 1x TBE buffer and transferred to a nylon membrane (Whatman® Nytran™ SuPerCharge (SPC) nylon blotting membrane) by Southern blotting. As all three plasmids contain a GFP sequence, a probe was prepared by digesting pEGFP-C1 with AgeI-HF (NEB) and XhoI (NEB), isolating and purifying the 0.7 kb band, by agarose gel electrophoresis. The probe was labeled with the AlkPhos Direct Labeling Module (GE Healthcare/Amersham) and hybridized overnight to detect the bands of the input and replicated plasmids on the membrane. To visualize the bands the membrane was treated with the chemilumiscent CDP-Star substrate (GE Healthcare/Amersham) and imaged with the LAS-3000 Imaging System (Fuji).

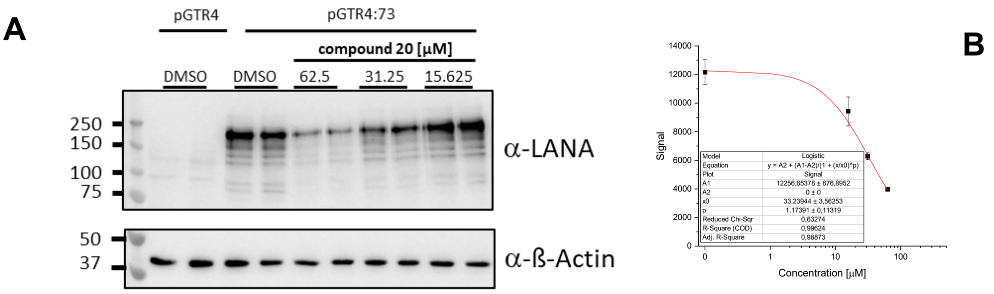

Fig S5: A) Western blot of lysates used for isolating the plasmids for the Southern blot shown in figure **4**. Western blot showing that equal numbers of cells were lysed for isolation of the plasmids for the southern blot (α-ß-Actin). Blotting for LANA shows that the LANA (required for the replication) was expressed from the transfected plasmid. Anti-HHV-8 LNA-1 antibody clone LN53 (MABE1109, Sigma-Aldrich) was used to detect LANA by western blot. Anti-ß-Actin (A5441, Sigma-Aldrich) was used as Actin antibody. **B)** Sigmoidal blot of southern blot for compound **20**. Values are means of two replicates.

2. ^{1}H and ^{13}C Spectra

4-Ethynylbenzoic acid (2)

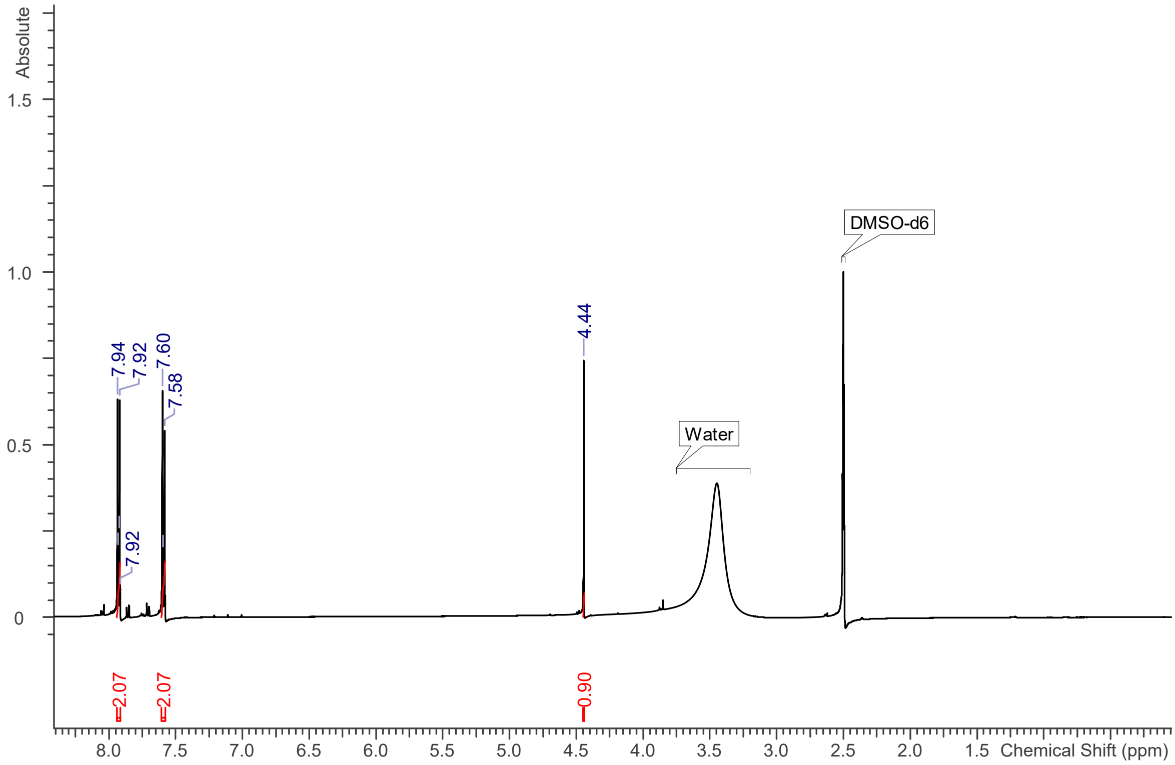

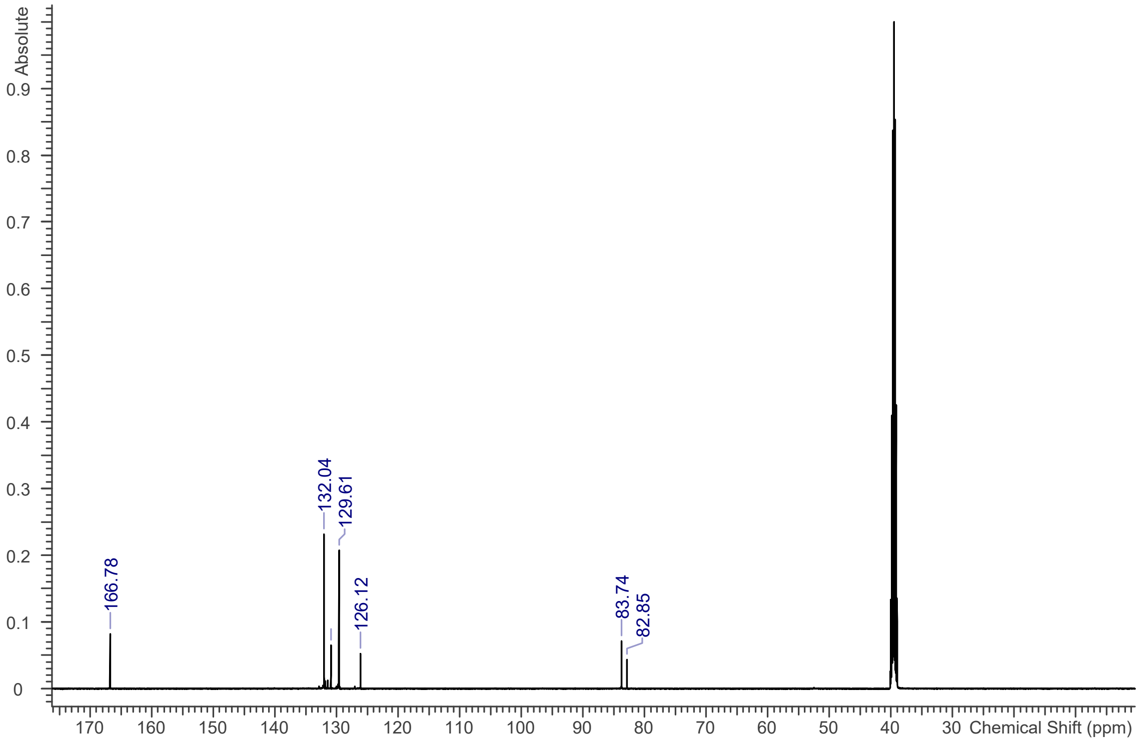

4-(1-(6-Bromo-4-methylpyridin-3-yl)-1H-1,2,3-triazol-4-yl)benzoic acid (3)

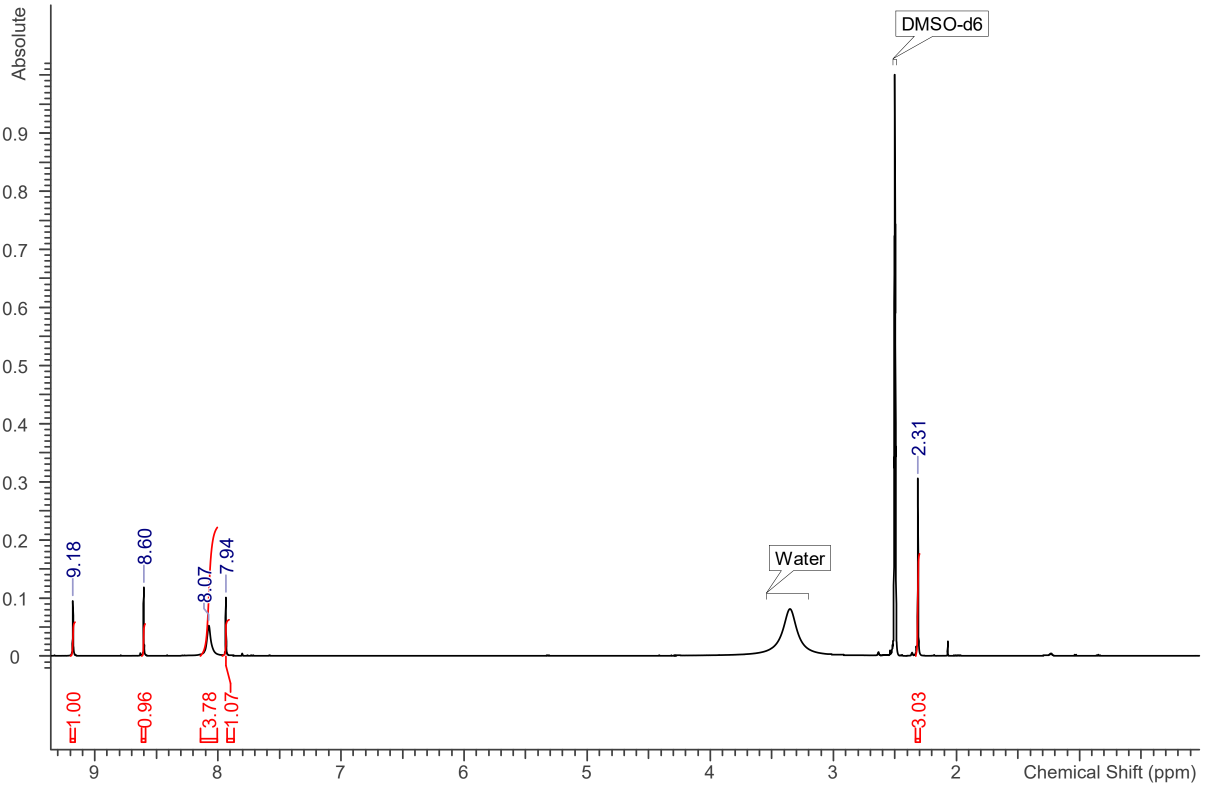

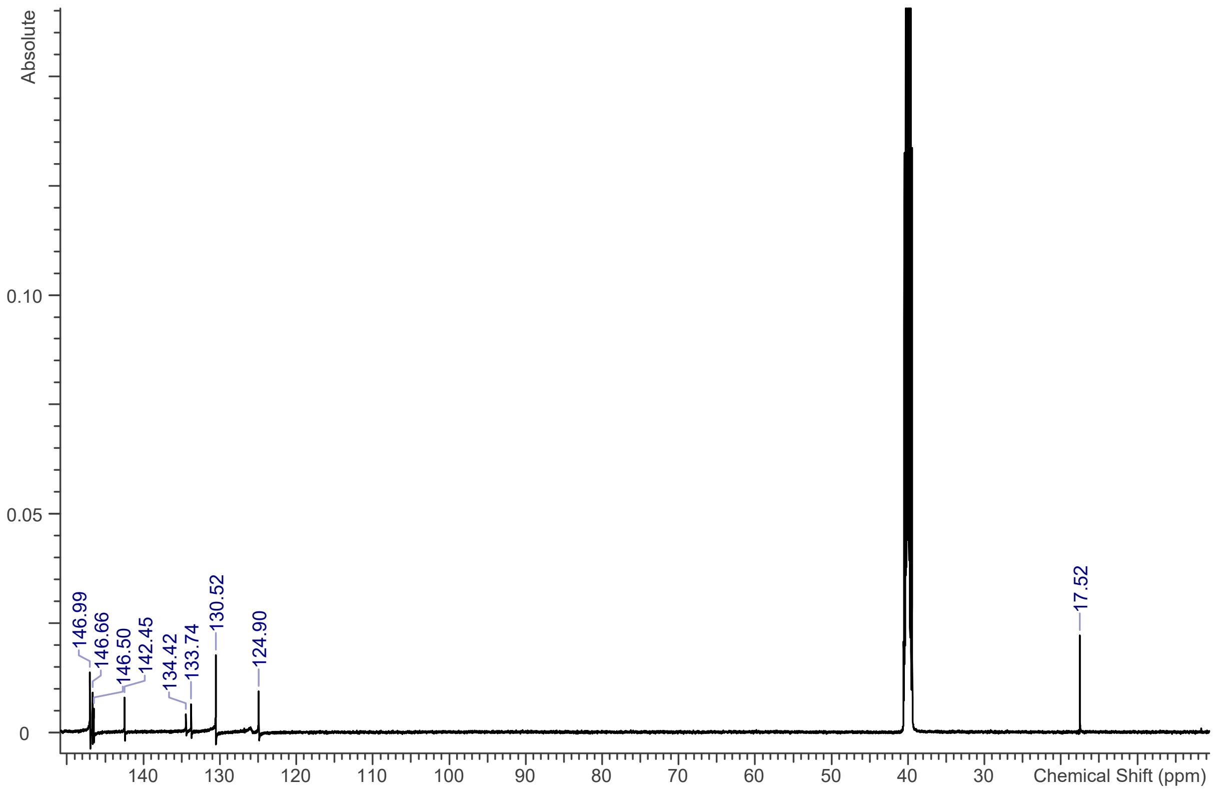

4-(1-(4-methyl-[2,3'-bipyridin]-5-yl)-1H-1,2,3-triazol-4-yl)benzoic acid (4)

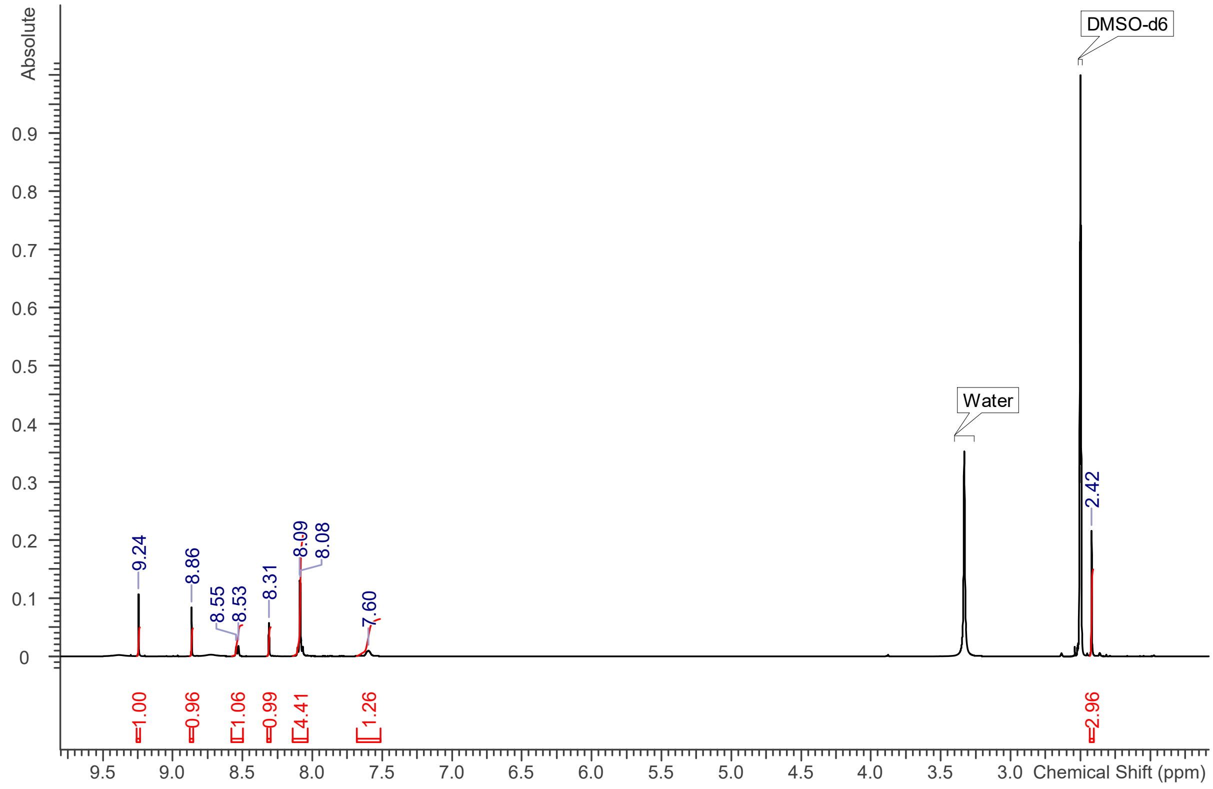

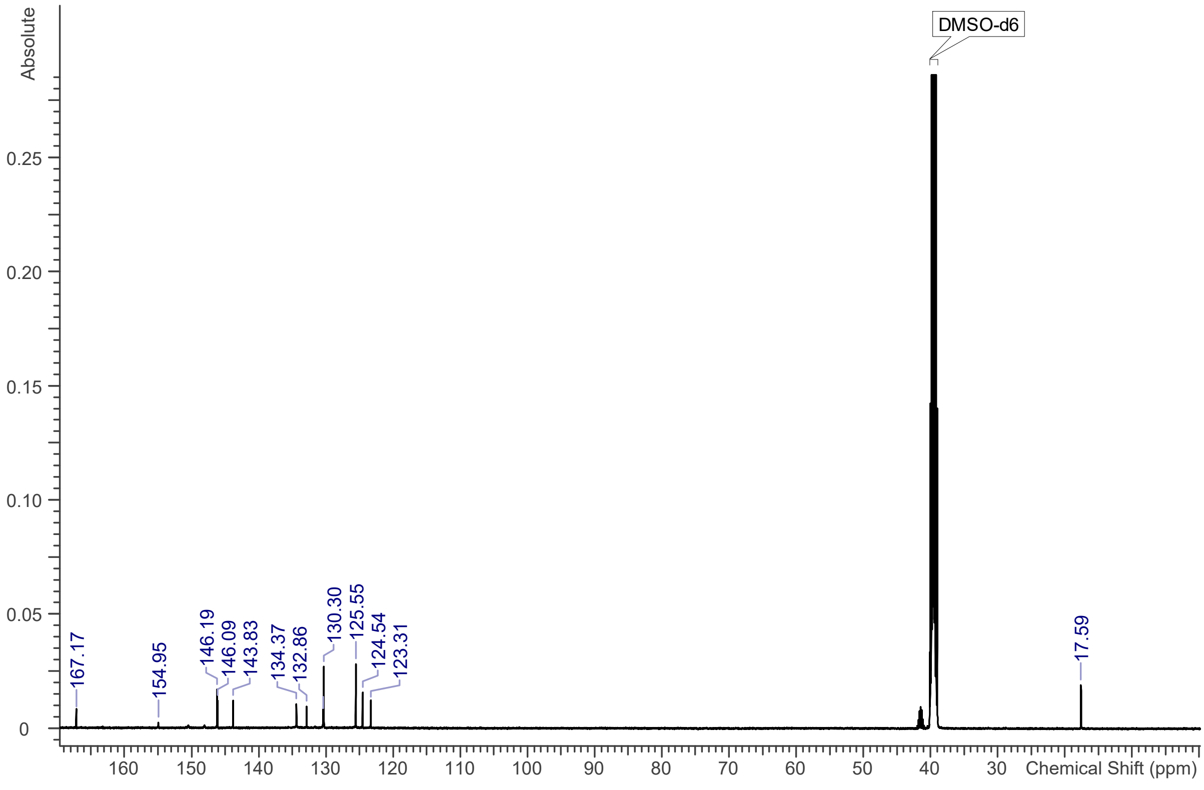

4-(1-(4-methyl-[2,4'-bipyridin]-5-yl)-1H-1,2,3-triazol-4-yl)benzoic acid (5)

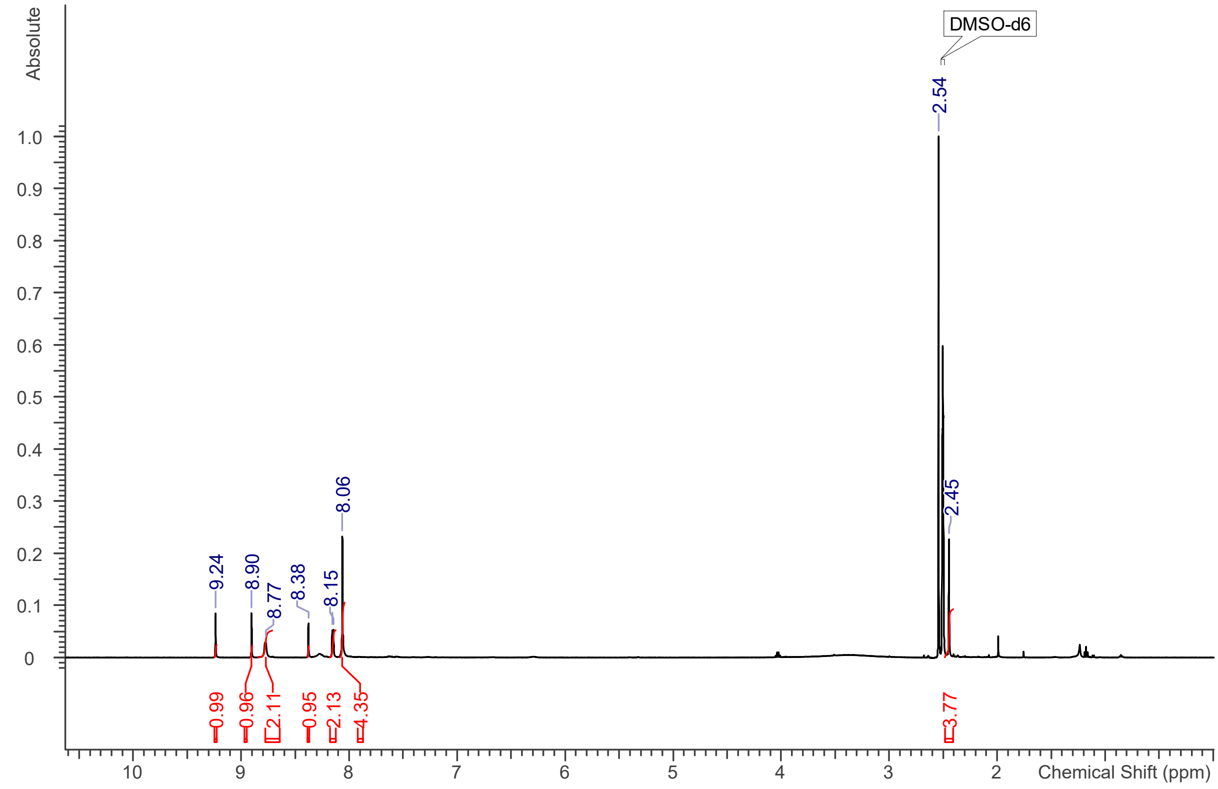

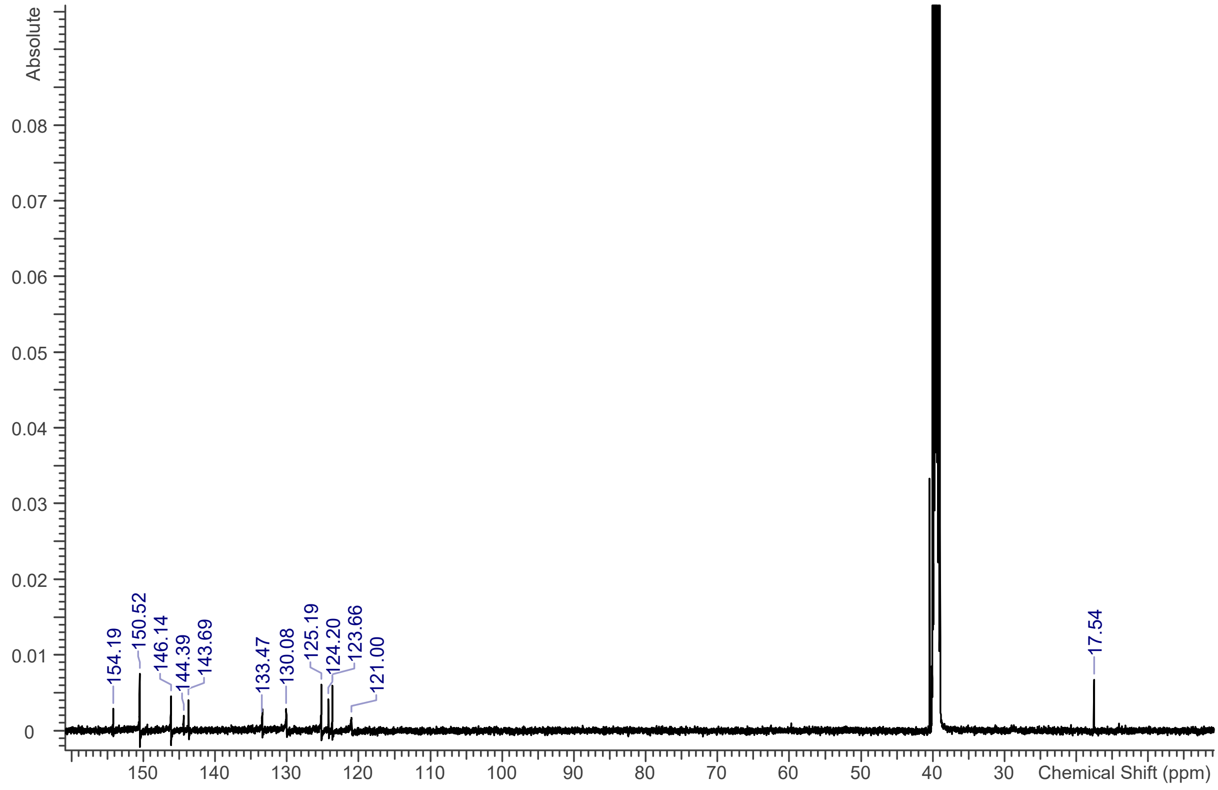

4-(1-(4-methyl-6-(pyrimidin-5-yl)pyridin-3-yl)-1H-1,2,3-triazol-4-yl)benzoic acid (6)

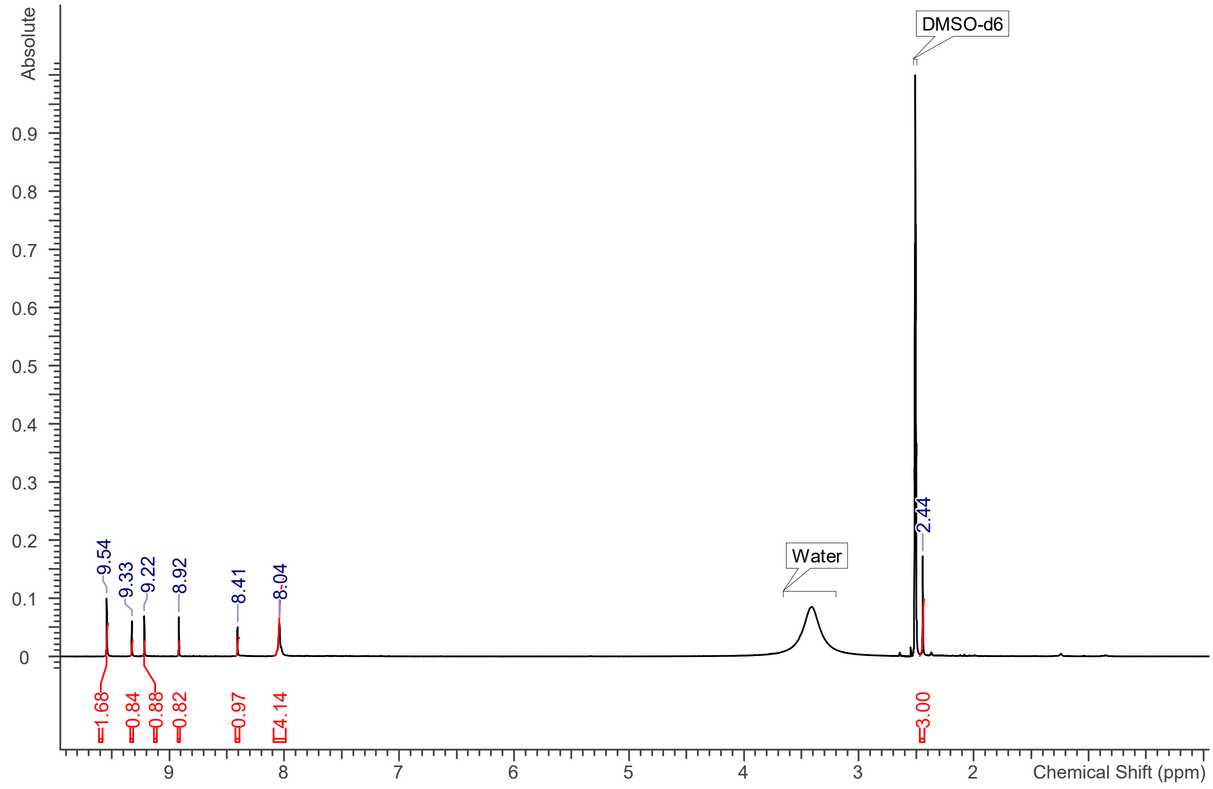

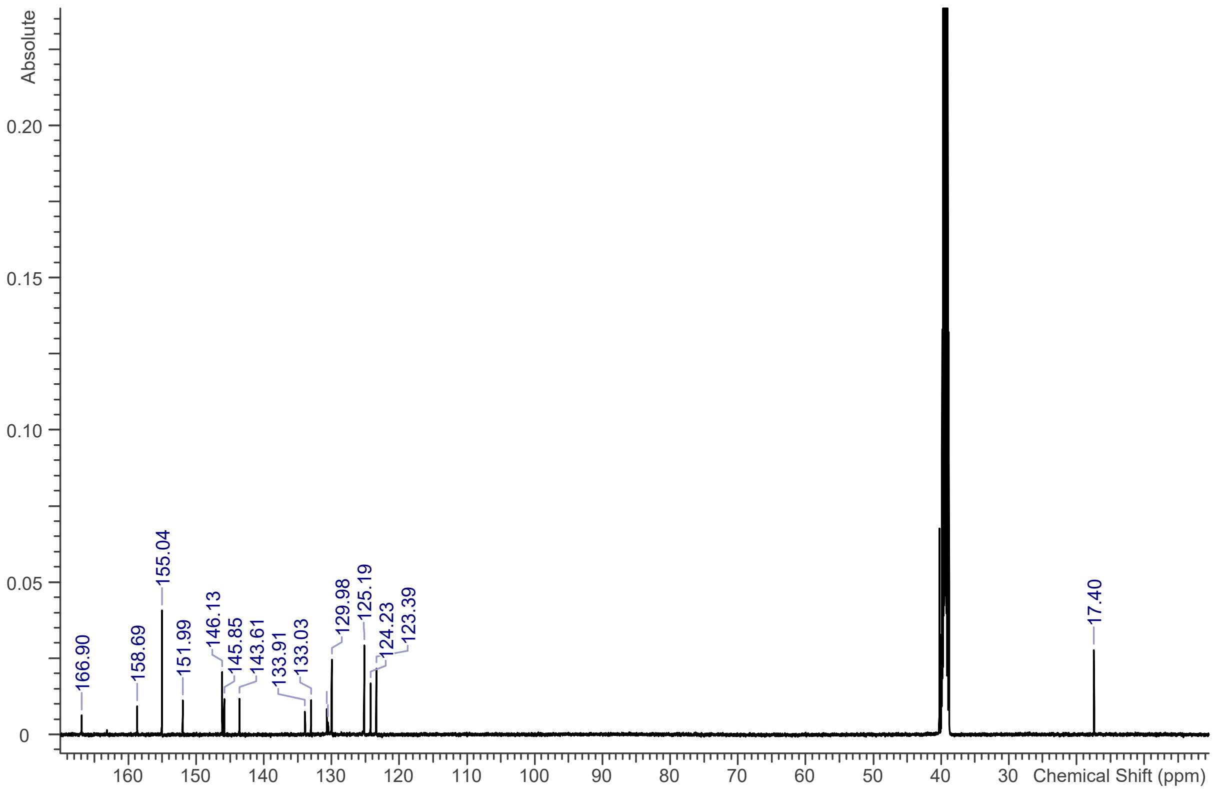

4-(1-(5'-hydroxy-4-methyl-[2,3'-bipyridin]-5-yl)-1H-1,2,3-triazol-4-yl)benzoic acid (7)

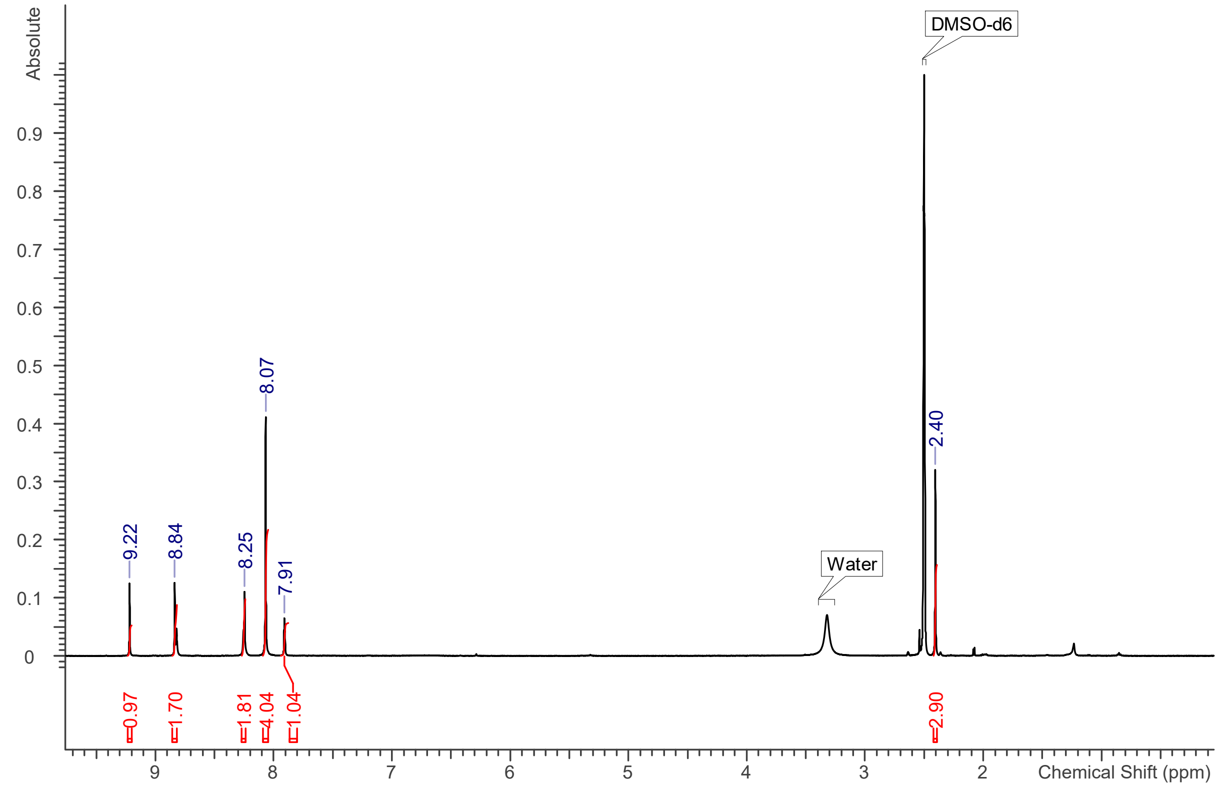

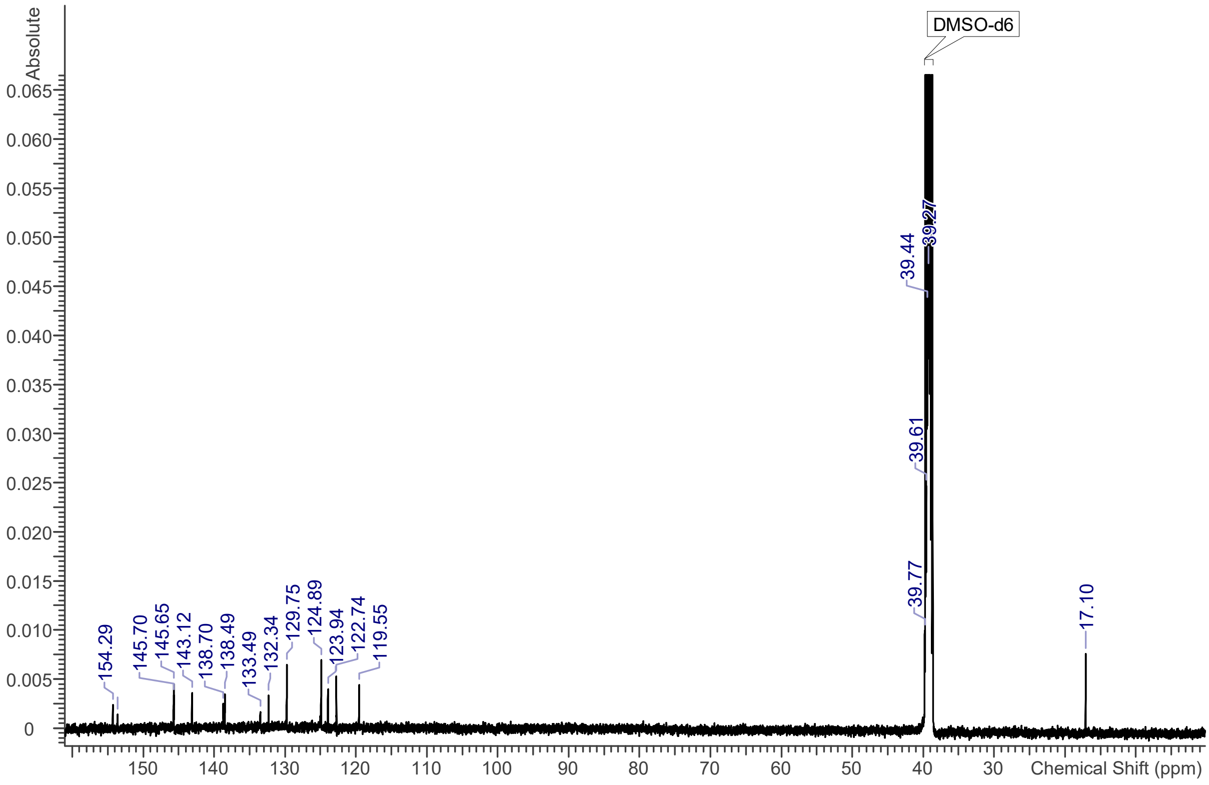

4-(1-(6'-hydroxy-4-methyl-[2,3'-bipyridin]-5-yl)-1H-1,2,3-triazol-4-yl)benzoic acid (8)

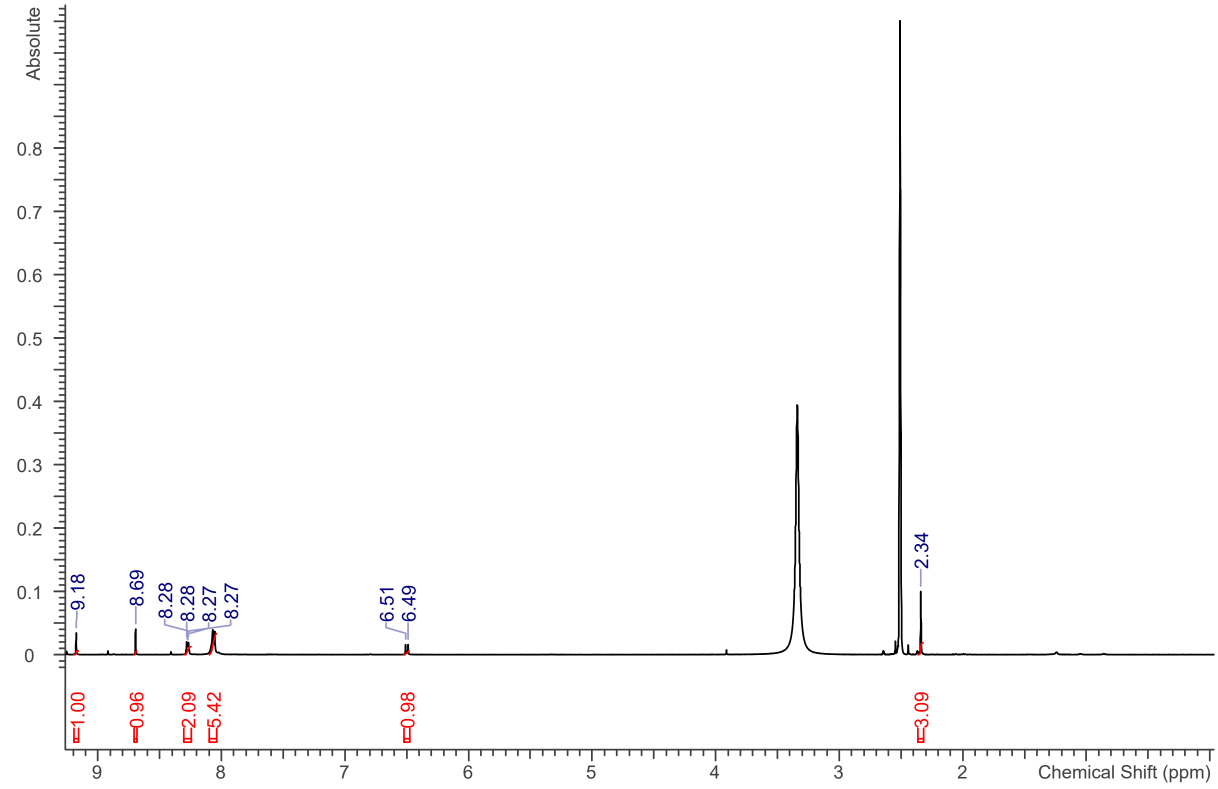

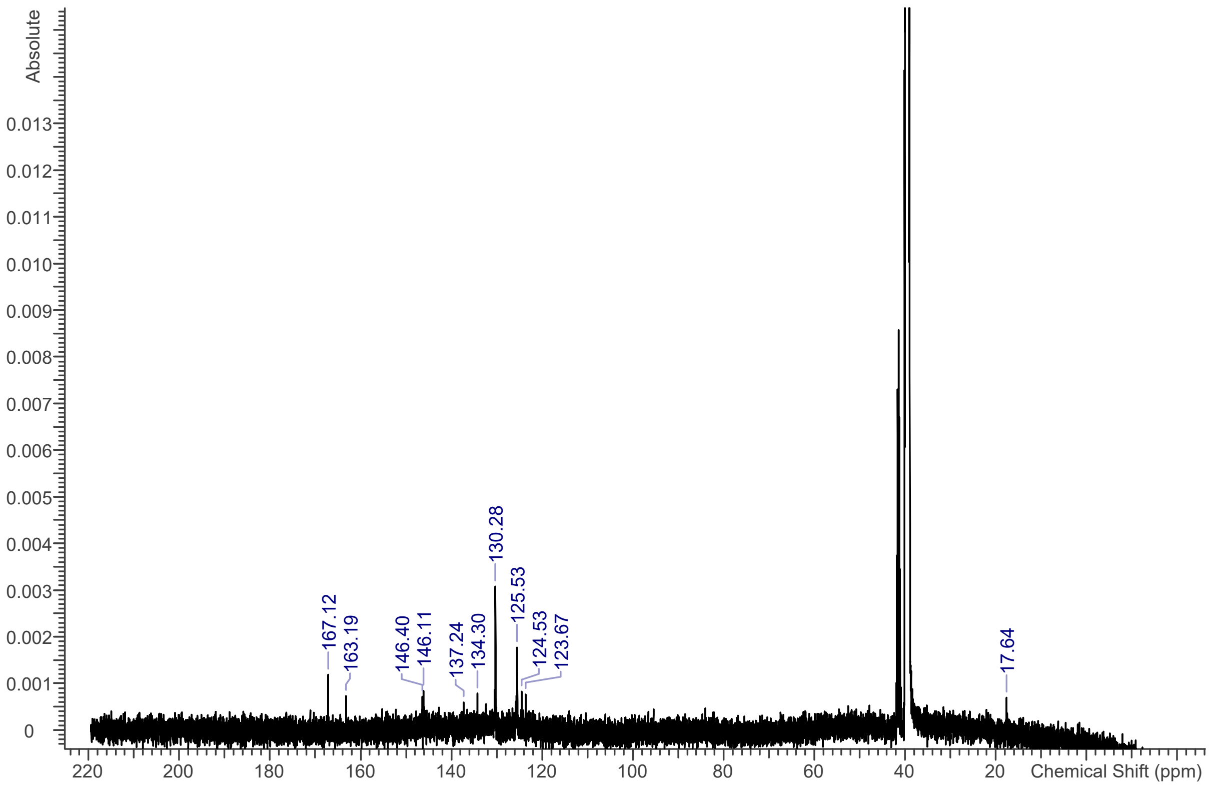

4-(1-(5'-methoxy-4-methyl-[2,3'-bipyridin]-5-yl)-1H-1,2,3-triazol-4-yl)benzoic acid (9)

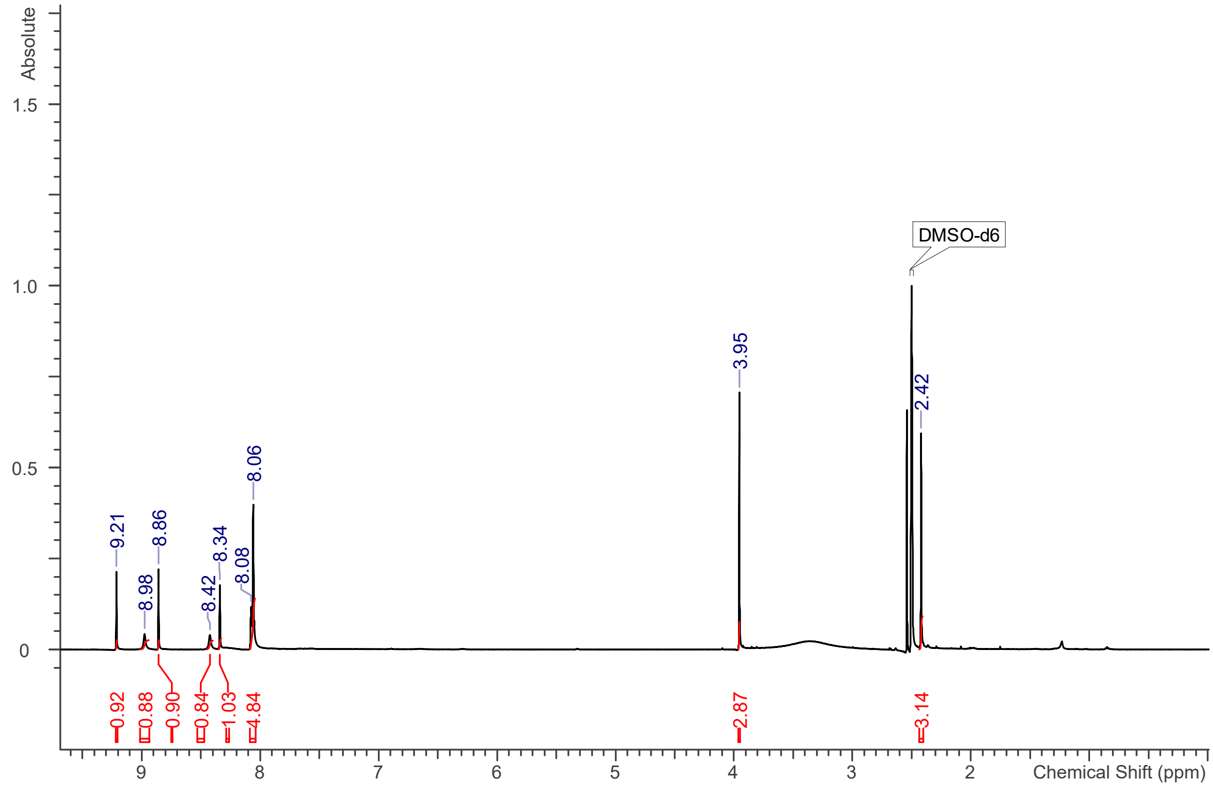

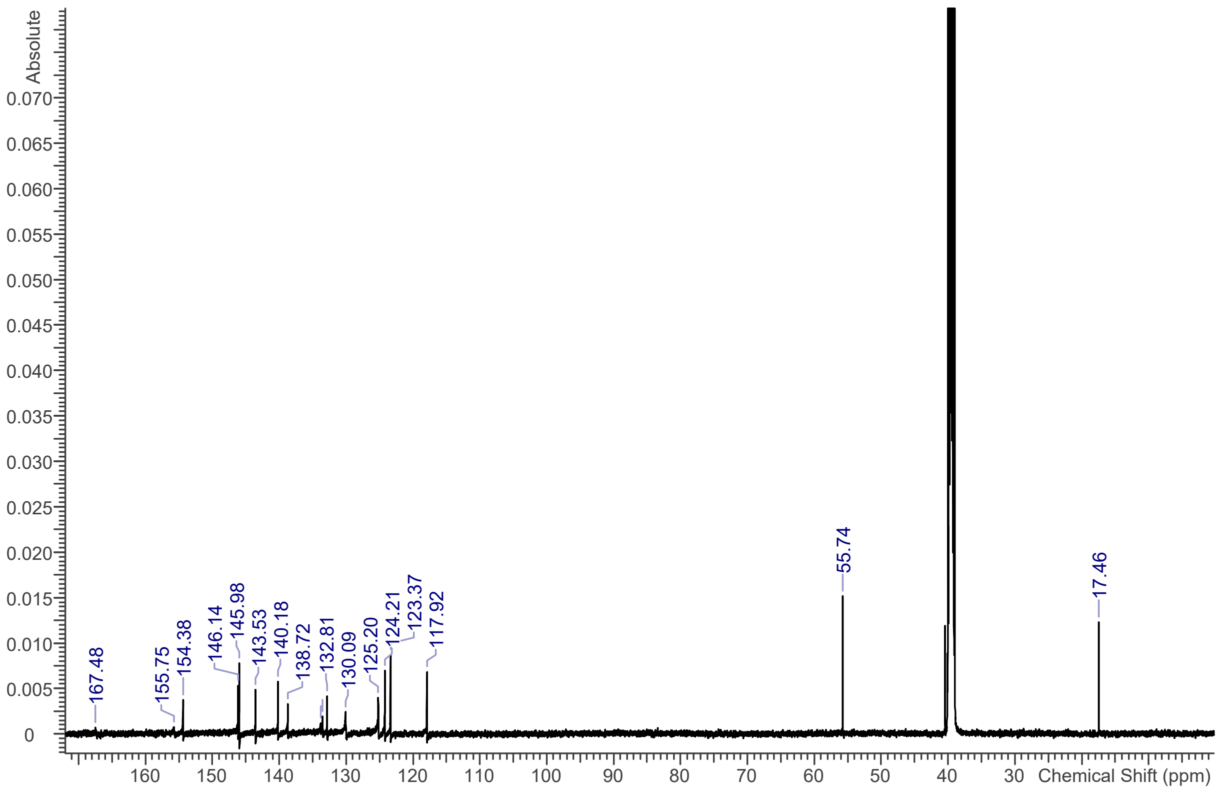

4-(1-(6'-methoxy-4-methyl-[2,3'-bipyridin]-5-yl)-1H-1,2,3-triazol-4-yl)benzoic acid (10)

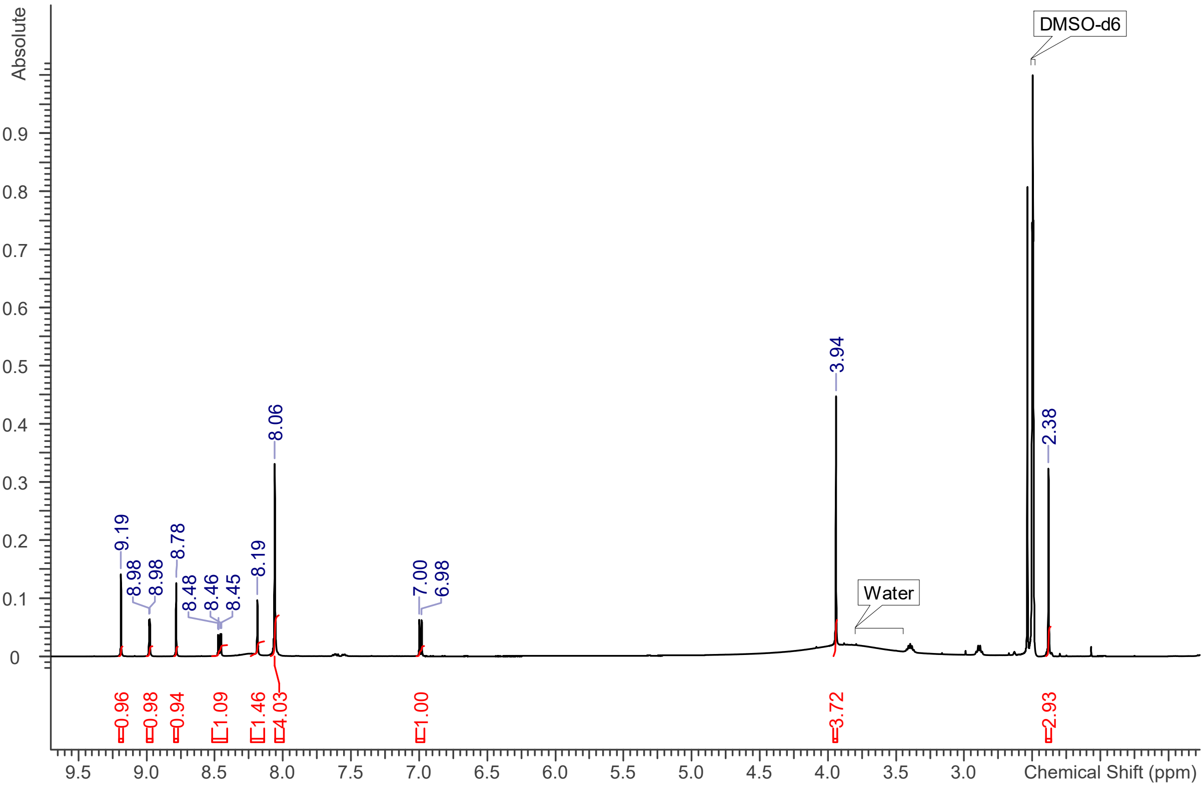

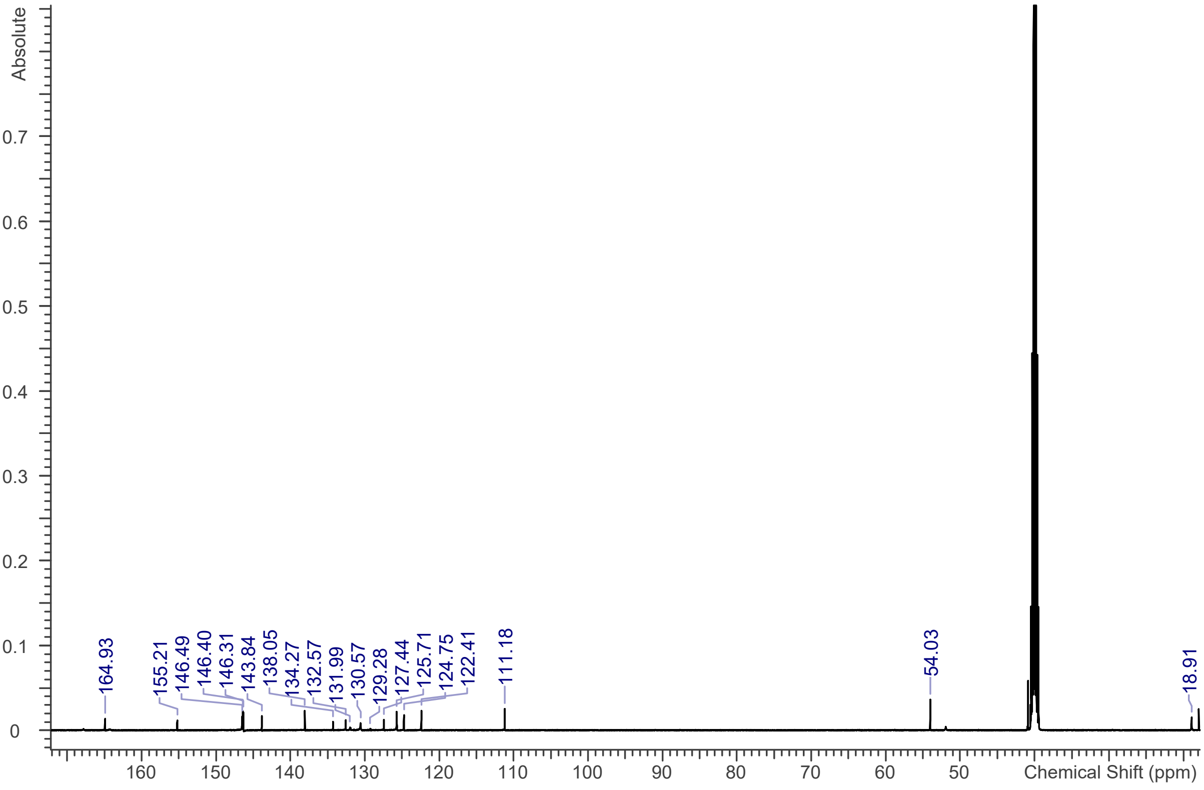

4-(1-(2'-methoxy-4,5'-dimethyl-[2,3'-bipyridin]-5-yl)-1H-1,2,3-triazol-4-yl)benzoic acid (11)

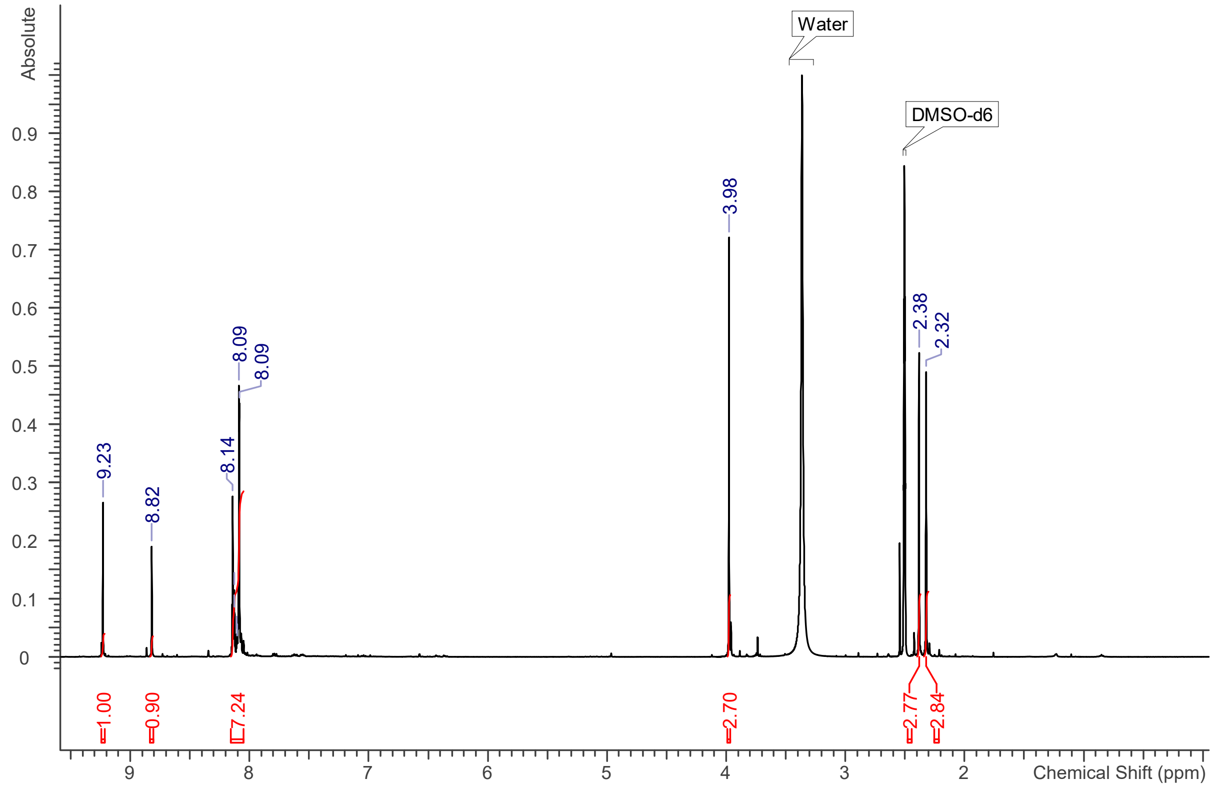

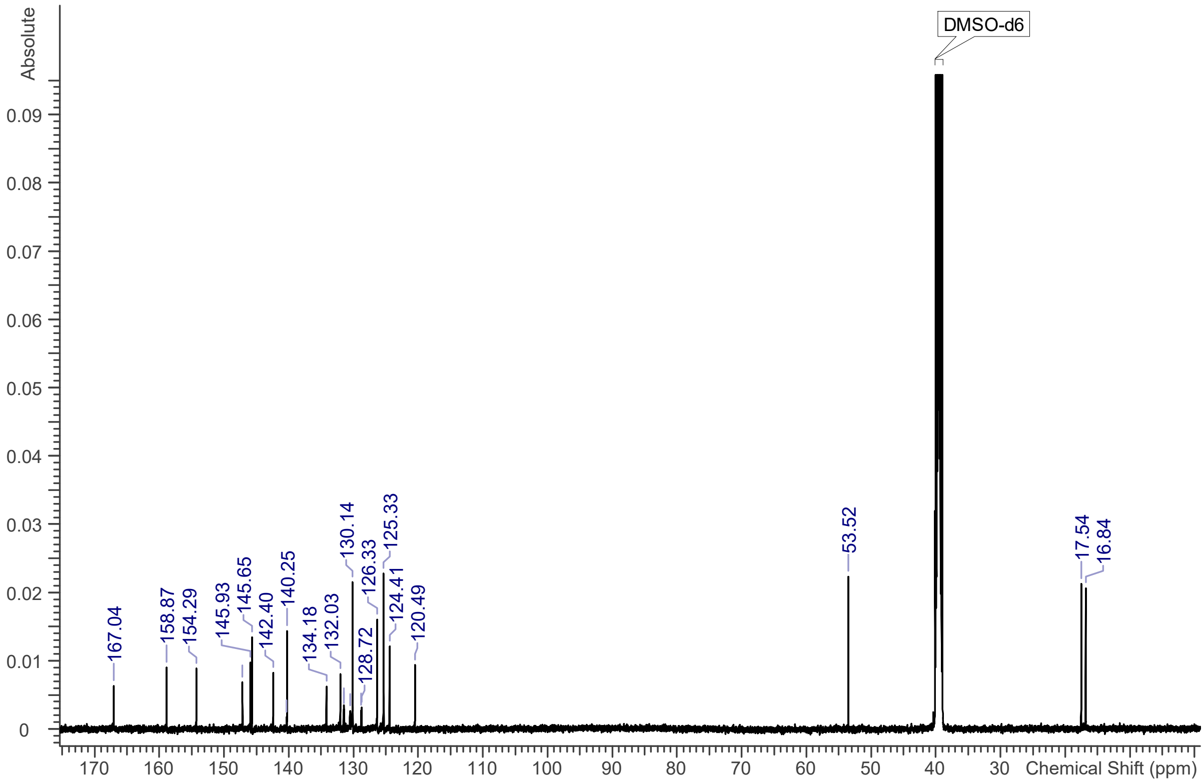

4-(1-(4-methyl-6-(quinolin-3-yl)pyridin-3-yl)-1H-1,2,3-triazol-4-yl)benzoic acid (12)

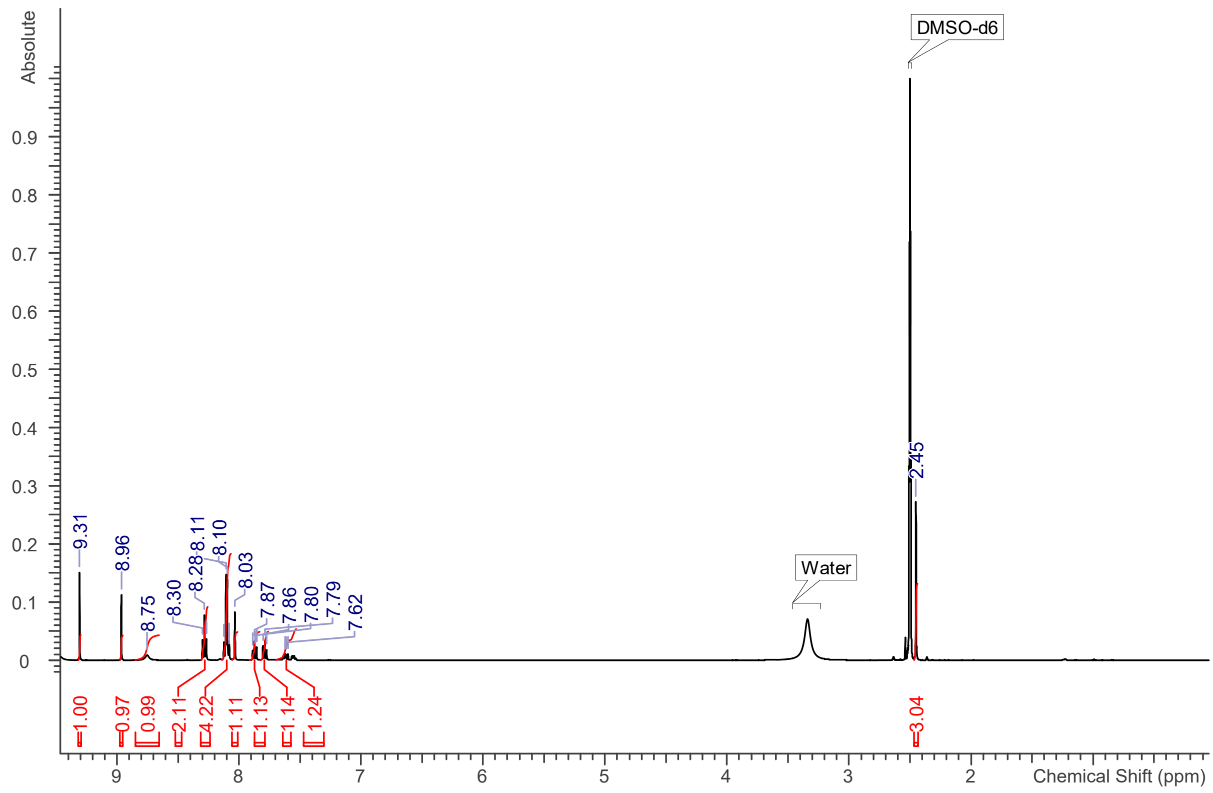

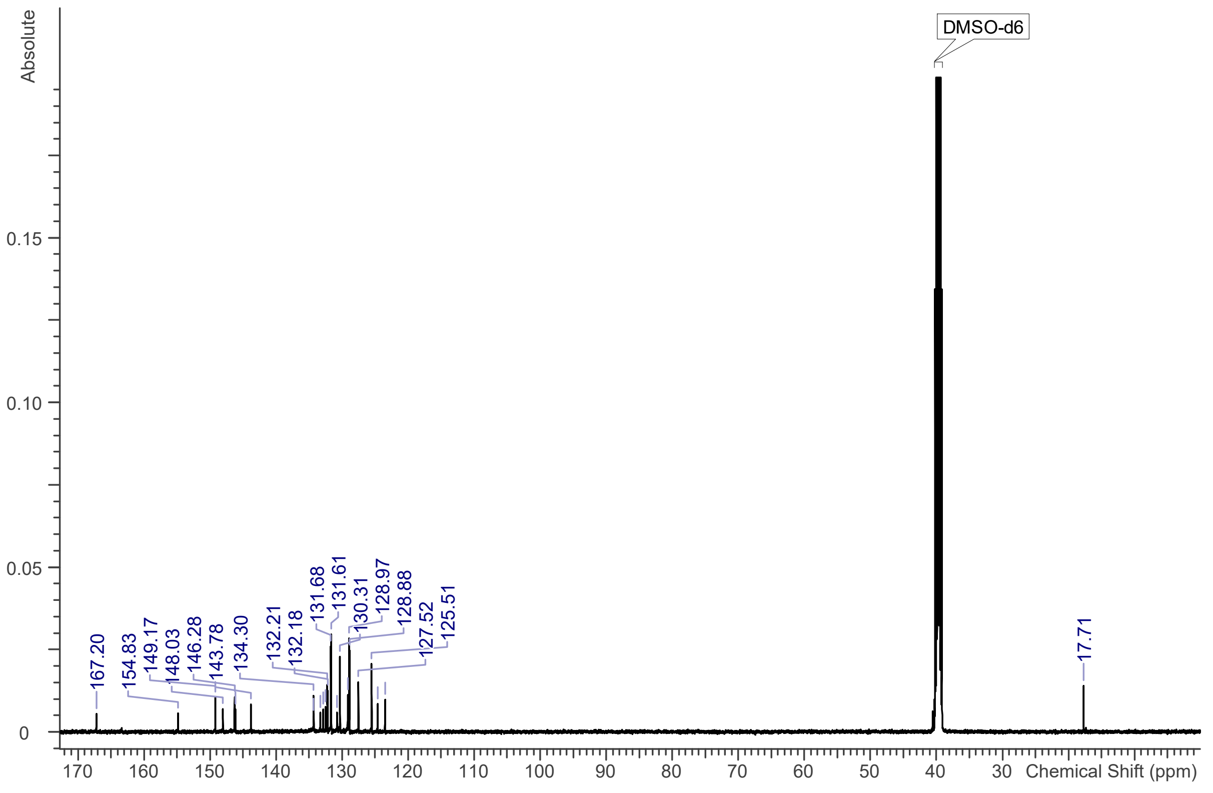

4-(1-(6-(2-chlorophenyl)-4-methylpyridin-3-yl)-1H-1,2,3-triazol-4-yl)benzoic acid (14)

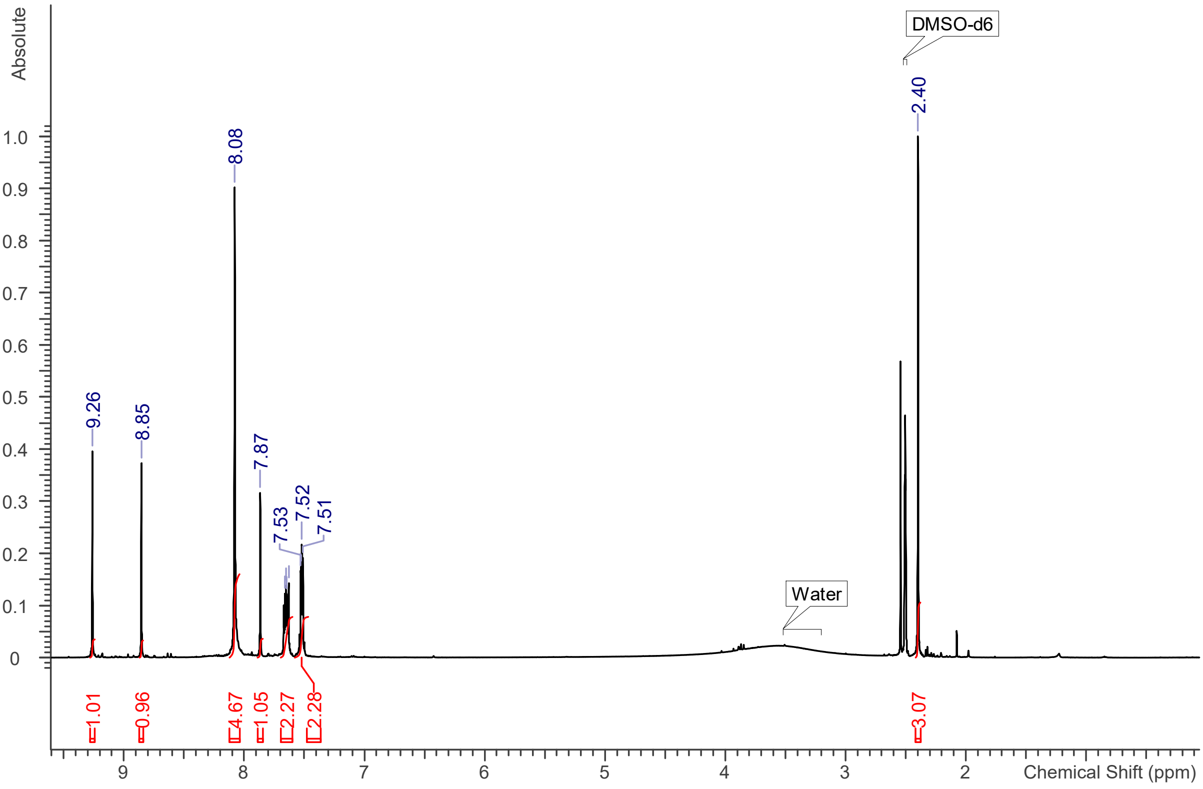

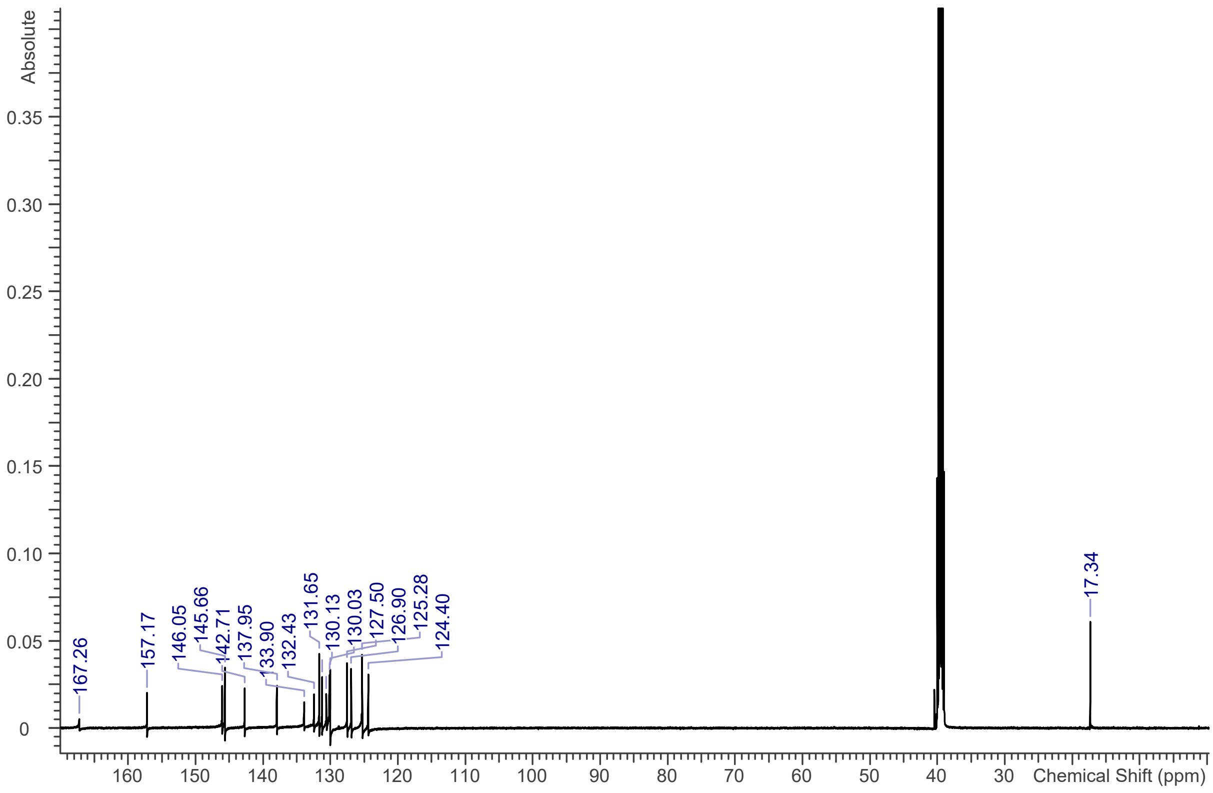

4-(1-(6-(3-chlorophenyl)-4-methylpyridin-3-yl)-1H-1,2,3-triazol-4-yl)benzoic acid (15)

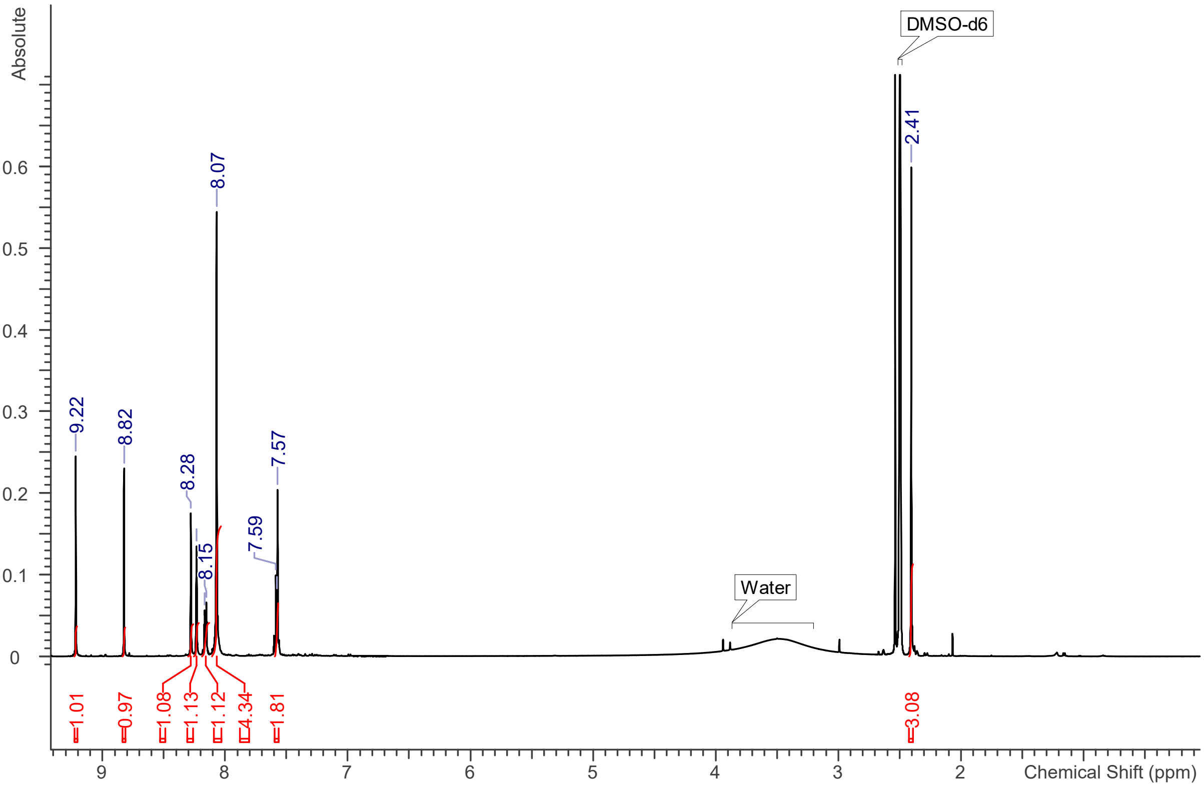

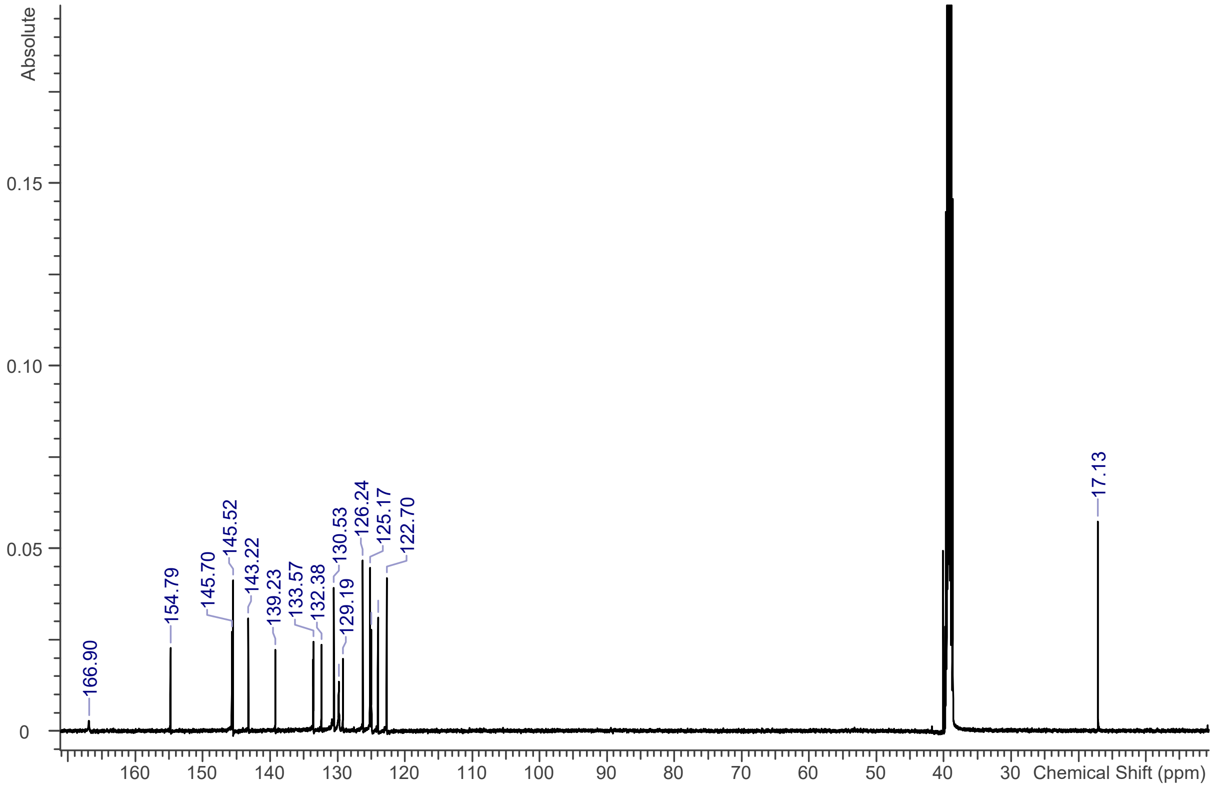

4-(1-(6-(2-methoxyphenyl)-4-methylpyridin-3-yl)-1H-1,2,3-triazol-4-yl)benzoic acid (17)

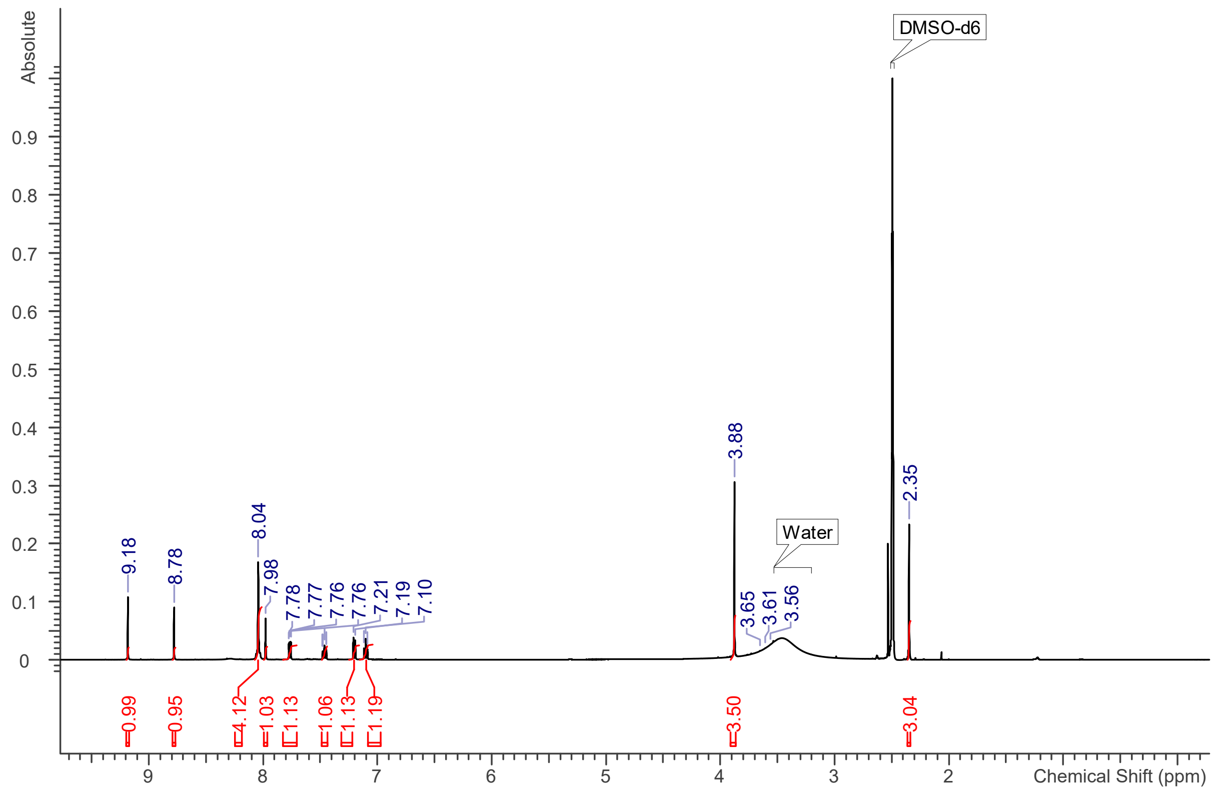

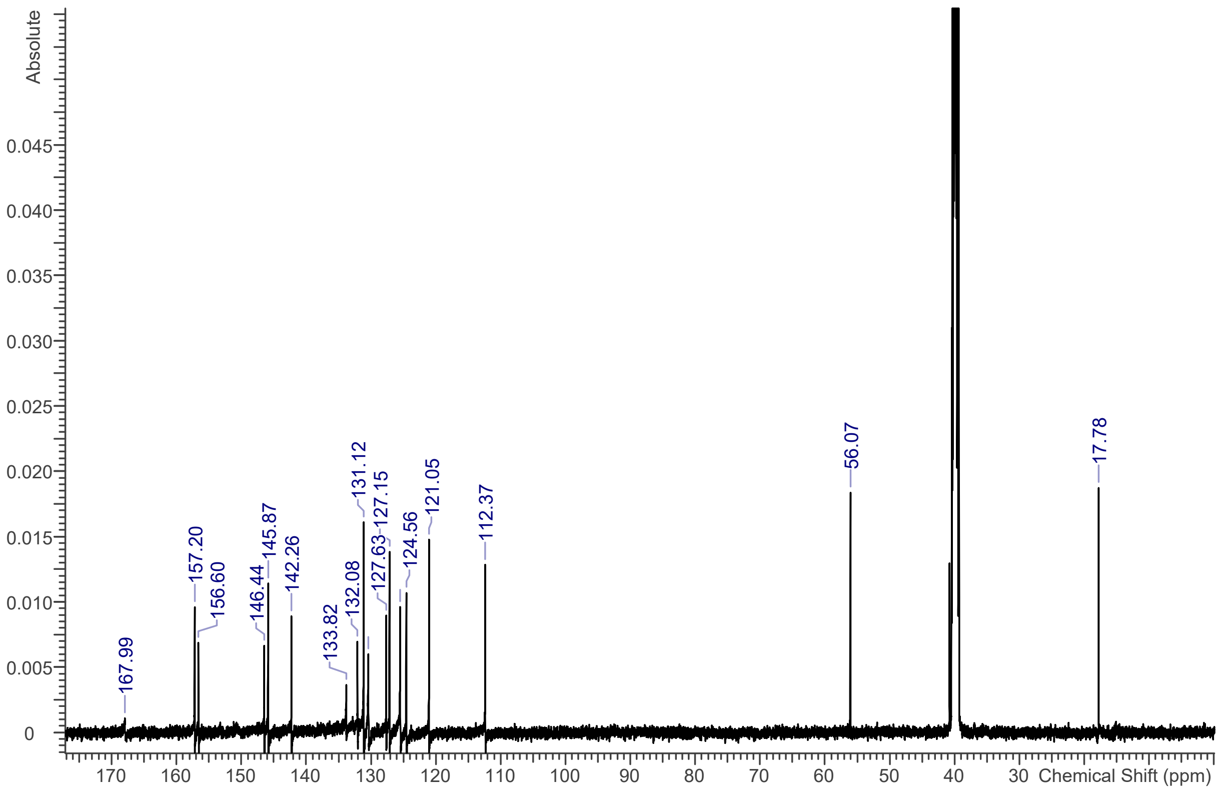

4-(1-(6-(3-methoxyphenyl)-4-methylpyridin-3-yl)-1H-1,2,3-triazol-4-yl)benzoic acid (18)

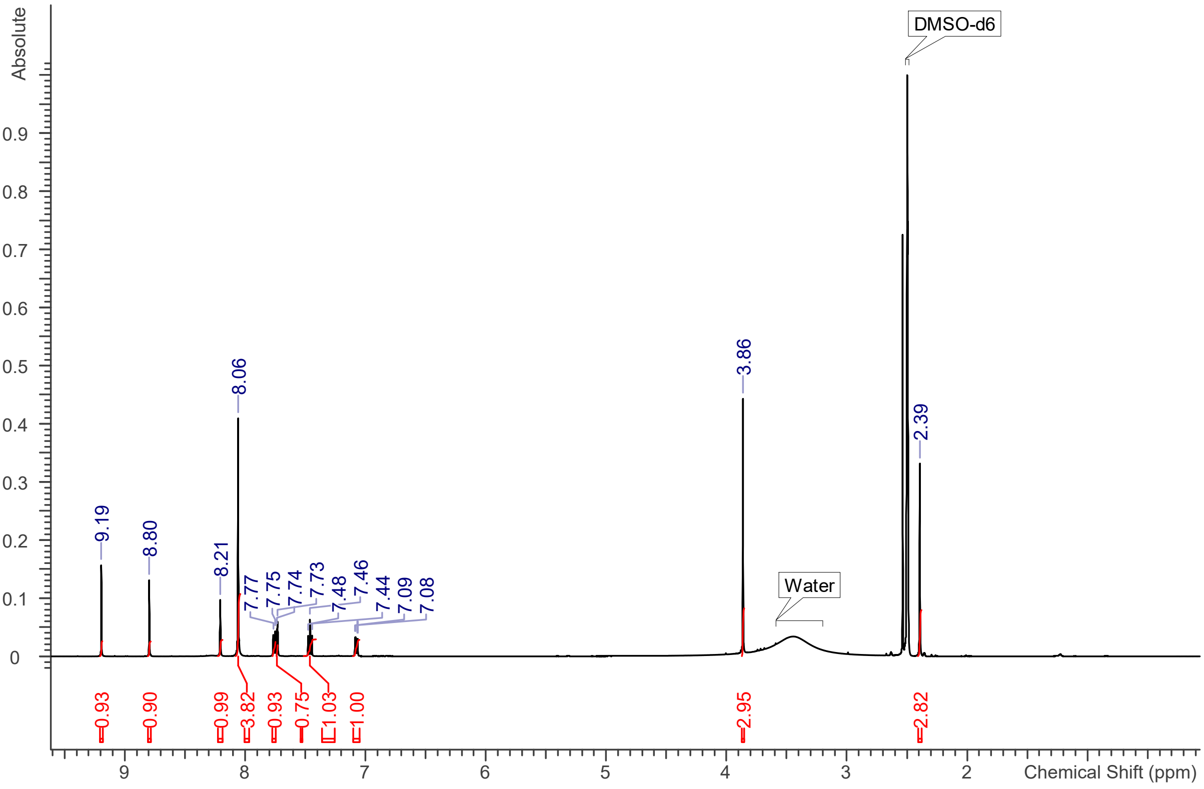

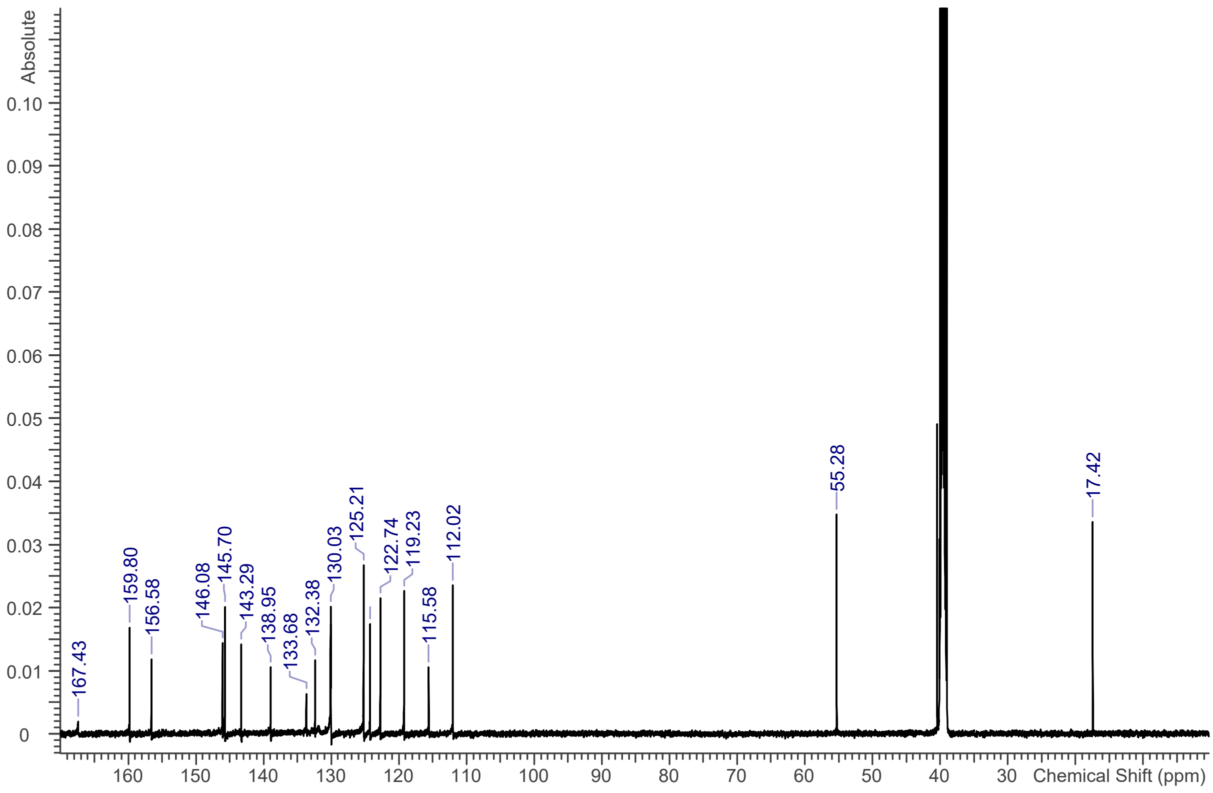

4-(1-(6-(4-methoxyphenyl)-4-methylpyridin-3-yl)-1H-1,2,3-triazol-4-yl)benzoic acid (19)

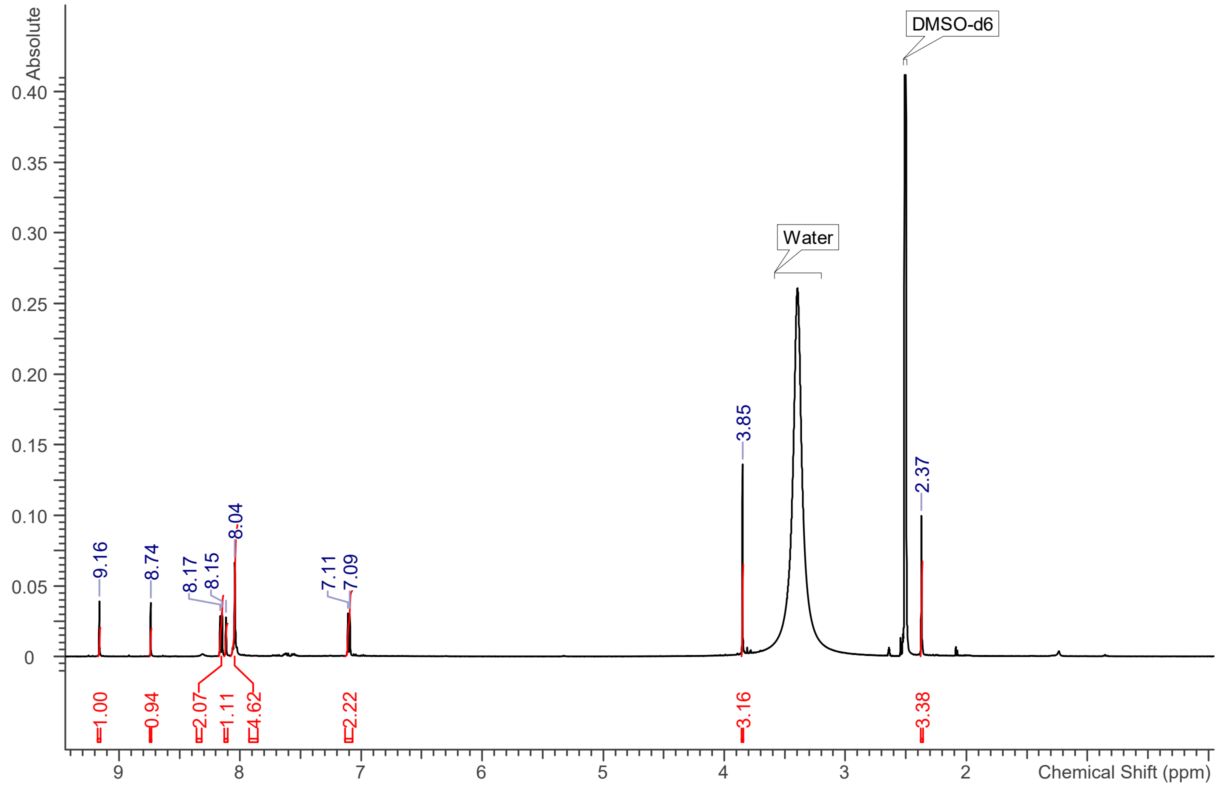

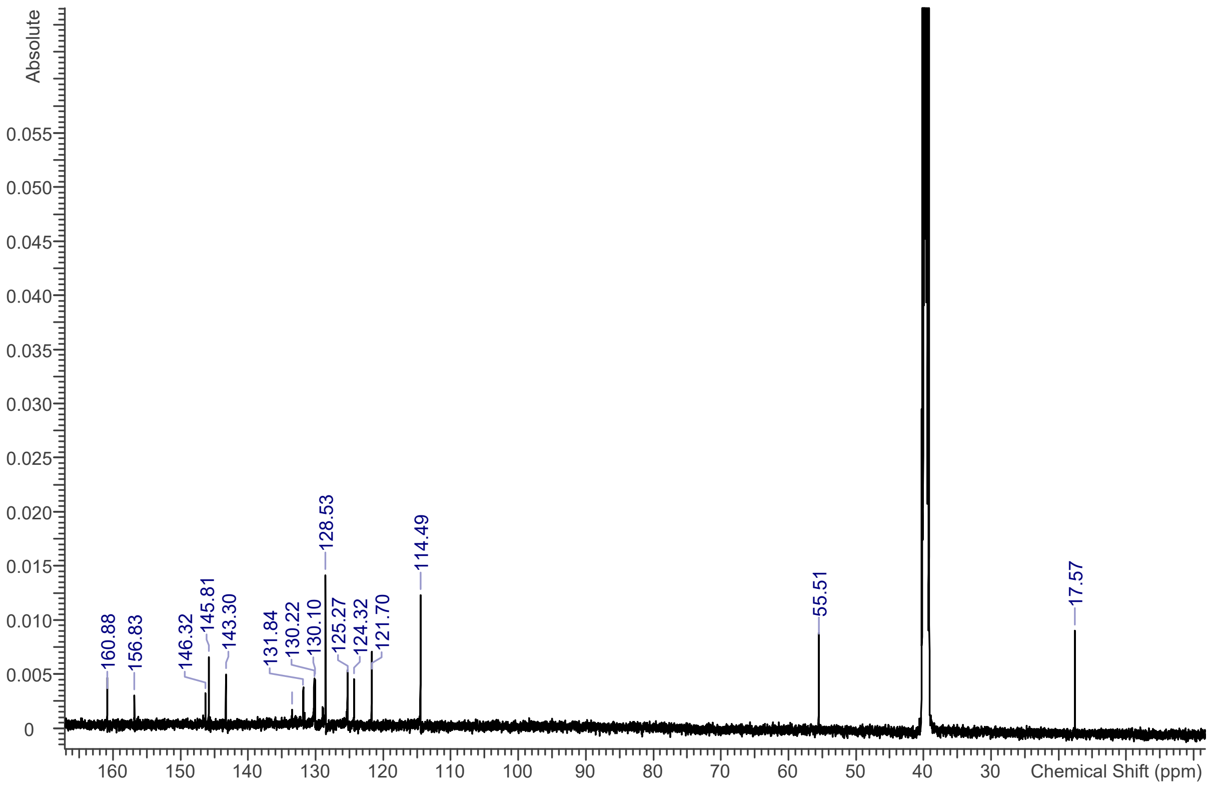

4-(1-(6-(2-chloro-4-methoxyphenyl)-4-methylpyridin-3-yl)-1H-1,2,3-triazol-4-yl)benzoic acid (20)

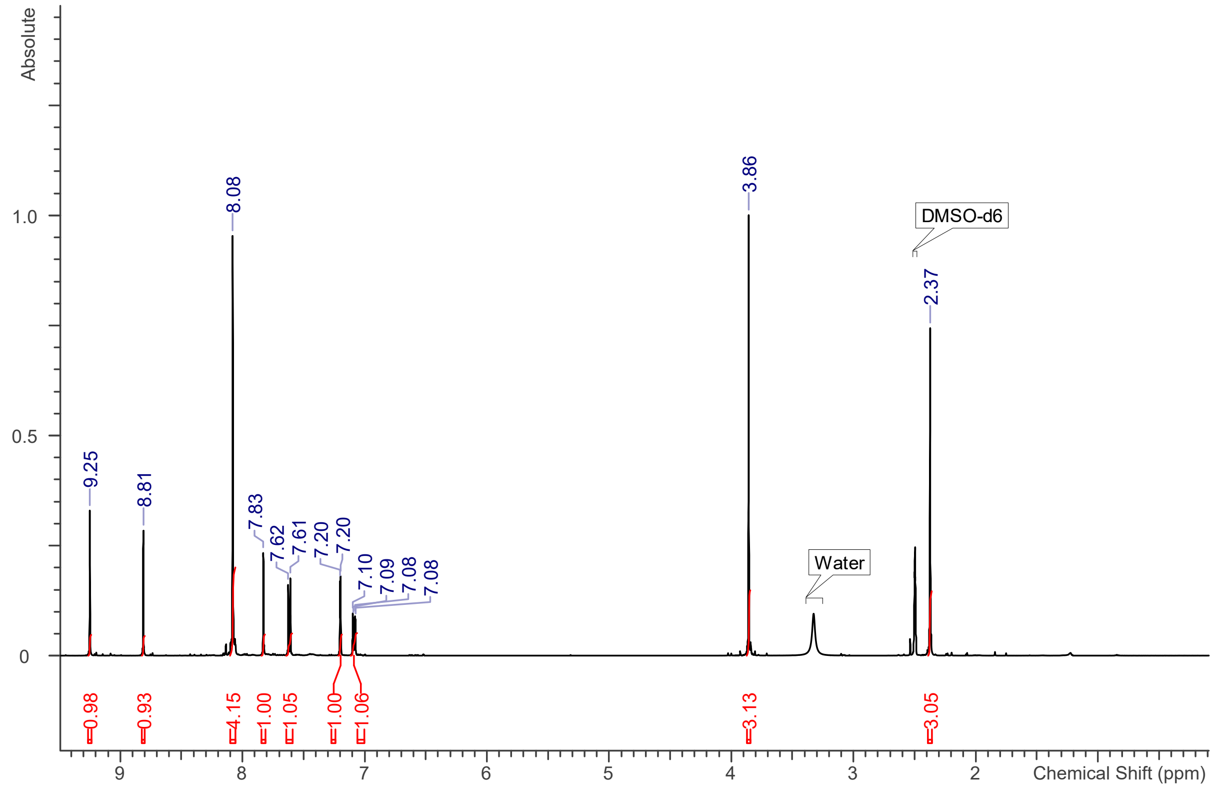

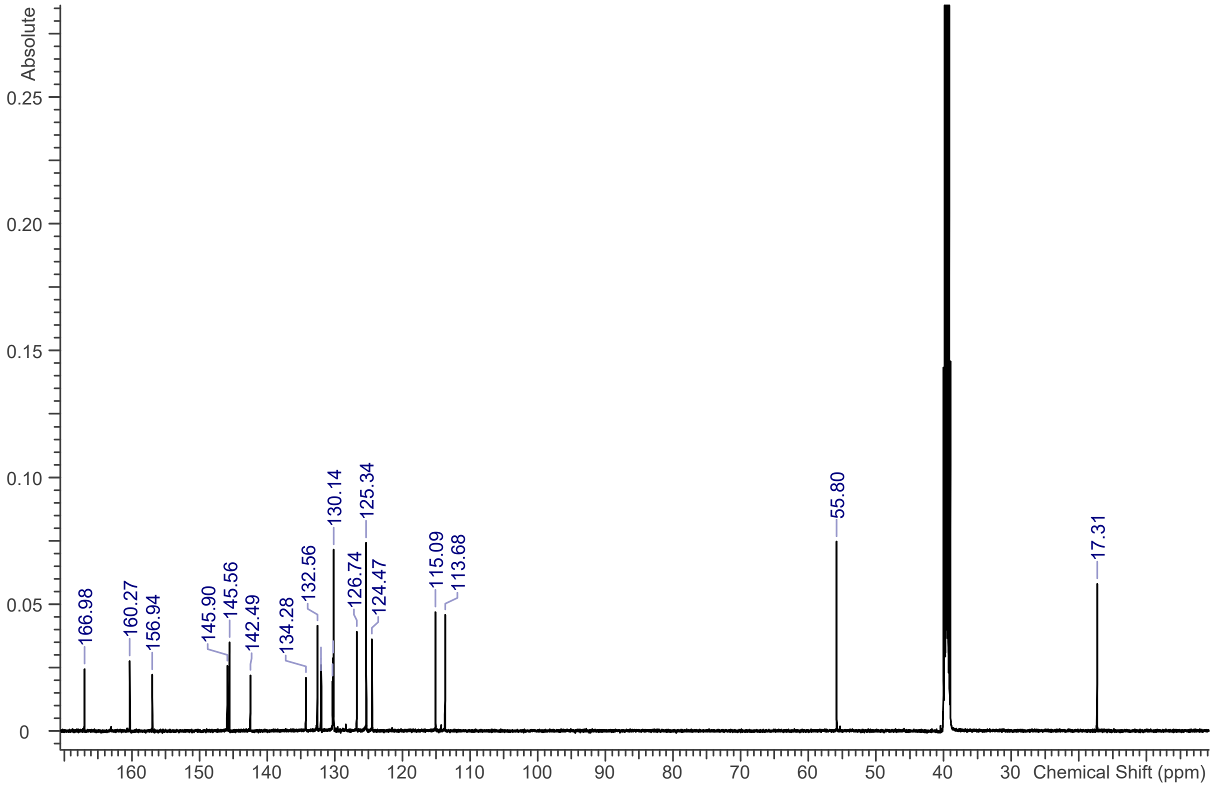

4-(1-(6-(3-hydroxyphenyl)-4-methylpyridin-3-yl)-1H-1,2,3-triazol-4-yl)benzoic acid (22)

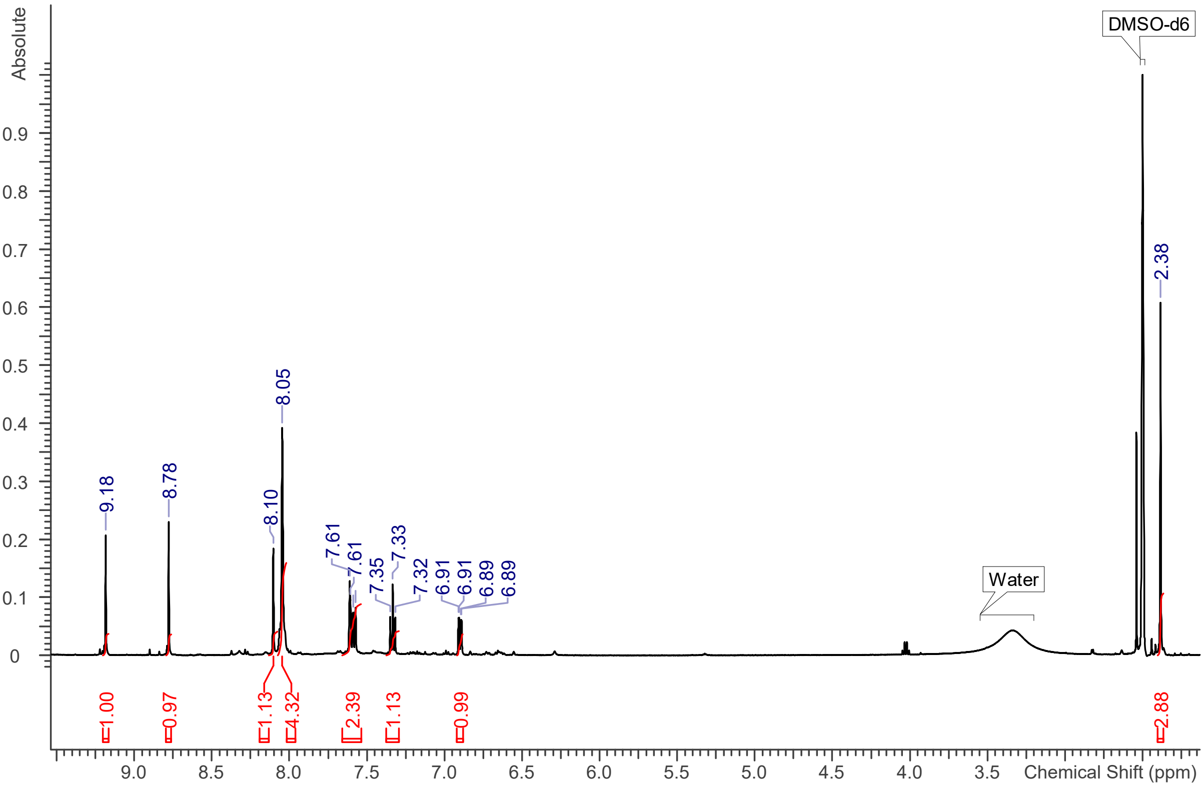

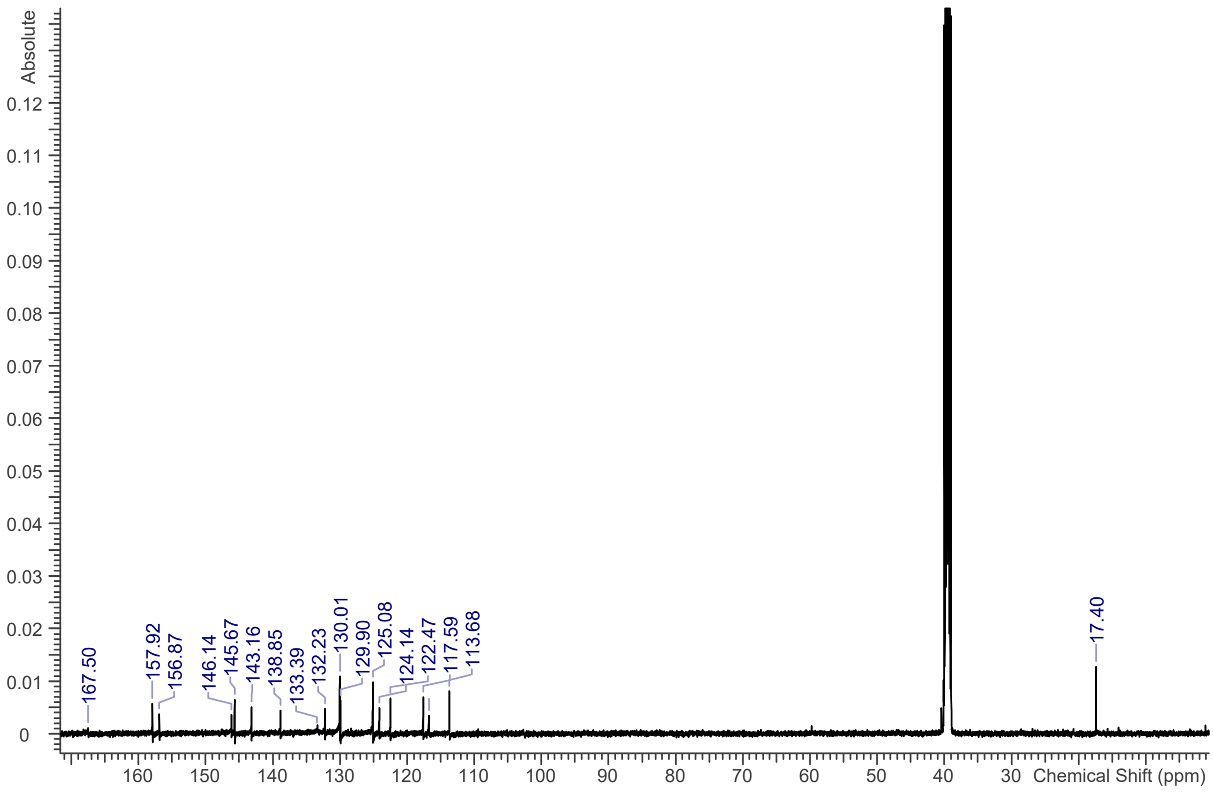

4-(1-(6-(4-hydroxyphenyl)-4-methylpyridin-3-yl)-1H-1,2,3-triazol-4-yl)benzoic acid (23)

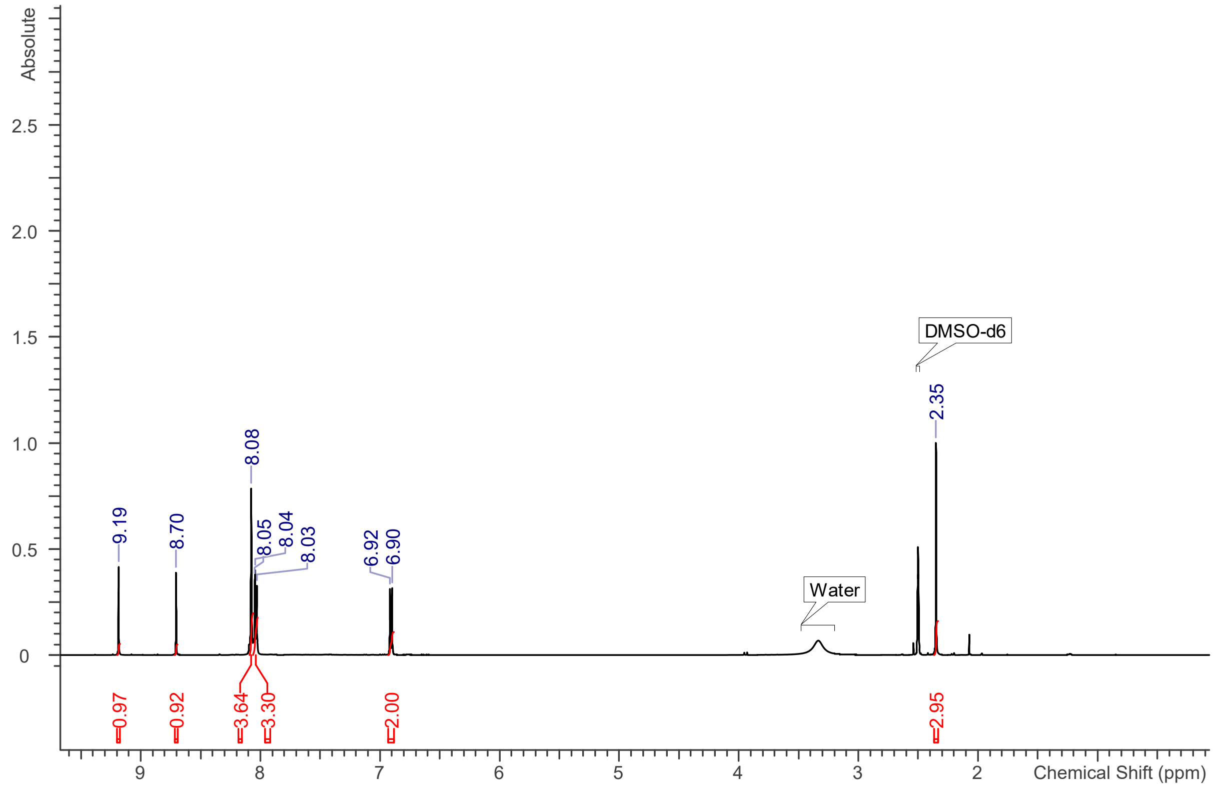

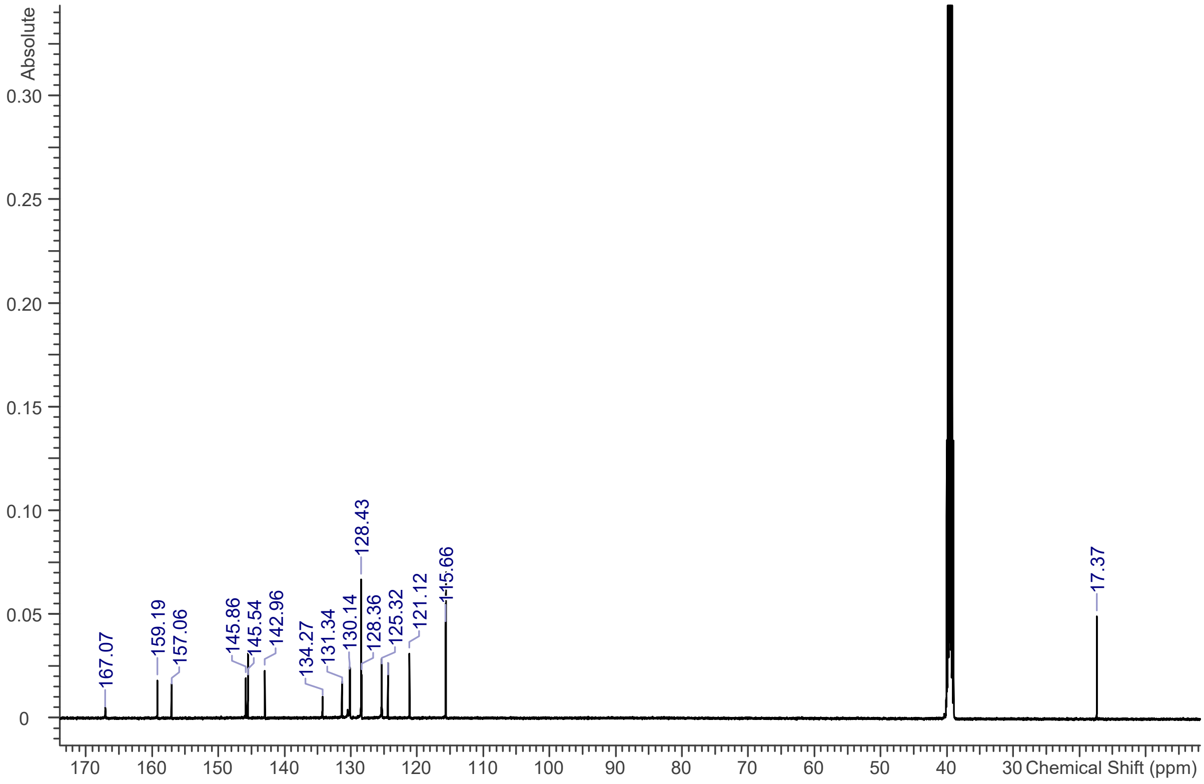

4-(1-(4-methyl-6-(3-(methylcarbamoyl)phenyl)pyridin-3-yl)-1H-1,2,3-triazol-4-yl)benzoic acid (24)

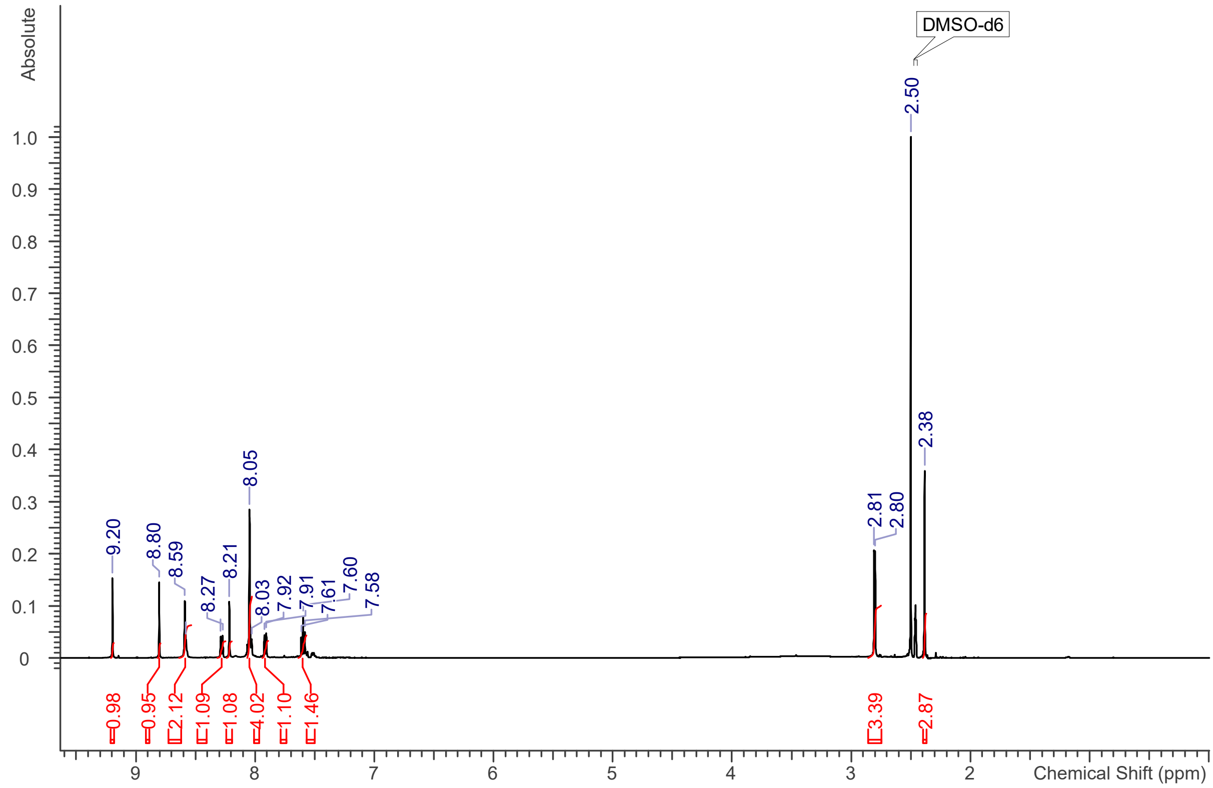

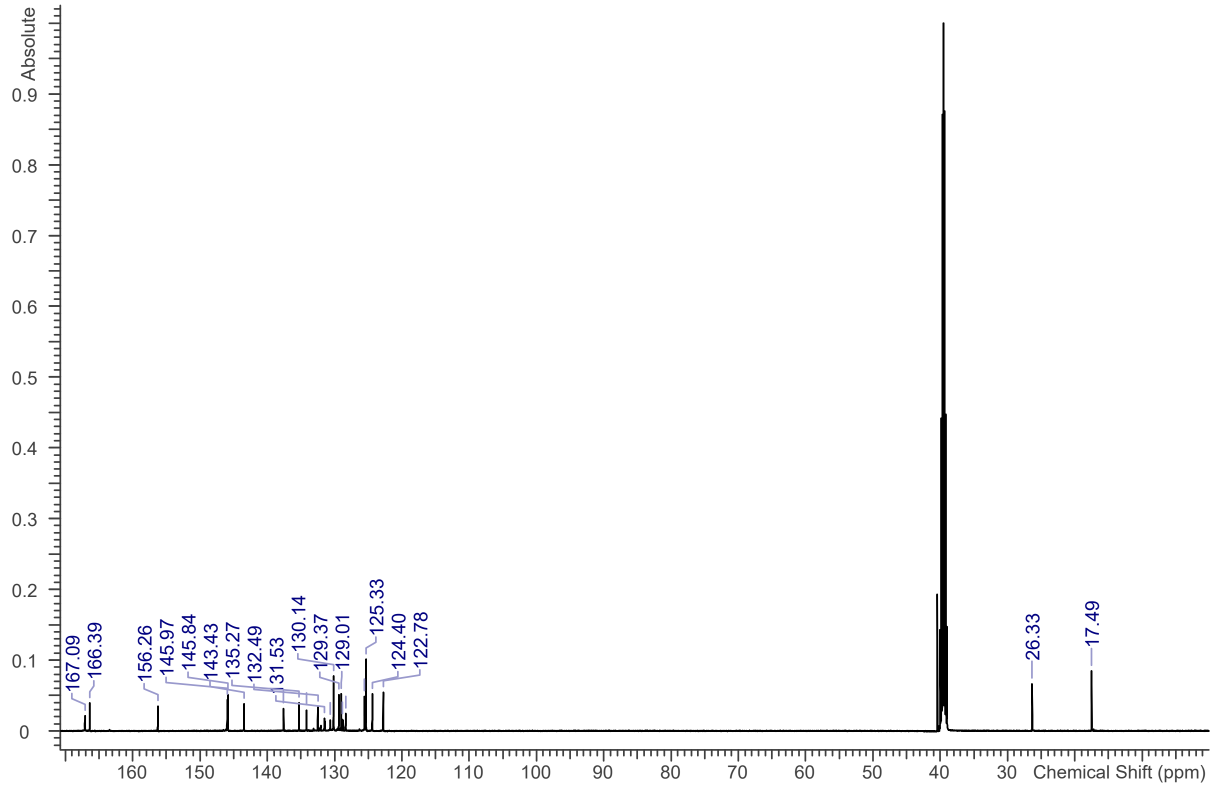

4-(1-(4-methyl-6-(4-(methylcarbamoyl)phenyl)pyridin-3-yl)-1H-1,2,3-triazol-4-yl)benzoic acid (25)

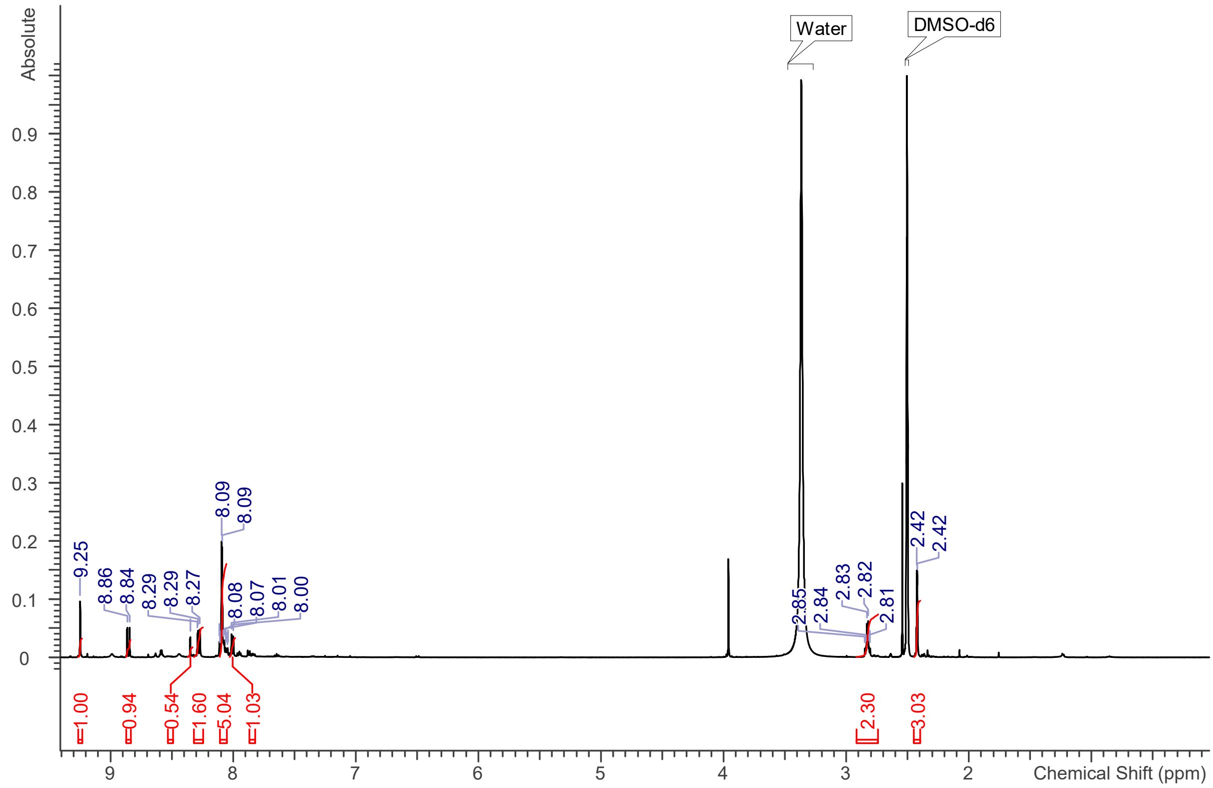

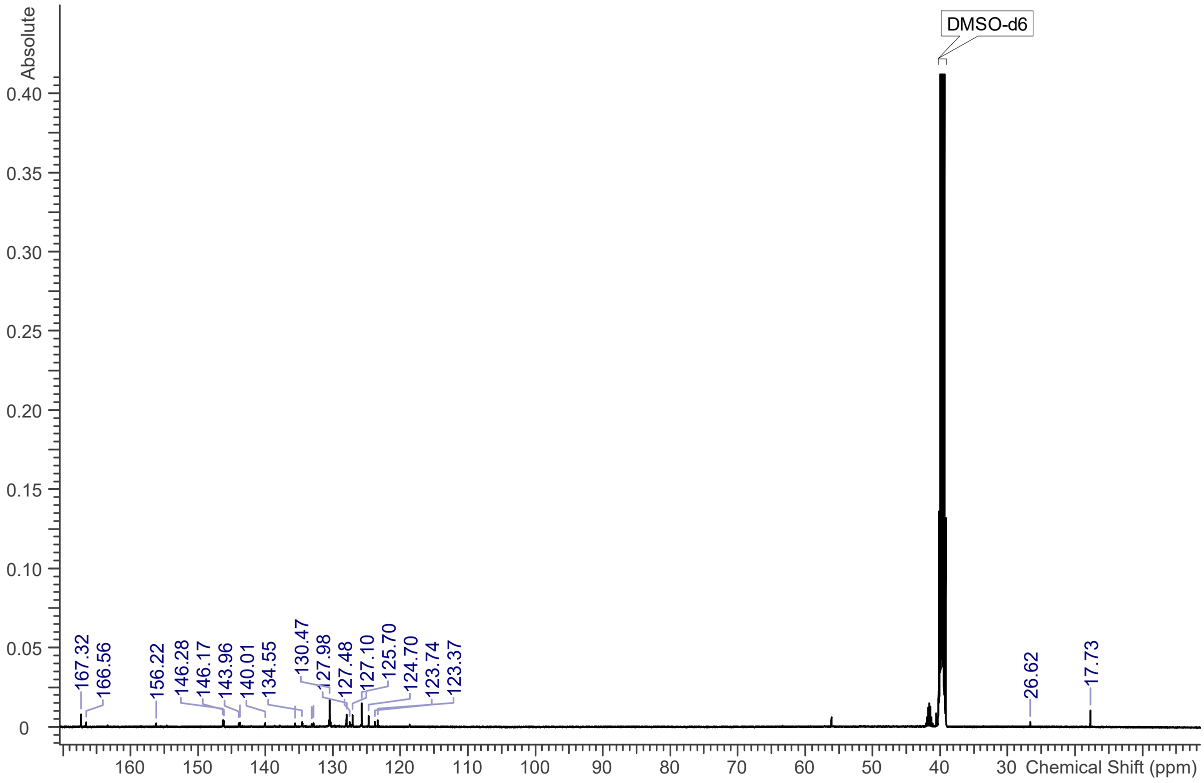

3-(5-(4-(4-Carboxyphenyl)-1H-1,2,3-triazol-1-yl)-4-methylpyridin-2-yl)benzoic acid (26)

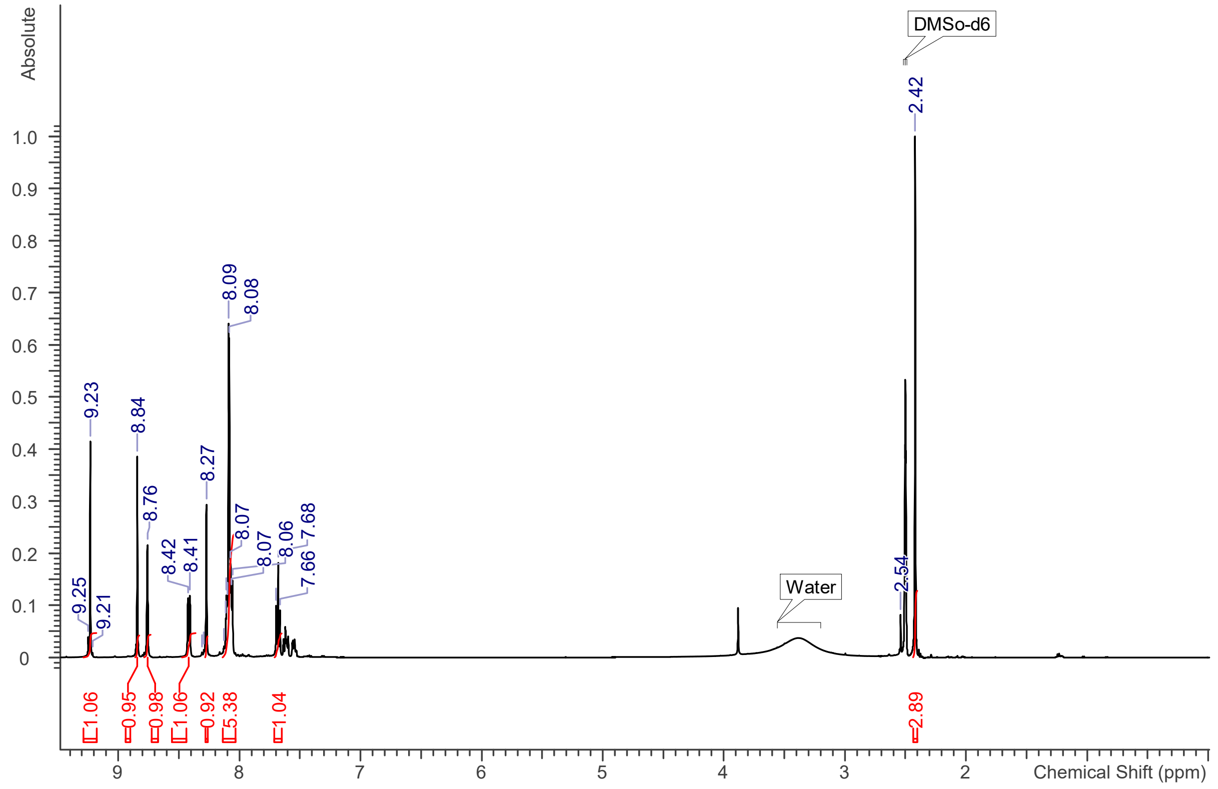

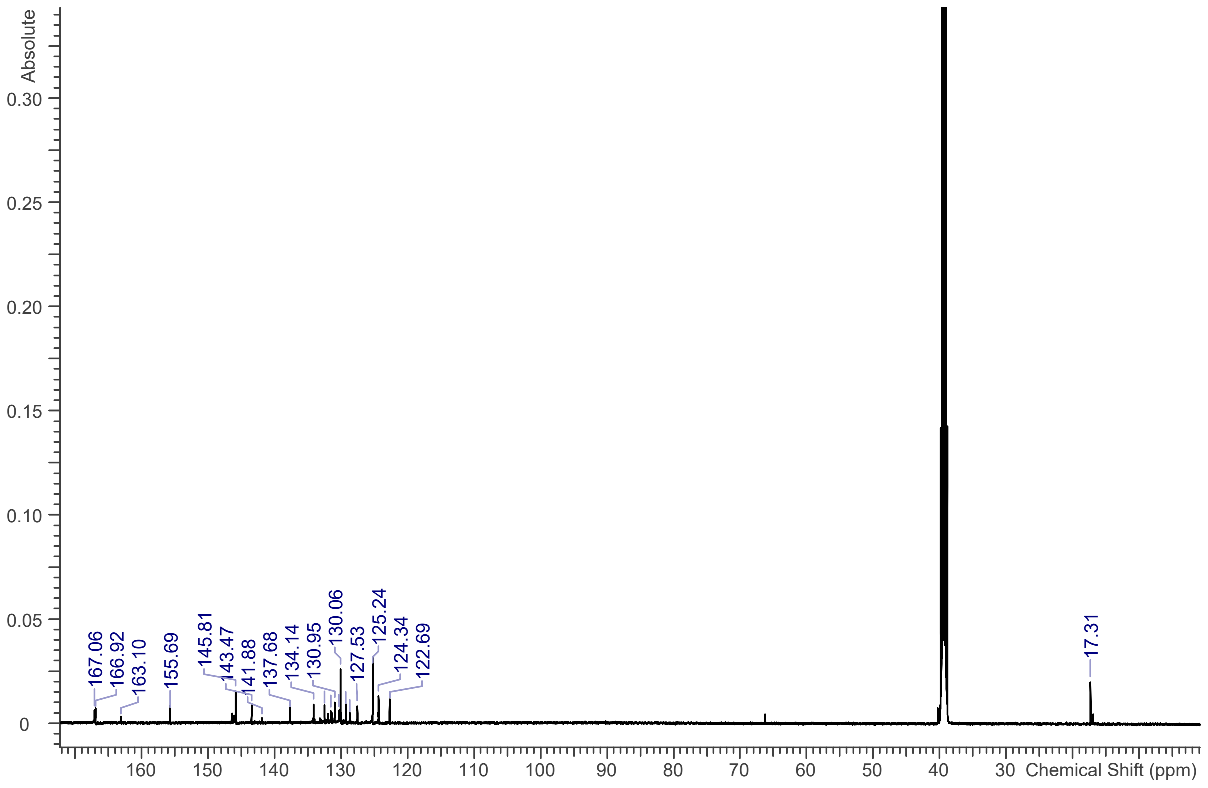

4-(5-(4-(4-Carboxyphenyl)-1H-1,2,3-triazol-1-yl)-4-methylpyridin-2-yl)benzoic acid (27)

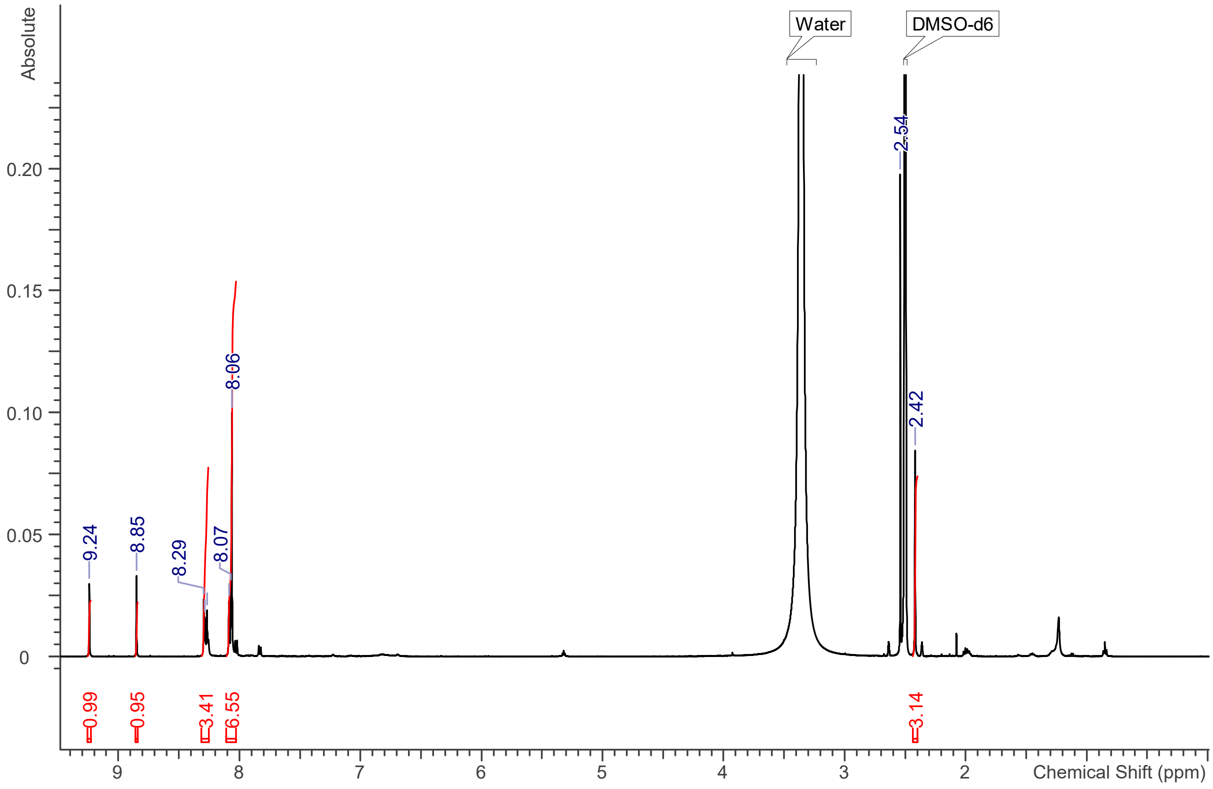

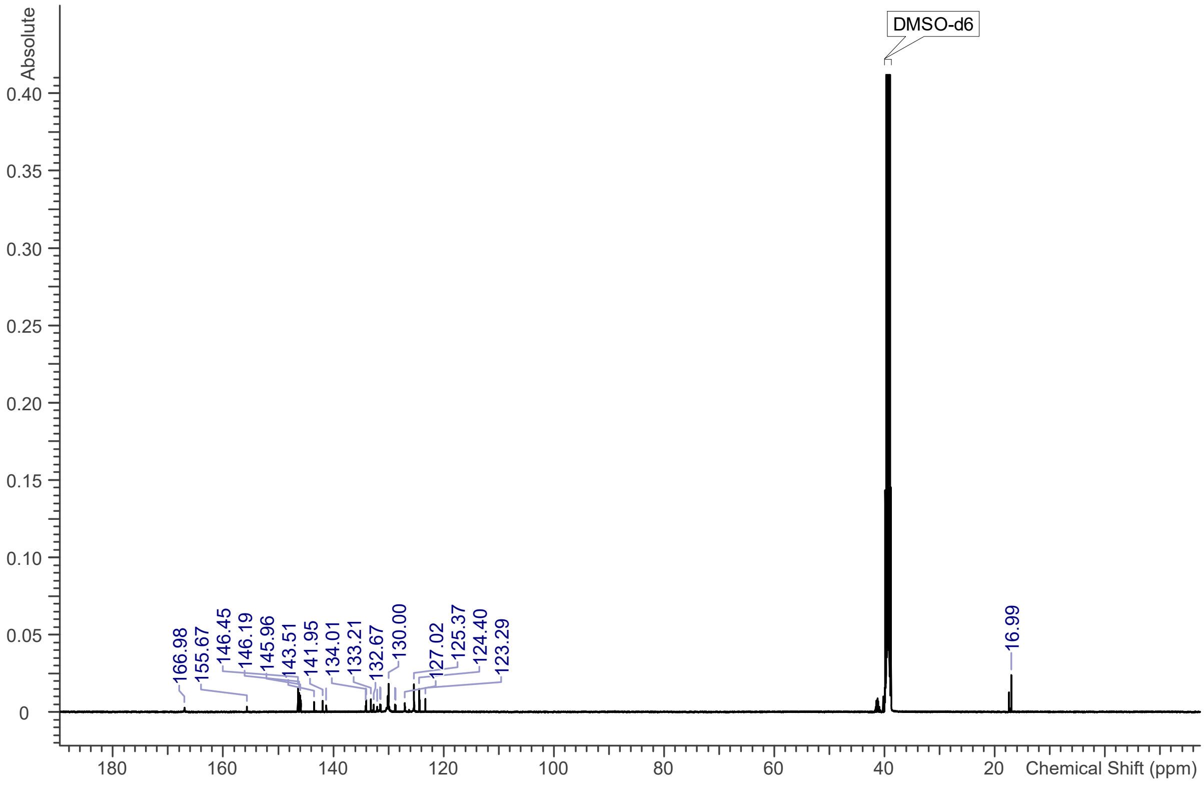

4-(1-(4-methyl-6-(3-phenoxyphenyl)pyridin-3-yl)-1H-1,2,3-triazol-4-yl)benzoic acid (28)

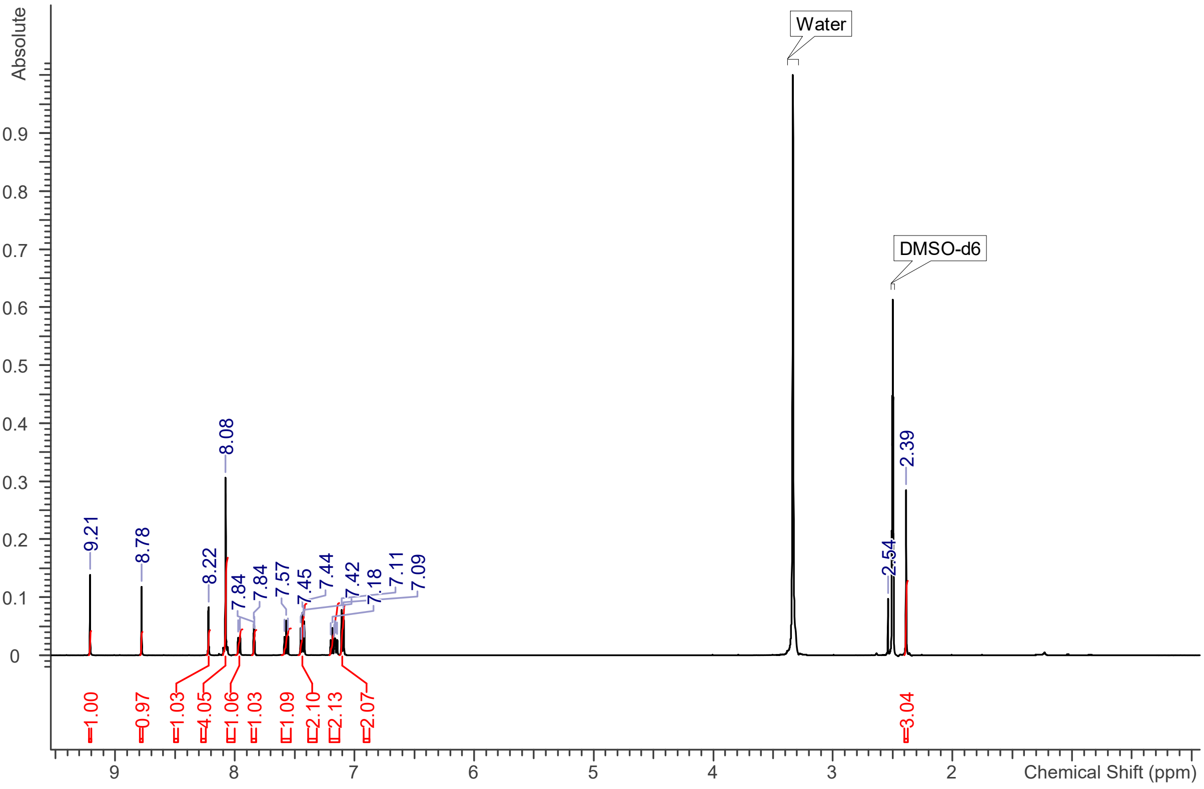

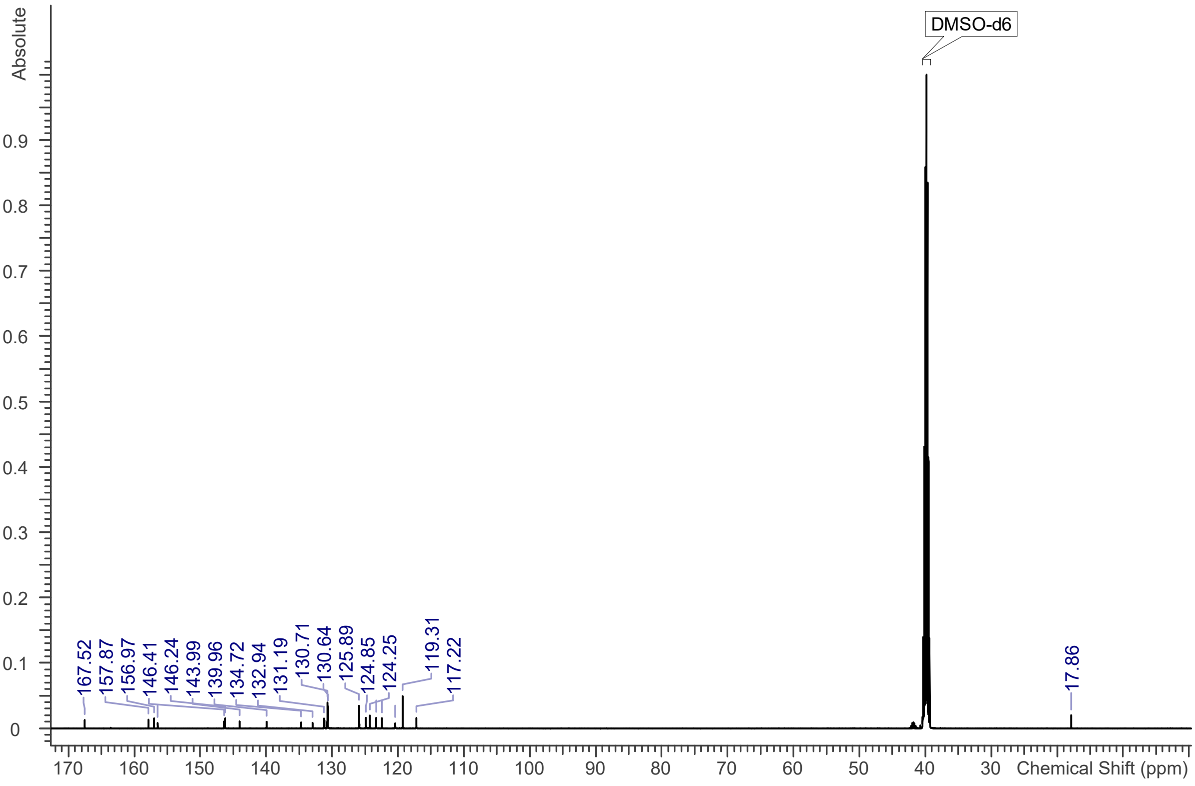

4-(1-(6-(3-(benzyloxy)phenyl)-4-methylpyridin-3-yl)-1H-1,2,3-triazol-4-yl)benzoic acid (29)

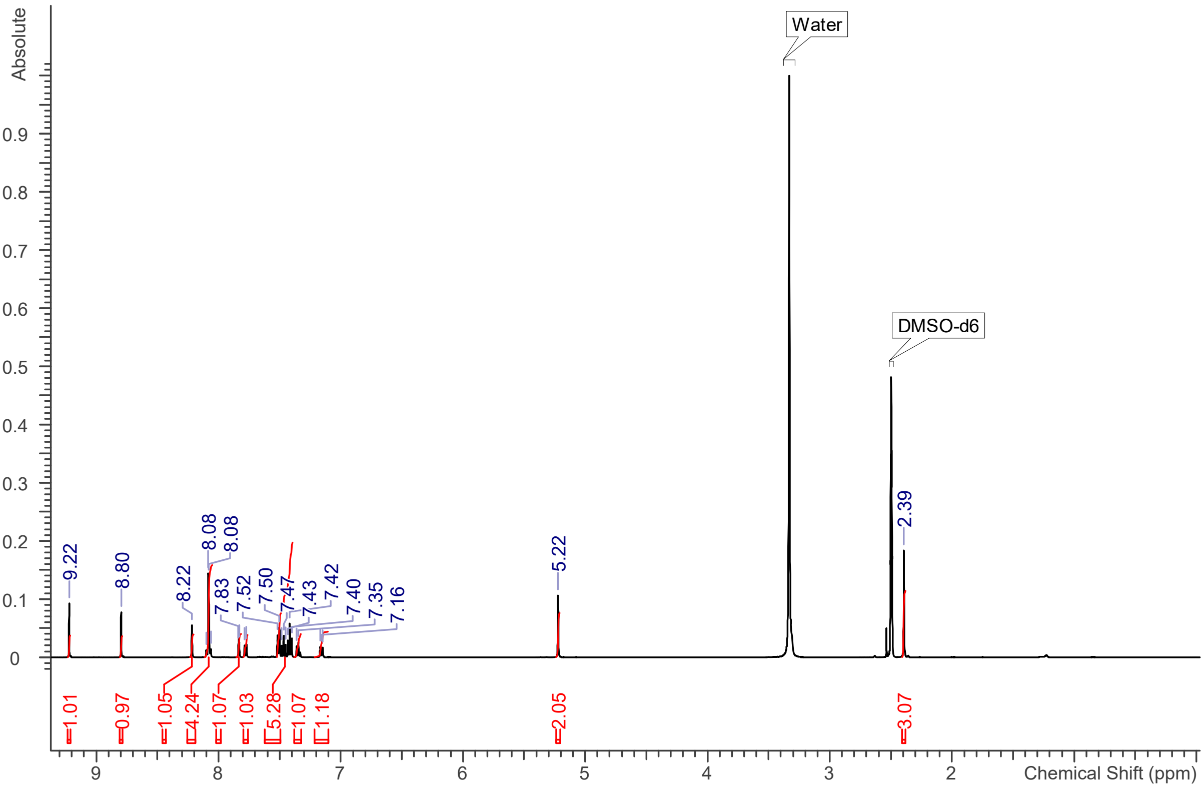

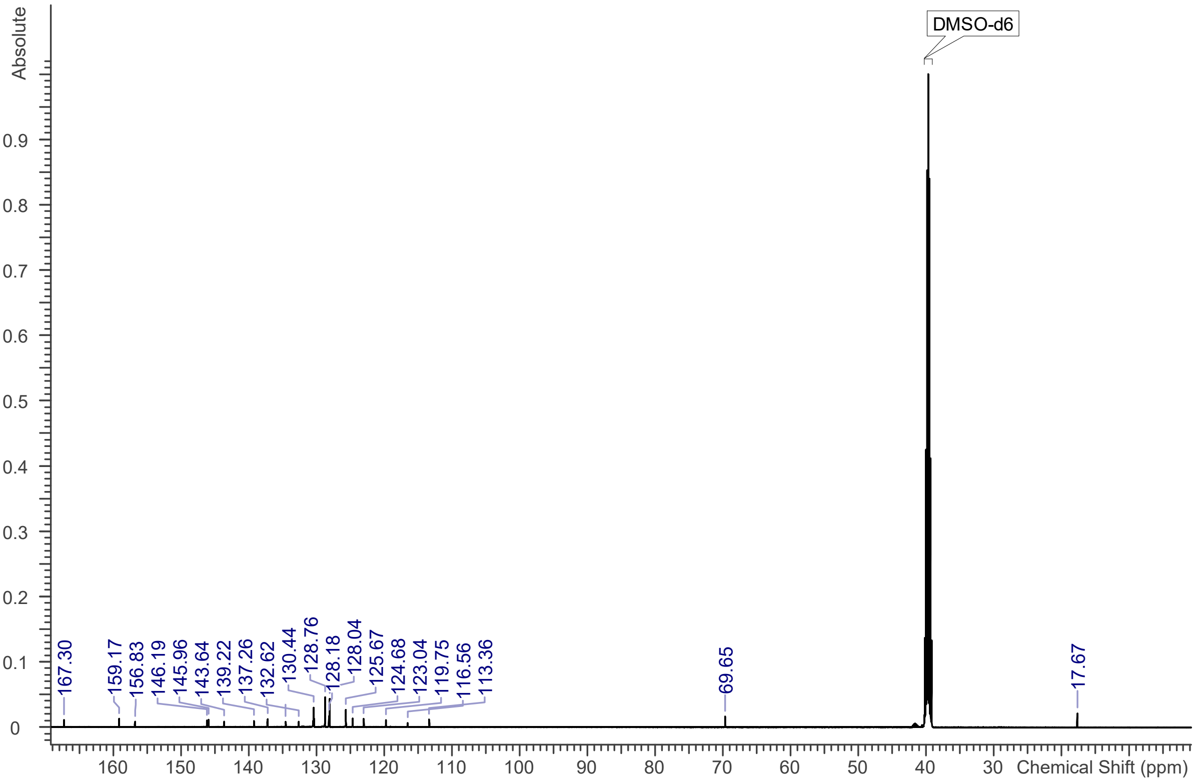

4-(1-(6-(3-((2-chlorobenzyl)oxy)phenyl)-4-methylpyridin-3-yl)-1H-1,2,3-triazol-4-yl) benzoic acid (30)

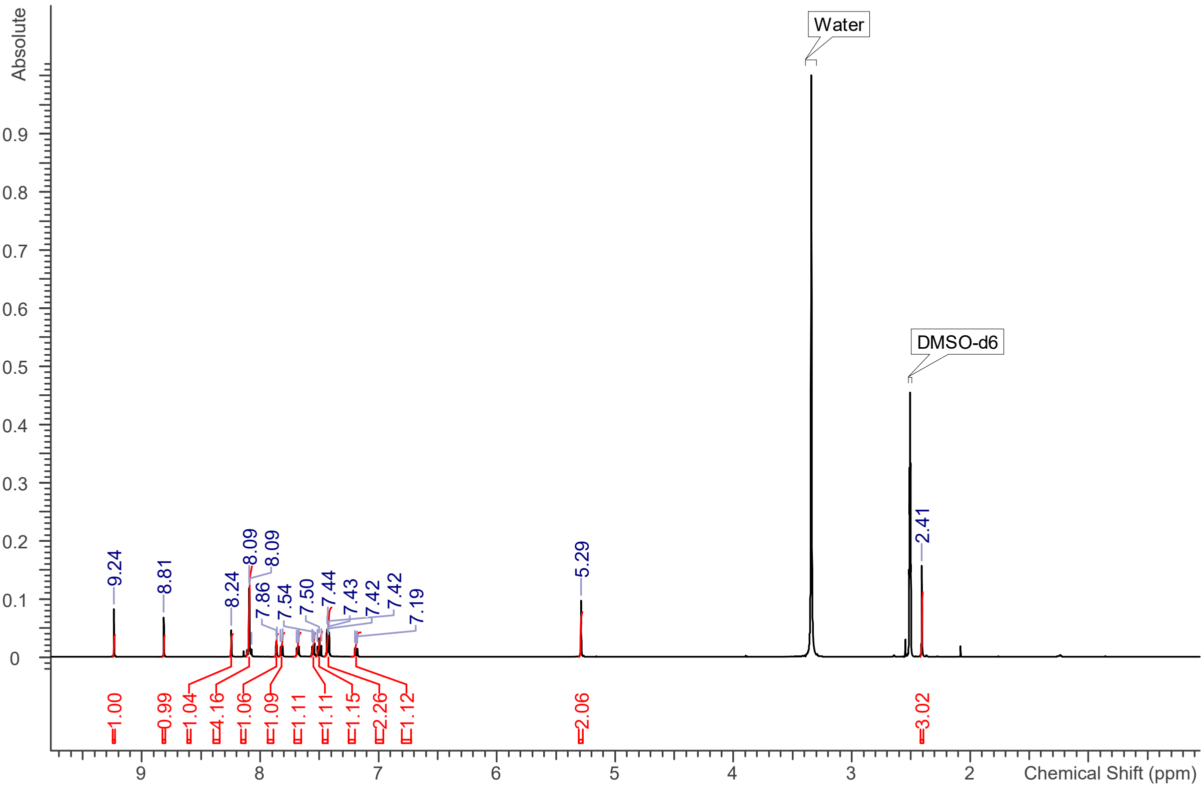

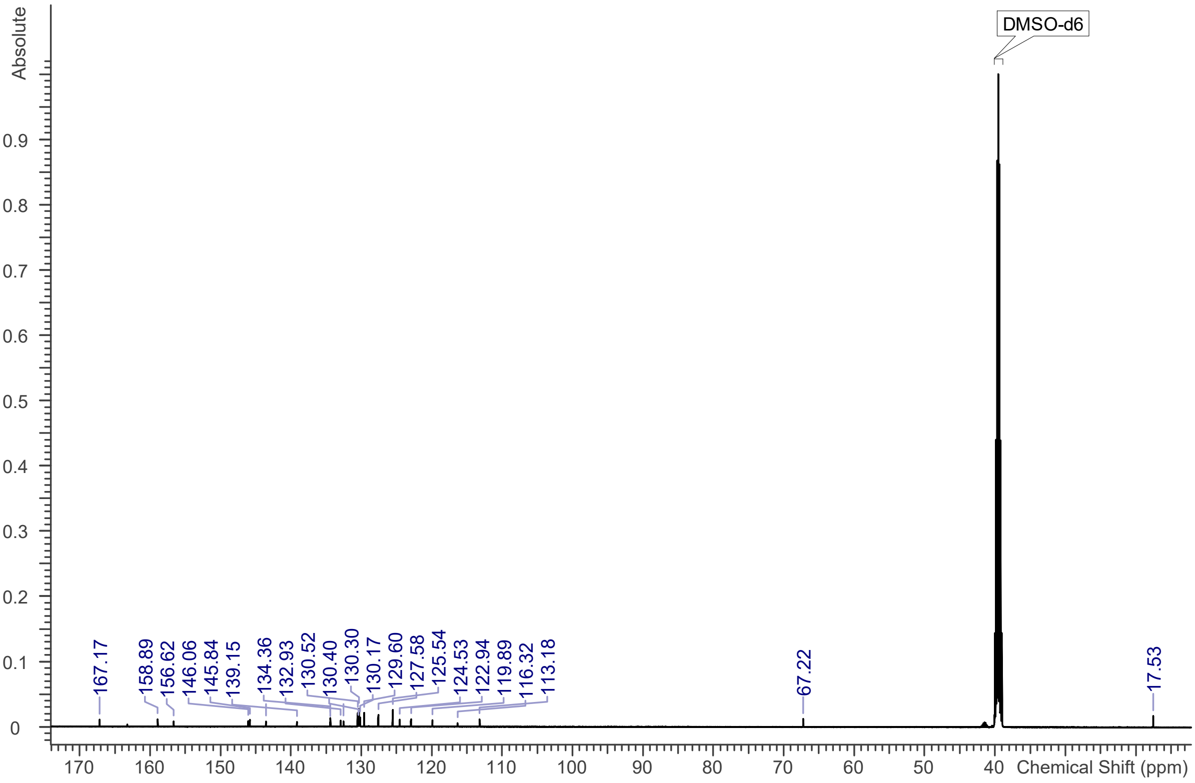

4-(1-(6-(3-((2-methoxybenzyl)oxy)phenyl)-4-methylpyridin-3-yl)-1H-1,2,3-triazol-4-yl) benzoic acid (31)

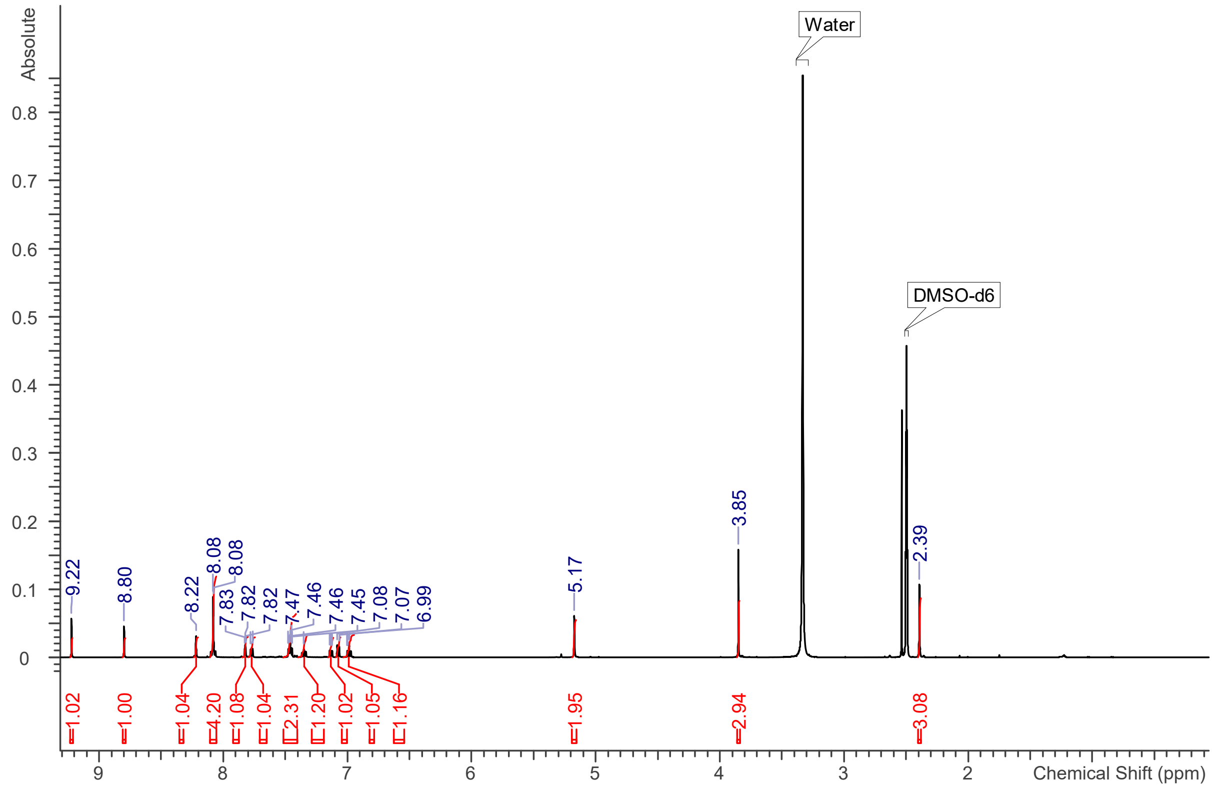

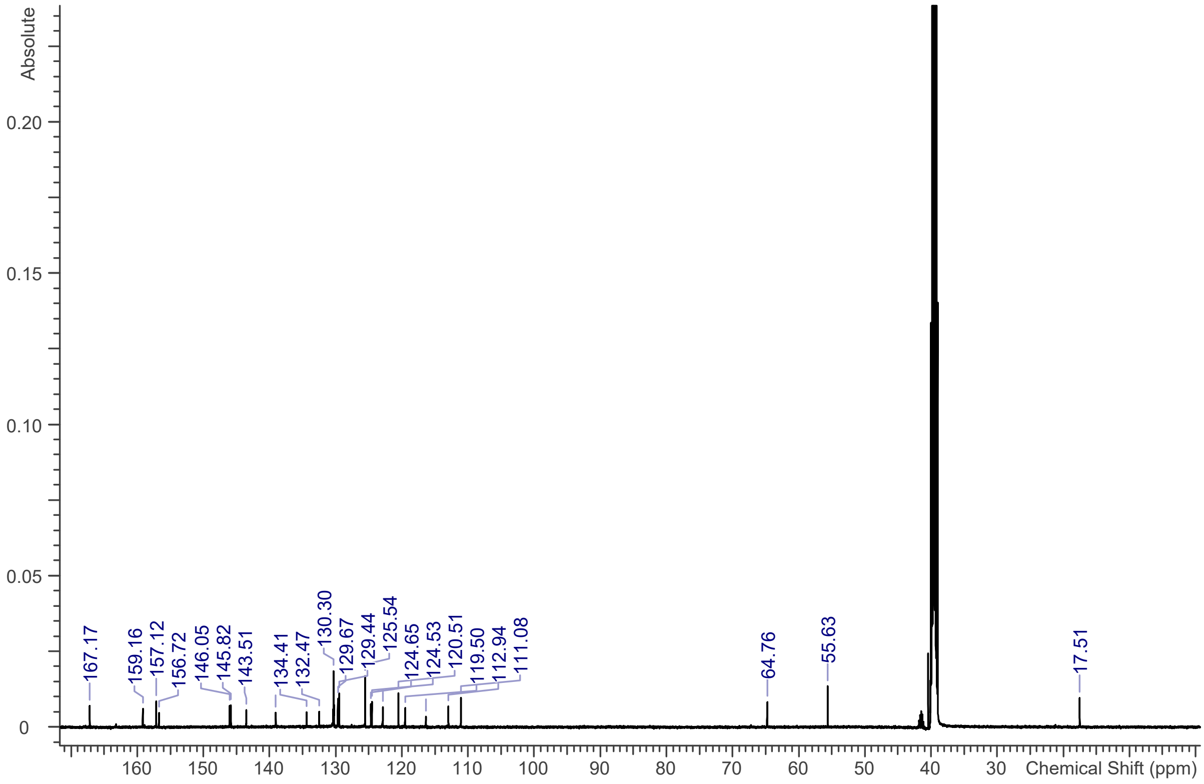

4-(1-(6-(3-((3-methoxybenzyl)oxy)phenyl)-4-methylpyridin-3-yl)-1H-1,2,3-triazol-4-yl) benzoic acid (32)

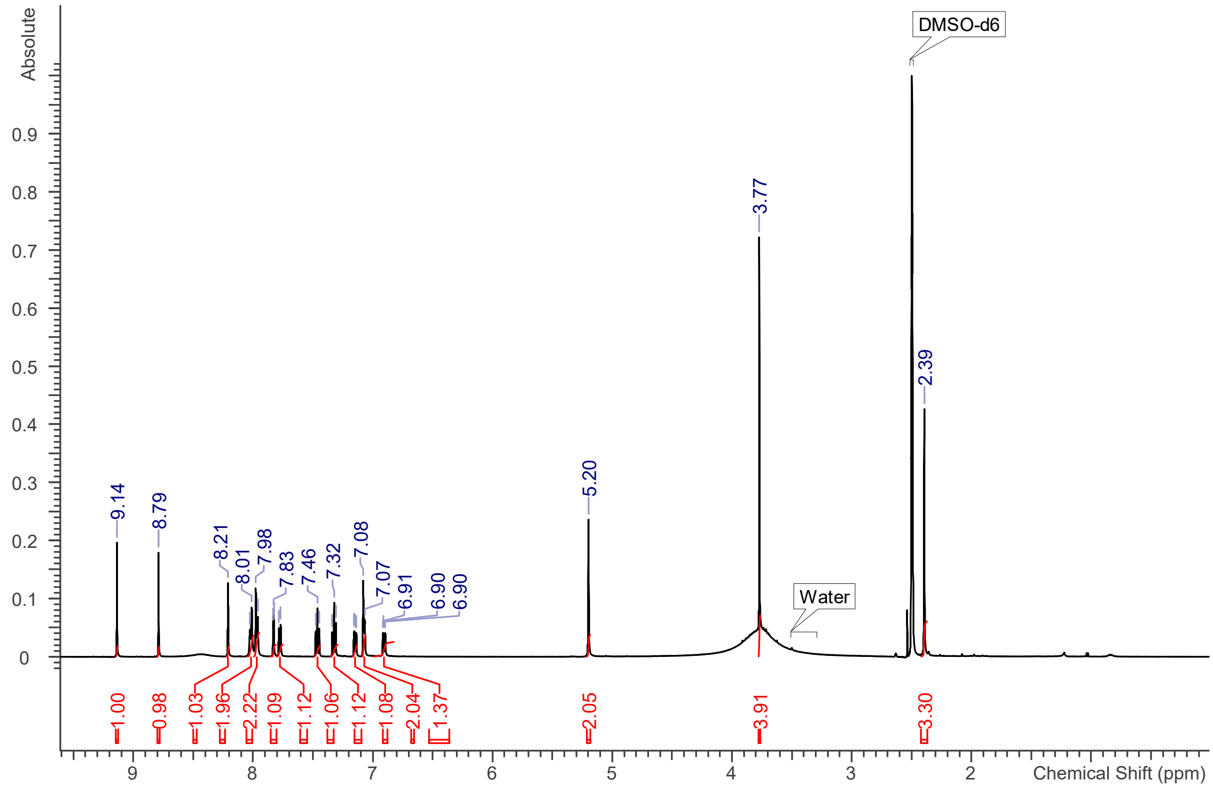

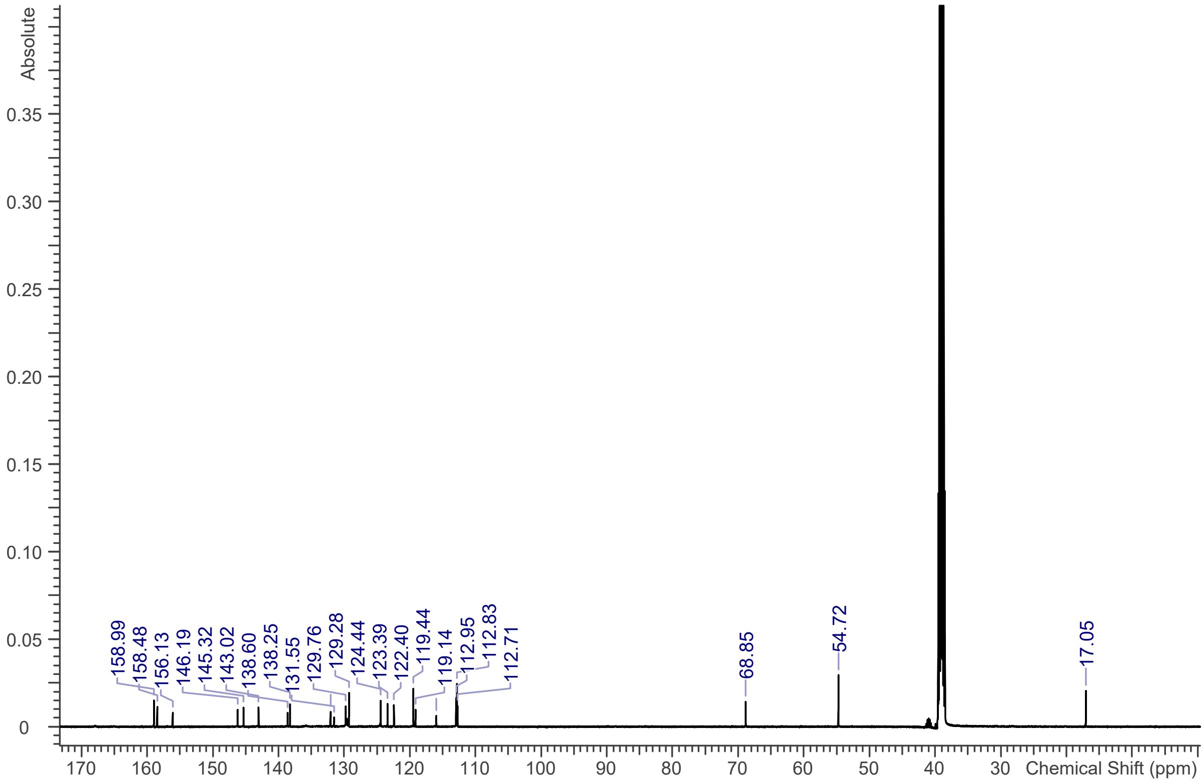

4-(1-(6-(3-((3,5-dimethoxybenzyl)oxy)phenyl)-4-methylpyridin-3-yl)-1H-1,2,3-triazol-4-yl) benzoic acid (33)

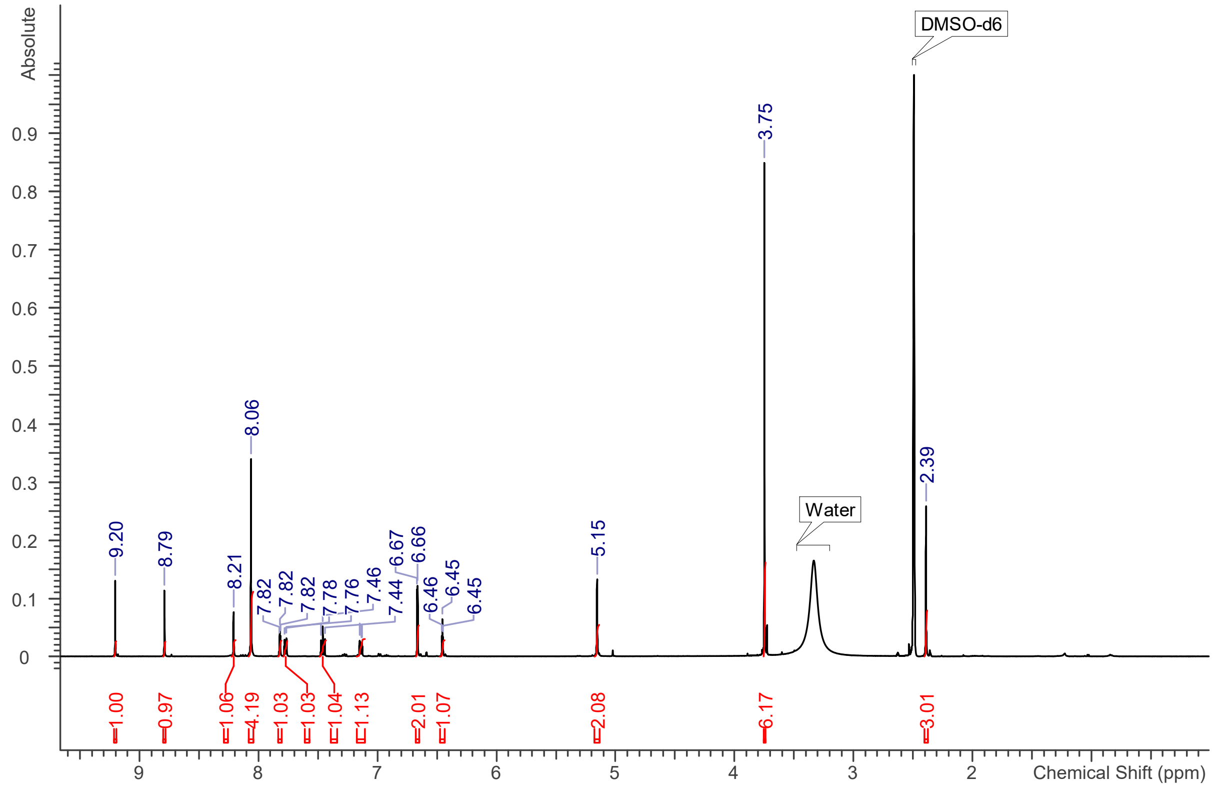

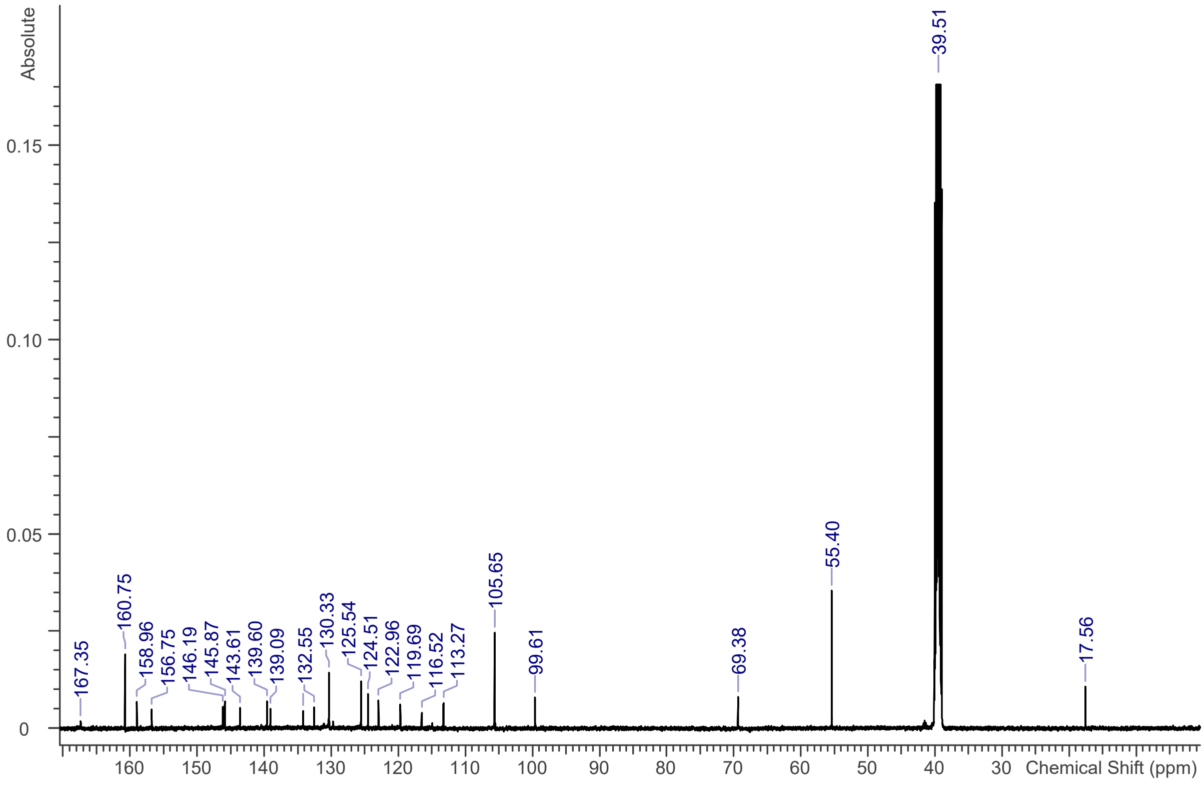

4-(1-(6-(3-((4-methoxybenzyl)oxy)phenyl)-4-methylpyridin-3-yl)-1H-1,2,3-triazol-4-yl) benzoic acid (34)

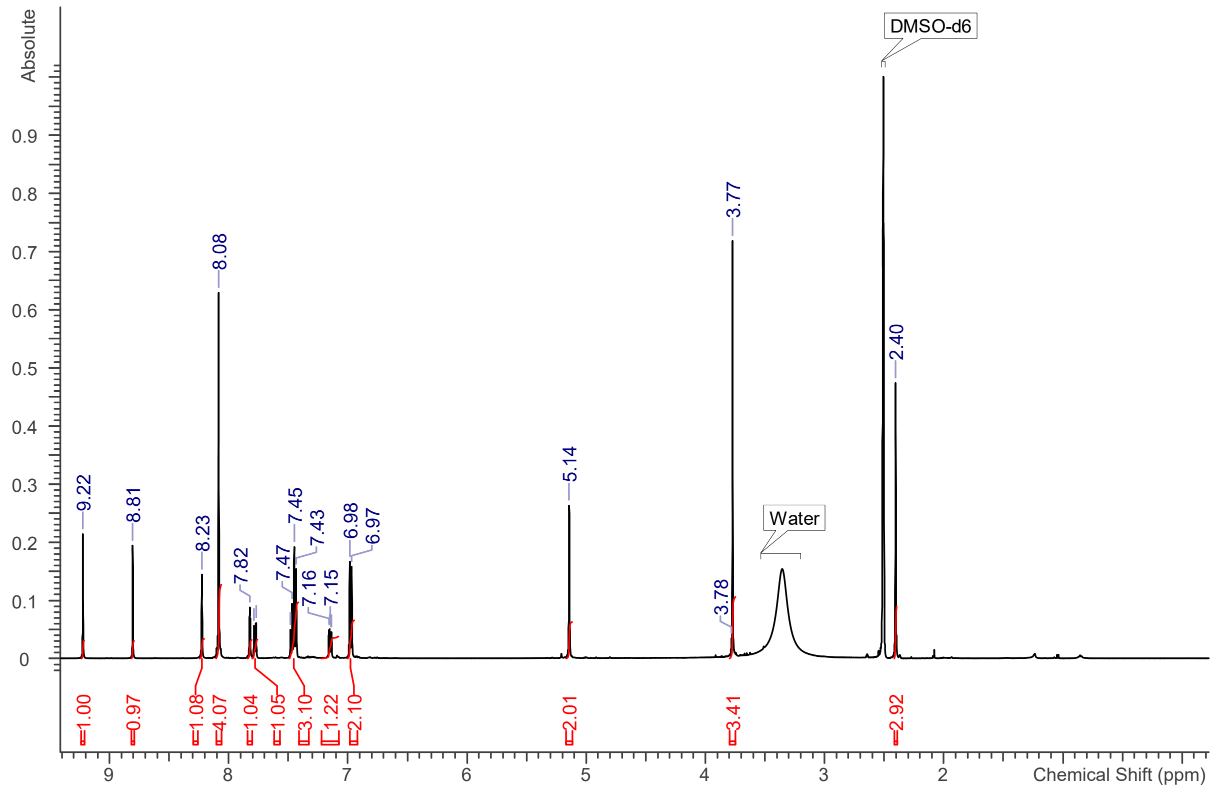

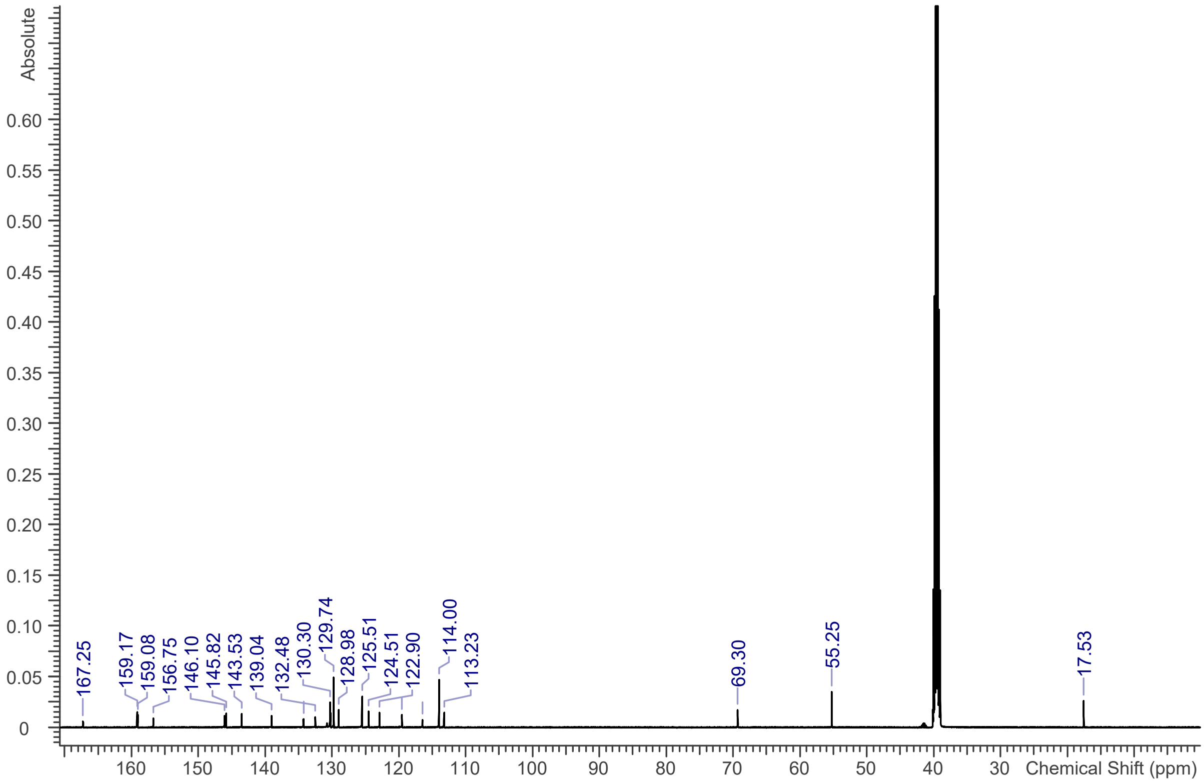

4-(1-(6-(2-((4-methoxybenzyl)oxy)phenyl)-4-methylpyridin-3-yl)-1H-1,2,3-triazol-4-yl)benzoic acid (35)

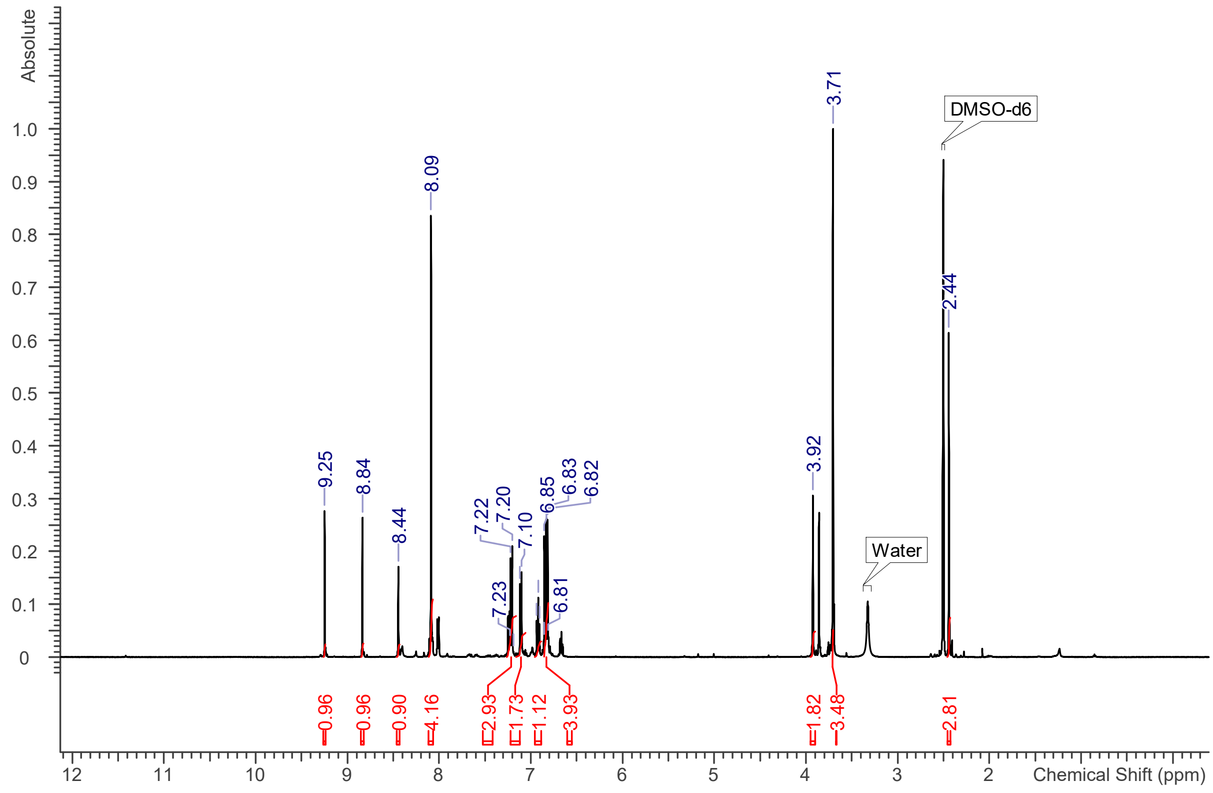

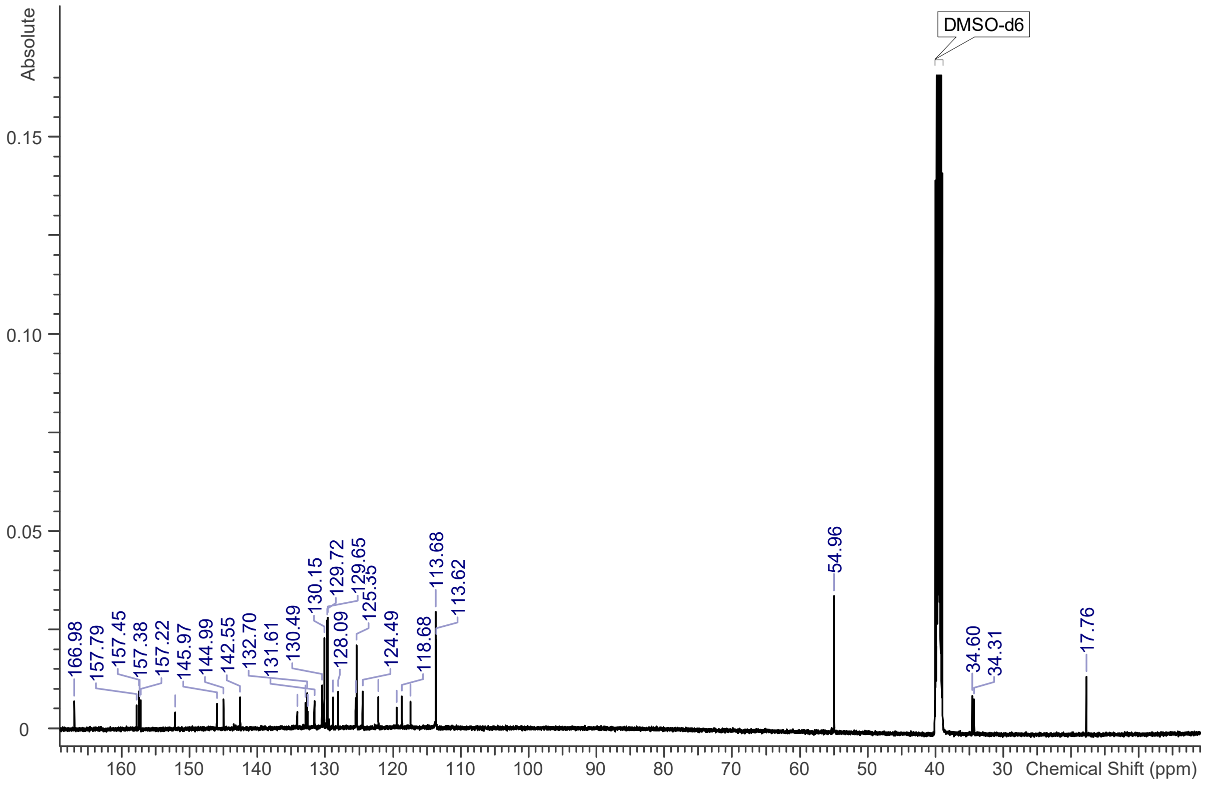

4-(1-(6-(4-((4-methoxybenzyl)oxy)phenyl)-4-methylpyridin-3-yl)-1H-1,2,3-triazol-4-yl)benzoic acid (36)

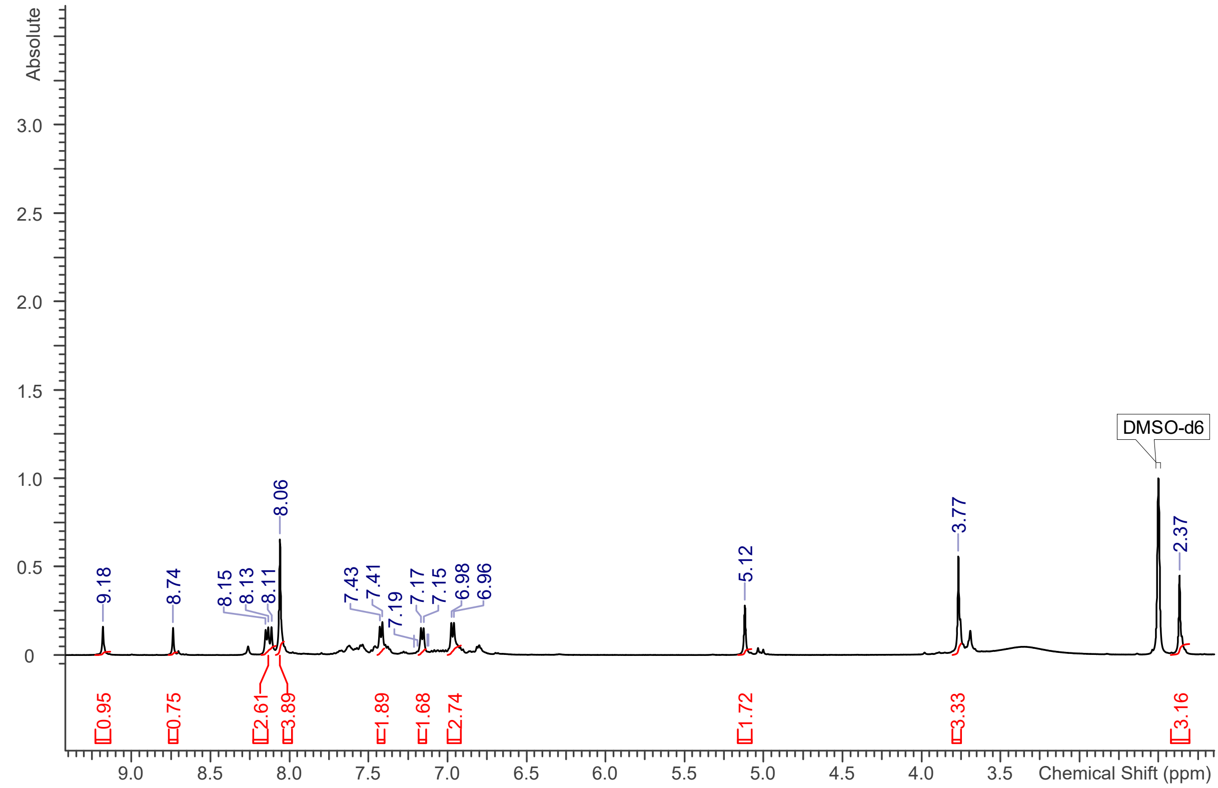

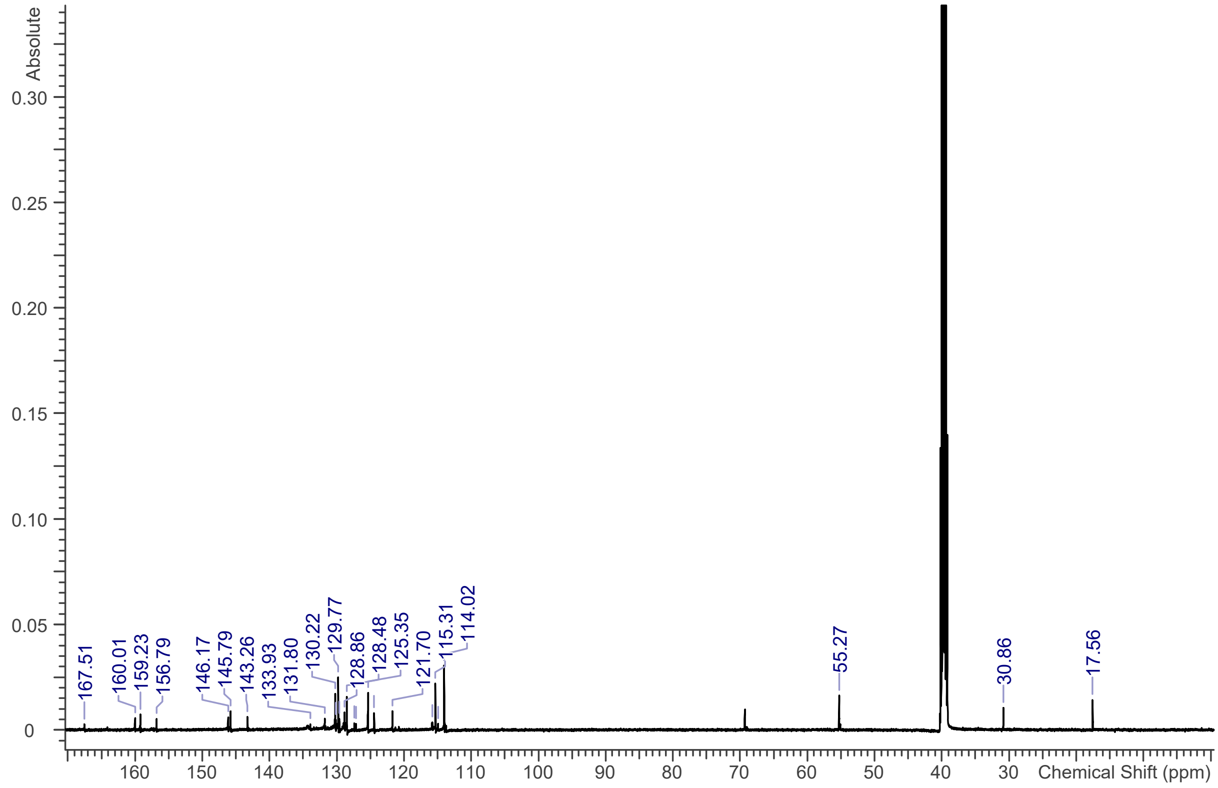

3. High Resolution Mass Spectra

4-(1-(4-methyl-[2,3'-bipyridin]-5-yl)-1H-1,2,3-triazol-4-yl)benzoic acid (4)

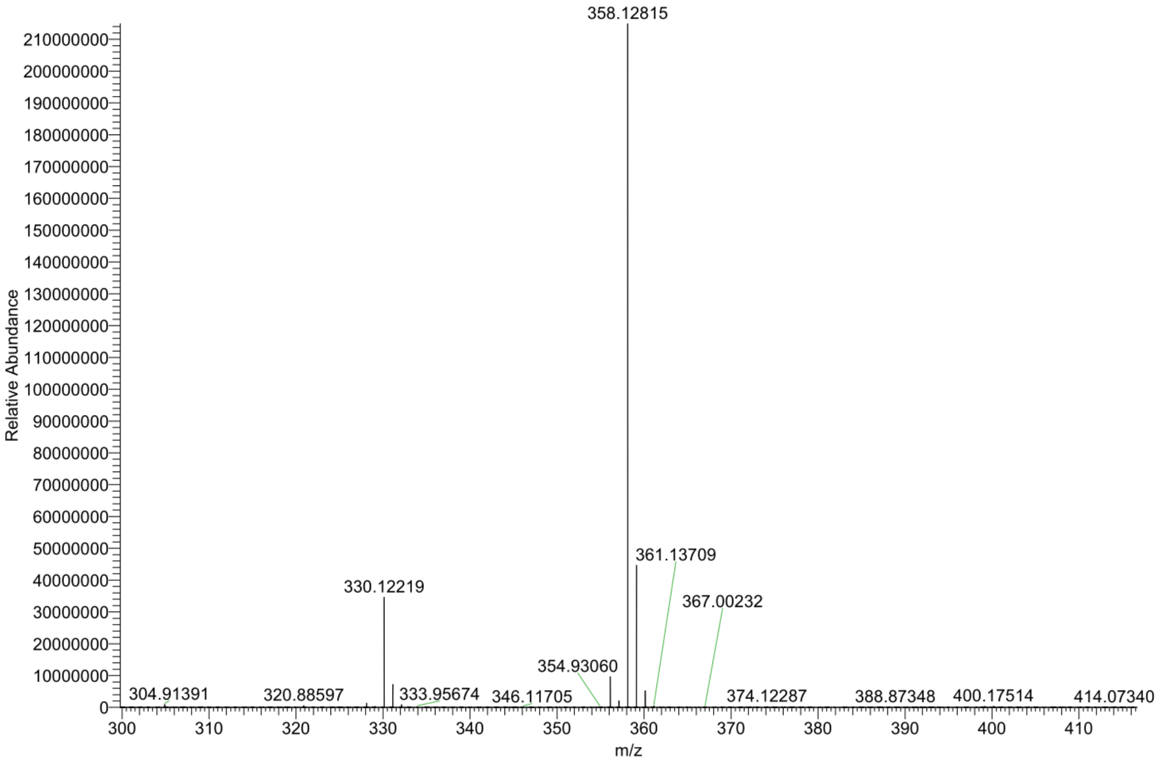

4-(1-(4-methyl-[2,4'-bipyridin]-5-yl)-1H-1,2,3-triazol-4-yl)benzoic acid (5)

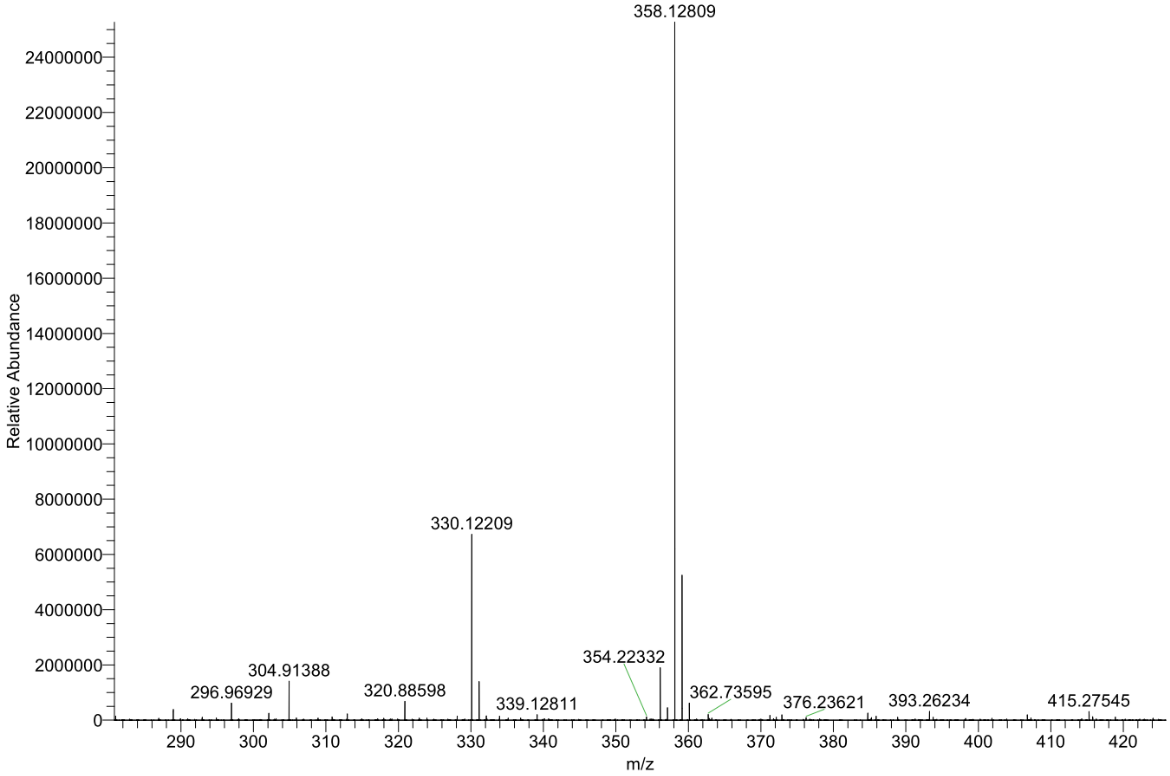

4-(1-(4-methyl-6-(pyrimidin-5-yl)pyridin-3-yl)-1H-1,2,3-triazol-4-yl)benzoic acid (6)

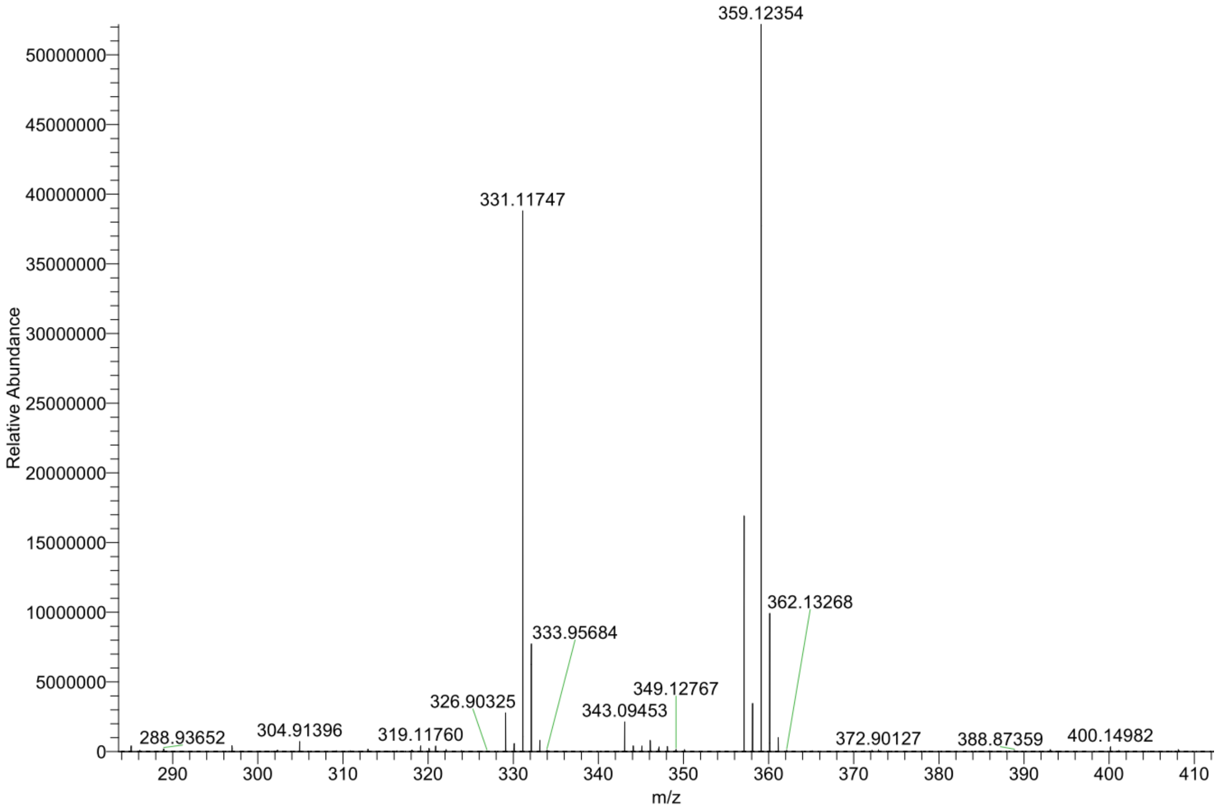

4-(1-(5'-hydroxy-4-methyl-[2,3'-bipyridin]-5-yl)-1H-1,2,3-triazol-4-yl)benzoic acid (7)

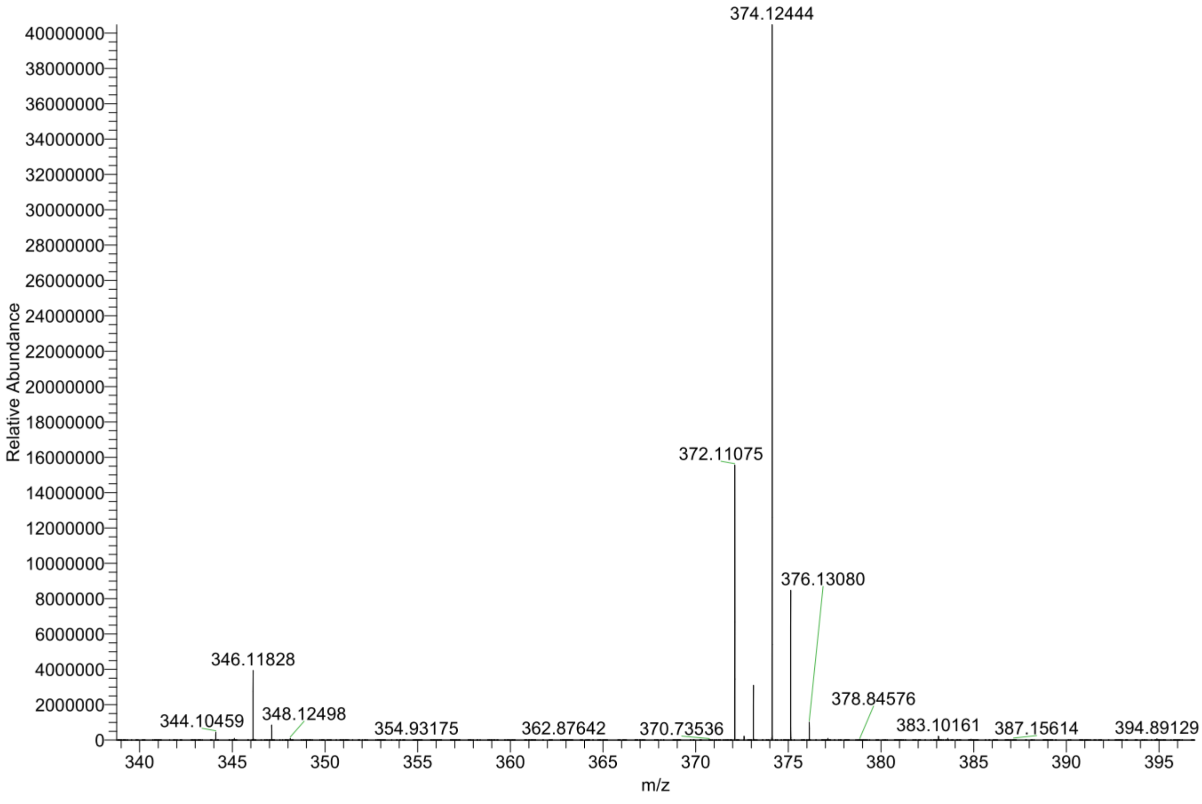

4-(1-(6'-hydroxy-4-methyl-[2,3'-bipyridin]-5-yl)-1H-1,2,3-triazol-4-yl)benzoic acid (8)

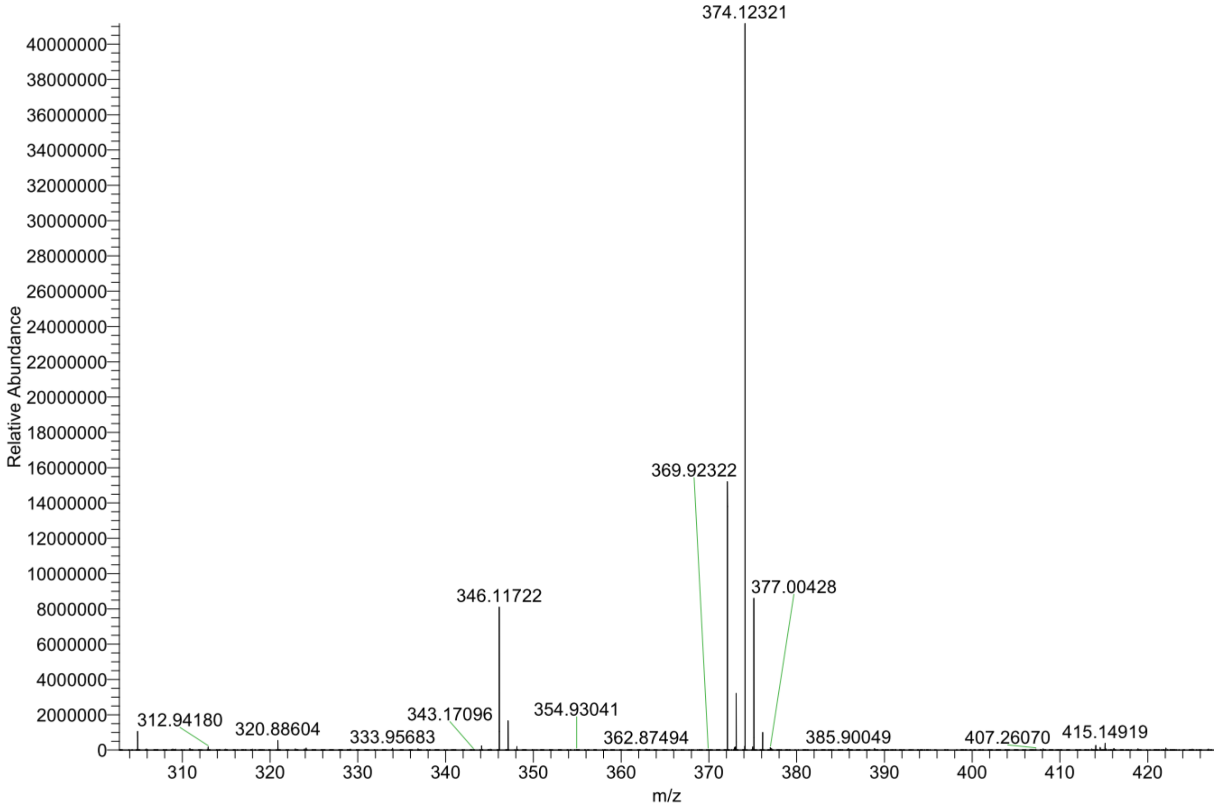

4-(1-(5'-methoxy-4-methyl-[2,3'-bipyridin]-5-yl)-1H-1,2,3-triazol-4-yl)benzoic acid (9)

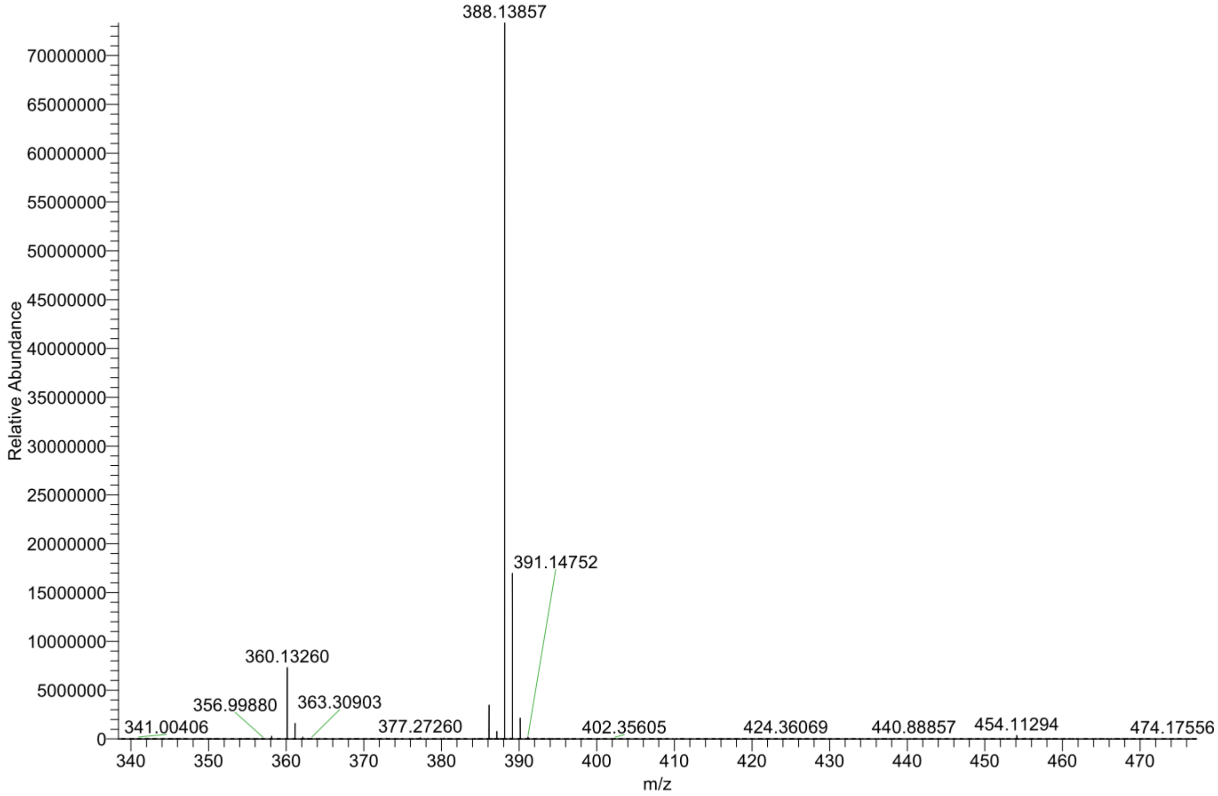

4-(1-(6'-methoxy-4-methyl-[2,3'-bipyridin]-5-yl)-1H-1,2,3-triazol-4-yl)benzoic acid (10)

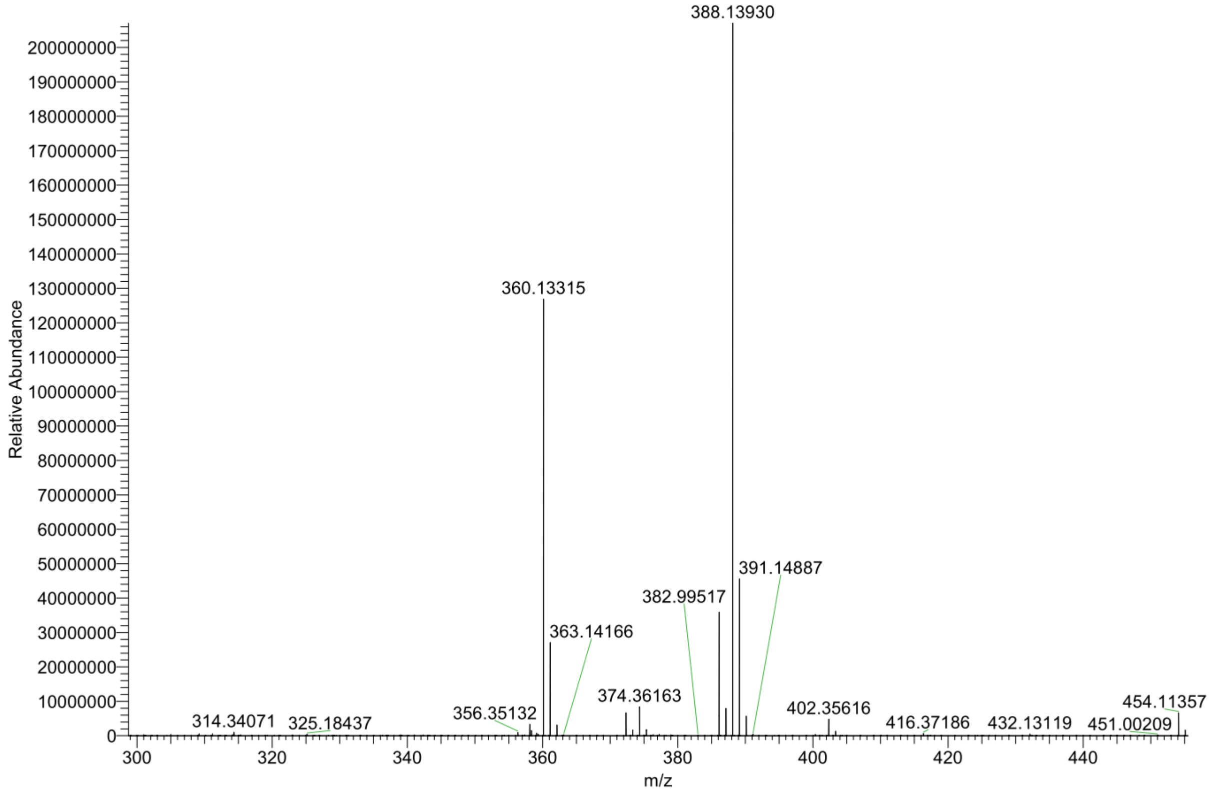

4-(1-(2'-methoxy-4,5'-dimethyl-[2,3'-bipyridin]-5-yl)-1H-1,2,3-triazol-4-yl)benzoic acid (11)

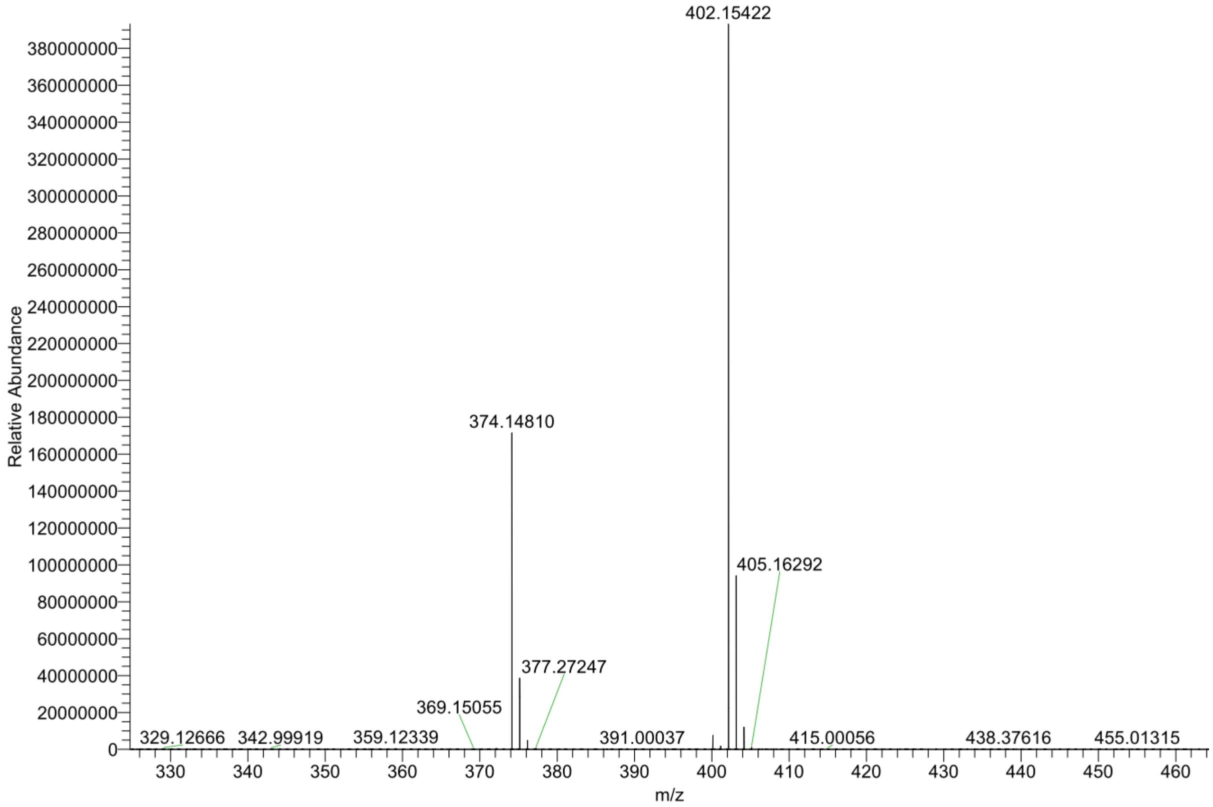

4-(1-(4-methyl-6-(quinolin-3-yl)pyridin-3-yl)-1H-1,2,3-triazol-4-yl)benzoic acid (12)

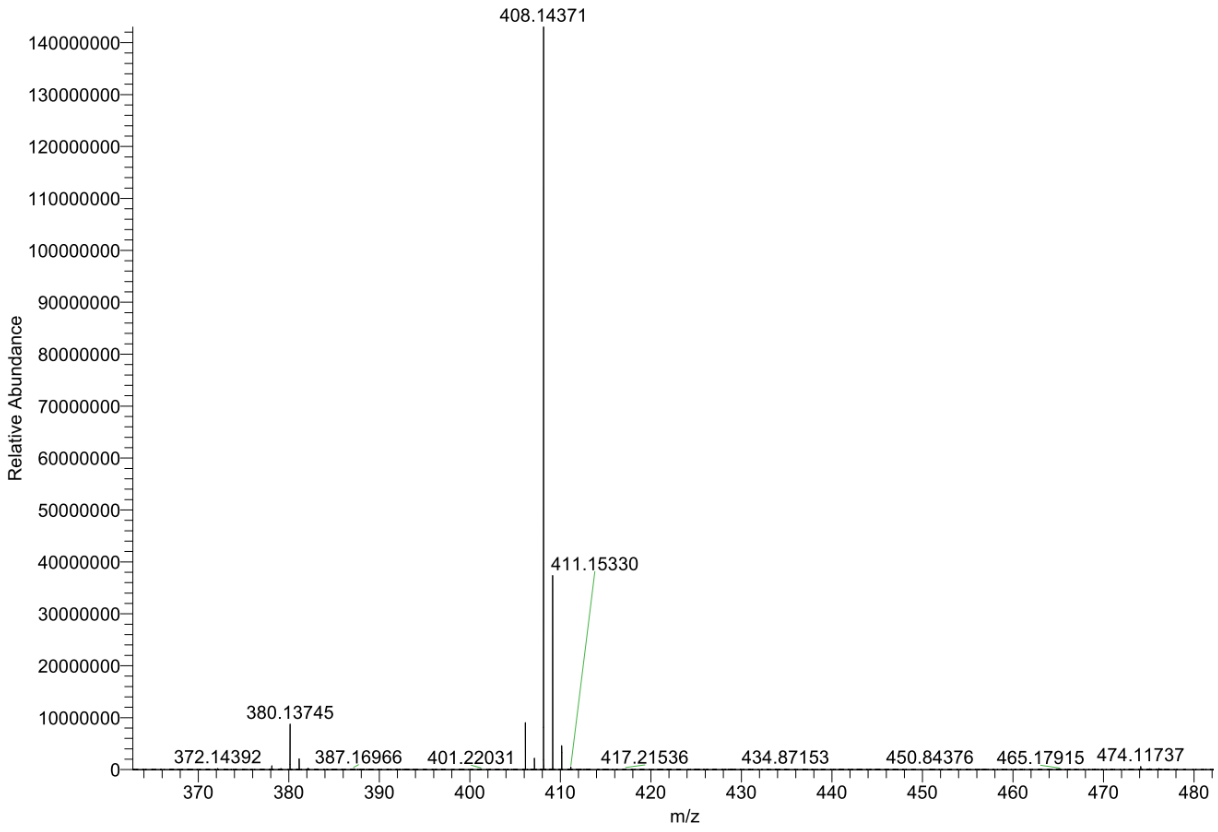

4-(1-(6-(2-chlorophenyl)-4-methylpyridin-3-yl)-1H-1,2,3-triazol-4-yl)benzoic acid (14)

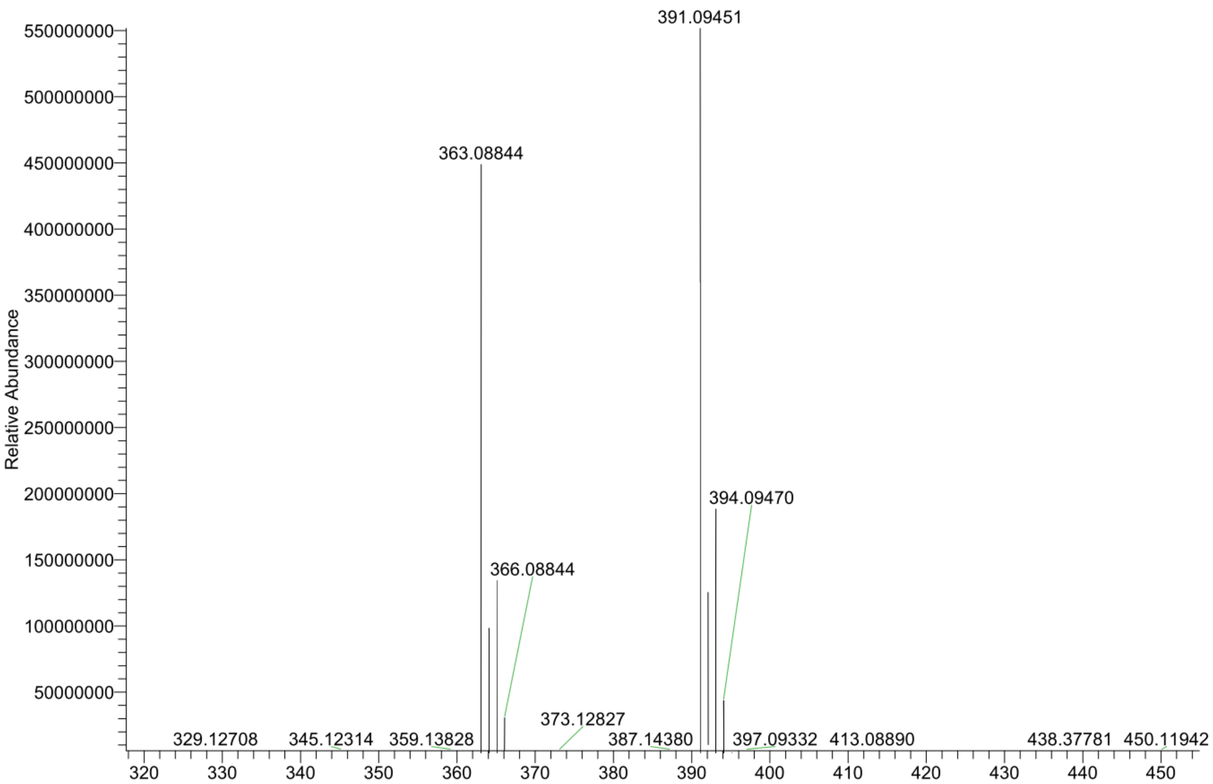

4-(1-(6-(3-chlorophenyl)-4-methylpyridin-3-yl)-1H-1,2,3-triazol-4-yl)benzoic acid (15)

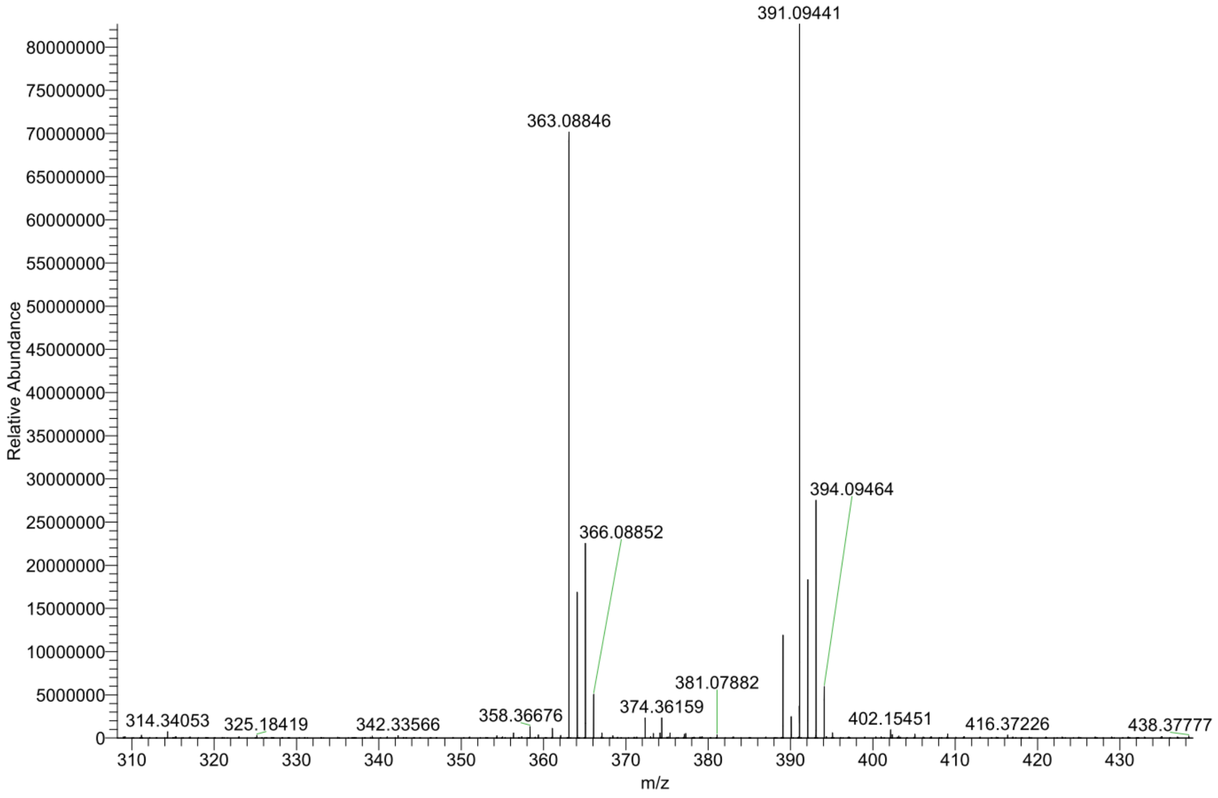

4-(1-(6-(2-methoxyphenyl)-4-methylpyridin-3-yl)-1H-1,2,3-triazol-4-yl)benzoic acid (17)

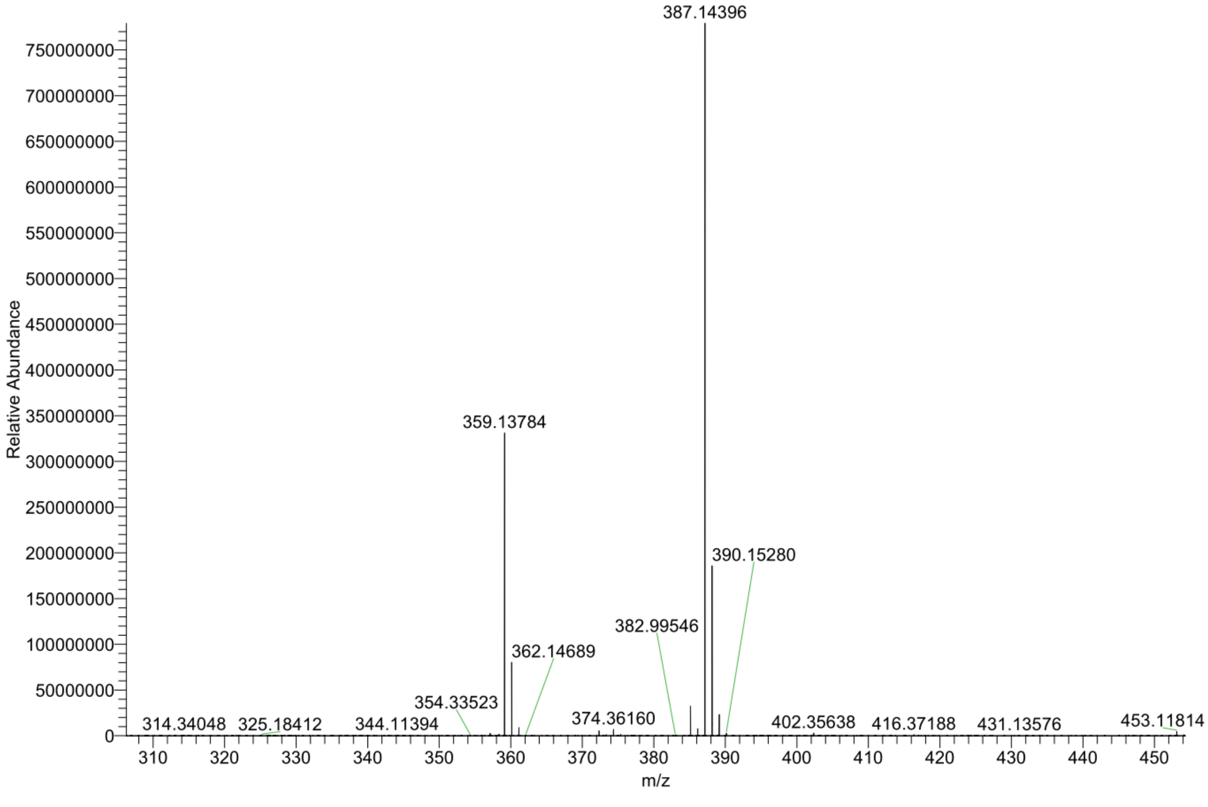

4-(1-(6-(3-methoxyphenyl)-4-methylpyridin-3-yl)-1H-1,2,3-triazol-4-yl)benzoic acid (18)

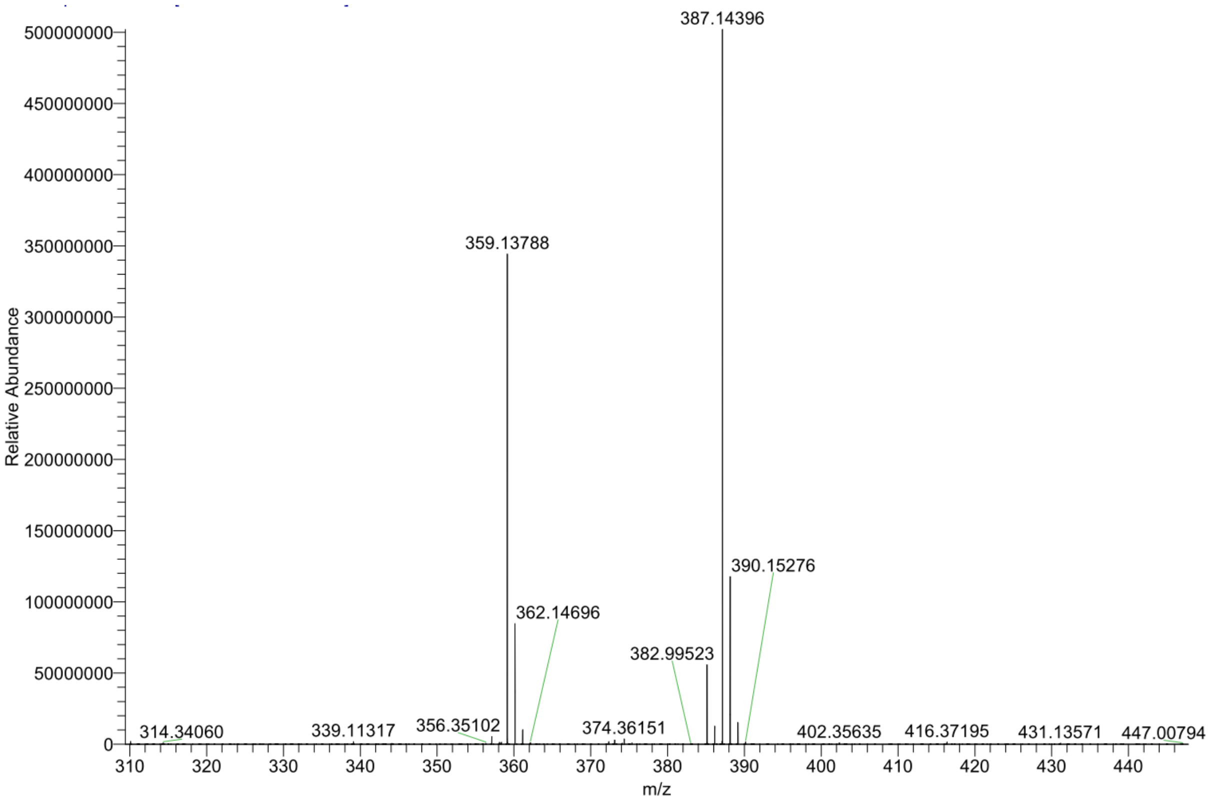

4-(1-(6-(4-methoxyphenyl)-4-methylpyridin-3-yl)-1H-1,2,3-triazol-4-yl)benzoic acid (19)

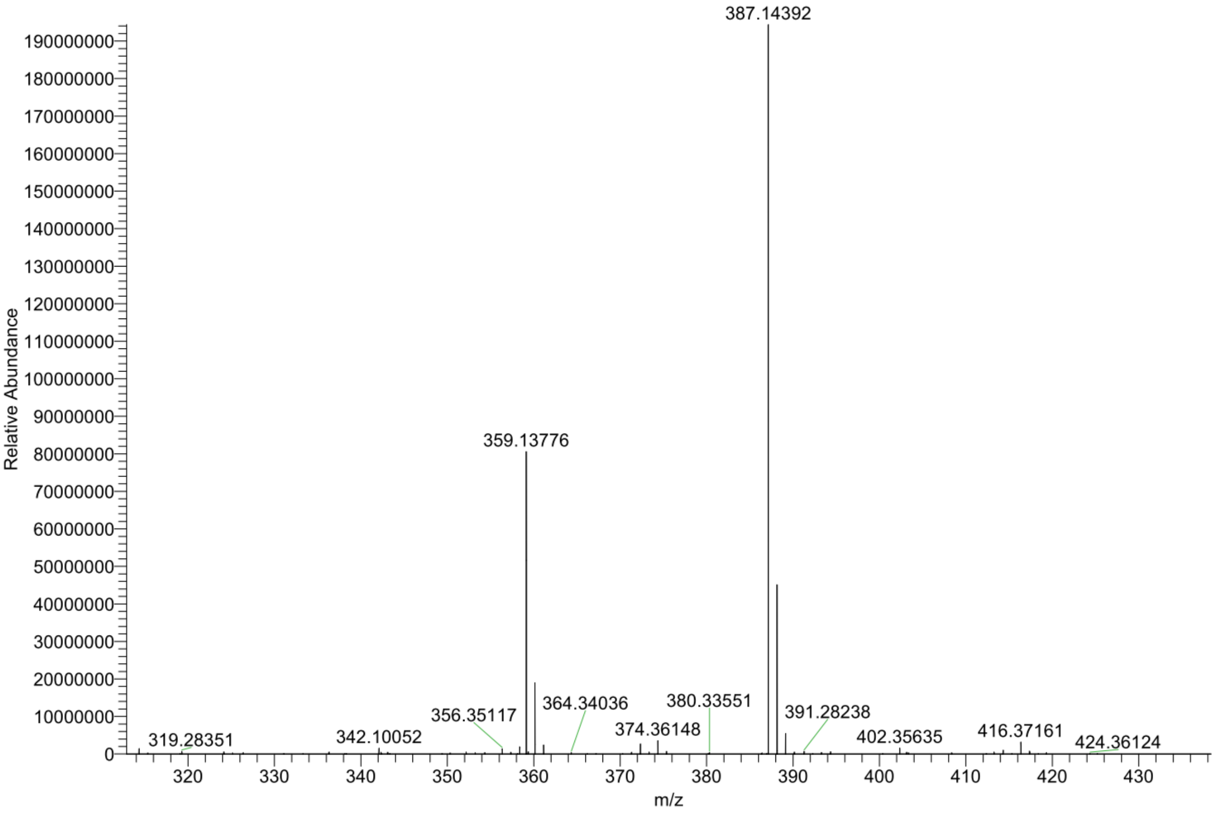

4-(1-(6-(2-chloro-4-methoxyphenyl)-4-methylpyridin-3-yl)-1H-1,2,3-triazol-4-yl)benzoic acid (20)

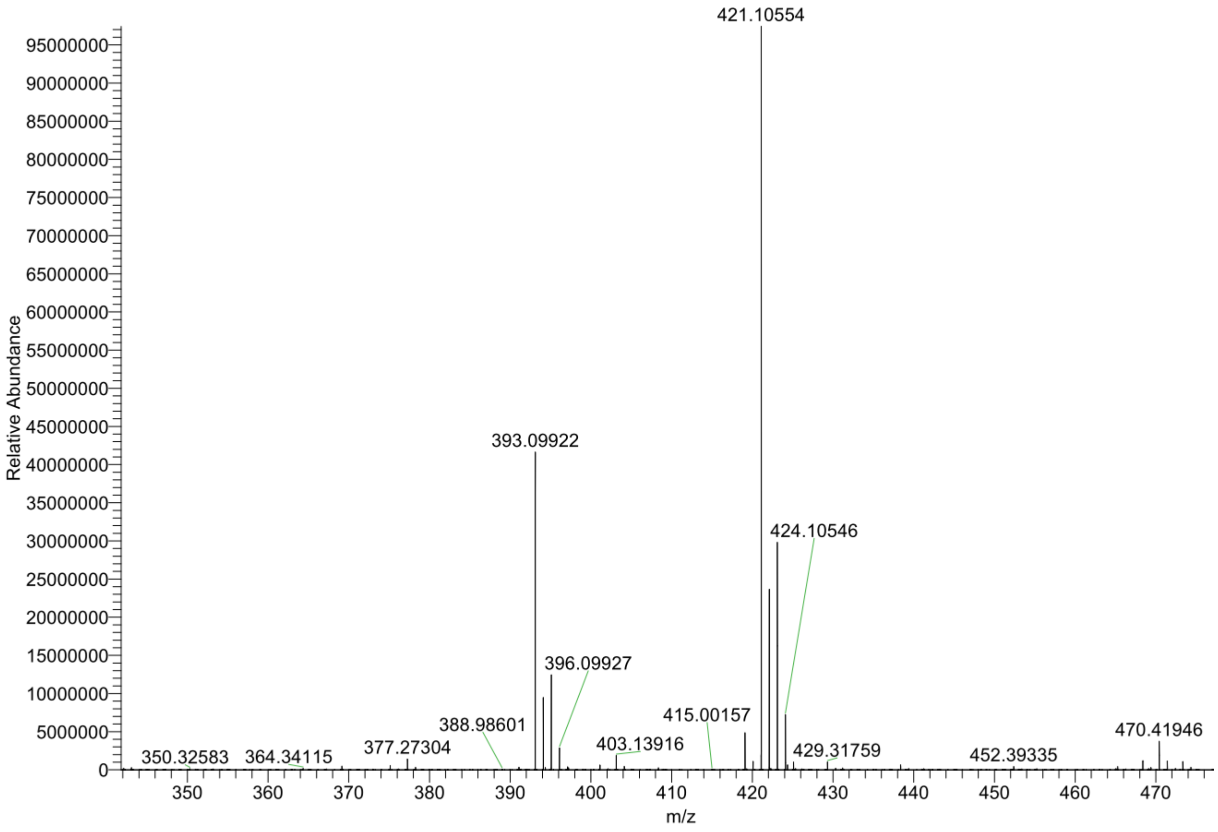

4-(1-(6-(3-hydroxyphenyl)-4-methylpyridin-3-yl)-1H-1,2,3-triazol-4-yl)benzoic acid (22)

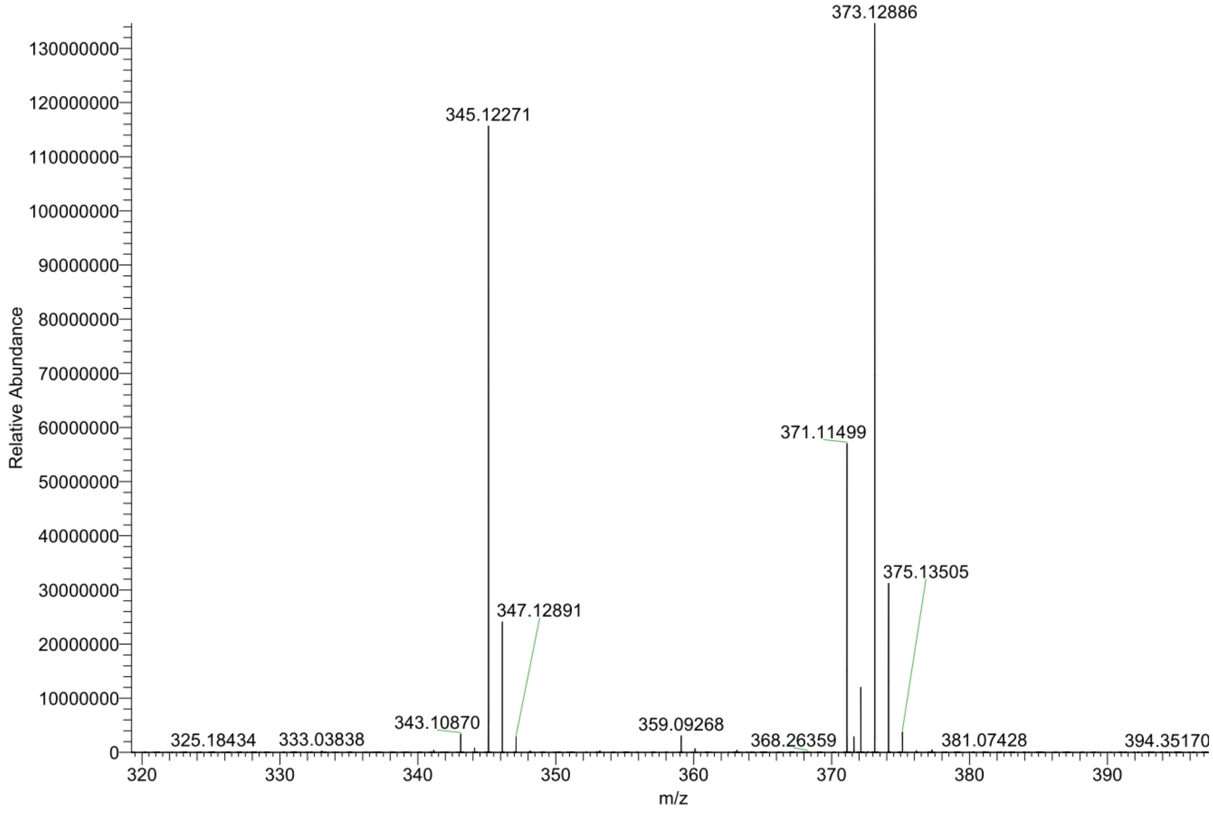

4-(1-(6-(4-hydroxyphenyl)-4-methylpyridin-3-yl)-1H-1,2,3-triazol-4-yl)benzoic acid (23)

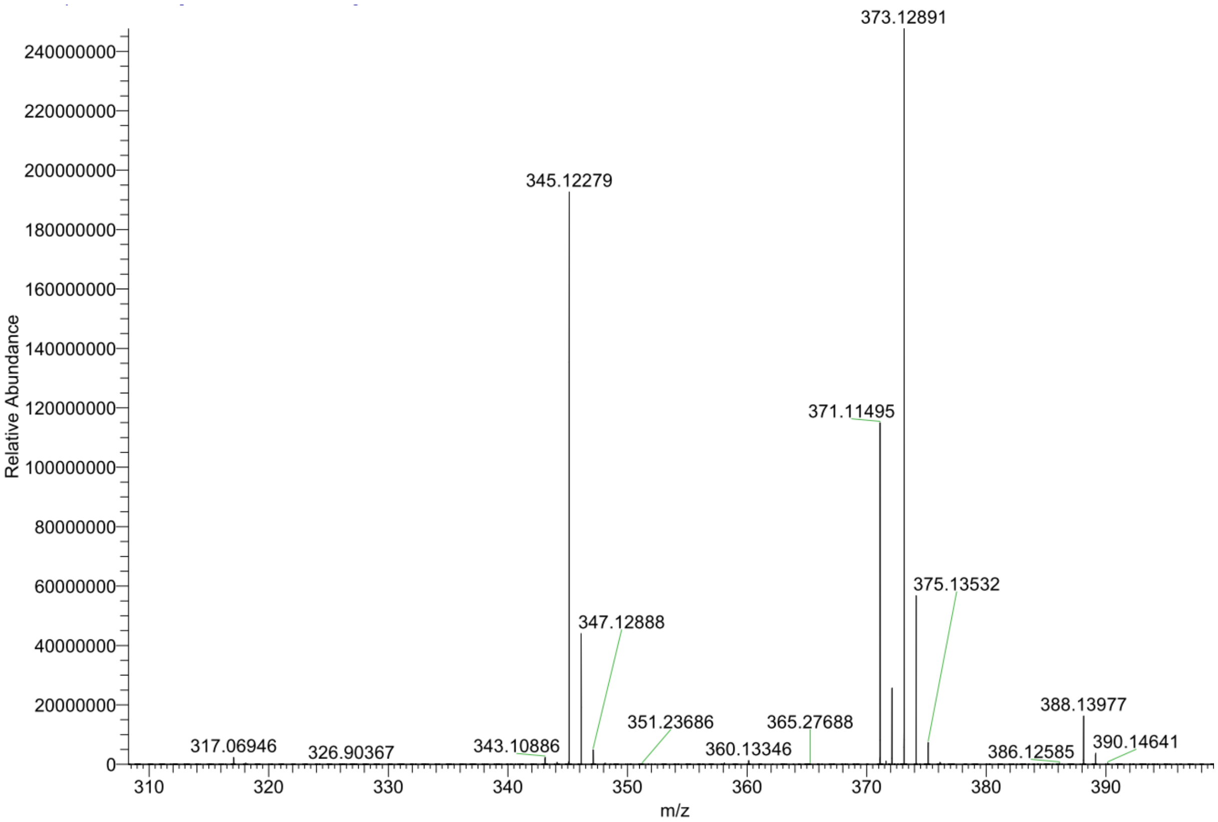

4-(1-(4-methyl-6-(3-(methylcarbamoyl)phenyl)pyridin-3-yl)-1H-1,2,3-triazol-4-yl)benzoic acid (24)

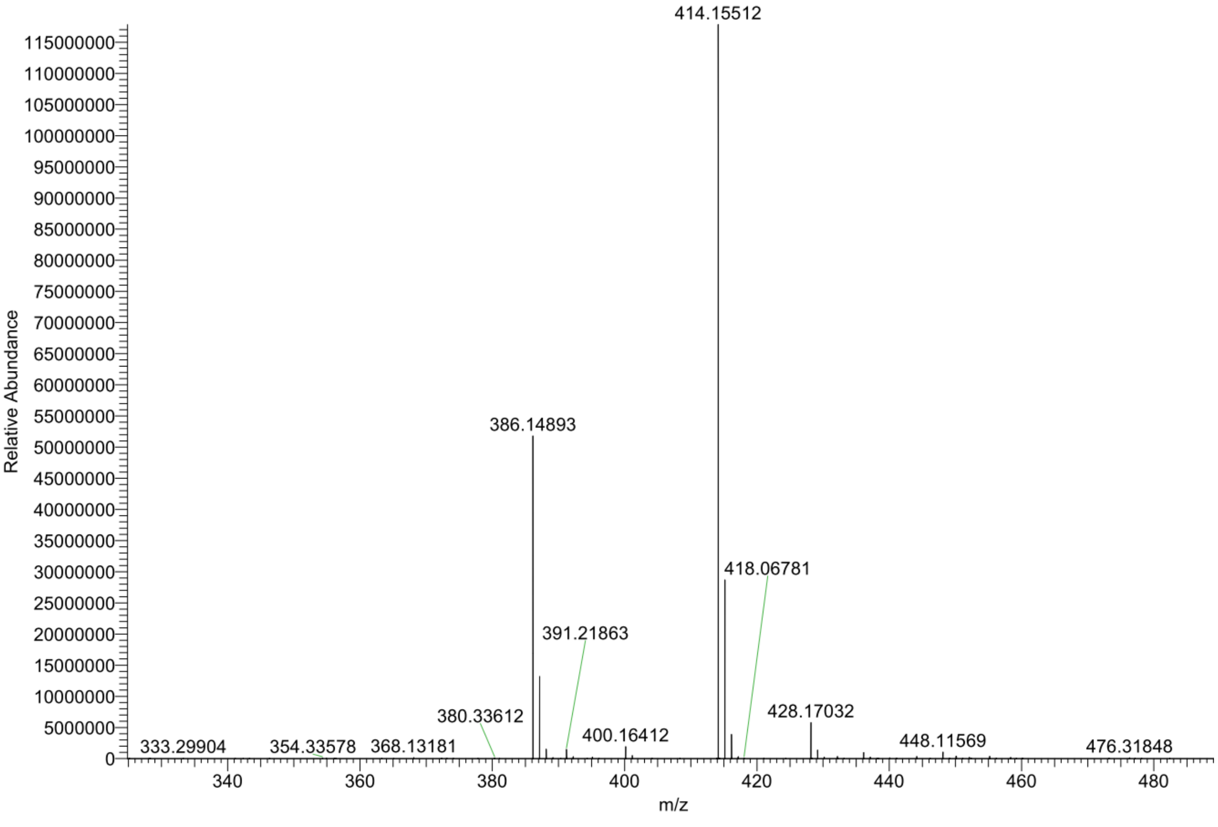

4-(1-(4-methyl-6-(4-(methylcarbamoyl)phenyl)pyridin-3-yl)-1H-1,2,3-triazol-4-yl)benzoic acid (25)

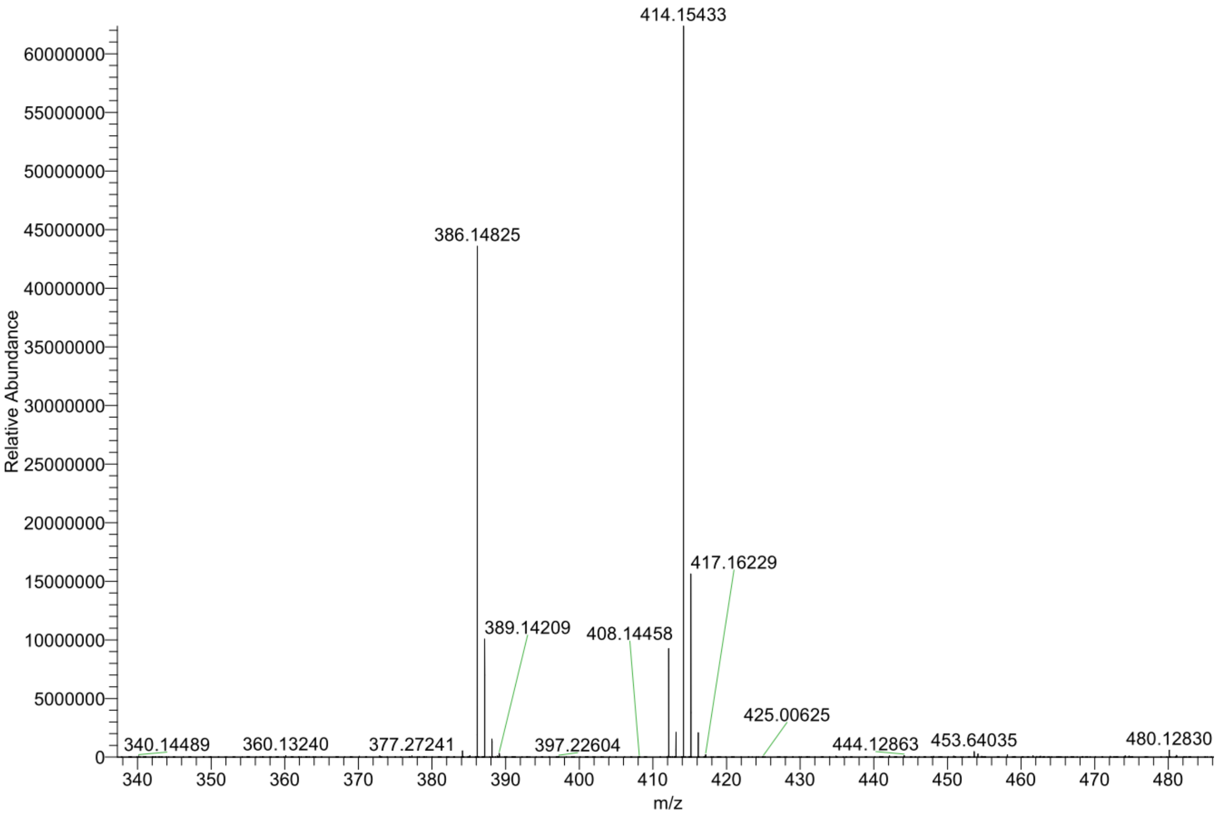

3-(5-(4-(4-Carboxyphenyl)-1H-1,2,3-triazol-1-yl)-4-methylpyridin-2-yl)benzoic acid (26)

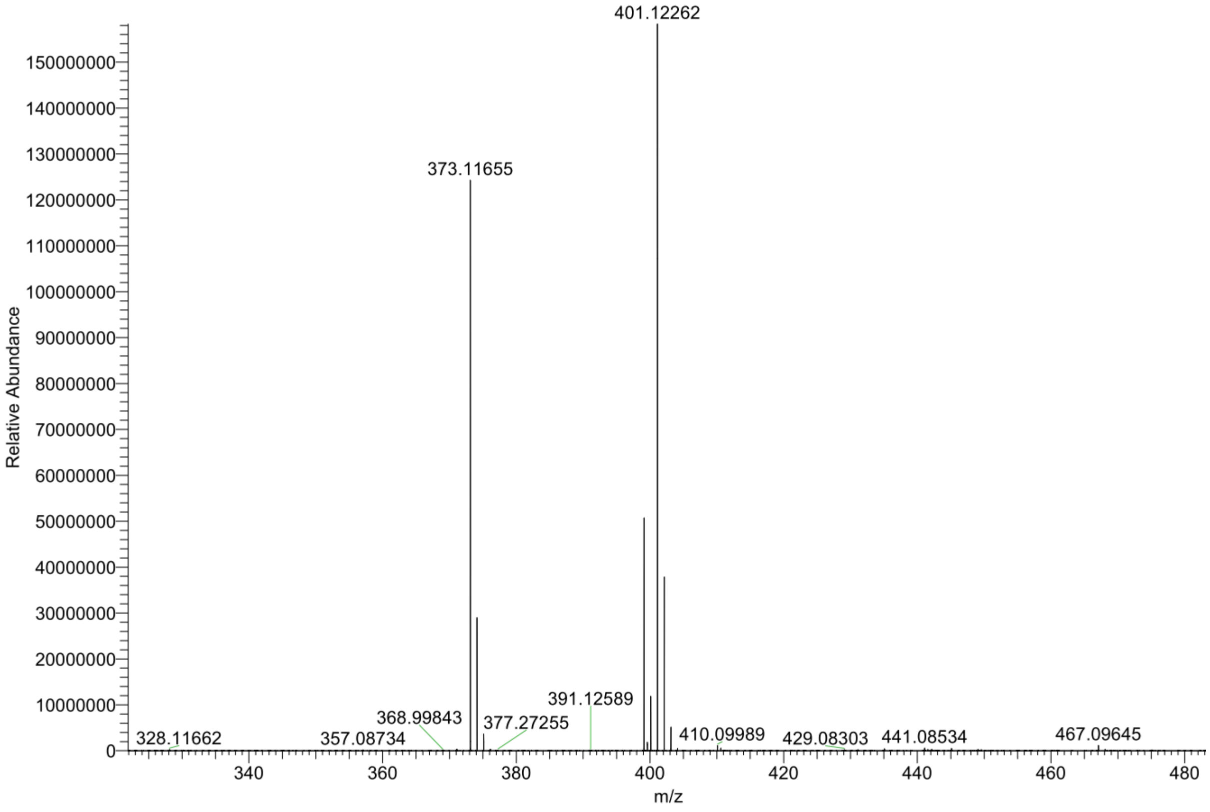

4-(5-(4-(4-Carboxyphenyl)-1H-1,2,3-triazol-1-yl)-4-methylpyridin-2-yl)benzoic acid (27)

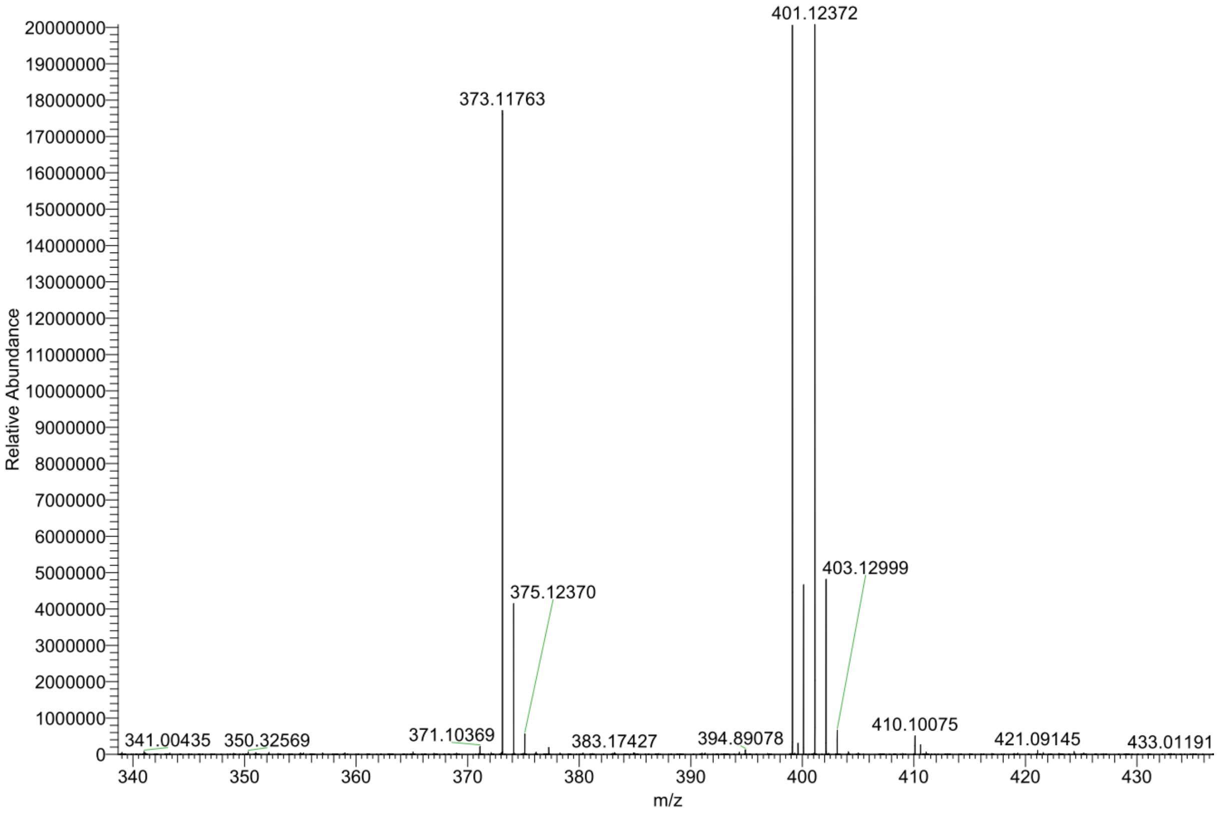

4-(1-(4-methyl-6-(3-phenoxyphenyl)pyridin-3-yl)-1H-1,2,3-triazol-4-yl)benzoic acid (28)

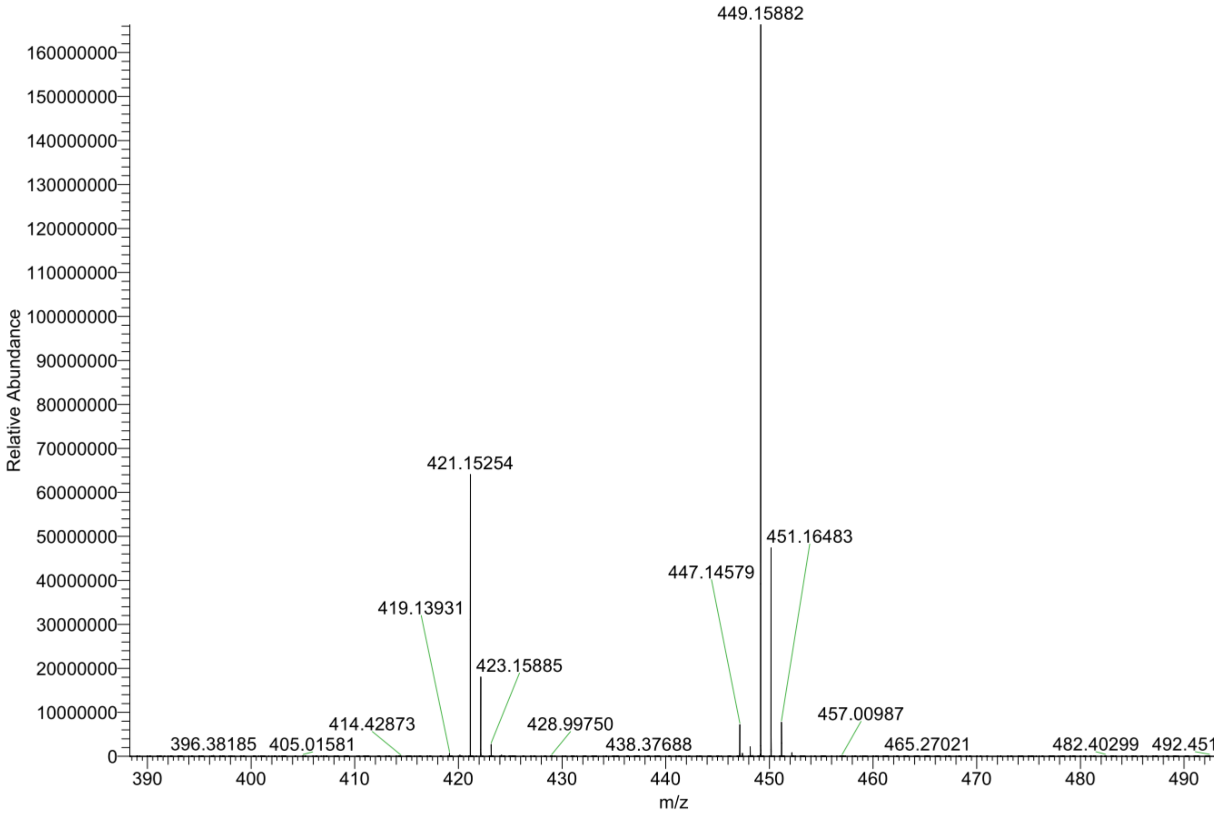

4-(1-(6-(3-(benzyloxy)phenyl)-4-methylpyridin-3-yl)-1H-1,2,3-triazol-4-yl)benzoic acid (29)

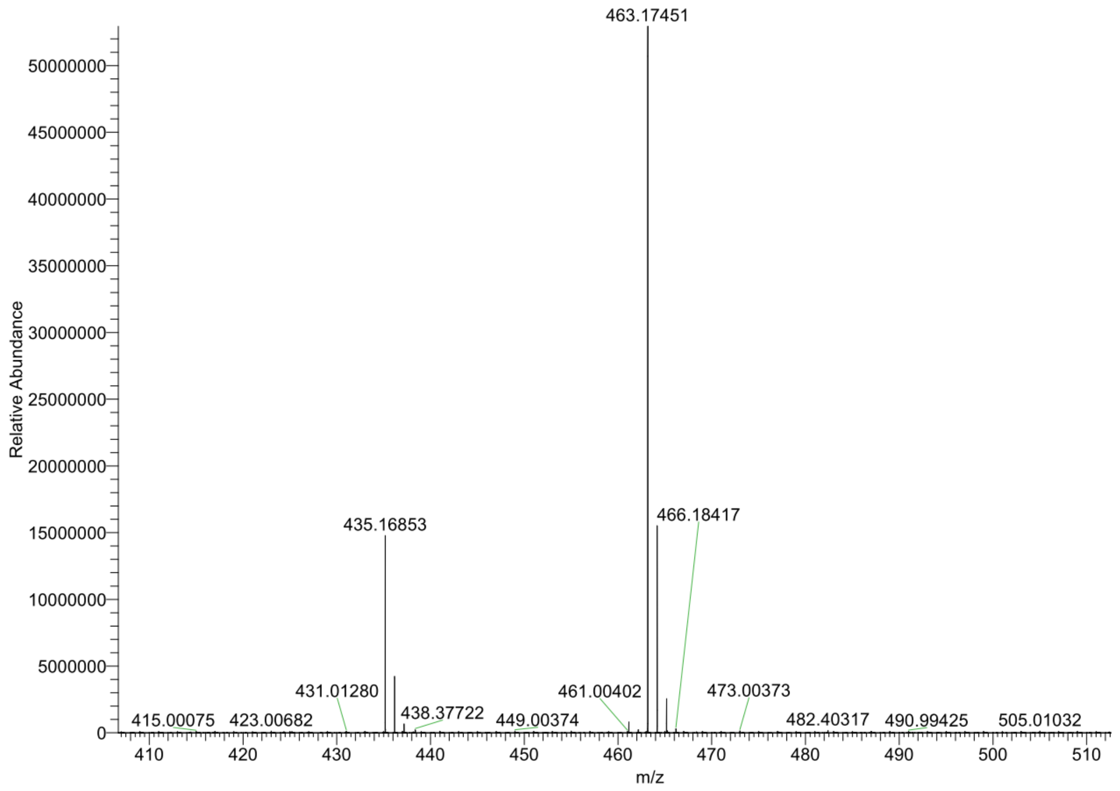

4-(1-(6-(3-((2-chlorobenzyl)oxy)phenyl)-4-methylpyridin-3-yl)-1H-1,2,3-triazol-4-yl) benzoic acid (30)

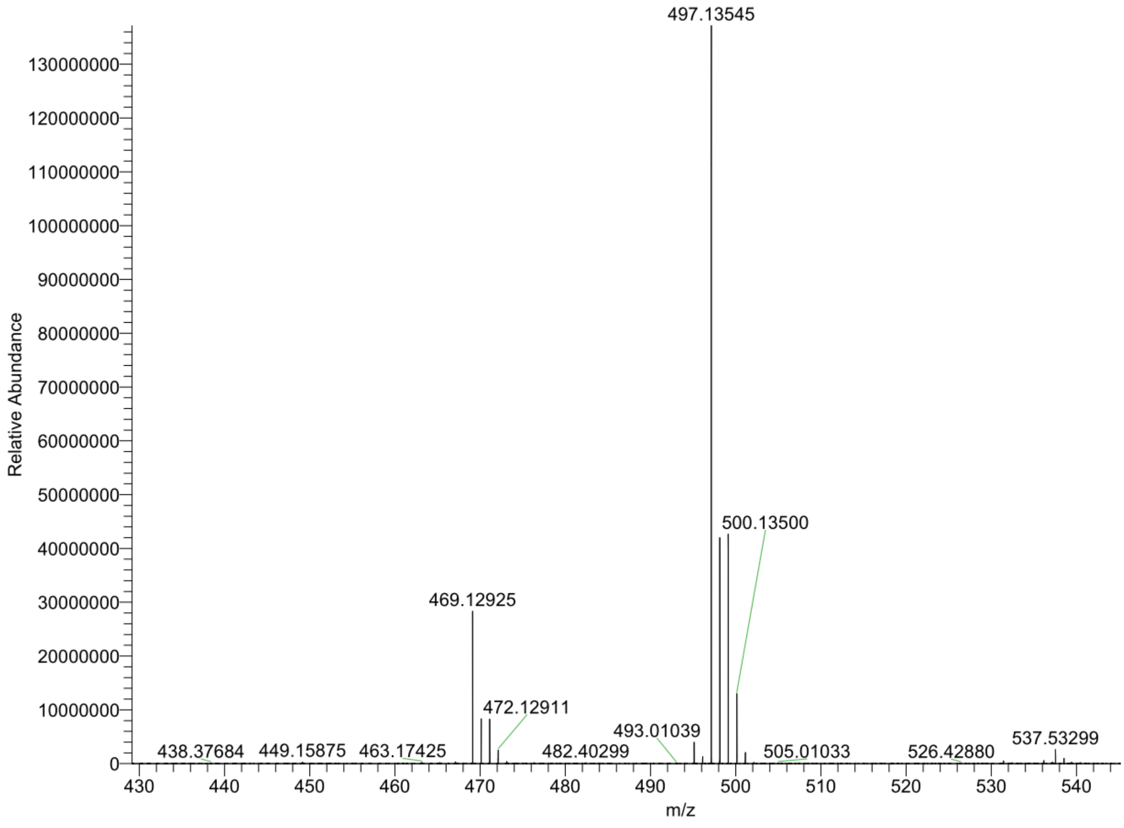

4-(1-(6-(3-((2-methoxybenzyl)oxy)phenyl)-4-methylpyridin-3-yl)-1H-1,2,3-triazol-4-yl) benzoic acid (31)

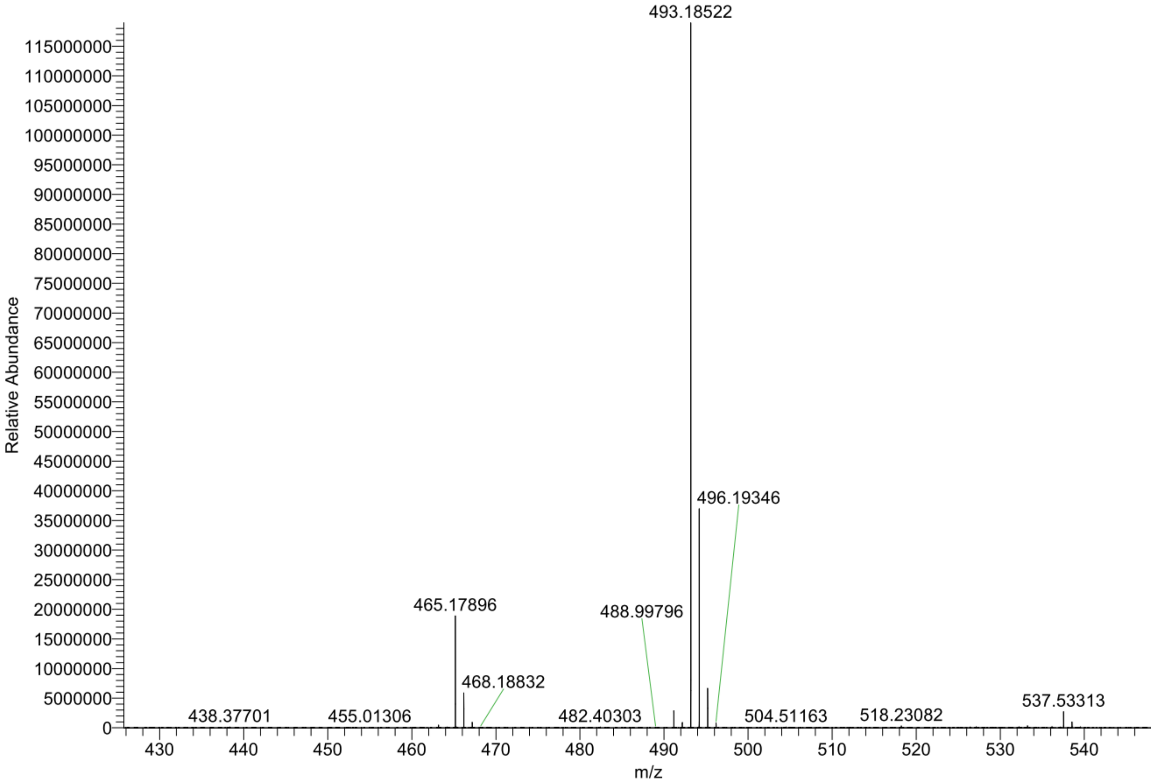

4-(1-(6-(3-((3-methoxybenzyl)oxy)phenyl)-4-methylpyridin-3-yl)-1H-1,2,3-triazol-4-yl) benzoic acid (32)

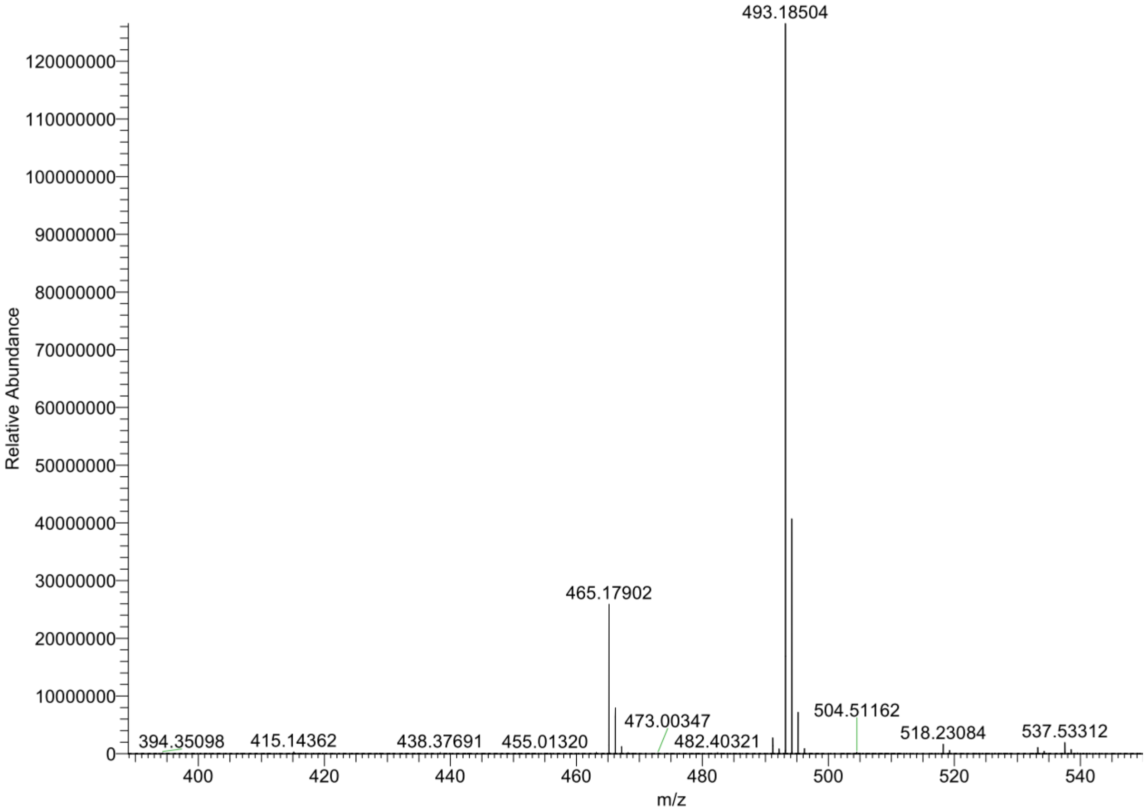

4-(1-(6-(3-((3,5-dimethoxybenzyl)oxy)phenyl)-4-methylpyridin-3-yl)-1H-1,2,3-triazol-4-yl) benzoic acid (33)

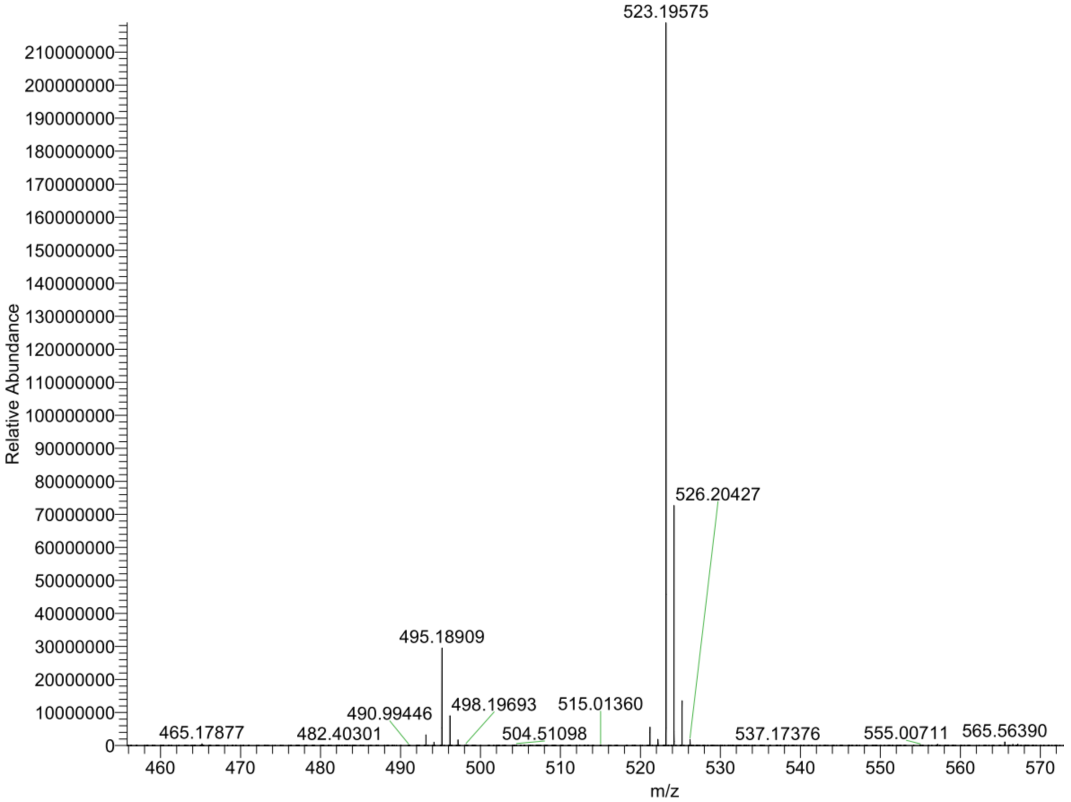

4-(1-(6-(3-((4-methoxybenzyl)oxy)phenyl)-4-methylpyridin-3-yl)-1H-1,2,3-triazol-4-yl) benzoic acid (34)

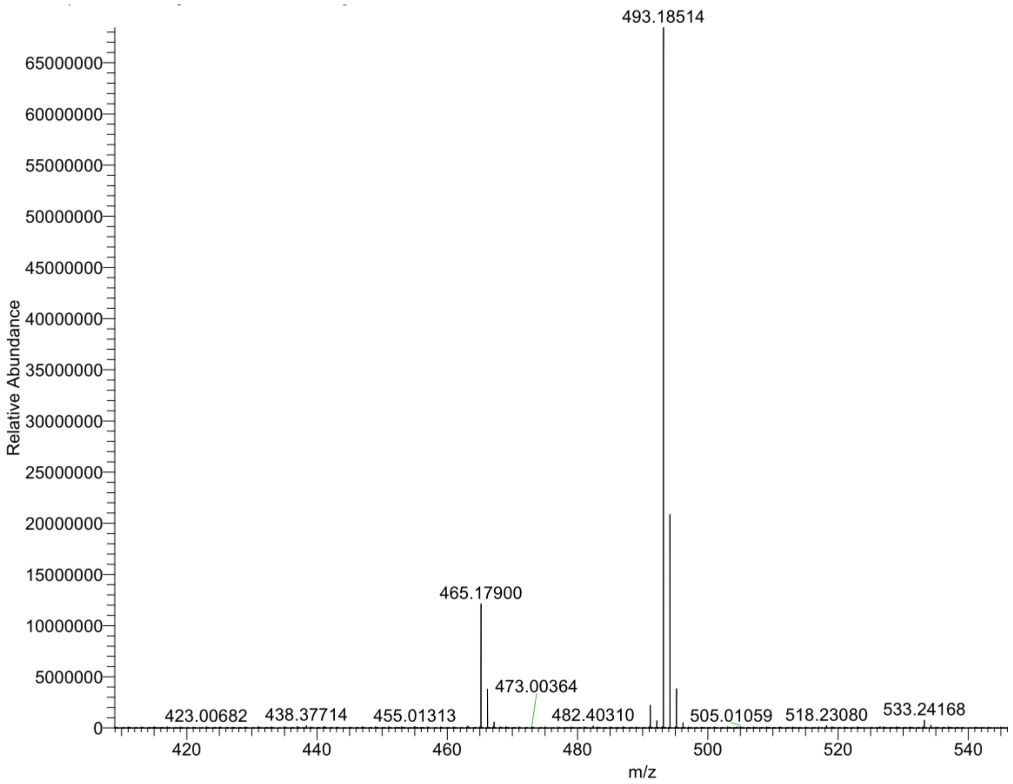

4-(1-(6-(2-((4-methoxybenzyl)oxy)phenyl)-4-methylpyridin-3-yl)-1H-1,2,3-triazol-4-yl)benzoic acid (35)

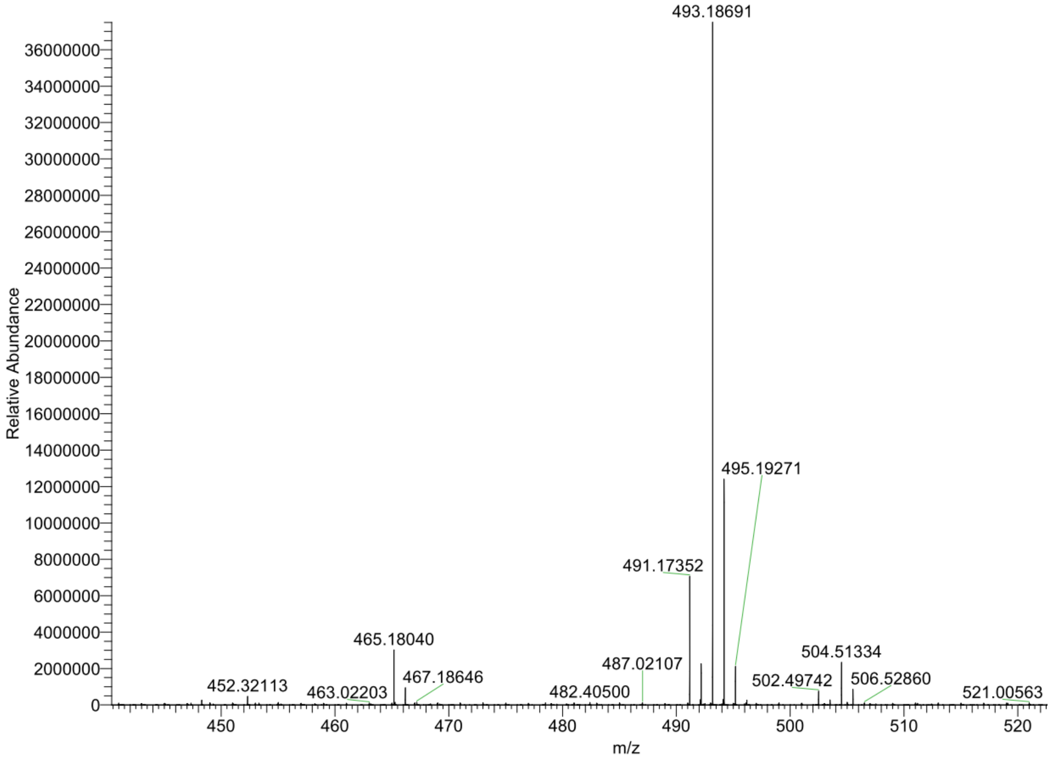

4-(1-(6-(4-((4-methoxybenzyl)oxy)phenyl)-4-methylpyridin-3-yl)-1H-1,2,3-triazol-4-yl)benzoic acid (36)

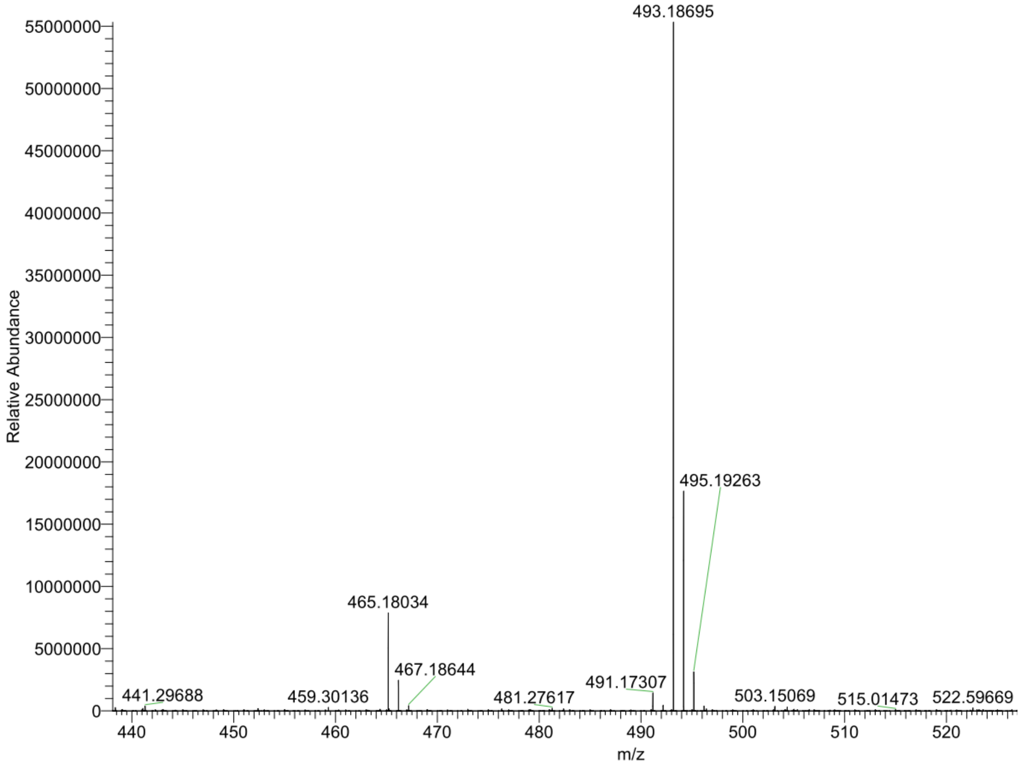

4. HPLC Traces

4

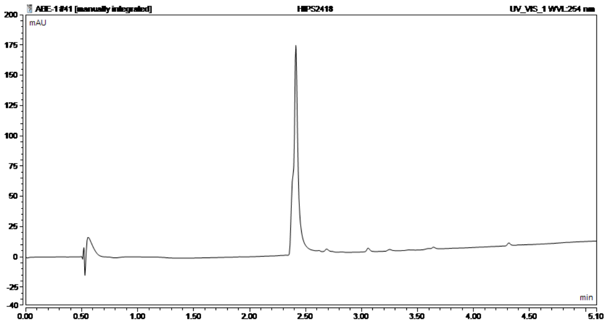

5

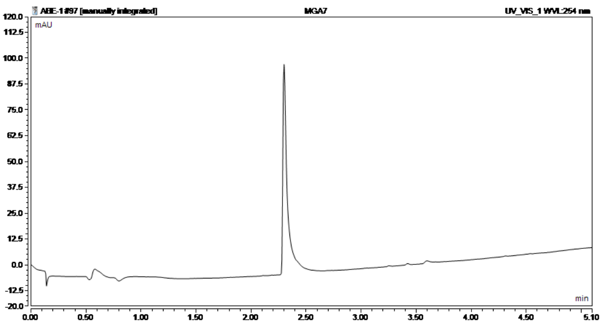

6

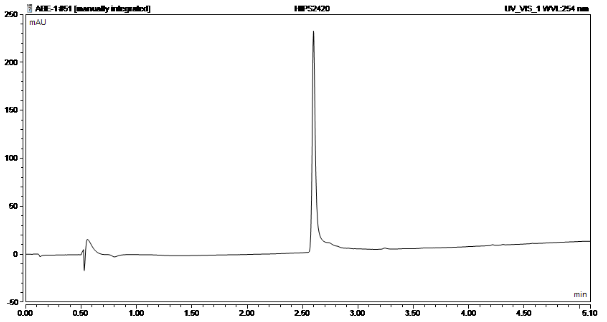

7

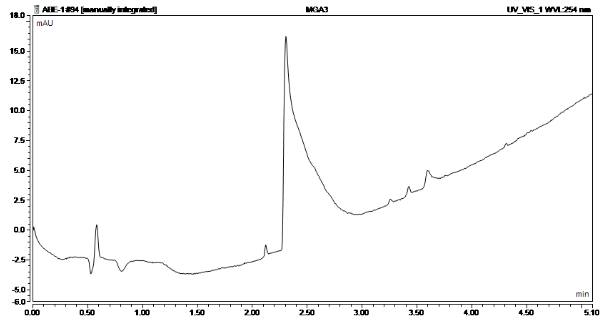

8

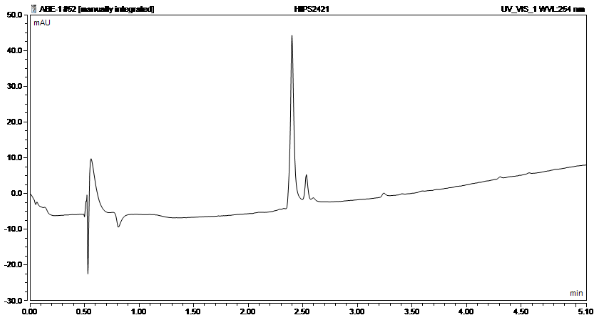

9

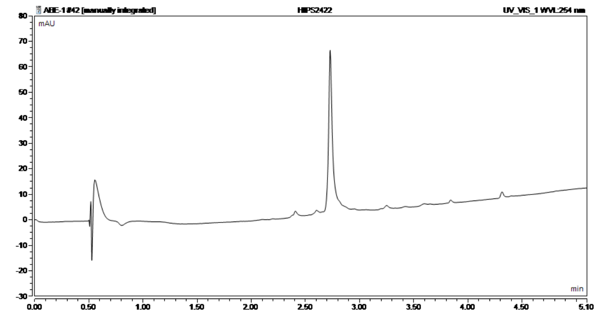

10

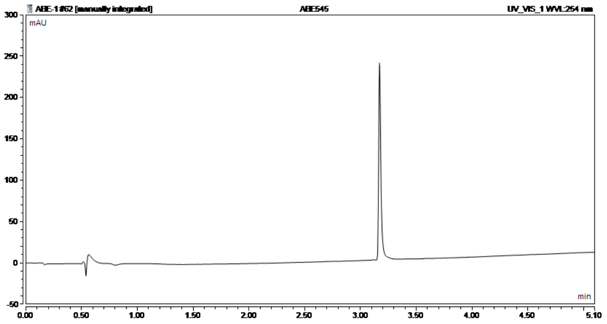

11

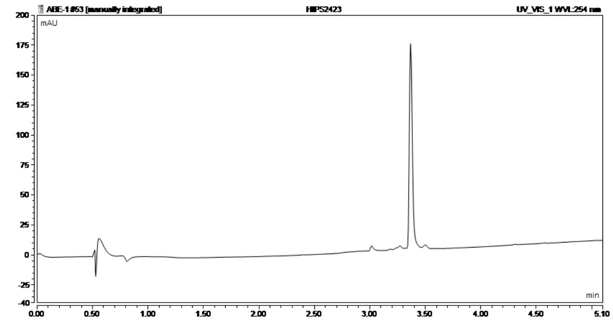

12

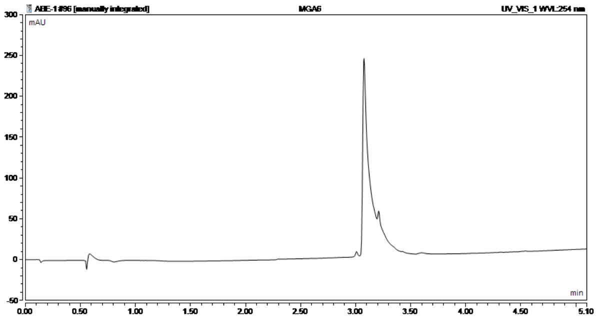

14

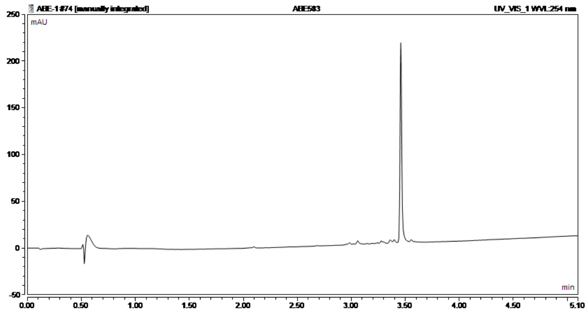

15

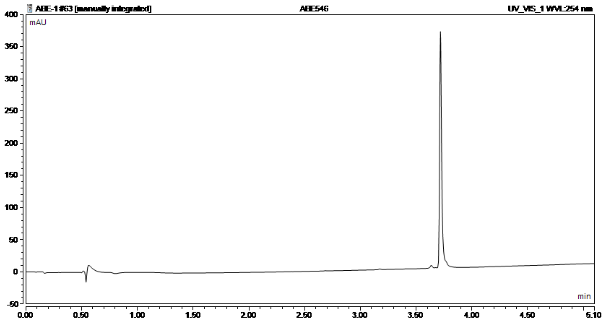

17

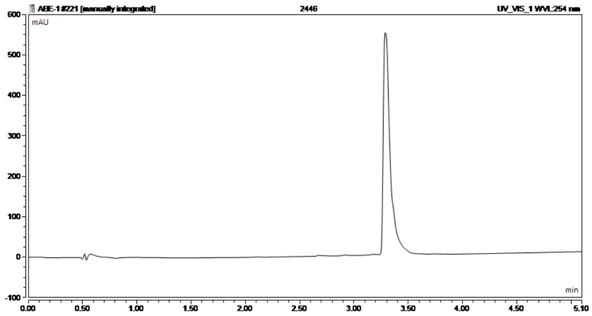

18

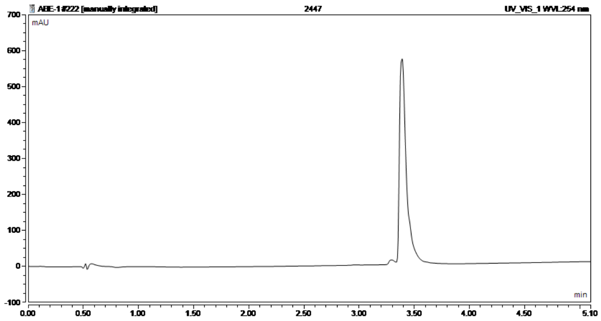

19

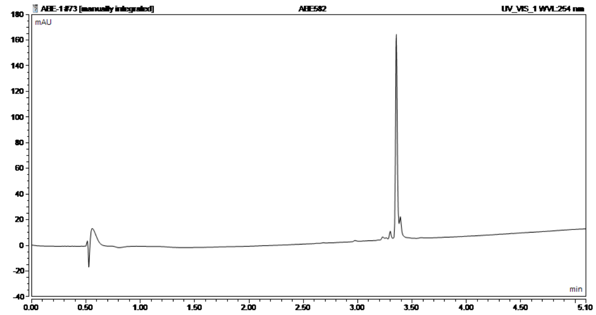

20

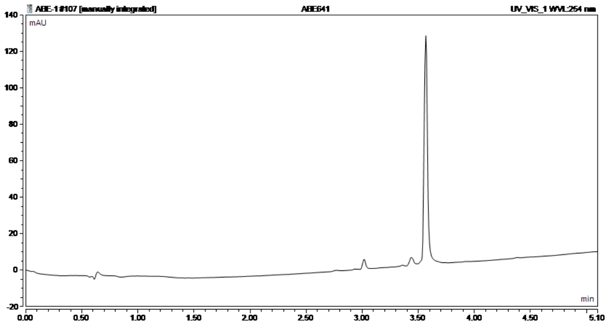

22

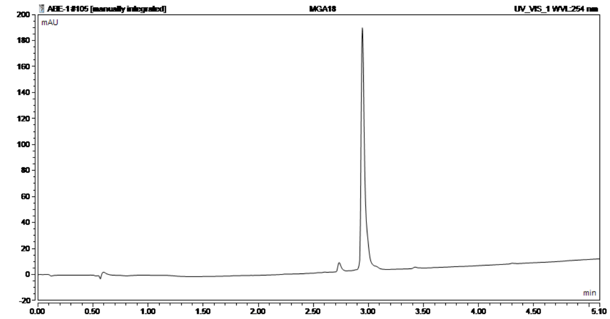

23

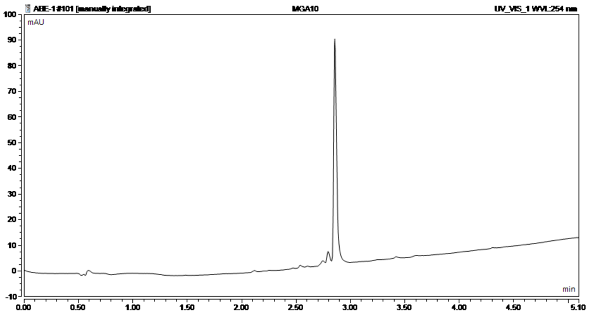

24

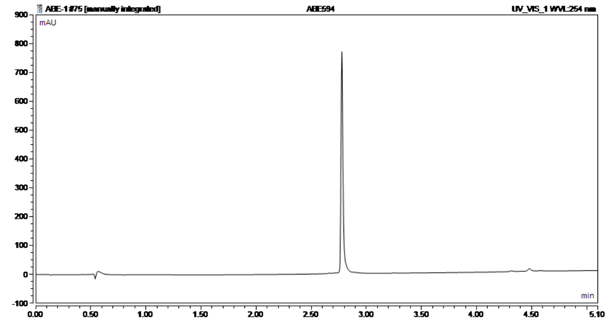

25

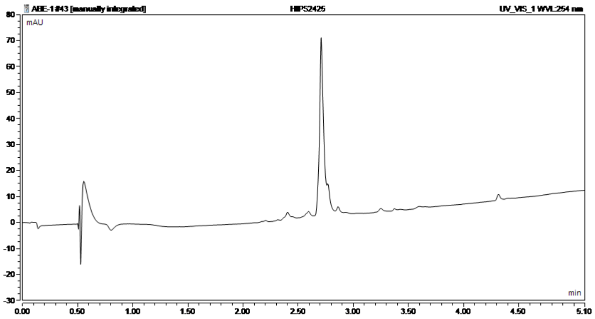

26

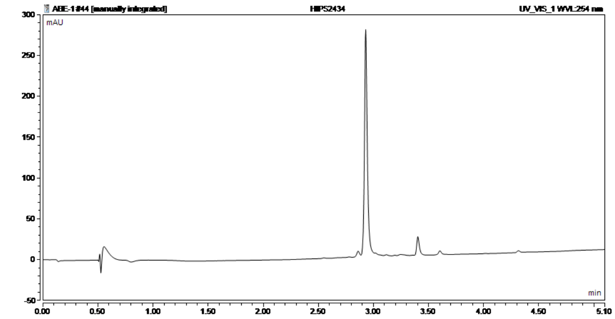

27

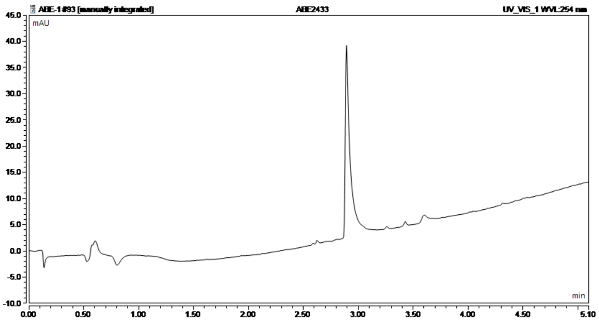

28

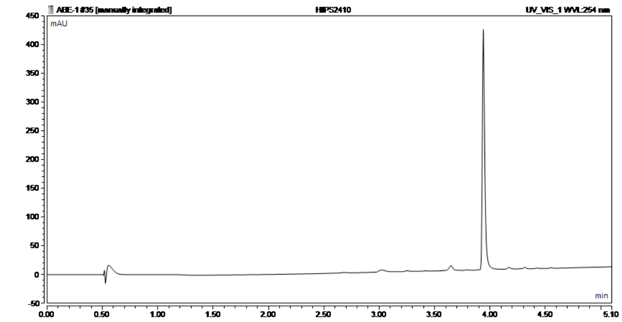

29

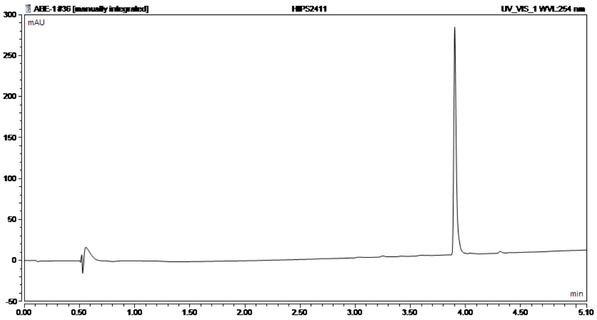

30

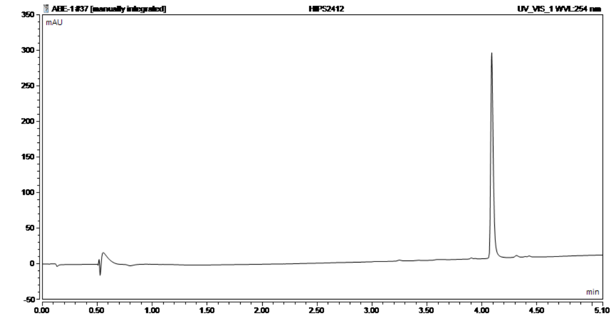

31

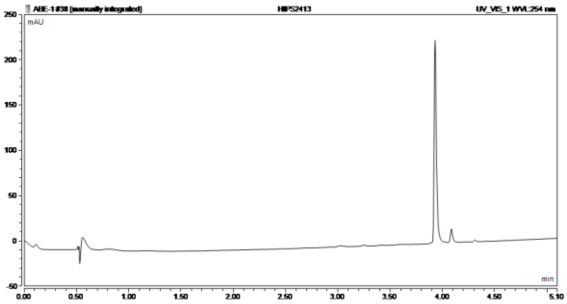

32

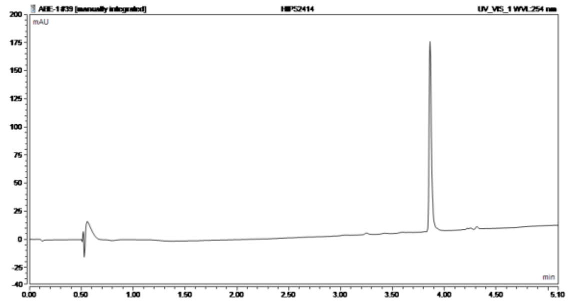

33

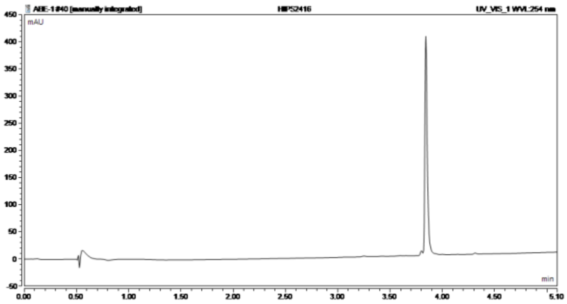

34

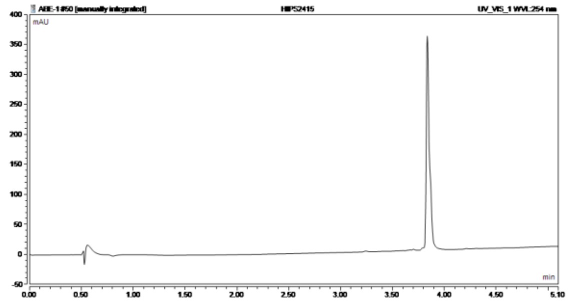

35

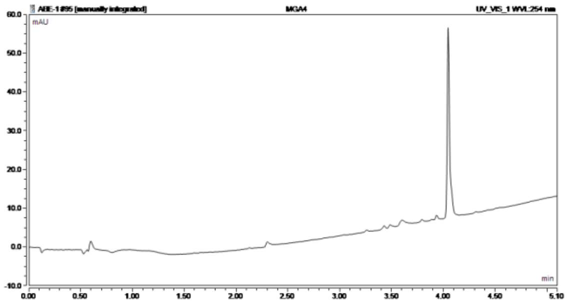

36

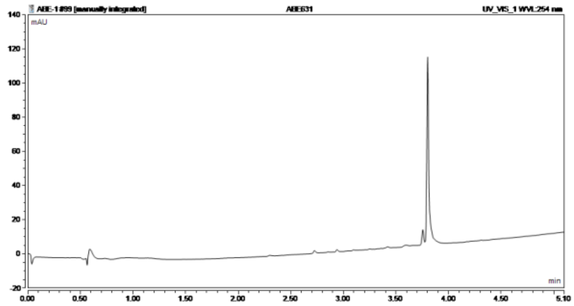

5. References

[1] P. Kirsch, S. C. Stein, A. Berwanger, J. Rinkes, V. Jakob, T. F. Schulz, M. Empting, *European journal of medicinal chemistry* **2020**, *202*, 112525.

[2] P. Kirsch, V. Jakob, K. Oberhausen, S. C. Stein, I. Cucarro, T. F. Schulz, M. Empting, *Journal of medicinal chemistry* **2019**, *62*, 3924.

[3] J. Hellert, M. Weidner-Glunde, J. Krausze, U. Richter, H. Adler, R. Fedorov, M. Pietrek, J. Rückert, C. Ritter, T. F. Schulz et al., *PLoS pathogens* **2013**, *9*, e1003640.

[4] A. Grundhoff, D. Ganem, *Journal of virology* **2003**, *77*, 2779.
